# Self-Immolative System for Disclosure of Reactive Electrophilic Alkylating Agents: Understanding the Role of the Reporter Group

**DOI:** 10.1021/acs.joc.1c00996

**Published:** 2021-07-22

**Authors:** Alexander G. Gavriel, Flavien Leroux, Gurjeet S. Khurana, Viliyana G. Lewis, Ann M. Chippindale, Mark R. Sambrook, Wayne Hayes, Andrew T. Russell

**Affiliations:** † Department of Chemistry, University of Reading, Whiteknights, Reading RG6 6AD, U.K.; ‡ CBR Division, Defence Science & Technology Laboratory (Dstl), Porton Down, Salisbury, Wiltshire SP4 0JQ, U.K.

## Abstract

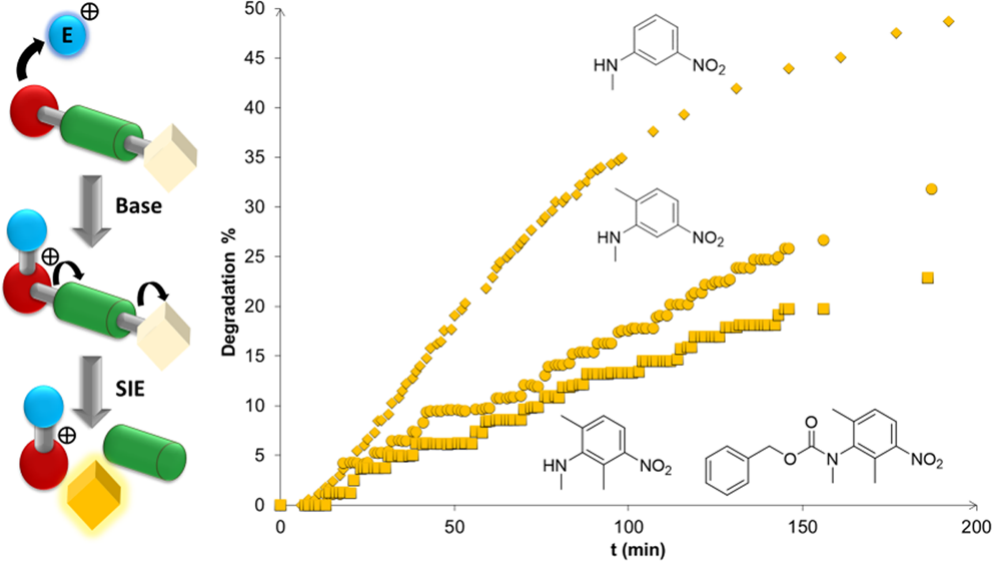

The development of stable, efficient chemoselective self-immolative systems, for use in applications such as sensors, requires the optimization of the reactivity and degradation characteristics of the self-immolative unit. In this paper, we describe the effect that the structure of the reporter group has upon the self-immolative efficacy of a prototype system designed for the disclosure of electrophilic alkylating agents. The amine of the reporter group (a nitroaniline unit) was a constituent part of a carbamate that functioned as the self-immolative unit. The number and position of substituents on the nitroaniline unit were found to play a key role in the rate of self-immolative degradation and release of the reporter group. The position of the nitro substituent (*meta*- vs *para*-) and the methyl groups in the *ortho*-position relative to the carbamate exhibited an influence on the rate of elimination and stability of the self-immolative system. The *ortho*-methyl substituents imparted a twist on the N–C (aromatic) bond leading to increased resonance of the amine nitrogen’s lone pair into the carbonyl moiety and a decrease of the leaving character of the carbamate group; concomitantly, this may also make it a less electron-withdrawing group and lead to less acidification of the eliminated β-hydrogen.

## Introduction

Stimuli-responsive compounds,^1−3^ such as self-immolative molecules and polymers,^4−7^ have become significant targets in organic-based material development as systems of this type offer enormous potential in a diverse range of applications that span drug delivery,^8−10^ biological and chemical sensors,^11,12^ diagnostics,^13^ and degradable polymers or degrade-on-demand adhesives.^14−19^ The seminal report by Katzenellenbogen and co-workers,^20^ based upon the “prodrug” concept discussed by Albert,^21^ set in place the key molecular design for self-immolative materials that have been explored and refined by several groups in recent years, one in which a substrate-specific trigger is coupled via a degradable linker group to a reporter moiety. Activation or deprotection of the trigger moiety, by either nucleophiles^4^ or electrophiles,^22−24^ renders the immolative linker unit labile via generation of an electron-rich center that then initiates a cascade of electrons and culminates in the release of the reporter group from the linker group. When designing a self-immolative system for a specific application, the delicate balance of reactivity and stability of each of the key components (*i.e*., trigger, linker, reporter) must be assessed and optimized. The system must feature a selective trigger unit to react with the desired substrate; equally, the linker to the reporter group must be sufficiently stable to withstand unwanted side reactions (such as environment-induced degradation) that can lead to “false-positive” results. The effect of the structure of the linker group upon self-immolative pathway and degradation rates has been shown to be pivotal in this design rationale.^25,26^ In a detailed study, Phillips and co-workers have elegantly shown^27^ the effect that the structure of aromatic self-immolative linkers has on a self-immolative process and were able to tune the controlled release of phenols under neutral conditions.

**Scheme 1:**
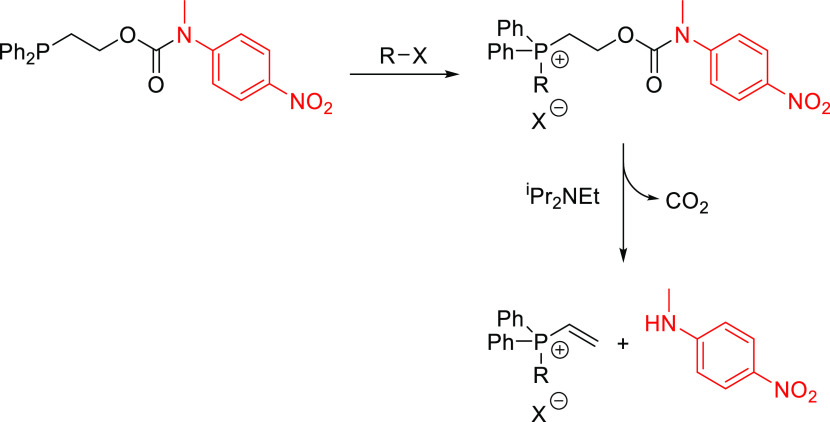
Self-Immolative System Triggered by Nonacidic, Electrophilic Alkylating Agents in the Presence of a Hindered Base

In a recent communication,^23^ we reported the first examples of selective solution-phase self-immolative systems triggered by a nonacidic electrophilic species, such as methyl, allyl, and benzylic halides, to afford a facile colorimetric visual disclosure of toxic electrophilic alkylating agents (Scheme 1).

These systems, based upon the rarely utilized carbamate-based 2-(methylthio)ethoxycarbonyl (Mteoc) protecting group developed^28^ by Kunz and the phosphorus analogue advanced by Verducci et al.,^29^ afforded a visual colorimetric response to such electrophiles following exposure to mild, basic conditions. A diphenylphosphine nucleophile was used as it offered the best balance between reactivity toward electrophiles while also offering reasonable stability toward aerobic oxidation. Recently, a related approach for detection of alkylating agents has been reported using a Förster resonance energy-transfer (FRET)-based profluorescent probe.^24^ In this system, alkylation of the probe results in the formation of a quaternary ammonium salt, which increases its leaving group capacity allowing the probe to facilitate the 1,6-elimination of the self-immolative process. When the elimination proceeds, the fluorophore is no longer quenched and can emit a fluorescence signal.

To the best of our knowledge, a systematic study on the effect of the structure of the reporter group (red moiety in Scheme 1) on self-immolative processes, embracing conformational and basicity variations, has not been reported in the literature. Indeed, in Katzenellenbogen’s seminal paper,^20^ and in a later paper by Shabat,^4b^ the nature of the reporter group was considered to have only a limited impact on the rate of self-immolative elimination of their carbamate systems. A related observation was made in respect of carbonate linkers highlighted by Schmidt and Jullien; interestingly, consideration was also given to conformational effects in the leaving group.^4c^ Hay has varied the substituent in the *para*-position of an aniline reporter group that fragmented from a 4-hydroxylaminobenzyl alcohol linker, observing that the extent of release was not affected by its leaving group ability, but rather by its subsequent reactions.^4d^ In this paper, we describe a systematic study that reveals valuable information on the role of the reporter group in relation to how this key component influences the self-immolative molecule’s stability and degradation rate.

## Results and Discussion

**Figure 1:**
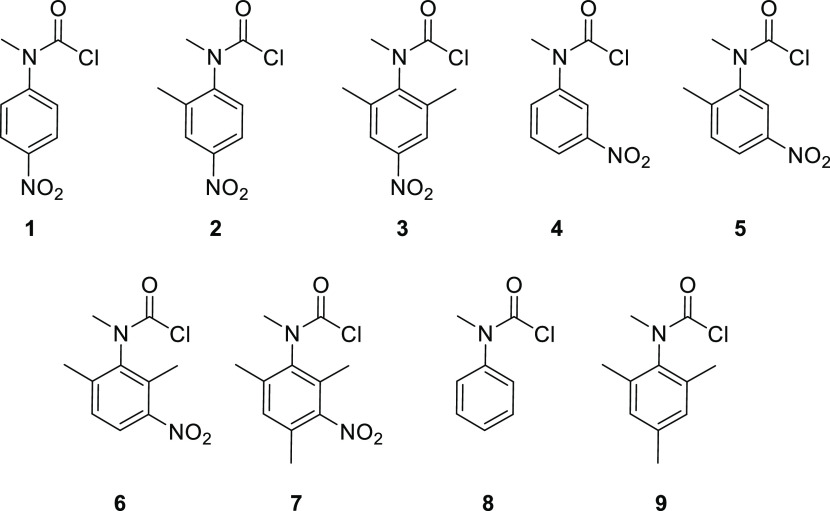
Structure of carbamoyl chlorides **1**–**9**.

In view of employing self-immolative systems in real-world environments for the specific disclosure of toxic alkylating agents, it is important that these materials possess suitable shelf-lives and are resistant toward fluctuations in ambient conditions (such as moisture and/or temperature) while stored. We have recently reported a novel self-immolative system^23^ for the selective disclosure of reactive electrophilic alkylating agents that is stable in solution (>72 h in CD_3_CN) and when exposed to water (10% D_2_O in CD_3_CN). However, disappointingly, release of the reporter group was observed when stored in the bulk. To study the influence of the reporter group structure on the stability and reactivity of the self-immolative system, a variety of analogue structures were prepared; these analogues have been designed to probe both electronic and steric effects. To this end, carbamoyl chloride precursors **1**–**9** were first synthesized (see Figure 1).

**Scheme 2:**
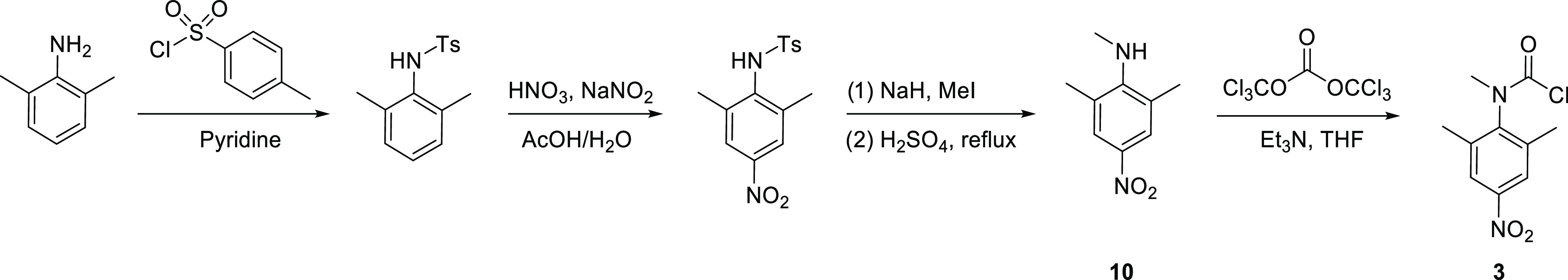
Synthesis of Carbamoyl Chloride **3**

Carbamoyl chloride **3**, substituted by a nitro group in the *para-*position relative to the carbamoyl group, was synthesized according to the route displayed in Scheme 2.^30,31^ Protection of the amine group using 4-toluenesulfonyl chloride (TsCl) was carried out prior to nitration to promote regioselective reaction *para*- to the *N*-tosylate group. The carbamoyl chloride **3** was then obtained via a two-pot, three-step process; that involved initial *N-*methylation of the protected aniline followed by deprotection of the tosylate group under acidic conditions to yield **10**, which was then converted to the corresponding carbamoyl chlorides using triphosgene (see Figures S1–S4 in the Supporting Information (SI) for analytical data for **10** and **3**).

**Scheme 3:**
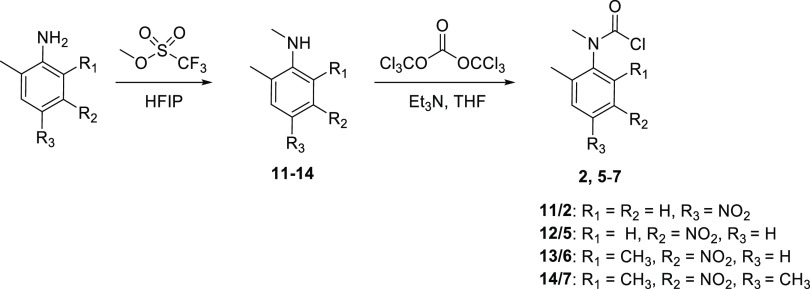
Synthesis of Carbamoyl Chlorides **2** and **5**–**7**

The carbamoyl chlorides **2** and **5**–**7** were synthesized utilizing an alternative synthetic pathway (Scheme 3). Regioselective nitration of the anilines leading to **5**–**7** afforded the corresponding *meta*-derivatives (2-methyl-4-nitroaniline was purchased), and then selective monomethylation was carried out according to the procedure reported by Lebleu et al.,^32^ using methyl triflate as an alkylating agent in hexafluoroisopropanol (HFIP), affording the desired *N*-methylated aniline products (**11**–**14**) in yields ranging from 68 to 83%. As in the case of **3**, the carbamoyl chlorides **2** and **5**–**7** were then obtained by reaction of the *N*-methylated aniline derivatives with triphosgene (see Figures S5–S20 in the SI for analytical data for **11–14** and **2**, **5**–**7**). The carbamoyl chlorides **1**, **4**, **8**, and **9** were obtained using procedures reported previously (see Figures S21–S28 in the SI for analytical data for **1**, **4**, **8**, and **9**).^31,33,34^

**Scheme 4:**
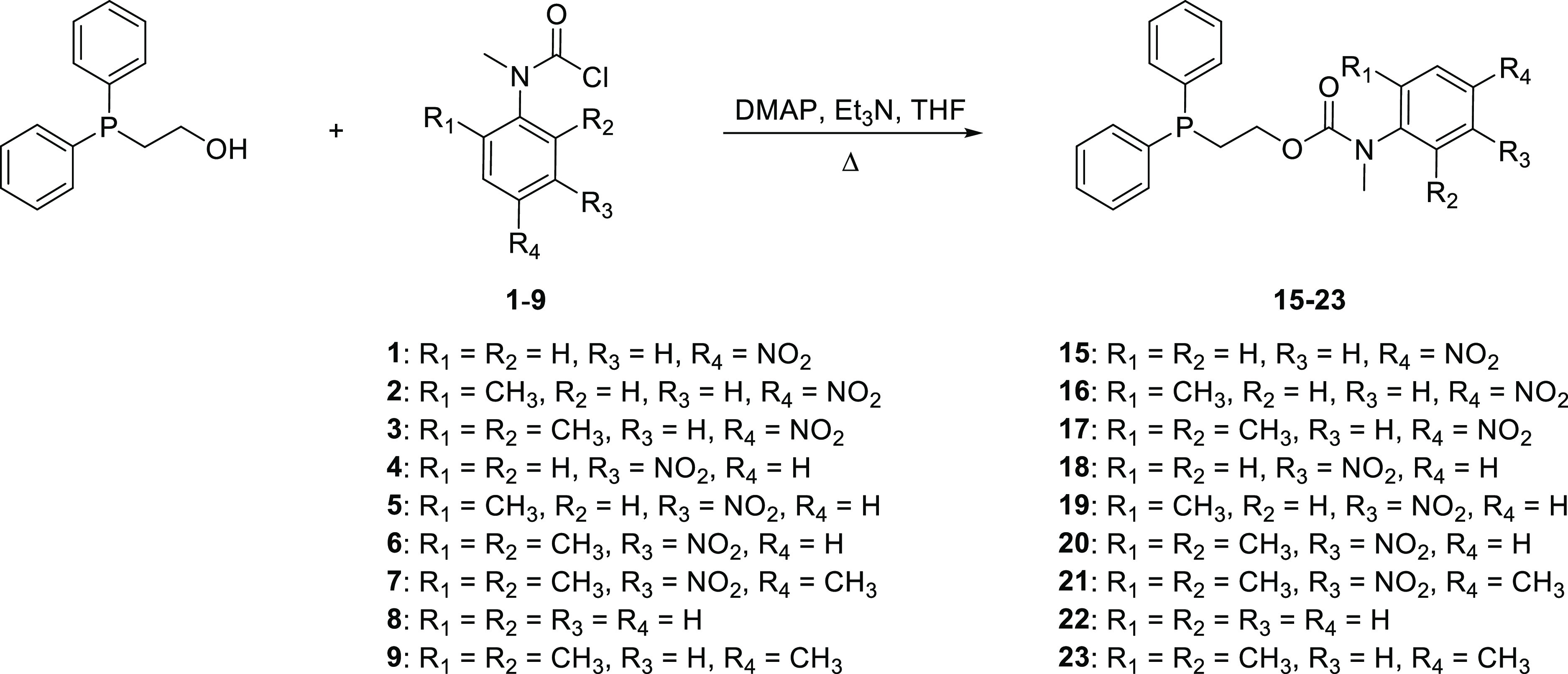
Synthesis of the Self-Immolative Systems **15**–**23**

The target self-immolative systems were obtained by conjugation of 2-diphenylphosphinoethanol to the *N*-methyl carbamoyl chloride derivatives **1**–**9** using 4-dimethylaminopyridine (DMAP) in a catalytic amount and heating under reflux in tetrahydrofuran (THF): this procedure afforded the self-immolative systems **15**–**23** (Scheme 4; see Figures S29–S60 in the SI for analytical data for **15**–**23**).^23^

**Figure 2:**
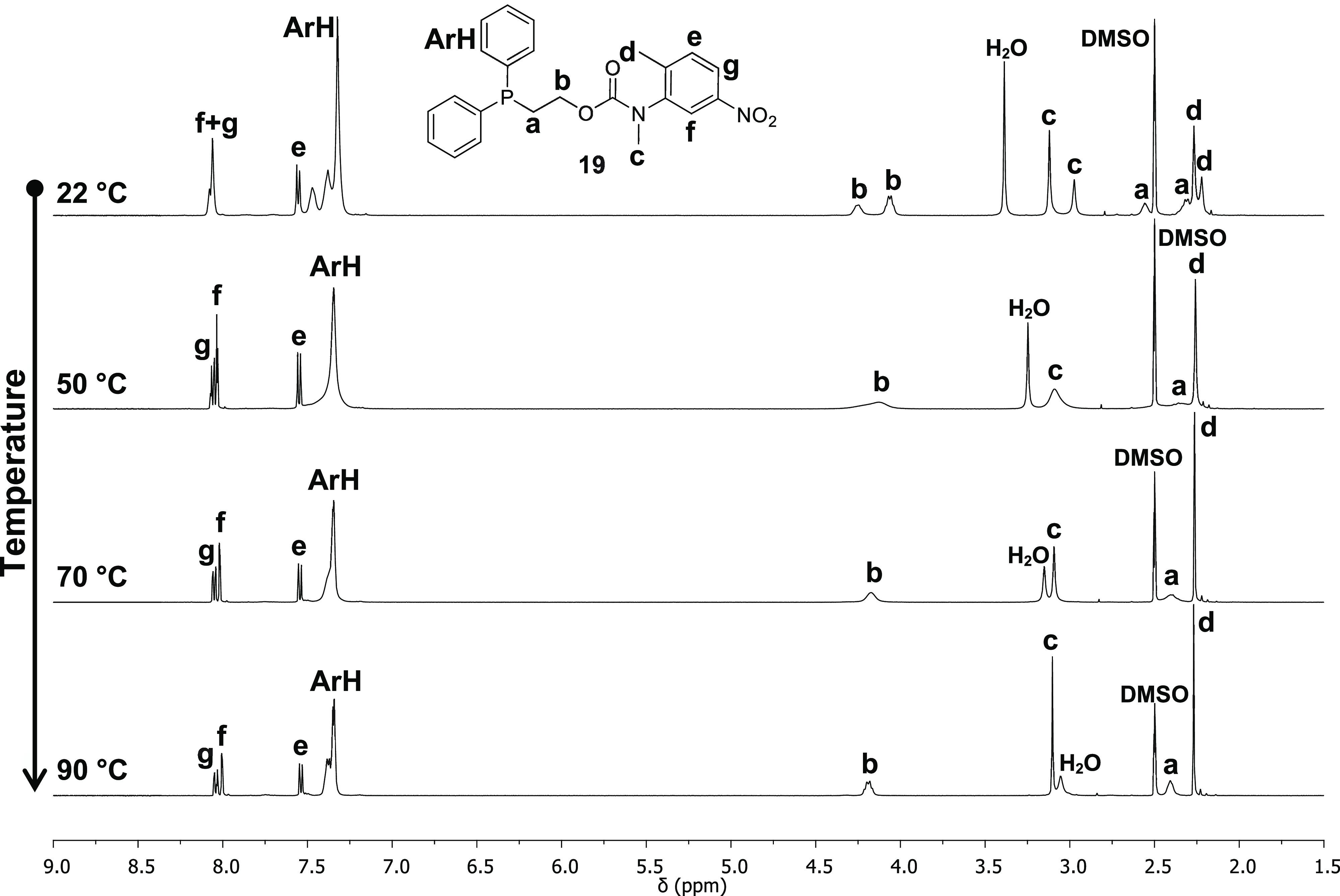
VT ^1^H NMR spectroscopic analysis of self-immolative system **19**: rotamer ratio 62/38 recorded in DMSO-*d*_6_ (500 MHz).

**Scheme 5:**
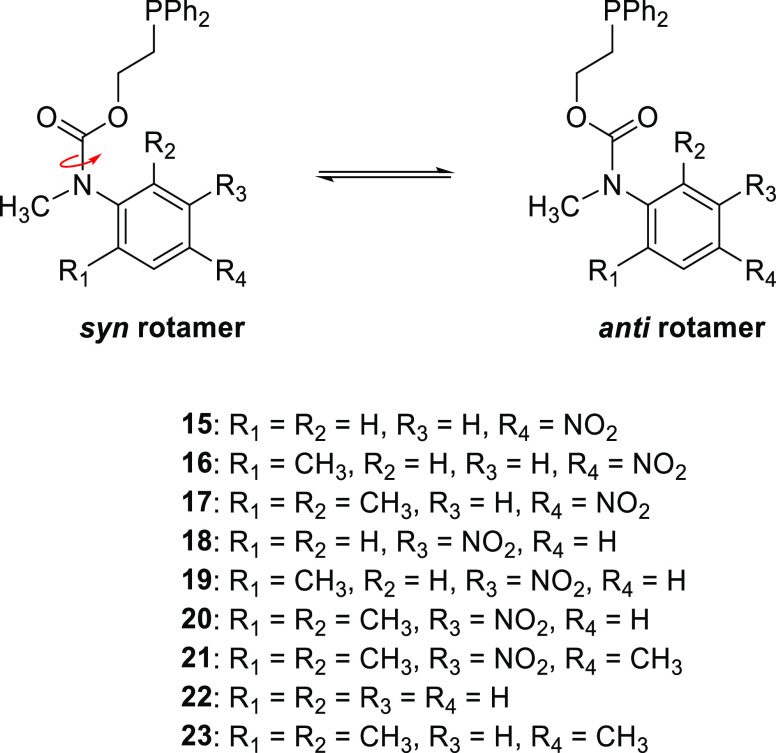
*Syn–anti* Rotamer Equilibrium Caused by the Restricted Rotation of the Carbamate C–N Bond

NMR spectroscopic analysis of the different self-immolative systems containing one or two methyl groups *ortho*- to the carbamate revealed the presence of two rotamers as a result of restricted rotation about their carbamate C–N bonds (see Scheme 5), and it is evident that the rotamer ratios for these disclosure systems were perturbed by changing the solvent (*e.g*., compound **19**; see Figure 2).^a^ In addition, the rotameric ratio increased with the number of electron-donating methyl groups present on the aromatic ring (see Table 1: **19** vs **20** vs **21**).^35^

**Table 1:**
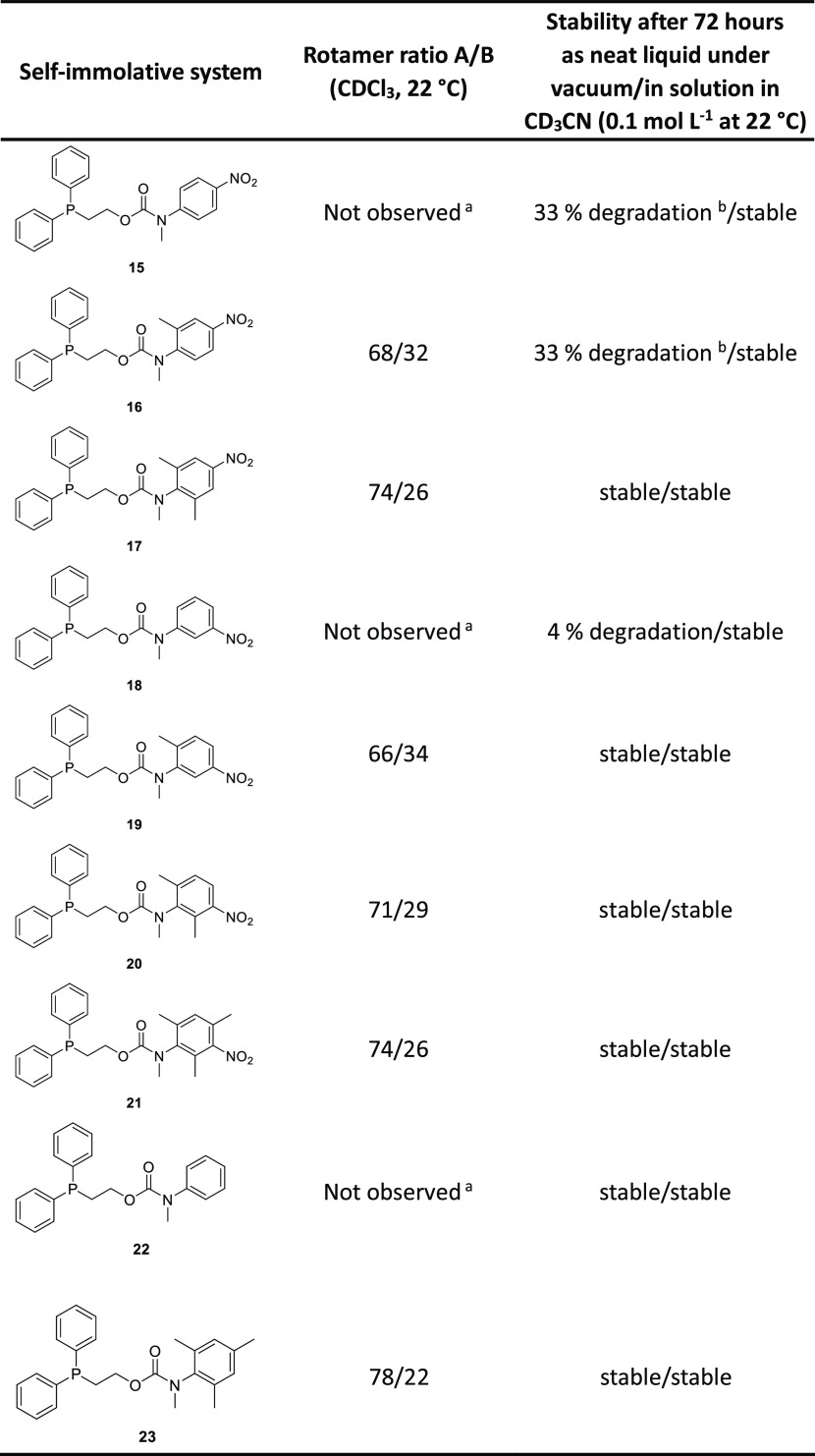
Rotamer Ratios and Stability Information in Neat/Solution Form for the Self-Immolative Systems **15**–**23**, Determined Using ^1^H NMR Spectroscopic Analysis (Errors Associated with the Integral Values Were ±5%)

a Rotameric ratio is not determined as the coalescence temperature is below 22 °C.

b The observed degradation occurs within the first 24 h, after which the physical state of the neat liquid changes to a paste-like consistency.

Since the energy barrier to free rotation of the C–N bonds of the carbamate group can be overcome at elevated temperatures, variable-temperature (VT) ^1^H and ^31^P NMR spectroscopic studies were conducted on **16**, **19**, and **20** to specifically facilitate two pairwise comparisons, **16**/**19** and **19**/**20** (see Figures S61–S65). The pair **16**/**19** was selected to evaluate the relative effect of a *para*- and *meta*-positioned nitro group on the double-bond character of the nominally C–N single bond of the carbamate, whereas **19**/**20** permitted the effect of one vs two *ortho*-methyl groups to be assessed. These results could then be correlated to the observed stability/reactivity of the systems. Thus, **16** and **19** were analyzed in DMSO-*d*_6_ in increments between 25 and 90 °C: Figure 2 shows the ^1^H NMR spectra obtained for the self-immolative system **19** at four selected temperatures. The resonances at 2.98–3.12 ppm (**c**, C*H*_3_–N), 4.06–4.25 ppm (**b**, C*H*_2_–O), and 8.06 ppm (**f** and **g**, C*H*_Ar_) began to broaden as the temperature was increased and eventually coalesced by *ca*. 50 °C into two single broad resonances. Meanwhile, the resonances corresponding to the aromatic protons C*H*_Ar_**f** and **g** sharpened from a broad signal to a doublet for proton **f** and a doublet of doublets for proton **g**. Upon heating to 90 °C, the resonances corresponding to the protons C*H*_3_–N and C*H*_2_–O sharpened into a singlet and an apparent quartet, respectively. Compound **16** showed similar spectroscopic changes but with coalescence being achieved at *ca*. 40 °C (see Figures S62 and S63). The determination of the coalescence temperature (*T*_c_), in addition to the value of the rotamer population difference (Δ*P*) and the limiting chemical shift difference (Δν), obtained from spectra at temperatures below coalescence for both **16** and **19** allowed the calculation of the free energy of activation (Δ*G*^‡^) for the rotational equilibrium of **16** and **19** by treating the data as an exchange between two unequally populated sites (see eqs S1–S6 in the SI).^36^ Lower values of the energy barriers were found for **16** (Δ*G*_C,rotamer A_^‡^ = 65.2 kJ·mol^–1^ and Δ*G*_C,rotamer B_^‡^ = 64.0 kJ·mol^–1^, respectively) when compared to **19** (Δ*G*_C,rotamer A_^‡^ = 67.8 kJ·mol^–1^ and Δ*G*_C,rotamer B_^‡^ = 66.5 kJ·mol^–1^, respectively), showing the influence of the nitro group position (*para*- vs *meta*-) on the carbamate C–N rotation equilibrium. As anticipated, the ability of the nitrogen lone pair to resonate into the carbamate increases when the nitro group was moved from the *para*- to the *meta*-position, consistent with the change in coalescence temperature.

**Figure 3:**
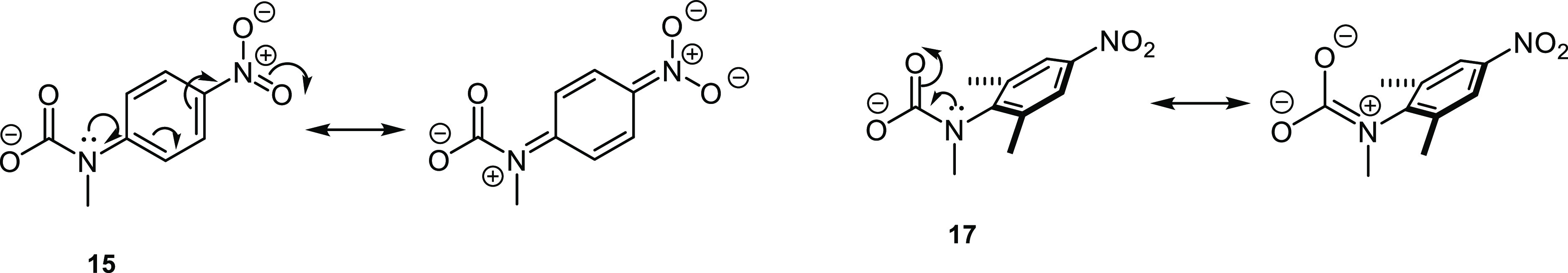
Resonance structures contributing to the stability of the leaving groups of **15** and **17**.

In contrast to **19**, **20** revealed only broadening of the key resonances (see Figures S64 and S65), without coalescence at 100 °C, indicating a greater resonance of the nitrogen lone pair into the carbamate carbonyl and a larger steric barrier to rotation (Δ*G*_C,rotamer A_^‡^ > 79.3 kJ·mol^–1^ and Δ*G*_C,rotamer B_^‡^ > 77.3 kJ·mol^–1^, respectively) when compared to **19**. A similar trend was observed by comparing the VT ^1^H and ^31^P NMR spectroscopic analysis obtained for **16** and **17** (with the nitro group in the *para*-position) (see Figures S62–S63 and S66–S67). The reduced resonance into the aromatic ring arises from a substantial torsion angle between the ring and the carbamate group (Figure 3).

To investigate the thermal stability of these self-immolative systems, the solutions were then cooled down from 100 to 25 °C and the sharp, well-resolved resonances observed at an elevated temperature for **16**, **17**, **19**, and **20** returned to their original shape, indicating their thermal stability across this temperature range (see Figures S68–S75). In addition, the different self-immolative systems **15**–**23** were found to be stable at both 0.1 and 0.025 M (reaction conditions for the self-immolative study) solutions in CD_3_CN over >72 h at 22 °C (see Figures S76–S84). However, a small amount of oxidation to the corresponding phosphine oxide was noted.

The stability of the different self-immolative systems as neat liquids was then investigated (see Table 1). The stability of **15**, bearing a nitro group in the *para*-position relative to the carbamate group, was first assessed. When left to stand at room temperature as a neat liquid, it proved to be unstable and release of the reporter group (*N*-methyl-*para*-nitroaniline) was observed, with 32% degradation evident after 24 h (see Figure S85). Lowering the storage temperature to −20 °C was ineffective in preventing degradation. The instability of **15** was attributed to the structure of the *N*-methyl-*para*-nitroaniline reporter unit; in particular, the resonance of the nitrogen lone pair into the aromatic ring and nitro group rendered the carbamate less electron-rich and more electrophilic. In contrast, the self-immolative system **18**, with the nitro group in the *meta*-position (wherein its influences are limited to inductive and field effects), proved more stable. However, while the stability of **18** improved relative to **15**, the observed release of 4% of the reporter group (*N*-methyl-*meta*-nitroaniline) over 72 h would still risk a false-positive response if used in real-world applications (see Figure S86). Removal of the nitro group afforded phenyl carbamate **22** that proved to be stable under identical conditions (see Figure S87). These observations indicated the influence of the presence of the nitro group on the carbamate unit stability. However, as a colorimetric response is required for visual disclosure, the presence of the nitro group was essential in the reporter moiety. Therefore, an alternative route to enhance the stability of the self-immolative system was investigated that involved the introduction of methyl groups in the *ortho*-position of the reporter unit (relative to the carbamate linkage). Introduction of the methyl groups in the *ortho*-position of the reporter group should impart a twist on the N–C (aromatic) bond, reducing the ability of the lone pair of the carbamate nitrogen to resonate into the aromatic ring (Figure 3). Indeed, resonance into a carbamate anion was noted by Johnson as significant in determining the p*K*_a_ of its conjugate acid.^37^

When added to the increase in steric size of the reporter group, this should have the effect of reducing the carbamate’s electrophilicity. Regarding the self-immolative system **16**, featuring a single *ortho*-methyl group and the nitro group in the *para-*position, relative to the carbamate group, a decrease of degradation rate was observed when compared to **15**. However, after 72 h, a similar amount of reporter group release was observed for **16** and **15** (see Figures S85 and S88). In contrast, regarding the self-immolative system **19**, with the nitro group located in the *meta*-position and a single methyl group in the *ortho*-position, relative to the carbamate group, degradation was not observed after 72 h (see Figure S89). In the cases of the self-immolative systems **17**, **20**, **21**, and **23**, substituted by two *ortho*-methyl groups, degradation was not observed at room temperature after 72 h (see Figures S90–S93). These results highlight the strong influence of the presence of the methyl groups in the *ortho*-position, relative to the carbamate group, on the stability of the self-immolative system.

These effects will also bear upon the ability of the carbamate to function as a leaving group in the manner identified in Schemes 1 and 8 and Figure 5. However, it is worth noting that such effects will be attenuated as compared to the leaving group ability of the aniline itself. Johnson has noted that while the p*K*_a_H^+^ of the amine in a carbamate can vary by as much as 8 p*K*_a_ units, the corresponding carbamic acids vary by around 1 p*K*_a_ unit.^37^ However, though the differences are attenuated, they do vary in the same direction as the amine p*K*_a_H^+^.

**Scheme 6:**
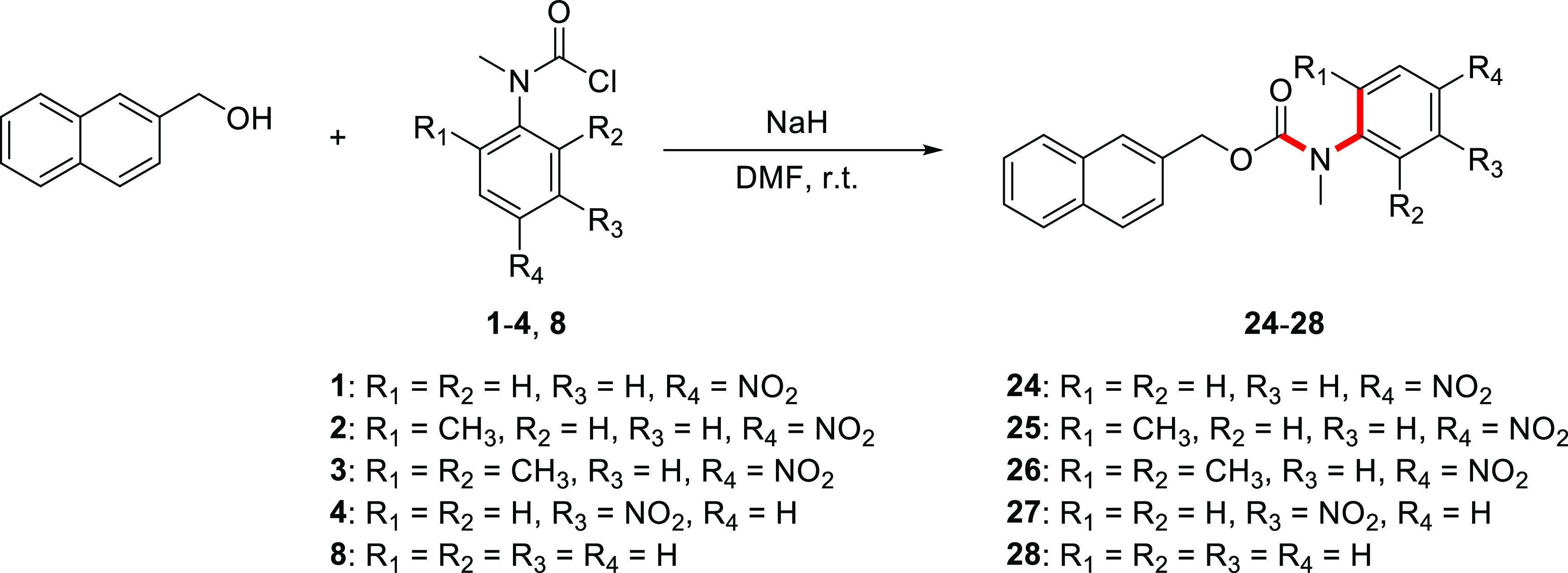
Synthesis of the Crystalline Carbamate Model Compounds **24**–**28**

To quantify the torsion angle imparted on the CCNC moiety, as highlighted in red (Scheme 6), the model compounds **24**–**28** that possess similar structures to the self-immolative systems **15**, **16**, **17**, **18**, and **22** were synthesized. 2-Naphthalenemethanol was selected as a result of its crystalline nature. Crystalline carbamates were obtained via the conjugation of 2-naphthalenemethanol to *N*-methyl-carbamoyl chloride derivatives **1**–**4** and **8** using sodium hydride (NaH) in dimethylformamide (DMF) (see Figures S94–S103).

The solid-state structures of **22**–**28** were obtained using X-ray crystallographic analysis. Partial structures are shown in Figure 4 to emphasize the CCNC torsion angle (for full structures, see Figures S104–S110 and Tables S2–S8).^b^

**Figure 4:**
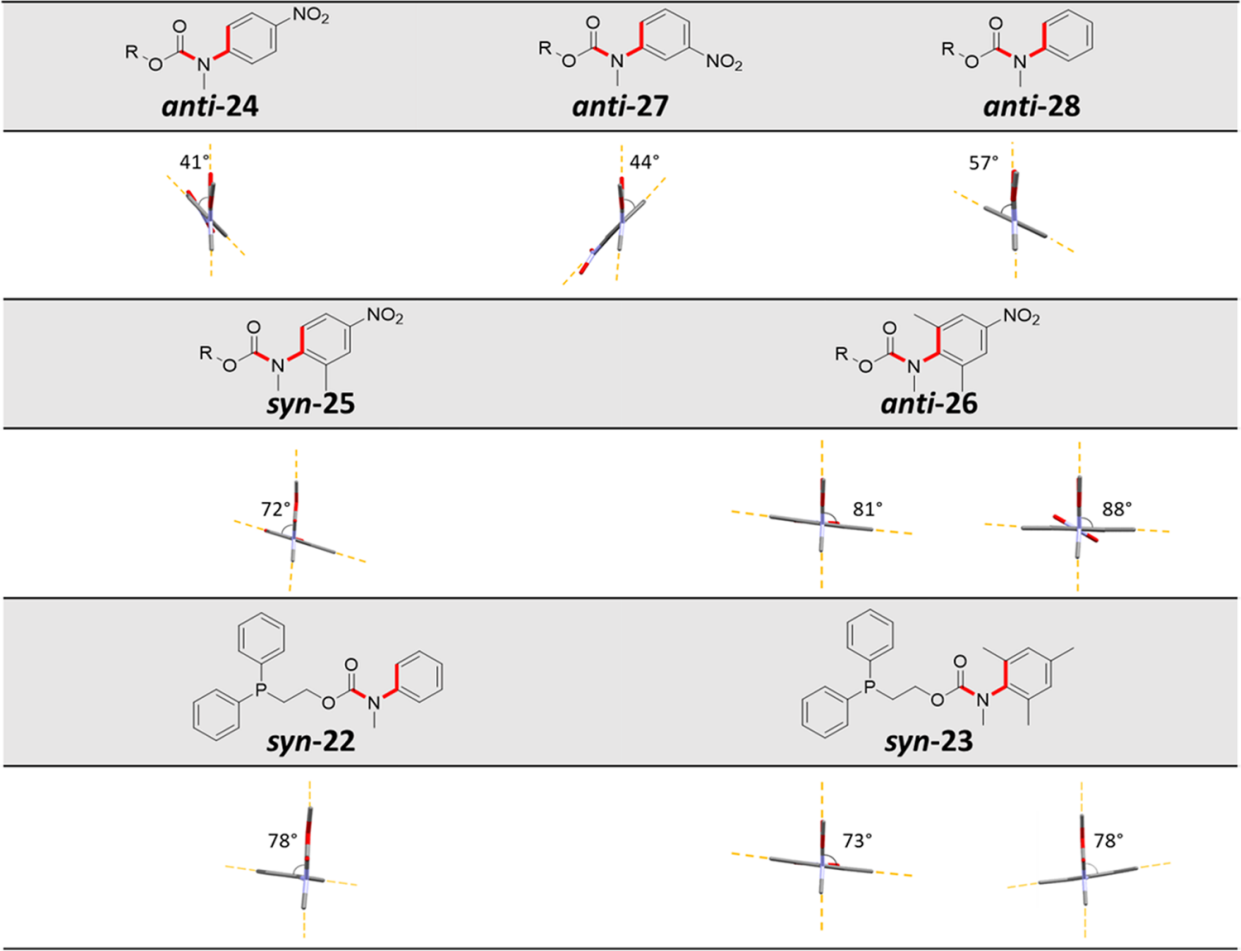
Partial solid-state structures of model compounds **22**–**28**.

The analysis showed no significant changes in geometry when the nitro group was located in either the *para*- or the *meta*-position, with torsion angles of 41(1) and 44(1)° for **24** and **27**, respectively. However, when the nitro group was not present (*e.g*., **28**), a higher torsion angle of 57(1)° was observed. In the cases of **24** and **27**, the torsion angles reflect the ability of the CCNC linkage to adopt a more planar geometry as a consequence of electronic effects when the electron-withdrawing nitro group is present. These results are in good agreement with the stability profiles observed for the self-immolative systems **15**, **18**, and **22** in their neat liquid form. In contrast to **24** and **27**, the solid-state structures of **25** and **26** that possess one and two *ortho*-methyl groups, respectively, revealed an increase in the torsion angle to 72(1)° for **25** and 81(1) or 88(1)° for **26** (two conformers were evident in the unit cell). Assuming that the solid-state data of the model carbamates translates to the self-immolative system in their liquid form, the additional twist imparted on the N–C (aromatic) bond by the presence of *ortho*-methyl groups should lead to a reduction in the electrophilicity of the carbamate linker. These effects could serve to explain the improved stability, in their neat liquid forms, of the self-immolative systems **17** when compared to that of **15** as well as the improved stability of **19** and **20** when compared to that of **18**.

The solid-state structures of the self-immolative systems **22** and **23** were also obtained. The CCNC torsion angle for **23** (that features two *ortho*-methyl groups but no nitro residue) was determined to be 73(1) or 78(1)° (two conformers were evident in the unit cell), comparable in value relative to that of the model carbamate **26** (81(1) or 88(1)°). However, the CCNC torsion angle of the phenyl carbamate **22** (78(1)°) was found to be higher than expected when compared to that of the model compound **28**; the difference between the torsion angles values could be attributed to crystal packing forces involving the planar aromatic units.

**Scheme 7:**
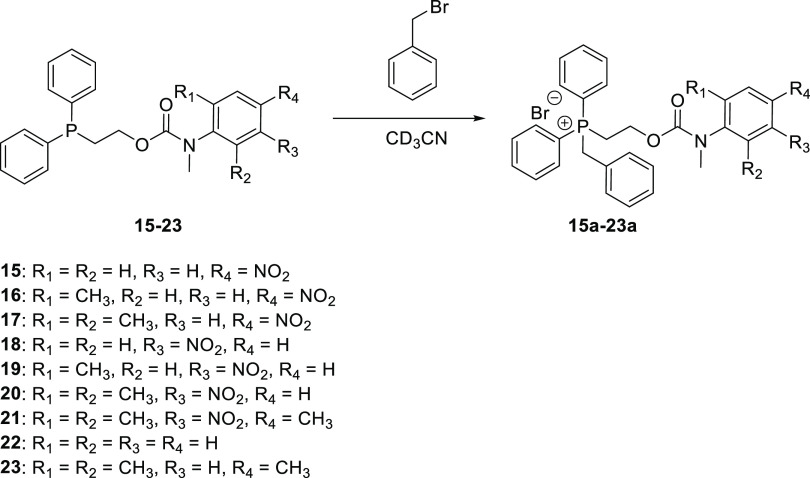
Alkylation of Self-Immolative Systems **15**–**23** Using BnBr as an Alkylating Agent in CD_3_CN

To assess the ability of the different self-immolative systems to disclose the presence of reactive electrophilic alkylating agents via a two-step process involving alkylation followed by elimination/decarboxylation (Scheme 1), the reactivity of **15**–**23** toward alkylation was first investigated, using benzyl bromide as the alkylating agent (Scheme 7).^38,39^

Cognizant that these are type II S_N_2 reactions,^38,39^ alkylation of self-immolative systems **15**–**23** was conducted by dissolution of the self-immolative system in the polar aprotic solvent CD_3_CN, followed by the addition of 10 molar equiv of benzyl bromide and recording the ^1^H NMR spectra at regular time intervals at 20 °C (see Figures S111–S119). Following analysis of the reactions under these pseudo-first-order conditions, it was found that the introduction of different substituents on the aromatic ring of the reporter moiety had no dramatic influence on the alkylation rate, with half-life times (*t*_1/2_) measured between 10.4 and 28.6 min (see Table 2 and Figure S120). Given that there is a developing positive charge at the phosphorus center, during alkylation, it is understandable that self-immolative systems with otherwise equivalent reporter groups, but with the nitro group *meta*- to the carbamate alkylate faster than those with it in the *para*-position, *e.g*., **18** vs **15**. Incorporation of *ortho*-methyl groups relative to the carbamate slowed the rate of alkylation, *e.g*., **18** vs **19**, vs **20**. Also evident was the slower alkylation of the nitro-substituted aromatic derivatives in comparison to those systems without nitro substituents, *e.g*., **22** vs **18**. Whether this arises purely by a charge–dipole interaction between the developing phosphonium center and the carbamate, or a more specific alignment of the developing σ*(C–P) orbital is not clear.

**Table 2:**
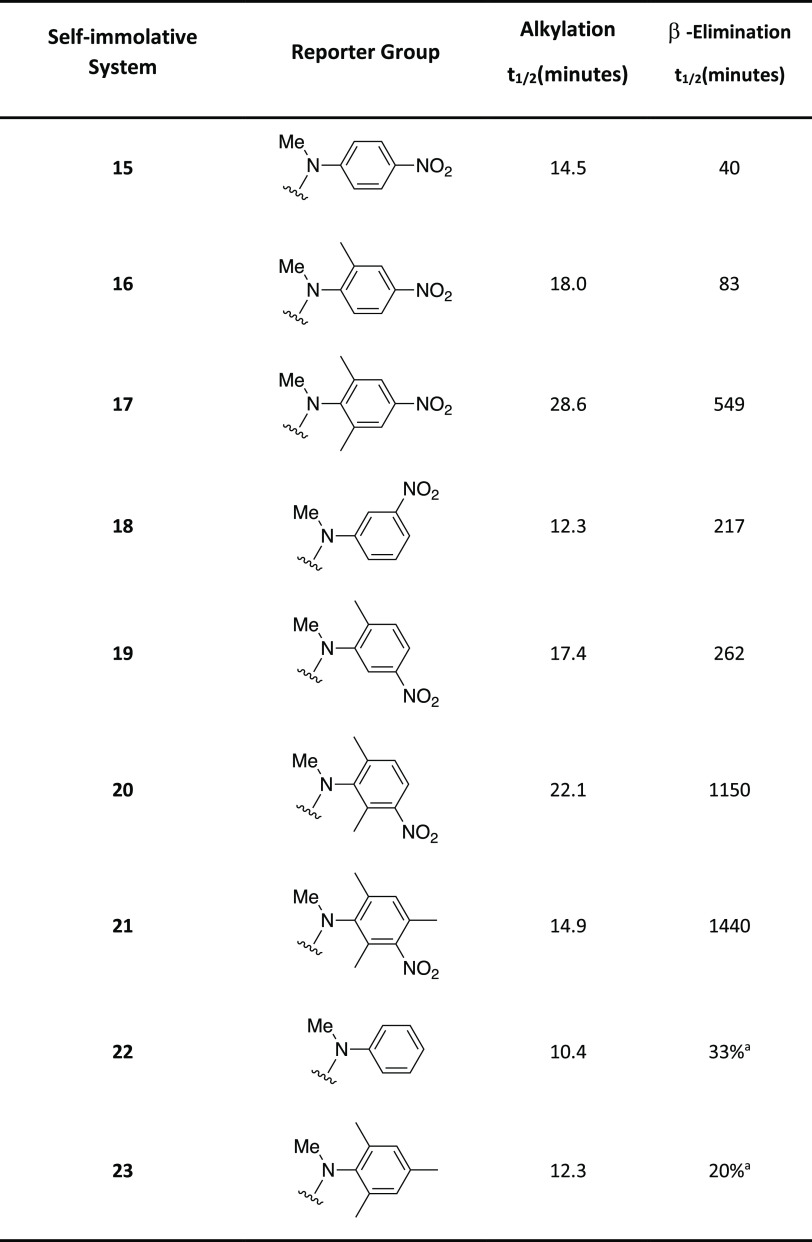
Alkylation Rate Data Obtained Following the Addition of Benzyl Bromide to the Self-Immolative Systems **15**–**23** and β-Elimination Rate Data Obtained Following the Addition of 2 molar equiv of DIPEA to Alkylated Self-Immolative Systems **15a**–**23a** Calculated Using ^1^H NMR Spectroscopic Data at 20 °C (Errors Associated with the Integral Values were ±5%)

a Percentage of β-elimination after 24 h.

**Scheme 8:**
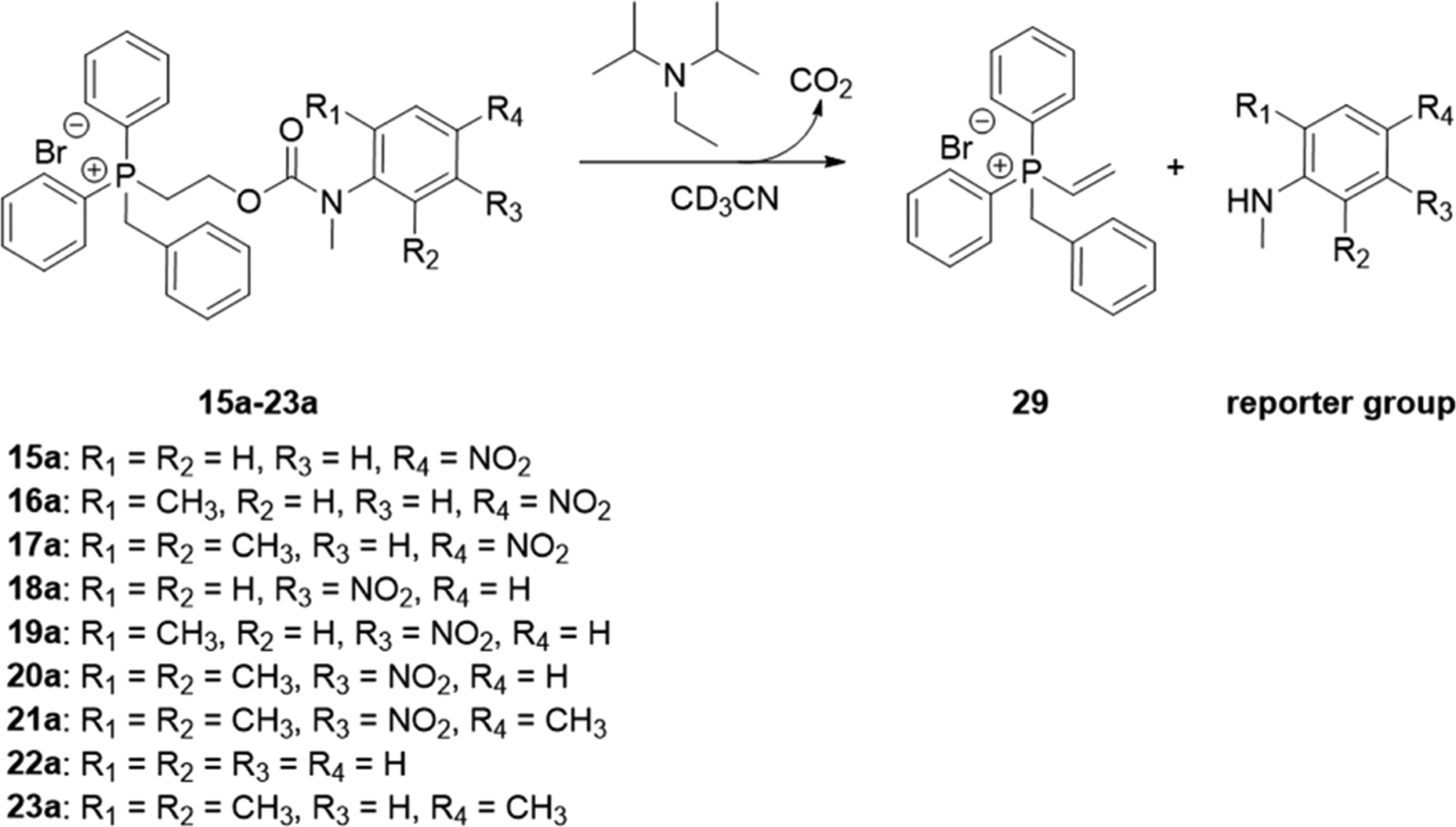
Degradation of Alkylated Self-Immolative Systems **15a**–**23a** using 2 molar equiv of DIPEA as a Base in CD_3_CN

The β-elimination of the alkylated systems **15a**–**23a** was then performed using 2 molar equiv of *N*,*N*-diisopropylethylamine (DIPEA) (see Figures S121–S131). All compounds underwent the expected elimination to produce the reporter unit and CO_2_ (Scheme 8). When the elimination of **21** was conducted in D_2_O/CD_3_CN, at partial conversion, no incorporation of deuterium was observed (^1^H NMR) in the PC**H**_2_CH_2_ resonance of residual **21** or the vinyl group of phosphonium salt **29**. However, both species were deuterated at the benzylic methylene. This limits the mechanism to either E2, E1cB (anion), or E1cB (irreversible).

In contrast to the alkylation profiles, the rate of elimination dramatically changed with respect to the structure of reporter groups (see Table 2). The highest elimination rates were observed for the reporter groups most able to withdraw electron density from the carbamate nitrogen, thus affording both the best leaving group and, concomitantly, acidifying the β-hydrogen removed during the elimination reaction. Therefore, for equivalently substituted reporter groups bearing the same substituents but located in different positions on the aromatic ring, a *para*-nitro substituent is more effective than a *meta*-substituent, *e.g*., **15a** vs **18a**, whereas additional methyl groups slowed the elimination, *e.g*., **18a** vs **19a** vs **20a**. In addition, these methyl groups are modestly electron donating and would be expected to restrict solvation of the leaving group. Other solvents were investigated to enhance the sensitivity of the alkylation and β-elimination pathways using **19** (see Figures S132–S136). The rate of alkylation varied with the solvent, with the observed order being DMSO-*d*_6_ > 10% MeOD/CD_3_CN > CD_3_CN > DMF; however, β-elimination was observed at a respectable rate in CD_3_CN in comparison to DMSO-*d*_6_ and DMF-*d*_7_. The variation in the rate of elimination in these three solvents may relate to changes in the p*K*_a_H^+^ for *^i^*Pr_2_EtNH^+^ in them. For example, p*K*_a_H^+^ Et_3_NH^+^ (CH_3_CN) > p*K*_a_H^+^ Et_3_NH^+^ (DMSO).^40^

The rate of elimination was enhanced using 10% MeOD as a cosolvent with CD_3_CN in comparison to neat CD_3_CN;^27^ however, for consistency within these studies, alkylation and elimination experiments were conducted in CD_3_CN.

In addition, under the pseudo-first-order conditions used, depending on the reporter group structure, it was observed that post degradation of the self-immolative system, alternative alkylation pathways could occur involving the excess benzyl bromide present. Therefore, three distinct reporter group categories have been determined: (i) partial alkylation of the carbamate leaving group was observed for the reporter groups featuring two *ortho*-methyl groups leading to the formation of Cbz-protected amines (observed for **17**, **20**, **21**, and **23**; see Figures S123, S126–S127, S129, and S130), (ii) full N-alkylation of the reporter group was observed for **22** after the release of *N*-methylaniline (see Figures S128 and S131), and (iii) in the case of reporter groups possessing either one or no *ortho*-methyl groups and a nitro substituent, alkylation was not observed (**15**, **16**, **18**, and **19**; see Figures S121–S122 and S124–S125). As a result of increasing the steric bulk of the reporter groups (case (i): **17**, **20**, **21**, and **23**), the lifetime of the carbamate anion generated upon initial degradation was sufficient to permit its alkylation. In the case of (ii), the increased nucleophilicity of *N*-methylaniline released from **22a** led to rapid N-alkylation with the excess benzyl bromide present. The optimum degradation pathway was obtained in the case of (iii), whereby the reporter units featured a nitro substituent and either a hydrogen or a single methyl group in the *ortho*-position. Since slow decarboxylation and subsequent alkylation lead to loss of the color of the reporter group, this is an important design point for this self-immolative system.

**Figure 5:**
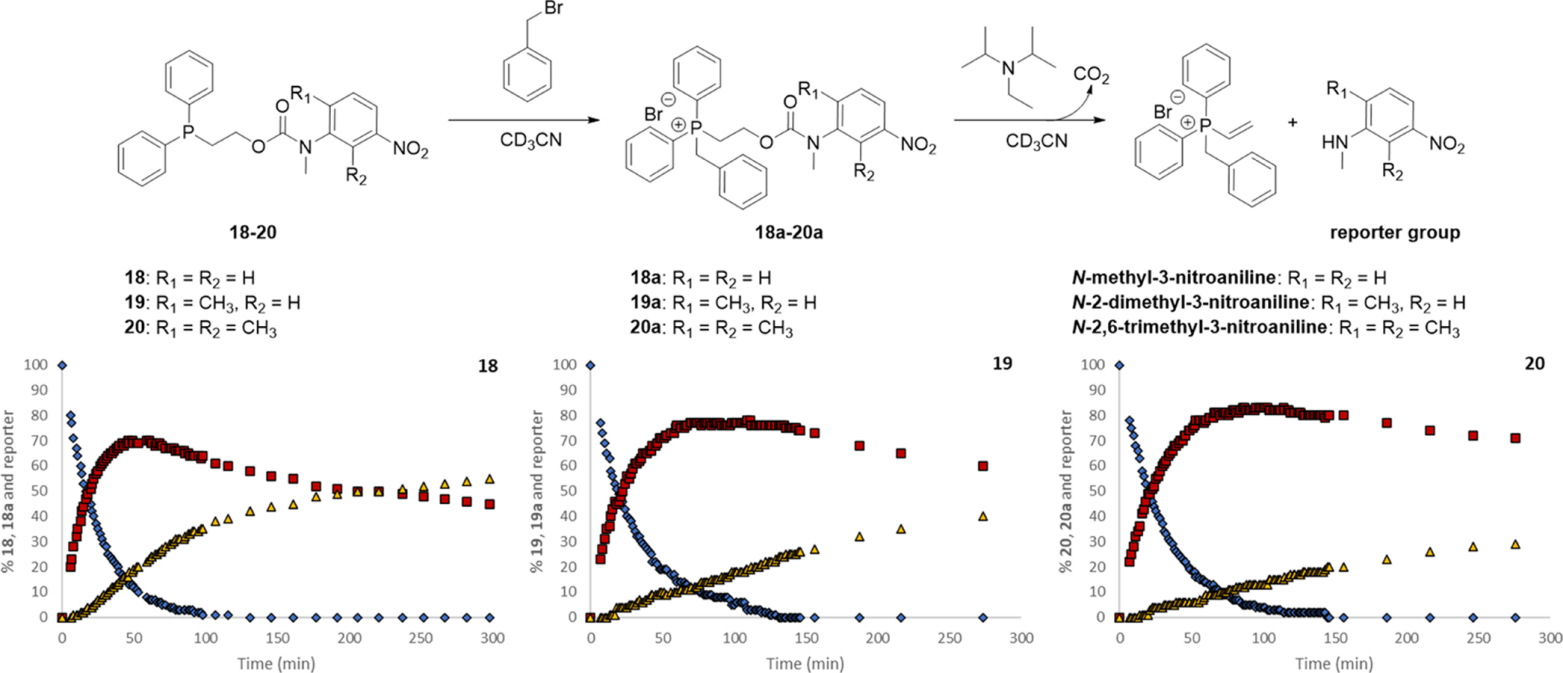
Top: reaction schematic of the self-immolative degradation of disclosure systems **18**–**20**. Bottom: reaction kinetics calculated from the ^1^H NMR spectra (20 °C) obtained following the addition of 10 molar equiv of benzyl bromide to **18**–**20** and *N*,*N*-diisopropylethylamine (DIPEA) in a one-pot approach (**18**–**20**, blue diamonds; **18a**–**20a**, red squares; reporter groups, yellow triangles). Errors associated with the integral values for these ^1^H NMR studies were ±5%.

In the light of the stability of **19** in both the neat and solution forms, taken together with its reactivity profile, this self-immolative system was selected for degradation studies, whereby the disclosure system and base were present and then exposed to the alkylating agent. As a result, a one-pot alkylation–elimination sequence was carried out by mixing system **19** with 2 molar equiv of DIPEA before the addition of benzyl bromide. From the plot shown in Figure 5, it can be observed that after addition of 10 molar equiv of benzyl bromide the reporter group *N-*methyl-2-methyl-5-nitroaniline was released, demonstrating the viability of this one-pot alkylation–elimination process (see Figure S137 and Table S10). Furthermore, an intense yellow coloration was evident within 15 min following the addition of the alkylating agent. In addition, identical degradation studies conducted on self-immolative systems **18** and **20** confirmed the viability of the one-pot alkylation–elimination process as well as the relative reactivities of these compounds relative to **19** (*e.g*., **18** > **19** > **20**; see Figures S138–S139 and Tables S9 and S11). In addition to these degradation studies, self-immolative system **19** was further studied via UV–visible spectroscopy, utilizing a solution of alkylated **19** and in relation to an external calibration plot. In an analogous one-pot study, the concentration of both **19** and benzyl bromide was decreased 10-fold and the release of the reporter group was monitored with respect to time (see Figures S140–S143).

## Conclusions

In summary, a range of selective self-immolative systems, triggered by a nonacidic electrophilic alkylating agent to afford a facile colorimetric visual disclosure, have been synthesized. The effect of the structure of the reporter group upon the rate of degradation and stability of the compounds has been determined. A significant influence on both the stability and the rate of elimination was determined in relation to the position of the nitro substituent (*meta*- vs *para*-) on the aniline reporter group. In addition, both the stability and reactivity of the self-immolative system could be tuned by the introduction of methyl groups in the *ortho*-position relative to the carbamate moiety of the reporter group. The *ortho-*methyl groups were found to have imparted an increased twist on the N–C (aromatic) bond, which led to a reduction of the ability of the carbamate nitrogen’s lone pair to resonate within the aromatic ring, resulting in a decrease of the leaving character of the carbamate group. Additionally, this should render the carbamate less electron withdrawing and so less able to acidify the β-hydrogen undergoing elimination. This study revealed that the structural modifications to the reporter group had a modest influence on the alkylation rate, with similar half-lives being observed for the benzylation of self-immolative systems **15**–**23**. In contrast, the β-elimination rates were affected by both the position of the nitro group on the aromatic ring (p*K*_a_) and the introduction of *ortho*-methyl groups relative to the carbamate group (conformation). The position and nature of the substituents on the reporter group have led to these being divided into three groups, depending on their fate post release. Additionally, the rate of the present 1,2-elimination appears to be more substantially affected by the nature of the reporter group than the original 1,6-elimination reported by Katzenellenbogen, and by Hay and 1,4- and 1,6- examples reported by Shabat.^4b,20,4d^ From the series of self-immolative disclosure systems synthesized, **19** was selected as the candidate for the disclosure of reactive alkylating agents, as it exhibited the optimum balance between stability and reactivity. Owing to the potential of these self-immolative systems to offer a practical route for the selective disclosure of reactive electrophilic species, such as chemical warfare agents, outside of the laboratory environment and without need for instrumentation, further studies are currently underway to produce clear and robust disclosure events for a broader range of electrophiles.

## Experimental Section

### General Information

All chemical reagents were purchased from Sigma-Aldrich and used as received, without purification. 2-Diphenylphosphinoethanol^41,42^ and *N*-(2,6-dimethyl-4-nitrophenyl)-4-methylbenzenesulfonamide^30^ were synthesized by modifications of literature procedures. Solvents were purchased from Fisher Scientific except for ethyl acetate and hexane, which were purchased from Sigma-Aldrich. All solvents were used as supplied except for THF that was distilled under argon from sodium and benzophenone prior to use. Fisher Scientific Silica 60A (particle size 35–70 μm) was used to perform column chromatography. Thin-layer chromatography (TLC) was performed on aluminum sheets coated with Merck 5735 Kieselgel 60 F_254_. Developed plates were air-dried and stained using a potassium permanganate solution. ^1^H (400 MHz), ^31^P{^1^H} (162 MHz), and ^13^C{^1^H} (100 MHz) NMR spectra were recorded at 20 °C on a Bruker Nanobay 400 MHz (9.39 T) or a Bruker DPX 400 (9.39 T) instrument. ^1^H (500 MHz), ^31^P{^1^H} (203 MHz), and ^13^C{^1^H} (125 MHz) variable-temperature NMR (VT-NMR) were recorded on a Bruker Avance III 500 MHz instrument (11.74 T). This instrument was calibrated at installation with ethylene glycol with an associated error of ±0.1 °C. ^1^H NMR spectra recorded in CDCl_3_ were referenced to tetramethylsilane (TMS) as the internal standard, whereas those recorded in CD_3_CN, DMF-*d*_7_, or DMSO-*d*_6_ were referenced to residual solvent. Chemical shifts (δ) are reported in parts per million (ppm) from low to high field. Coupling constants (*J*) are reported in hertz (Hz). Standard abbreviations indicating multiplicity are used as follows: br. = broad, s = singlet, d = doublet, t = triplet, q = quartet, m = multiplet, and app. = apparent. Fourier transform infrared (FTIR) spectra were recorded on a PerkinElmer Spectrum FTIR directly using a diamond ATR sampling accessory. Mass spectrometry was conducted using a ThermoFisher Scientific Orbitrap XL LCMS. The sample was introduced by liquid chromatography, and sample ionization was achieved by electrospray ionization (ESI). Elemental microanalyses of **2**, **3**, **6**, and **7** were performed by MEDAC Ltd. Melting points were recorded using a Stuart MP10 melting point. UV–visible spectra were measured with a Varian Cary 300 spectrophotometer using a 10 mm inner width Quartz cuvette in the wavelength range of 250–800 nm.

Crystals of **22**, **23**, **24**, **25**, **26, 27**, and **28** were mounted under paratone-N oil and flash-cooled to either 100 K (**22**, **23**, **26**, **27**, **28**) or 150 K (**24**, **25**) under nitrogen in an Oxford Cryosystems Cryostream. Single-crystal X-ray intensity data were collected using either an Agilent Gemini S Ultra diffractometer (Mo Kα radiation (λ = 0.71073 Å)) (**24**, **25**) or a Rigaku XtaLAB Synergy diffractometer (Cu Kα radiation (λ = 1.54184 Å)) (**22**, **23**, **26**, **27**, **28**). The data were reduced within CrysAlisPro software.^43^ The structures were solved using the program Superflip,^44^ and all nonhydrogen atoms were located. Least-squares refinements on *F* were carried out using the CRYSTALS suite of programs.^45^ The nonhydrogen atoms were refined anisotropically. All of the hydrogen atoms were located in difference Fourier maps and then placed geometrically with a C–H distance of 0.95 Å and a *U*_iso_ of 1.2 times the value of *U*_eq_ of the parent C atom. The hydrogen atoms attached to C were then refined with riding constraints. The crystallographic details for compounds **22**–**28** are presented in the Supporting Information.

#### Synthesis of *N*,2,6-Trimethyl-4-nitroaniline (**10**)

*N*-(2,6-Dimethyl-4-nitrophenyl)-4-methylbenzenesulfonamide^30^ (4.0 g, 12.49 mmol) was dissolved in THF (20 mL) and cooled to room temperature. Upon cooling, 60% sodium hydride dispersion in mineral oil (0.55 g, 13.73 mmol) was added followed by iodomethane (1.17 mL, 18.73 mmol) and left to stir overnight. The volatiles were removed *in vacuo* to yield a white crystalline solid and used without further purification. *N-*(2,6-Dimethyl-4-nitrophenyl)-*N*-4-dimethylbenzenesulfonamide was dissolved in H_2_SO_4_ (15 mL) and water (1 mL) and warmed at 40 °C (using an aluminum heating block) for 16 h. The reaction mixture was poured slowly into an ice/water/NaOH mixture. This was extracted with ethyl acetate, dried over MgSO_4_, filtered, and the solvent was removed *in vacuo* to yield yellow crystals (1.89 g, 84%). Mp 90–92 °C. ^1^H NMR (CDCl_3_, 400 MHz) δ 7.86 (s, 2H), 3.88 (br. s, 1H), 3.06 (s, 3H), 2.33 (s, 6H). ^13^C{^1^H} NMR (CDCl_3_, 100 MHz) δ 153.8, 139.4, 125.4, 124.9, 24.9, 19.6. FTIR (ATR/ν_max_) 3425, 2920, 2383, 1592, 1525, 1421, 1352, 1254, 1235, 1153, 1107, 1029 cm^–1^. HRMS (ESI) *m*/*z*: [M + H]^+^ calcd for C_9_H_13_O_2_N_2_ 181.0972; found 181.0972.

### General Procedure 1: Synthesis of *N*-Methylated Anilines (**11–14**)

To a solution of HFIP (hexafluoroisopropanol) (10 equiv) and aniline (1 equiv) was added methyl trifluoromethanesulfonate (MeOTf) (1.5 equiv). The mixture was stirred for 1 h at room temperature and then quenched by a solution of 2 N HCl. Volatiles were evaporated *in vacuo*. The resulting mixture was neutralized with a saturated aqueous solution of NaHCO_3_ and extracted with CH_2_Cl_2_ (3 × 30 mL). The organic phases were dried over MgSO_4_, filtered, and the solvent was removed *in vacuo*. The crude product was further purified by column chromatography.

#### Synthesis of *N*,2-Dimethyl-4-nitroaniline (**11**)

Following general procedure 1, 2-methyl 4-nitroaniline (2.0 g, 13.14 mmol) was treated with HFIP (13.8 mL, 131.45 mmol) and MeOTf (2.16 mL, 19.72 mmol) to provide **11** (1.58 g, 72%) as a yellow solid after silica-gel column chromatography (THF/hexane 10/90). Mp 138–140 °C. ^1^H NMR (CDCl_3_, 400 MHz) δ 8.09 (dd, ^3^*J* = 9.0 Hz, ^4^*J* = 2.5 Hz, 1H), 7.99–7.96 (d, ^4^*J* = 2.5 Hz, 1H), 6.53 (d, ^3^*J* = 9.0 Hz, 1H), 4.37 (br. s, 1H), 3.00 (d, ^3^*J* = 5.0 Hz, 3H), 2.17 (s, 3H). ^13^C{^1^H} NMR (CDCl_3_, 100 MHz) δ 152.6, 137.6, 126.0, 124.9, 121.0, 107.4, 30.5, 17.3. FTIR (ATR/ν_max_) 3378, 2908, 1608, 1587, 1541, 1486, 1463, 1407, 1386, 1290, 1261, 1189, 1118, 1094, 1036, 1001 cm^–1^. HRMS (ESI) *m*/*z*: [M + H]^+^ calcd for C_8_H_11_O_2_N_2_ 167.0815; found 167.0810.

#### Synthesis of *N*,2-Dimethyl-5-nitroaniline (**12**)

Following general procedure 1, 2-methyl-5-nitroaniline (2.0 g, 13.14 mmol) was treated with HFIP (13.8 mL, 131.45 mmol), and MeOTf (2.16 mL, 19.72 mmol) to provide **12** (1.49 g, 68%) as a yellow solid after silica-gel column chromatography (THF/hexane 10/90). Mp 110–112 °C. ^1^H NMR (CDCl_3_, 400 MHz) δ 8.31 (dd, ^3^*J* = 8.0 Hz, ^4^*J* = 2.5 Hz, 1H), 7.37 (d, ^4^*J* = 2.5 Hz, 1H), 7.13 (d, ^3^*J* = 8.0 Hz, 1H), 3.85 (br. s, 1H), 2.96 (d, ^3^*J* = 4.0 Hz, 3H), 2.19 (s, 3H). ^13^C{^1^H} NMR (CDCl_3_, 100 MHz) δ 148.0, 147.9, 130.0, 129.3, 111.9, 103.1, 30.7, 17.7. FTIR (ATR/ν_max_) 3429, 2923, 2801, 1621, 1524, 1498, 1336, 1284, 1162, 1093, 1067 cm^–1^. HRMS (ESI) *m*/*z*: [M + H]^+^ calcd for C_8_H_11_O_2_N_2_ 167.0815; found 167.0816.

#### Synthesis of *N*,2,6-Trimethyl-3-nitroaniline (**13**)

Following general procedure 1, 2,6-dimethyl-3-nitroaniline (2.0 g, 12.04 mmol) was treated with HFIP (12.64 mL, 120.35 mmol) and MeOTf (1.97 mL, 18.05 mmol) to provide **13** (1.67 g, 77%) as a yellow oil after silica-gel column chromatography (THF/hexane 10/90). ^1^H NMR (CDCl_3_, 400 MHz) δ 7.39 (d, ^3^*J* = 8.5 Hz, 1H), 7.07 (d, ^3^*J* = 8.5 Hz, 1H), 3.23 (br. s, 1H), 2.80 (s, 3H), 2.42 (s, 3H), 2.33 (s, 3H). ^13^C{^1^H} NMR (CDCl_3_, 100 MHz) δ 150.2, 149.5, 134.4, 128.3, 124.3, 117.6, 35.5, 18.9, 14.9. FTIR (ATR/ν_max_) 3407, 2953, 1599, 1465, 1376, 1293, 1260, 1229, 1144, 1158, 1013 cm^–1^. HRMS (ESI) *m*/*z*: [M + H]^+^ calcd for C_9_H_13_O_2_N_2_ 181.0972; found 181.0970.

#### Synthesis of *N*,2,4,6-Tetramethyl-3-nitroaniline (**14**)

Following general procedure 1, 2,4,6-trimethyl-3-nitroaniline (2.0 g, 11.10 mmol) was treated with HFIP (11.66 mL, 110.98 mmol) and MeOTf (1.82 mL, 16.65 mmol) to provide **14** (1.79 g, 83%) as a yellow solid after silica-gel column chromatography (THF/hexane 10/90). Mp 59–60 °C. ^1^H NMR (CDCl_3_, 400 MHz) δ 6.89 (app. s, 1H), 2.98 (br. s, 1H), 2.75 (s, 3H), 2.27 (s, 3H), 2.20 (s, 6H). ^13^C{^1^H} NMR (CDCl_3_, 100 MHz) δ 151.6, 146.5, 131.8, 130.7, 122.3, 121.2, 35.6, 18.4, 16.9, 13.3. FTIR (ATR/ν_max_) 3407, 2930, 1582, 1507, 1476, 1432, 1377, 1356, 1309, 1281, 1249, 1216, 1151, 1063, 1038, 1013 cm^–1^. HRMS (ESI) *m*/*z*: [M + H]^+^ calcd for C_10_H_15_O_2_N_2_ 195.1128; found 195.1123.

### General Procedure 2: Synthesis of Carbamoyl Chlorides (**1–9**)

To a solution of triphosgene (2 equiv) in dry hexane (10 mL), aliquat 336 (0.5 equiv) was added and left to stir at room temperature for 16 h. A solution of the *N*-methylated aniline derivative (1 equiv) and triethylamine (2 equiv) in THF (20 mL) was added dropwise to the solution at 0 °C. After stirring the solution at room temperature for 2 h, the precipitate was filtered and the solvent was removed *in vacuo*. The crude product was further purified by column chromatography.

#### Synthesis of Methyl(4-nitrophenyl)carbamic Chloride (**1**)^31^

Following the general procedure 2, *N*-methyl-4-nitroaniline (0.77 g, 5.05 mmol) was treated with triphosgene (3.00 g, 10.11 mmol), aliquat 336 (1.00 g, 2.53 mmol), and triethylamine (1.41 mL, 10.11 mmol) to provide **1** (0.91 g, 84% yield) as a white solid after silica-gel column chromatography (10 → 20% EtOAc/hexane) followed by a recrystallization from EtOAc/hexane (9/1 v/v). Mp 101–103 °C. ^1^H NMR (CDCl_3_, 400 MHz) δ 8.31 (app. d, 2H), 7.48 (app. d, 2H), 3.48 (s, 3H). ^13^C{^1^H} NMR (CDCl_3_, 100 MHz) δ 143.3, 142.9, 141.6 (br.), 122.5, 119.7, 35.3 (br.). FTIR (ATR/ν_max_) 3111–3081, 1726, 1594, 1512, 1495, 1418, 1342, 1255 cm^–1^.

#### Synthesis of Methyl(2-methyl-4-nitrophenyl)carbamic Chloride (**2**)

Following the general procedure 2, **11** (0.84 g, 5.05 mmol) was treated with triphosgene (3.00 g, 10.11 mmol), aliquat 336 (1.00 g, 2.53 mmol), and triethylamine (1.86 mL, 13.30 mmol) to provide **2** (0.89 g, 76% yield) as a white solid with an 83:17 mixture of rotamers A/B after silica-gel column chromatography (10 → 20% EtOAc/hexane). Mp 85–87 °C. ^1^H NMR (CDCl_3_, 400 MHz) δ 8.23–8.17 (s, 1H, rotamer A + rotamer B), 8.17–8.10 (m, 1H, rotamer A + rotamer B), 7.37 (app. d, 1H, rotamer A + rotamer B), 3.46 (s, 3H, rotamer B), 3.32 (s, 3H, rotamer A), 2.42 (s, 3H rotamer A), 2.38 (s, 3H rotamer B). ^13^C{^1^H} NMR (CDCl_3_, 100 MHz) δ 149.1, 148.5, 147.7, 147.4, 147.1, 147.0, 138.1, 137.3, 129.4, 127.9, 126.6, 122.9, 122.7, 41.3, 39.0, 17.9, 17.8. FTIR (ATR/ν_max_) 3081–2932, 1724, 1586, 1529, 1513, 1491, 1344, 1304, 1252, 1113, 1085 cm^–1^. Anal. calcd for C_9_H_9_ClN_2_O_3_: C, 47.28; H, 3.97; N, 12.25. Found: C, 46.97; H, 3.75; N, 11.97.

#### Synthesis of (2,6-Dimethyl-4-nitrophenyl)(methyl)carbamic Chloride (**3**)

Following the general procedure 2, **10** (0.91 g, 5.05 mmol) was treated with triphosgene (3.00 g, 10.11 mmol), aliquat 336 (1.00 g, 2.53 mmol), and triethylamine (1.86 mL, 13.30 mmol) to provide **3** (0.97 g, 72% yield) as a white solid with an 88:12 mixture of rotamers A/B after silica-gel column chromatography (10 → 20% EtOAc/hexane). Mp 124–126 °C. ^1^H NMR (CDCl_3_, 400 MHz) δ 8.02 (s, 1H, rotamer A), 8.00 (s, 1H, rotamer B), 3.37 (s, 3H, rotamer B), 3.26 (s, 3H, rotamer A), 2.39 (s, 6H, rotamer A), 2.36 (s, 6H, rotamer B). ^13^C{^1^H} NMR (CDCl_3_, 100 MHz) δ 148.8, 147.4, 146.0, 138.1, 137.2, 124.0, 39.7, 37.3, 18.1 (×2). FTIR (ATR/ν_max_) 3086–2966, 1744, 1587, 1516, 1473, 1348, 1299, 1238, 1118, 1090 cm^–1^. Anal. calcd for C_10_H_11_ClN_2_O_3_: C, 49.50; H, 4.57; N, 11.54. Found: C, 49.13; H, 4.60; N, 11.18.

#### Synthesis of Methyl(3-nitrophenyl)carbamic Chloride (**4**)^33^

Following the general procedure 2, *N*-methyl-3-nitroaniline (0.77 g, 5.05 mmol) was treated with triphosgene (3.00 g, 10.11 mmol), aliquat 336 (1.00 g, 2.53 mmol), and triethylamine (1.41 mL, 10.11 mmol) to provide **4** (0.75 g, 70% yield) as a white solid after silica-gel column chromatography (10 → 20% EtOAc/hexane). Mp 92–94 °C. ^1^H NMR (CDCl_3_, 400 MHz) δ 8.26 (m, 1H), 8.18 (s, 1H), 7.76–7.58 (m, 2H) 3.47 (br. s, 3H). ^13^C{^1^H} NMR (CDCl_3_, 100 MHz) δ 148.8, 143.9, 133.8 (br.), 130.6, 123.3 (br.), 40.4. FTIR (ATR/ν_max_) 3073, 1727, 1532, 1480, 1418, 1352, 1252 cm^–1^.

#### Synthesis of Methyl(2-methyl-5-nitrophenyl)carbamic Chloride (**5**)^34^

Following the general procedure 2, **12** (0.84 g, 5.05 mmol) was treated with triphosgene (3.00 g, 10.11 mmol), aliquat 336 (1.00 g, 2.53 mmol), and triethylamine (1.86 mL, 13.30 mmol) to provide **5** (1.01 g, 87% yield) as a colorless oil with an 83:17 mixture of rotamers A/B after silica-gel column chromatography (10 → 20% EtOAc/hexane). ^1^H NMR (CDCl_3_, 400 MHz) δ 8.16 (dd, ^3^*J* = 8.5 Hz, ^4^*J* = 2.5 Hz, 1H rotamer A), 8.11 (dd, ^3^*J* = 8.5 Hz, ^4^*J* = 2.5 Hz, 1H, rotamer B), 8.09–8.05 (m, 1H, rotamer A + rotamer B), 7.49 (d, ^3^*J* = 8.5 Hz, 1H, rotamer A), 7.46 (d, ^3^*J* = 8.5 Hz, 1H, rotamer B), 3.48 (s, 3H, rotamer B), 3.33 (s, 3H, rotamer A), 2.40 (s, 3H, rotamer A), 2.36 (s, 3H, rotamer B).^13^C{^1^H} NMR (CDCl_3_, 100 MHz) δ 149.4 (rotamer B), 148.8 (rotamer A), 147.1 (rotamer A), 147.0 (rotamer B), 144.0 (rotamer A), 143.3 (rotamer B), 142.5 (rotamer A), 142.1 (rotamer B), 132.3 (rotamer A), 132.2 (rotamer B), 123.9 (rotamer A), 123.5 (rotamer A), 123.4 (rotamer B), 122.3 (rotamer B), 41.4 (rotamer B), 39.1 (rotamer A), 17.9 (rotamer B), 17.8 (rotamer A). FTIR (ATR/ν_max_) 2923–2852, 1733, 1520, 1480, 1347, 1325, 1270, 1253 cm^–1^.

#### Synthesis of (2,6-Dimethyl-3-nitrophenyl)(methyl)carbamic Chloride (**6**)

Following the general procedure 2, **13** (1.20 g, 6.67 mmol) was treated with triphosgene (3.95 g, 13.30 mmol), aliquat 336 (1.32 g, 3.33 mmol), and triethylamine (1.86 mL, 13.30 mmol) to provide **6** (1.20 g, 74% yield) as a white solid with an 87:13 mixture of rotamers A/B after silica-gel column chromatography (10% EtOAc/hexane). Mp 64–66 °C. ^1^H NMR (CDCl_3_, 400 MHz) δ 7.88 (d, ^3^*J* = 8.5 Hz, 1H, rotamer A), 7.84 (d, ^3^*J* = 8.5 Hz, 1H rotamer B), 7.30 (d, ^3^*J* = 8.5 Hz, 1H, rotamer A + rotamer B), 3.38 (s, 3H, rotamer B), 3.26 (s, 3H, rotamer A), 2.45 (s, 3H, rotamer A), 2.44 (s, 3H, rotamer B), 2.36 (s, 3H, rotamer A), 2.34 (s, 3H, rotamer B). ^13^C{^1^H} NMR (CDCl_3_, 100 MHz) δ 151.5, 146.4, 131.7, 130.5, 122.2, 121.1, 40.0, 37.5, 18.3, 14.2. Anal. calcd for C_10_H_11_ClN_2_O_3_: C, 49.50; H, 4.57; N, 11.54. found: C, 49.38; H, 4.40; N, 11.38.

#### Synthesis of Methyl(2,4,6-trimethyl-3-nitrophenyl)carbamic Chloride (**7**)

Following the general procedure 2, **14** (0.55 g, 2.84 mmol) was treated with triphosgene (1.68 g, 5.67 mmol), aliquat 336 (0.56 g, 1.42 mmol), and triethylamine (0.79 mL, 5.67 mmol) to provide **7** (0.97 g, 72% yield) as a colorless oil with an 88:12 mixture of rotamers A/B after silica-gel column chromatography (10% EtOAc/hexane). ^1^H NMR (CDCl_3_, 400 MHz) δ 7.09 (s, 1H, rotamer A), 7.07 (s, 1H, rotamer B), 3.35 (s, 3H, rotamer B), 3.23 (s, 3H, rotamer A), 2.30 (s, 3H, rotamer A), 2.28 (s, 3H, rotamer A), 2.27 (s, 3H, rotamer B), 2.26 (s, 3H, rotamer B), 2.19 (s, 3H, rotamer A), 2.17 (s, 3H, rotamer B). ^13^C{^1^H} NMR (CDCl_3_, 100 MHz) δ 150.9, 149.4, 139.3, 138.4, 137.5, 131.3, 130.2, 129.9, 128.0, 40.1, 37.6, 17.7, 17.4, 12.9. FTIR (ATR/ν_max_) 2931, 1734, 1524, 1477, 1415, 1366, 1350, 1330, 1304, 1219, 1119, 1058, 1015 cm^–1^. Anal. calcd for C_11_H_13_ClN_2_O_3_: C, 51.47; H, 5.10; N, 10.91. Found: C, 51.48; H, 5.11; N, 10.78.

#### Synthesis of Methyl(phenyl)carbamic Chloride (**8**)^31^

Following the general procedure 2, *N*-methylaniline (0.77 g, 7.19 mmol) was treated with triphosgene (4.27 g, 14.40 mmol), aliquat 336 (1.00 g, 2.53 mmol), and triethylamine (2 mL, 14.4 mmol) to provide **8** (0.91 g, 84% yield) as a white solid after silica-gel column chromatography (10% EtOAc/hexane) followed by a recrystallization from hexane. Mp 88–90 °C. ^1^H NMR (CDCl_3_, 400 MHz) δ 7.50–7.32 (m, 3H), 7.32–7.18 (m, 2H), 3.35 (br. s, 3H). ^13^C{^1^H} NMR (CDCl_3_, 100 MHz) δ 149.2, 143.3, 129.6, 128.5, 127.4, 40.4. FTIR (ATR/ν_max_) 2942, 1727, 1598, 2587, 1497, 1454, 1418, 1360, 1329, 1263, 1160, 1113, 1071, 1034, 1018 cm^–1^.

#### Synthesis of Mesityl(methyl)carbamic Chloride (**9**)^33^

Following the general procedure 2, *N*-methyl-2,4,6-trimethylaniline (1.0 g, 6.70 mmol) was treated with triphosgene (3.98 g, 13.40 mmol), aliquat 336 (1.65 g, 3.34 mmol), and triethylamine (1.9 mL, 13.62 mmol) to provide **9** (1.10 g, 50% yield) as a white solid with a 90:10 mixture of rotamers A/B after silica-gel column chromatography (10% EtOAc/hexane) followed by a recrystallization from hexane. Mp 73–75 °C. ^1^H NMR (CDCl_3_, 400 MHz) δ 6.94 (m, 2H rotamer A), 6.92 (m, 2H, rotamer B), 3.34 (s, 3H, rotamer B), 3.23 (s, 3H, rotamer A), 2.30 (s, 3H, rotamer A), 2.27 (s, 3H rotamer B), 2.22 (s, 6H, rotamer A + rotamer B). ^13^C{^1^H} NMR (CDCl_3_, 100 MHz) δ 150.0, 138.6, 138.4, 135.2, 129.5, 37.6, 21.0, 17.5. FTIR (ATR/ν_max_) 2920, 1727, 1483, 1409, 1379, 1354, 1333, 1302, 1248, 1169, 1100, 1035, 1011 cm^–1^.

### General Procedure 3: Synthesis of Self-Immolative Systems (**15–23**)

Carbamoyl chloride (1 equiv), 2-(diphenylphosphino)ethanol (1 equiv), 4-dimethylaminopyridine (0.1 equiv), and triethylamine (2 equiv) were dissolved in THF (20 mL), and the solution was heated (using an aluminum heating block) under reflux overnight. The solvent was removed *in vacuo*, and the crude product was purified via column chromatography.

#### Synthesis of 2-(Diphenylphosphanyl)ethyl Methyl(4-nitrophenyl)carbamate (**15**)^23^

Following the general procedure 3, **1** (0.30 g, 1.40 mmol) was treated with 2-(diphenylphosphino)ethanol (0.32 g, 1.40 mmol), 4-dimethylaminopyridine (0.018 g, 0.14 mmol), and triethylamine (0.38 mL, 2.80 mmol) to provide **15** (0.40 g, 73%) as a pale yellow oil after silica-gel column chromatography (hexane/EtOAc 75/25). ^1^H NMR (CDCl_3_, 400 MHz) δ 8.18 (2H, AA′XX′), 7.48–7.38 (m, 6H), 7.38–7.28 (m, 6H), 4.35 (dt, *J* = 9.5, 7.0 Hz, 2H), 3.25 (s, 3H), 2.48 (t, *J* = 7.0 Hz, 2H). ^13^C{^1^H} NMR (CDCl_3_, 100 MHz) δ 154.5, 148.8, 144.3, 137.6 (d, ^1^*J*_CP_ = 12.0 Hz), 132.6 (d, ^2^*J*_CP_ = 19.0 Hz), 128.9, 128.6 (d, ^3^*J*_CP_ = 7.0 Hz), 124.4, 124.2, 64.2 (d, ^2^*J*_CP_ = 23.3 Hz), 36.8, 28.1 (d, ^1^*J*_CP_ = 14.4 Hz). ^31^P{^1^H} NMR (CDCl_3_, 162 MHz) −22.27. FTIR (ATR/ν_max_) 3051–2954, 1705, 1593, 1513, 1498, 1433, 1325, 1254, 1151, 1103 cm^–1^. HRMS (ESI) *m*/*z*: [M + H]^+^ calcd for C_22_H_22_N_2_O_4_P 409.1312; found 409.1304.

#### Synthesis of 2-(Diphenylphosphanyl)ethyl Methyl(2-methyl-4-nitrophenyl)carbamate (**16**)

Following the general procedure 3, **2** (0.30 g, 1.31 mmol) was treated with 2-(diphenylphosphino)ethanol (0.30 g, 1.31 mmol), 4-dimethylaminopyridine (0.016 g, 0.13 mmol), and triethylamine (0.37 mL, 2.62 mmol) to provide **16** (0.38 g, 69%) as a colorless oil after silica-gel column chromatography (hexane/EtOAc 75/25). ^1^H NMR (CDCl_3_, 400 MHz) δ 8.18–7.95 (m, 2H rotamer A + rotamer B), 7.55–7.15 (m, 11H, rotamer A + rotamer B), 4.44–4.29 (m, 2H, rotamer B), 4.29–4.11 (m, 2H, rotamer A), 3.20 (s, 3H, rotamer A), 3.06 (s, 3H, rotamer B), 2.59–2.18 (m, 5H, rotamer A + rotamer B). ^13^C{^1^H} NMR (CDCl_3_, 100 MHz) δ 154.9, 147.5, 146.7, 137.7, 132.6 (d, ^1^*J*_cp_ =18.5 Hz), 129.1–128.1 (m, 7C), 126.2, 122.2, 63.9 (d, ^2^*J* = 25.0 Hz), 37.2, 28.2 (d, ^1^*J*_cp_ = 15.0 Hz), 18.0 (br.). ^31^P{^1^H} NMR (CDCl_3_, 162 MHz) δ −22.08 (rotamer B), −22.80 (rotamer A). FTIR (ATR/ν_max_) 3070–2925, 1704, 1586, 1520, 1492, 1433, 1345, 1154, 1086 cm^–1^. HRMS (ESI) *m*/*z*: [M + H]^+^ calcd for C_23_H_24_O_4_N_2_P 423.14468; found 423.1459.

#### Synthesis of 2-(Diphenylphosphanyl)ethyl (2,6-Dimethyl-4-nitrophenyl)(methyl)carbamate (**17**)

Following the general procedure 3, **3** (0.30 g, 1.24 mmol) was treated with 2-(diphenylphosphino)ethanol (0.29 g, 1.24 mmol), 4-dimethylaminopyridine (0.015 g, 0.12 mmol), and triethylamine (0.35 mL, 2.48 mmol) to provide **17** (0.37 g, 68%) as a colorless oil after silica-gel column chromatography (hexane/CH_2_Cl_2_ 1/1 → hexane/EtOAc 75/25). ^1^H NMR (CDCl_3_, 400 MHz) δ 7.95 (s, 2H, rotamer B), 7.91 (s, 2H rotamer A), 7.52–7.27 (m, 10H, rotamer A + rotamer B), 4.36 (dt, *J* = 9.5, 7.5 Hz, 2H, rotamer B), 4.18 (m, 2H, rotamer A), 3.13 (s, 3H, rotamer A), 2.99 (s, 3H, rotamer B), 2.53 (t, ^2^*J* = 7.5 Hz, 2H, rotamer B), 2.33–2.24 (m, 8H rotamer A + rotamer B). ^13^C{^1^H} NMR (CDCl_3_, 100 MHz) δ 155.1 (rotamer A), 154.5 (rotamer B), 146.7 (rotamer B), 146.6 (rotamer A), 146.5 (rotamer B), 146.0 (rotamer A), 138.2 (rotamer B), 137.9 (rotamer A), 137.8 (d, ^1^*J*_cp_ = 12.0 Hz, rotamer B), 137.7 (d, ^1^*J*_cp_ = 12.5 Hz, rotamer A), 132.8 (d, ^2^*J*_cp_ = 19.0 Hz, rotamer B), 132.6 (d, ^2^*J*_cp_ = 19.0 Hz, rotamer A), 129.0 (rotamer B), 128.9 (rotamer A), 128.7 (d, ^3^*J*_cp_ = 7.0 Hz, rotamer B), 128.6 (d, ^3^*J*_cp_ = 7.0 Hz, rotamer A), 123.7 (rotamer B), 123.6 (rotamer A), 63.9 (d, ^2^*J*_cp_ = 25.5 Hz, rotamer A), 63.8 (d, ^2^*J*_cp_ = 24.0 Hz, rotamer B), 35.6 (rotamer B), 35.4 (rotamer A), 28.5 (d, ^1^*J*_cp_ = 14.5 Hz, rotamer B), 28.3 (d, ^1^*J*_cp_ = 15.0 Hz, rotamer A), 18.2 (rotamer A and rotamer B). ^31^P{^1^H} NMR (CDCl_3_, 162 MHz) δ −22.17 (rotamer B), −22.99 (rotamer A). FTIR (ATR/ν_max_) 2923–2852, 2361, 1702, 1591, 1520, 1478, 1433, 1389, 1345, 1298, 1153, 1090 cm^–1^. HRMS (ESI) *m*/*z*: [M + H]^+^ calcd for C_24_H_26_O_4_N_2_P 437.162; found 437.1621.

#### Synthesis of 2-(Diphenylphosphanyl)ethyl Methyl(3-nitrophenyl)carbamate (**18**)

Following the general procedure 3, **4** (0.30 g, 1.40 mmol) was treated with 2-(diphenylphosphino)ethanol (0.32 g, 1.40 mmol), 4-dimethylaminopyridine (0.018 g, 0.14 mmol), and triethylamine (0.38 mL, 2.80 mmol) to provide **18** (0.40 g, 73%) as a colorless oil after silica-gel column chromatography (hexane/CH_2_Cl_2_ 1/1 → hexane/EtOAc 75/25). ^1^H NMR (CDCl_3_, 400 MHz) δ 8.10 (s, 1H), 8.03 (d, *J* = 8.0 Hz, 1H), 7.61 (d, *J* = 8.0 Hz, 1H), 7.48 (t, *J* = 8.0 Hz, 1H), 7.42 (m, 4H), 7.33 (m, 6H), 4.34 (q, ^3^*J* = 7.0 Hz, 2H), 3.24 (s, 3H), 2.46 (t, ^2^*J* = 7.0 Hz, 2H). ^13^C{^1^H} NMR (CDCl_3_, 100 MHz) δ 154.9, 148.5, 144.3, 137.7 (d, ^1^*J*_CP_ = 12.1 Hz), 132.8 (d, ^2^*J*_CP_ = 19.0 Hz), 131.3, 129.5, 129.0, 128.7 (d, ^3^*J*_CP_ = 6.9 Hz), 120.4, 120.0, 64.2 (d, ^2^*J*_CP_ = 23.4 Hz), 37.2, 28.2 (d, ^1^*J*_CP_ = 14.3 Hz). ^31^P{^1^H} NMR (CDCl_3_, 162 MHz) δ −22.3. FTIR (ATR/ν_max_) 3071–2954, 1702, 1526, 1481, 1433, 1386, 1342, 1261, 1154, 1126 cm^–1^. HRMS (ESI) *m*/*z*: [M + H]^+^ calcd for C_22_H_22_N_2_O_4_P 409.1312; found 409.1312.

#### Synthesis of 2-(Diphenylphosphanyl)ethyl Methyl(2-methyl-5-nitrophenyl)carbamate (**19**)

Following the general procedure 3, **5** (0.32 g, 1.40 mmol) was treated with 2-(diphenylphosphino)ethanol (0.32 g, 1.40 mmol), 4-dimethylaminopyridine (0.018 g, 0.14 mmol), and triethylamine (0.38 mL, 2.80 mmol) to provide **19** (0.18 g, 30%) as a colorless oil with a 65:35 mixture of rotamers A/B after silica-gel column chromatography (hexane/acetone 95/5). ^1^H NMR (CDCl_3_, 400 MHz) δ 8.04 (app. d, ^3^*J* = 8.5 Hz, 1H, rotamer A + rotamer B), 7.99 (s, 1H, rotamer B), 7.94 (s, 1H, rotamer A), 7.59–7.27 (m, 10H, rotamer A + rotamer B), 4.38 (br. s, 2H, rotamer B), 4.28–4.10 (m, 2H, rotamer A), 3.21 (s, 3H, rotamer A), 3.06 (s, 3H, rotamer B), 2.54 (br. s, 2H, rotamer B), 2.30 (br. s, 2H, rotamer A), 2.30 (s, 3H, rotamer A + rotamer B). ^13^C{^1^H} NMR (CDCl_3_, 100 MHz) δ 155.2 (rotamer A), 155.0 (rotamer B), 146.9 (rotamer A + rotamer B), 144.3 (rotamer B), 143.1 (rotamer A), 143.0 (rotamer B), 142.5 (rotamer A), 138.0–137.5 (m, rotamer A + rotamer B), 133.1–132.4 (m, rotamer A + rotamer B), 131.8 (rotamer B), 131.7 (rotamer A), 128.8 (d, ^2^*J*_cp_ = 22.5 Hz, rotamer A + rotamer B), 128.6 (rotamer A + rotamer B), 123.1 (rotamer A + rotamer B), 122.5 (br. s, rotamer A + rotamer B), 64.0 (d, ^2^*J*_cp_ = 25.5 Hz, rotamer A + rotamer B), 37.4 (rotamer A), 37.3 (rotamer B), 28.4 (d, ^1^*J*_cp_ = 15.0 Hz, rotamer B), 28.3 (d, ^1^*J*_cp_ = 14.5 Hz, rotamer A), 18.1 (rotamer A + rotamer B). ^31^P{^1^H} NMR (CDCl_3_, 162 MHz) δ −21.8 (rotamer B), −23.1 (rotamer A). FTIR(ATR/ν_max_) 3071–2954, 1702, 1519, 1433, 1340, 1153, 1123 cm^–1^. HRMS (ESI) *m*/*z*: [M + H]^+^ calcd for C_23_H_24_N_2_O_4_P 423.1468; found 423.1463.

#### Synthesis of 2-(Diphenylphosphanyl)ethyl (2,6-Dimethyl-3-nitrophenyl)(methyl)carbamate (**20**)

Following the general procedure 3, **6** (0.34 g, 1.40 mmol) was treated with 2-(diphenylphosphino)ethanol (0.32 g, 1.40 mmol), 4-dimethylaminopyridine (0.018 g, 0.14 mmol), and triethylamine (0.38 mL, 2.80 mmol) to provide **20** (0.10 g, 33%) as a colorless oil with a 72:28 mixture of rotamers A/B after silica-gel column chromatography (hexane/acetone 95/5). ^1^H NMR (CDCl_3_, 400 MHz) δ 7.77 (app. d, *J* = 8.5 Hz, 1H, rotamer A + rotamer B), 7.54–7.27 (m, 10H, rotamer A + rotamer B), 7.21 (app. d, *J* = 8.5 Hz, 1H rotamer B), 7.20 (app. d, ^3^*J* = 8.5 Hz, 1H, rotamer A), 4.38 (dt, ^3^*J*_HP_ = 8.5 Hz, ^3^*J* = 7.5 Hz, 2H, rotamer B), 4.23–4.13 (m, 2H, rotamer A), 3.14 (s, 3H rotamer A), 3.00 (s, 3H, rotamer B), 2.55 (t, *J* = 7.5 Hz, 2H, rotamer B), 2.34–2.29 (m, 2H, rotamer A), 2.28 (s, 3H, rotamer A), 2.27 (s, 3H, rotamer B). ^13^C{^1^H} NMR (CDCl_3_, 100 MHz) δ 155.3 (rotamer A), 154.7 (rotamer B), 149.1 (rotamer B), 149.0 (rotamer A), 142.6 (rotamer B), 142.4 (rotamer B), 142.3 (rotamer A), 141.9 (rotamer A), 141.3 (rotamer A), 137.85 (d, ^1^*J*_cp_ = 12.5 Hz, rotamer B, Ph′ or Ph″), 137.84 (d, ^1^*J*_cp_ = 12.5 Hz, rotamer B, Ph′ or Ph″), 137.66 (d, ^1^*J*_cp_ = 12.5 Hz, rotamer A, Ph″), 137.64 (d, ^1^*J*_cp_ = 12.5 Hz, rotamer A, Ph′), 132.74 (d, ^2^*J*_cp_ = 18.0 Hz, rotamer B, Ph′ or Ph″), 132.72 (d, ^2^*J*_cp_ = 18.5 Hz, rotamer B, Ph′ or Ph″), 132.60 (d, ^2^*J*_cp_ = 18.5 Hz, rotamer A, Ph″), 132.56 (d, ^2^*J*_cp_ = 18.5 Hz, rotamer A, Ph′), 132.0 (rotamer B), 131.7 (rotamer A), 128.9 (rotamer B), 128.8 (rotamer A), 128.74 (d, ^3^*J*_cp_ = 6.0 Hz, rotamer B), 128.70 (d, ^3^*J*_cp_ = 7.5 Hz, rotamer A), 128.5 (rotamer A + rotamer B), 123.8 (rotamer B), 123.7 (rotamer A), 63.7 (d, ^3^*J*_cp_ = 26.0 Hz, rotamer A), 63.6 (d, ^3^*J*_cp_ = 23.0 Hz, rotamer B), 35.8 (rotamer B), 35.7 (rotamer A), 28.4 (d, ^3^*J*_cp_ = 14.5 Hz, rotamer B), 28.3 (d, ^3^*J*_cp_ = 15.0 Hz, rotamer A), 18.43 (rotamer A), 18.40 (rotamer B), 14.3 (rotamer A + rotamer B). ^31^P{^1^H} NMR (CDCl_3_, 162 MHz) δ −22.15 (rotamer B), −23.37 (rotamer A). FTIR (ATR/ν_max_) 3081–2932, 1724, 1586, 1529, 1513, 1491, 1422, 1344, 1304, 1252, 1113, 1085 cm^–1^. HRMS (ESI) *m*/*z*: [M + H]^+^ calcd for C_24_H_26_N_2_O_4_P 437.1625; found 437.1624. As an example of signal NMR assignment for these self-immolative systems, further NMR analyses were carried out for **20** (see Figures S44–S51).

#### Synthesis of 2-(Diphenylphosphanyl)ethyl Methyl(2,4,6-trimethyl-3-nitrophenyl)carbamate (**21**)

Following the general procedure 3, **7** (0.36 g, 0.82 mmol) was treated with 2-(diphenylphosphino)ethanol (0.32 g, 1.40 mmol), 4-dimethylaminopyridine (0.018 g, 0.14 mmol), and triethylamine (0.38 mL, 2.80 mmol) to provide **21** (0.11 g, 30%) as a colorless oil with a 74:26 mixture of rotamers A/B after silica-gel column chromatography (hexane/acetone 95/5). ^1^H NMR (CDCl_3_, 400 MHz) δ 7.50–7.27 (m, 10H, rotamer A + rotamer B), 7.01 (s, 1H, rotamer B), 6.99 (s, 1H, rotamer A), 4.35 (dt, 2H, ^3^*J*_HP_ = 9.5 Hz, ^3^*J* = 7.5 Hz rotamer B), 4.16 (AA′BB′, 2H, rotamer A), 3.11 (s, 3H, rotamer A), 2.97 (s, 3H, rotamer B), 2.52 (app. t, ^3^*J* = 8.0 Hz, 3H rotamer B), 2.31 (app. t, ^3^*J* = 8.0 Hz, 2H, rotamer A), 2.27 (s, 3H, rotamer A), 2.26 (s, 3H, rotamer B), 2.19 (s, 3H, rotamer A + rotamer B), 2.12 (s, 3H, rotamer A), 2.11 (s, 3H, rotamer B). ^13^C{^1^H} NMR (CDCl_3_, 100 MHz) δ 155.5 (rotamer A), 154.9 (rotamer B), 151.0 (rotamer B), 150.9 (rotamer A), 139.6 (rotamer B), 139.1 (rotamer A), 138.8 (rotamer B), 138.6 (rotamer A), 137.90 (d, ^1^*J*_cp_ = 12.0 Hz, rotamer B, Ph′ or Ph″), 137.87 (d, ^1^*J*_cp_ = 12.0 Hz, rotamer B, Ph′ or Ph″), 137.72 (d, ^1^*J*_cp_ = 12.0 Hz, rotamer A, Ph′ or Ph″), 137.68 (d, ^1^*J*_cp_ = 12.0 Hz, rotamer A, Ph′ or Ph″), 132.85 (d, ^2^*J*_CP_ = 18.0 Hz, rotamer B, Ph′ or Ph″), 132.82 (d, ^2^*J*_CP_ = 19.0 Hz, rotamer B, Ph′ or Ph″), 132.77 (d, ^2^*J*_cp_ = 18.0 Hz, rotamer A, Ph″), 132.69 (d, ^2^*J*_cp_ = 18.5 Hz, rotamer A, Ph′), 131.0 (rotamer B), 130.9 (rotamer A), 129.0–128.5 (m, 7C), 128.3 (rotamer B), 128.0 (rotamer A), 63.74 (d, ^2^*J*_cp_ = 25.5 Hz, rotamer A), 63.68 (d, ^2^*J*_cp_ = 24.0 Hz, rotamer B), 36.0 (rotamer B), 35.9 (rotamer A), 29.2 (d, ^1^*J*_cp_ = 14.5 Hz, rotamer B), 28.4 (d, ^1^*J*_cp_ = 15.0 Hz, rotamer A), 18.0 (rotamer A), 17.9 (rotamer B), 17.5 (rotamer A), 17.4 (rotamer B), 13.1 (rotamer A + rotamer B). ^31^P{^1^H} NMR (CDCl_3_, 162 MHz) δ −22.1 (rotamer B), −23.3 (rotamer A). FTIR (ATR/ν_max_) 3054–2932, 1702, 1524, 1480, 1454, 1433, 1388, 1345, 1311, 1284, 1220, 1152, 1124, 1095, 1027 cm^–1^. HRMS (ESI) *m*/*z*: [M + H]^+^ calcd for C_25_H_28_N_2_O_4_P 451.1781; found 451.1766.

#### Synthesis of 2-(Diphenylphosphanyl)ethyl Methyl(phenyl)carbamate (**22**)

Following the general procedure 3, **8** (0.24 g, 1.40 mmol) was treated with 2-(diphenylphosphino)ethanol (0.32 g, 1.40 mmol), 4-dimethylaminopyridine (0.018 g, 0.14 mmol), and triethylamine (0.38 mL, 2.80 mmol) to provide **22** (0.30 g, 59%) as a white solid after silica-gel column chromatography (hexane/EtOAc 95/5 → 90/10) followed by crystallization via vapor diffusion using a mixture of *n*-hexane and ethyl acetate to obtain colorless single crystals. Mp 53–55 °C. ^1^H NMR (CDCl_3_, 400 MHz) δ 7.47–7.37 (m, 4H), 7.37–7.29 (m, 8H), 7.25–7.17 (m, 3H), 4.27 (q, *J* = 7.5 Hz, 2H), 3.22 (s, 3H), 2.44 (t, *J* = 7.5 Hz, 2H). ^13^C{^1^H} NMR (CDCl_3_, 100 MHz) δ 155.5, 143.3, 137.9 (d, ^1^*J*_CP_ = 12.2 Hz), 132.8 (d, ^2^*J*_CP_ = 18.9 Hz), 128.9 (×2), 128.7 (d, ^3^*J*_CP_ = 6.7 Hz), 126.1, 125.8, 63.5 (d, ^2^*J*_CP_ = 25.0 Hz), 37.7, 28.4 (d, ^1^*J*_CP_ = 14.0 Hz). ^31^P{^1^H} NMR (CDCl_3_, 162 MHz) δ −22.5. FTIR (ATR/ν_max_) 3066–2961, 1688, 1595, 1493, 1451, 1433, 1393, 1351, 1307, 1295, 1283, 1151, 1040 cm^–1^. HRMS (ESI) *m*/*z*: [M + H]^+^ calcd for C_22_H_23_NO_2_P 364.1461; found 364.1458.

#### Synthesis of 2-(Diphenylphosphanyl)ethyl Mesityl(methyl)carbamate (**23**)

Following the general procedure 3, **9** (0.26 g, 1.40 mmol) was treated with 2-(diphenylphosphino)ethanol (0.32 g, 1.40 mmol), 4-dimethylaminopyridine (0.018 g, 0.14 mmol), and triethylamine (0.38 mL, 2.80 mmol) to provide **23** (0.20 g, 35%) as a white solid after silica-gel column chromatography (hexane/acetone 95/5) followed by crystallization via vapor diffusion using a mixture of *n*-hexane and ethyl acetate to obtain colorless single crystals. Mp 93–95 °C. ^1^H NMR (CDCl_3_, 400 MHz) δ 7.53–7.23 (m, 10H, rotamer A + rotamer B), 6.88 (s, 2H, rotamer A + rotamer B), 4.33 (AA′BB′, 2H, rotamer B), 4.15 (AA′BB′, 2H, rotamer A), 3.11 (s, 3H, rotamer A), 2.98 (s, 3H, rotamer B), 2.52 (AA′BB′, 2H, rotamer B), 2.33 (AA′BB′, 2H, rotamer A), 2.28 (s, 3H, rotamer A), 2.26 (s, 3H, rotamer B), 2.14 (s, 6H, rotamer A + rotamer B). ^13^C{^1^H} NMR (CDCl_3_, 100 MHz) δ 156.0 (rotamer A) 155.0 (rotamer A), 138.2 (rotamer A + rotamer B), 137.9 (d, ^1^*J*_cp_ = 12.5 Hz, rotamer A), 137.7 (d, ^1^*J*_cp_ = 12.5 Hz, rotamer B), 137.7 (rotamer A), 137.1 (br. s, rotamer A + rotamer B), 135.4 (rotamer B), 135.3 (rotamer A), 132.8 (d, ^2^*J*_cp_ = 19.0 Hz, rotamer B), 132.7 (d, ^2^*J*_cp_ = 19.0 Hz, rotamer A), 129.3 (rotamer B), 129.1 (rotamer A), 128.8 (rotamer B), 128.7 (rotamer A), 128.6 (d, ^3^*J*_cp_ = 7.0 Hz, rotamer B), 128.5 (d, ^3^*J*_cp_ = 6.5 Hz, rotamer A), 63.3 (d, ^2^*J*_cp_ = 28.0 Hz, rotamer A), 63.2 (d, ^2^*J*_cp_ = 23.0 Hz, rotamer B), 35.99 (rotamer B), 35.97 (rotamer A), 28.5 (d, ^1^*J*_cp_ = 14.5 Hz, rotamer B), 28.3 (d, ^1^*J*_cp_ = 14.5 Hz, rotamer A), 21.11 (rotamer A), 21.06 (rotamer B), 17.75 (rotamer A), 17.72 (rotamer B). ^31^P{^1^H} NMR (CDCl_3_, 162 MHz) δ −22.0 (rotamer B), −23.1 (rotamer A). FTIR (ATR/ν_max_) 3049–2915, 1707, 1655, 1584, 1483, 1454, 1432, 1390, 1348, 1308, 1277, 1217, 1172, 1148, 1097, 1070, 1027 cm^–1^. HRMS (ESI) *m*/*z*: [M + H]^+^ calcd for C_25_H_29_NO_2_P 406.1924; found 406.1930.

### General Procedure **4** for the Synthesis of Carbamate Model Compounds (**24–28**)

Sodium hydride (NaH, 60% dispersion in mineral oil, 1.1 equiv) was added to a solution of 2-naphthalenemethanol (1 equiv) in anhydrous DMF (10 mL) at 0 °C and left to stir for 15 min. Carbamoyl chloride (1 equiv) was added, and the mixture was left to stir overnight. The salts formed were filtered off, and volatiles were removed *in vacuo*. The crude product was further purified by column chromatography, followed by recrystallization via vapor diffusion using a mixture of *n*-hexane and ethyl acetate to obtain colorless single crystals.

#### Synthesis of Naphthalen-2-ylmethyl Methyl(4-nitrophenyl)carbamate (**24**)

Following the general procedure 4, 2-naphthalenemethanol (0.122 g, 0.77 mmol) was treated with NaH (0.034 g, 0.85 mmol) and **1** (0.166, 0.77 mmol) to provide **24** (0.200 g, 77%) as a white solid after silica-gel column chromatography (hexane/EtOAc 75/25). Mp 109–111 °C. ^1^H NMR (CDCl_3_, 400 MHz) δ 8.21 (d, *J* = 9.0 Hz, 2H), 7.88–7.80 (m, 4H), 7.67–7.34 (m, 5H), 5.38 (s, 2H), 3.42 (s, 3H). ^13^C{^1^H} NMR (CDCl_3_, 100 MHz) δ 154.8, 149.0, 144.6, 133.31, 133.28, 128.6, 128.1, 127.9, 127.7, 126.6, 125.9, 124.7, 124.4, 68.5, 37.2. FTIR (ATR/ν_max_) 3057–2963, 1709, 1602, 1590, 1512, 1442, 1329, 1257, 1160, 1104 cm^–1^. HRMS (ESI) *m*/*z*: [M + Na]^+^ calcd for C_19_H_16_O_4_N_2_Na 359.1002; found 359.0999.

#### Synthesis of Naphthalen-2-ylmethyl Methyl(2-methyl-4-nitrophenyl)carbamate (**25**)

Following the general procedure 4, 2-naphthalenemethanol (0.122 g, 0.77 mmol) was treated with NaH (0.034 g, 0.85 mmol) and **2** (0.176 g, 0.77 mmol) to provide **25** (0.197 g, 73%) as an off-white solid after silica-gel column chromatography (hexane/EtOAc 75/25). Mp 99–101 °C. ^1^H NMR (CDCl_3_, 400 MHz) δ 8.08 (br. s, 1H, rotamer A + rotamer B), 8.09–8.04 (m, 1H, rotamer A + rotamer B), 7.90–7.71 (m, 3H, rotamer A + rotamer B), 7.63 (br. s, 1H, rotamer A + rotamer B), 7.55–7.44 (m, 2H, rotamer A + rotamer B), 7.38–7.27 (m, 2H, rotamer A + rotamer B), 5.43–5.18 (m, 2H, rotamer A + rotamer B), 3.24 (br. s, 3H, rotamer A + rotamer B), 2.32 (s, 3H, rotamer B), 2.24 (s, 3H, rotamer A). ^13^C{^1^H} NMR (CDCl_3_, 100 MHz) δ 154.8, 147.4, 146.6, 137.7, 133.5, 133.0, 128.4, 128.3, 127.9, 127.7, 127.4, 127.1, 126.3, 126.3, 126.0, 125.8, 125.5, 122.2, 67.9, 37.3, 17.9. FTIR (ATR/ν_max_) 3070–2956, 1698, 1582, 1514, 1431, 1304, 1155, 1088 cm^–1^. HRMS (ESI) *m*/*z*: [M + Na]^+^ calcd for C_20_H_18_O_4_N_2_Na 373.1159; found 373.1155.

#### Synthesis of Naphthalen-2-ylmethyl (2,6-Dimethyl-4-nitrophenyl)(methyl)carbamate (**26**)

Following the general procedure 4, 2-naphthalenemethanol (0.100 g, 0.63 mmol) was treated with NaH (0.028 g, 0.70 mmol) and **3** (0.153 g, 0.63 mmol) to provide **26** (0.178 g, 77%) as an off-white solid after silica-gel column chromatography (hexane/EtOAc 75/25). Mp 87–89 °C. ^1^H NMR (CDCl_3_, 400 MHz) δ 7.97 (s, 2H, rotamer B), 7.95 (s, 2H, rotamer A), 7.91–7.84 (m, 1H, rotamer A + rotamer B), 7.82–7.79 (m, 1H, rotamer A + rotamer B), 7.77 (app. d, *J* = 8.0 Hz, 1H, rotamer A + rotamer B), 7.74–7.72 (m, 1H, rotamer A + rotamer B), 7.62 (s, 1H, rotamer A), 7.55 (s, 1H, rotamer B), 7.54–7.44 (m, 2H, rotamer A + rotamer B), 7.28–7.23 (m, 1H, rotamer A + rotamer B), 5.40 (s, 2H, rotamer B), 5.25 (s, 2H, rotamer A), 3.18 (s, 3H, rotamer B), 3.15 (s, 3H, rotamer A), 2.30 (s, 6H, rotamer B), 2.22 (s, 6H, rotamer A). ^13^C{^1^H} NMR (CDCl_3_, 100 MHz) δ 155.0, 154.6, 146.7, 146.1, 138.2, 138.0, 133.7, 133.3, 133.2, 133.2, 128.6, 128.4, 128.1, 128.1, 127.9, 127.8, 127.4, 127.2, 126.5, 126.5, 126.4, 126.4, 125.9, 125.7, 123.7, 123.6, 68.0, 67.8, 35.8, 35.4, 18.1. FTIR (ATR/ν_max_) 3052–2928, 1693, 1602, 1518, 1479, 1435, 1303, 1168, 1088 cm^–1^. HRMS (ESI) *m*/*z*: [M + Na]^+^ calcd for C_21_H_20_O_4_N_2_Na 387.1315; found 387.1316.

#### Synthesis of Naphthalen-2-ylmethyl Methyl(3-nitrophenyl)carbamate (**27**)

Following the general procedure 4, 2-naphthalenemethanol (0.122 g, 0.77 mmol) was treated with NaH (0.034 g, 0.85 mmol) and **4** (0.166 g, 0.77 mmol) to provide **27** (0.160 g, 62%) as a white solid after silica-gel column chromatography (hexane/EtOAc 75/25). Mp 85–87 °C. ^1^H NMR (CDCl_3_, 400 MHz) δ 8.19 (app. t, *J* = 2.0 Hz, 1H), 8.06 (dd, *J* = 8.0, 2.0 Hz, 1H), 7.87–7.78 (m, 4H), 7.65 (d, *J* = 8.0 Hz, 1H), 7.54–7.41 (m, 4H), 5.36 (s, 2H), 3.41 (s, 3H). ^13^C{^1^H} NMR (CDCl_3_, 100 MHz) δ 155.1, 148.6, 144.4, 133.4, 133.3, 131.3, 129.6, 128.6, 128.2, 127.9, 127.5, 126.5, 126.5, 125.9, 120.6, 120.3, 68.3, 37.5. FTIR (ATR/ν_max_) 3090–2925, 1703, 1615, 1579, 1528, 1486, 1443, 1348, 1336, 1251, 1169, 1122, 1026 cm^–1^. HRMS (ESI) *m*/*z*: [M + Na]^+^ calcd for C_19_H_16_O_4_N_2_Na 359.1002; found 359.0998.

#### Synthesis of Naphthalen-2-ylmethyl Methyl(phenyl)carbamate (**28**)

Following the general procedure 4, 2-naphthalenemethanol (0.122 g, 0.77 mmol) was treated with NaH (0.034 g, 0.85 mmol) and **8** (0.131 g, 0.77 mmol) to provide **28** (0.180 g, 80%) as a white solid after silica-gel column chromatography (hexane/EtOAc 75/25). Mp 93–95 °C. ^1^H NMR (CDCl_3_, 400 MHz) δ 7.84–7.70 (m, 4H), 7.50–7.45 (m, 2H), 7.44–7.39 (m, 1H), 7.39–7.32 (m, 2H), 7.25–7.19 (m, 3H), 5.32 (s, 2H), 3.34 (s, 3H). ^13^C{^1^H} NMR (CDCl_3_, 100 MHz) δ 155.6, 143.4, 134.2, 133.3, 133.1, 129.0, 128.3, 128.1, 127.8, 126.9 (br. s), 126.3 (br. s), 126.2, 126.0 (br. s), 125.7, 67.5, 38.0. FTIR (ATR/ν_max_) 3060–2946, 1697, 1597, 1438, 1397, 1362, 1337, 1261, 1180, 1119 cm^–1^. HRMS (ESI) *m*/*z*: [M + H]^+^ calcd for C_19_H_18_O_2_N 292.1332; found 292.1332.

### General Procedure for the Alkylation Reactions of Self-Immolative Systems (**15–23**)

^1^H NMR spectroscopic studies were conducted by dissolution of the various disclosure systems (**15**–**23**) in MeCN-*d*_3_ (0.5 mL, [disclosure system]_0_ = 0.025 mol·L^–1^), followed by the addition of 10 molar equiv of benzyl bromide (BnBr) directly to the NMR tube. The ^1^H NMR spectra were recorded at regular time intervals. The area of the singlet resonance *CH*_*3*_N of **15**–**23** was standardized to the area of the MeCN solvent resonance at 1.94 ppm and was used to calculate the percentage of disclosure system that remained nonalkylated over time with respect to the initial area of the methyl resonance (*i.e*., before the addition of the alkylating agent, time point = 0). The same procedure was employed for the solvent screening of self-immolative system **19**.

### General Procedure for the β-Elimination Reactions of Self-Immolative Systems **15a–23a**

^1^H NMR spectroscopic studies were conducted by addition of 2 molar equiv of *N*,*N*-diisopropylethylamine (DIPEA) to the corresponding alkylated disclosure system (**15a**–**23a**) directly to the NMR tube. The percentage of alkylated disclosure system **15a**–**23a** remaining over time was calculated by comparison of the integrations of the *CH*_*3*_N or aromatic ortho- aromatic proton of the reporter moiety prior to β-elimination and standardized to the area of the MeCN solvent resonance at 1.94 ppm. The same procedure was employed for the solvent screening of self-immolative system **19a**.

### General Procedure for the “One-Pot” Reactions of Self-Immolative Systems **18–20**

^1^H NMR spectroscopic studies were conducted by addition of 10 molar equiv of benzyl bromide to a solution of the corresponding self-immolative disclosure system **18–20** (V = 0.5 mL, [disclosure system] = 0.025 mol·L^–1^) and 2 molar equiv of DIPEA directly to the NMR tube. The ^1^H NMR spectra were recorded at regular time intervals. The area of the singlet C*H*_*3*_N for the self-immolative disclosure systems **18** and **20**, the area of the singlet C*H*_Ar_ for the self-immolative disclosure system **19**, the area of the multiplet resonance C*H*_2_P^+^ for the self-immolative alkylated disclosure system **18a**–**20a** as well as the area for the vinyl proton corresponding to the phosphonium salt **29** were used to calculate the percentage of the different species remaining over time (*i.e*., before the addition of BnBr, time point = 0).

## Supporting Information Available

^1^H NMR, ^13^C NMR, and ^31^P spectra; calculations of rotational barriers; and X-ray crystallographic data of CCDC 1980913 (**22**), 1962710 (**23**), 1908144 (**24**), 1908143 (**25**), 1908145 (**26**), 1908146 (**27**), and 1980598 (**28**) (PDF)

## Accession Codes

CCDC 1908143–1908146, 1962710, 1980598, and 1980913 contain the supplementary crystallographic data for this paper. These data can be obtained free of charge via www.ccdc.cam.ac.uk/data_request/cif, or by emailing data_request@ccdc.cam.ac.uk, or by contacting The Cambridge Crystallographic Data Centre, 12 Union Road, Cambridge CB2 1EZ, UK; fax: +44 1223 336033.

## Footnotes

a When the reporter group is unsymmetrically substituted, the molecule is chiral and, as a result, the phenyl groups attached to the phosphine are diastereotopic.

b With respect to the *syn*/*anti* conformation of the carbamate unit, in agreement with Smith et al., we observe in solution that the methylene protons in the α-position to the carbamate group of the major rotamer are always at the higher field.^35^
