# Supplementary material for: Self-Immolative System for Disclosure of Reactive Electrophilic Alkylating Agents: Understanding the Role of the Reporter Group
Source: J Org Chem. 2021 Jul 22;86(15):10263–79. doi: 10.1021/acs.joc.1c00996 (PMC8389931; doi:10.1021/acs.joc.1c00996)
Supplement: Supplementary file 1 — jo1c00996_si_001.pdf [file jo1c00996_si_001.pdf]

A self-immolative system for disclosure of reactive electrophilic alkylating agents - Understanding the role of the reporter group

Alexander G. Gavriel,[a] Flavien Leroux,[a] Gurjeet S. Khurana,[a] Viliyana G. Lewis,[a] Ann M. Chippindale,[a] Mark R. Sambrook,[b] Wayne Hayes[a] and Andrew T. Russell[a]*

[a] Department of Chemistry, University of Reading, Whiteknights, Reading, RG6 6AD, UK
[b] CBR Division, Defence Science & Technology Laboratory (Dstl), Porton Down, Salisbury, Wiltshire, SP4 0JQ, UK
Email: a.t.russell@reading.ac.uk

Supporting Information

Contents | **Page No.**

Contents	Page No.
NMR Characterization of compounds **1-14**	S2
NMR Characterization of self-immolative systems **15-23**	S16
Variable Temperature ^{1}H and $^{31}P\{^{1}H\}$ NMR of self-immolative systems **16, 17**, **19** and **20**	S32
Calculation of rotational barriers for self-immolative systems **16, 17**, **19** and **20**	S40
Degradation study in solution for self-immolative systems **15-23**	S41
Degradation study for neat self-immolative systems **15-23**	S46
NMR Characterization of crystalline model carbamate compounds **24-28**	S51
X-ray characterization of crystalline compounds **22-28**	S56
Alkylation data for self-immolative system **15-23**	S63
β-elimination data for alkylated self-immolative systems **15a-23a**	S68
Solvent screening for self-immolative system **19**	S75
One-pot alkylation/elimination data for self-immolative systems **18-20**	S78
UV-visible data for self-immolative system **19**	S87
Reference	S88

NMR Characterization of compounds 1-14

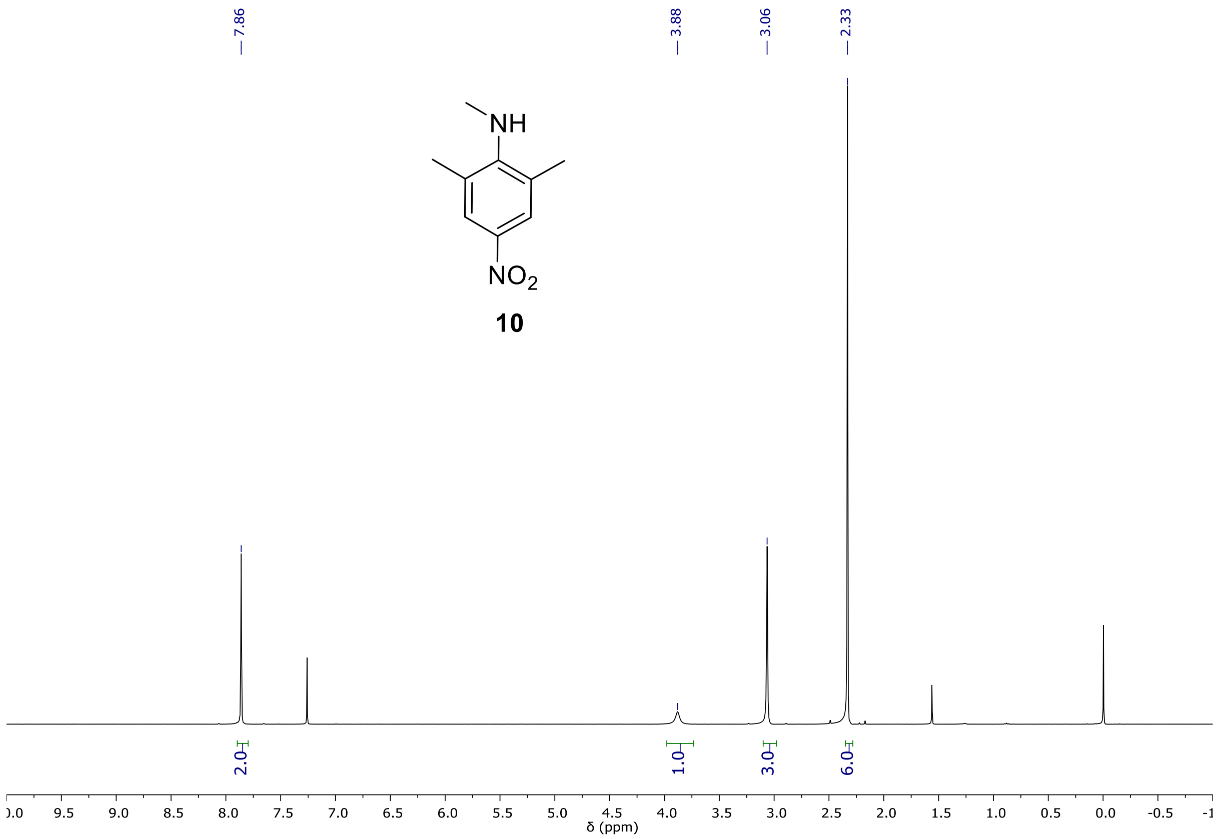

Figure S1. ^{1}H NMR spectra of **10** ($CDCl_3$, 400 MHz).

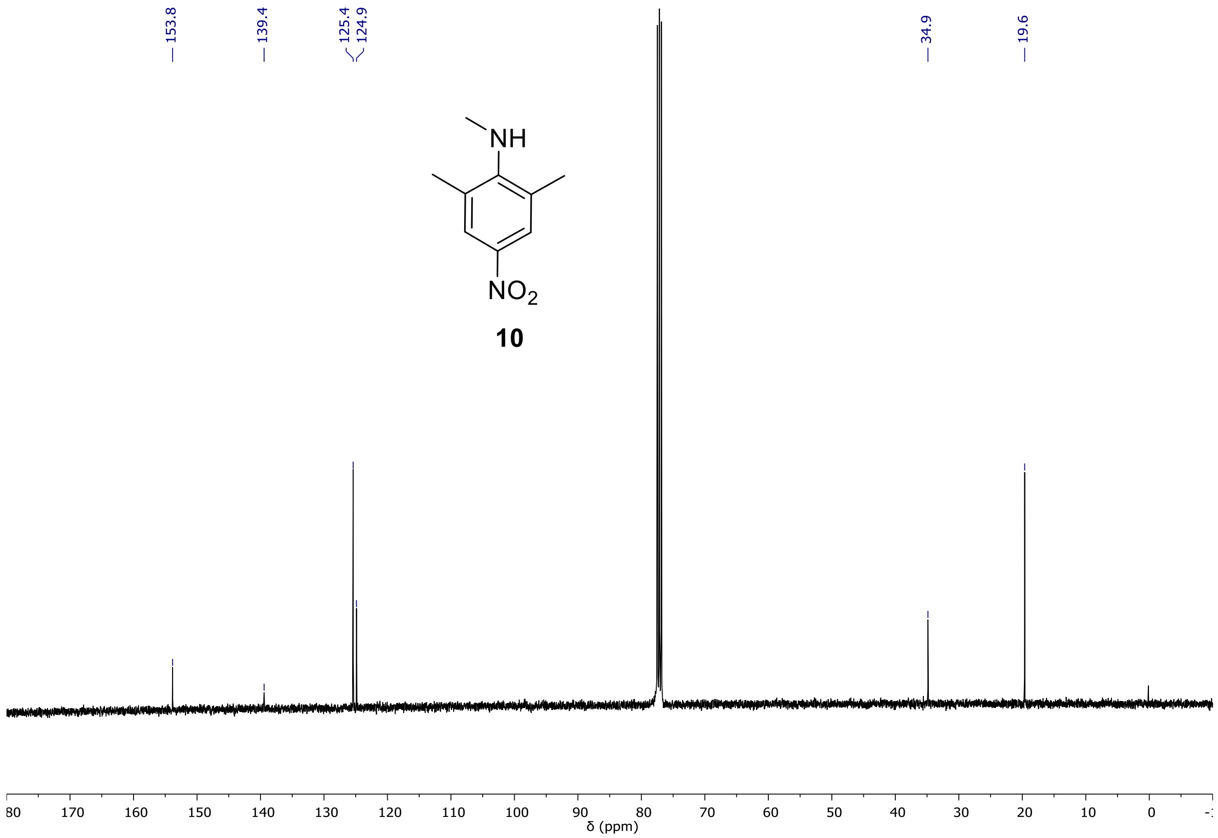

Figure S2. $^{13}C\{^{1}H\}$ NMR spectra of **10** ($CDCl_3$, 100 MHz).

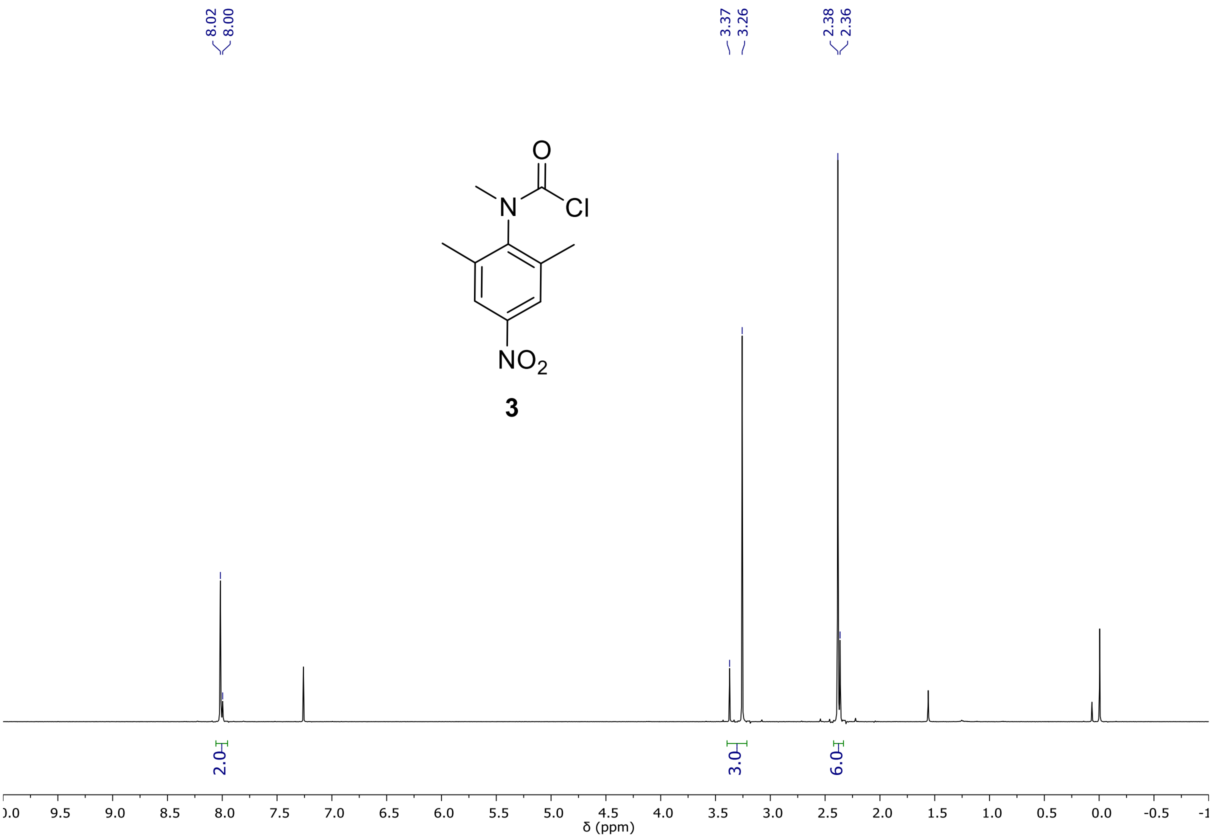

Figure S3. ^{1}H NMR spectra of **3** ($CDCl_3$, 400 MHz).

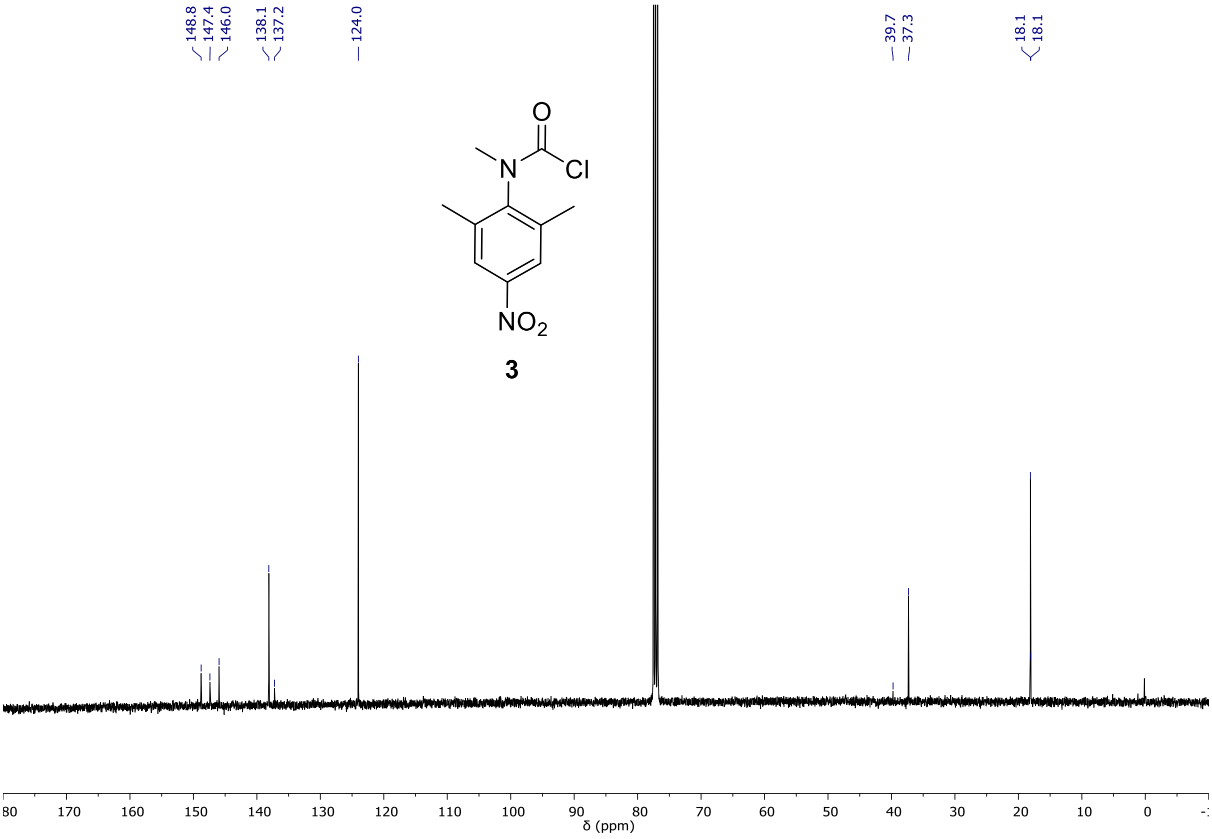

Figure S4. $^{13}C\{^{1}H\}$ NMR spectra of **3** ($CDCl_3$, 100 MHz).

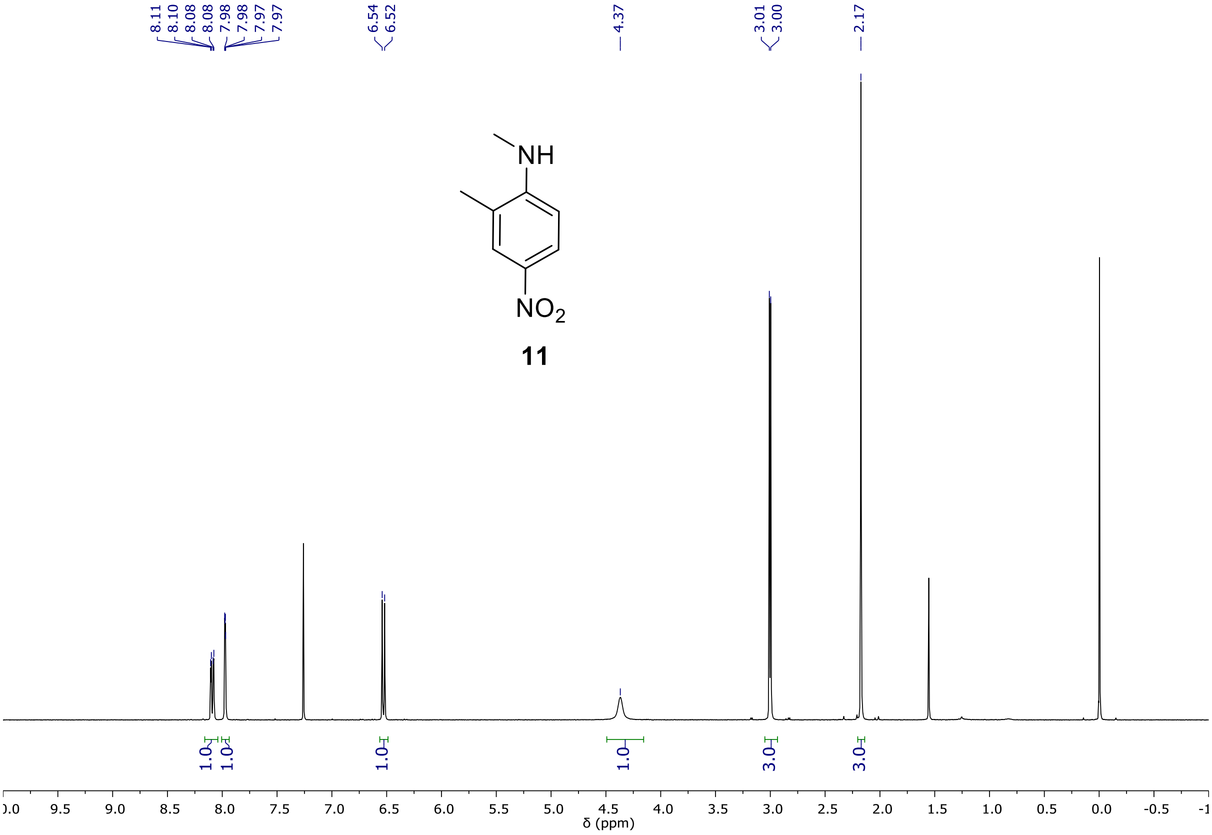

11

Figure S5. ^{1}H NMR spectra of **11** ($CDCl_3$, 400 MHz).

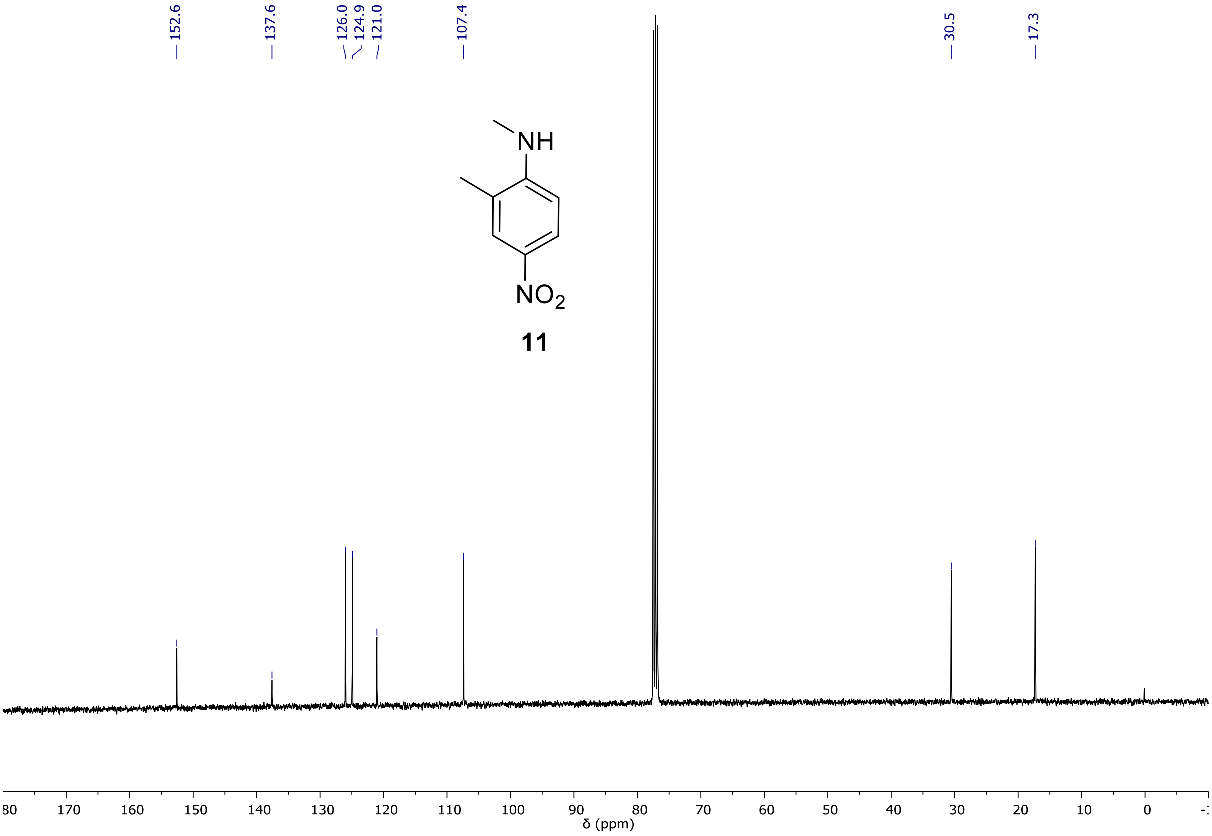

11

Figure S6. $^{13}C\{^1H\}$ NMR spectra of **11** ($CDCl_3$, 100 MHz).

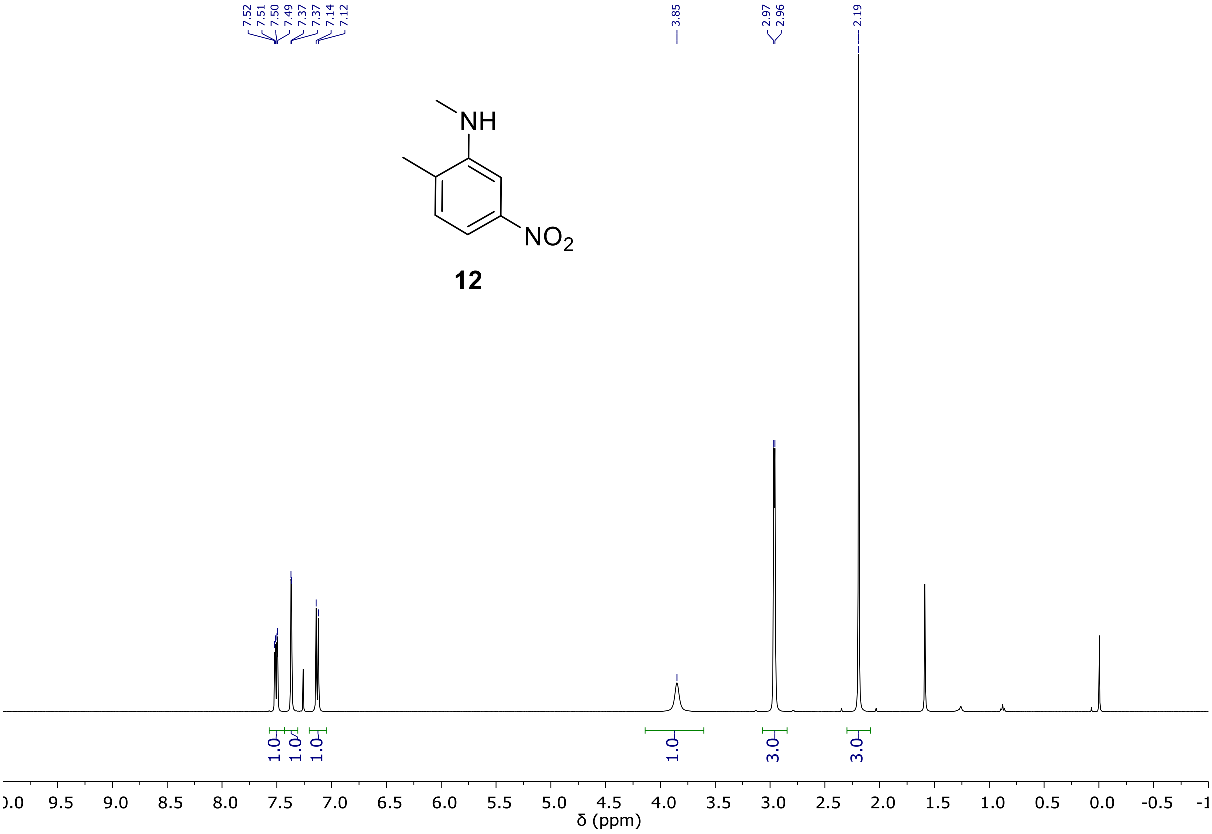

Figure S7. ^{1}H NMR spectra of **12** ($CDCl_3$, 400 MHz).

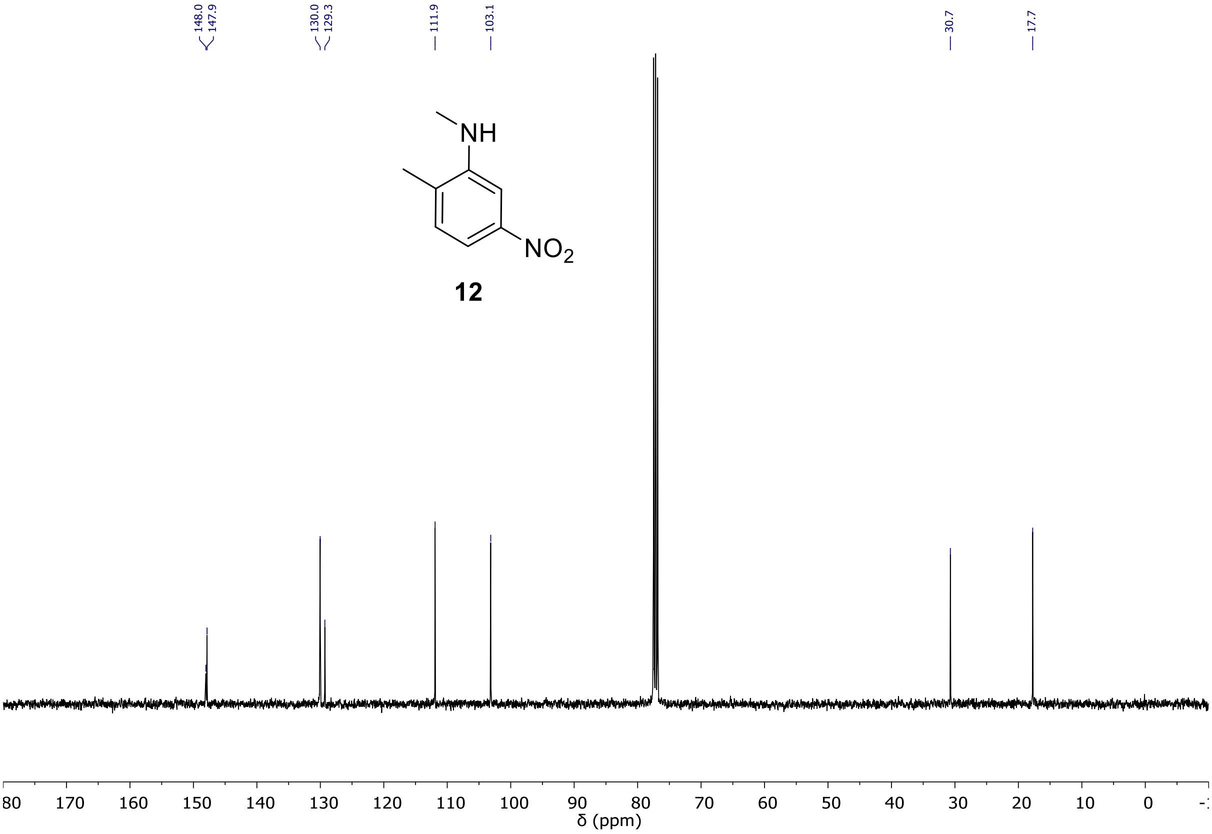

Figure S8. $^{13}C\{^{1}H\}$ NMR spectra of **12** ($CDCl_3$, 100 MHz).

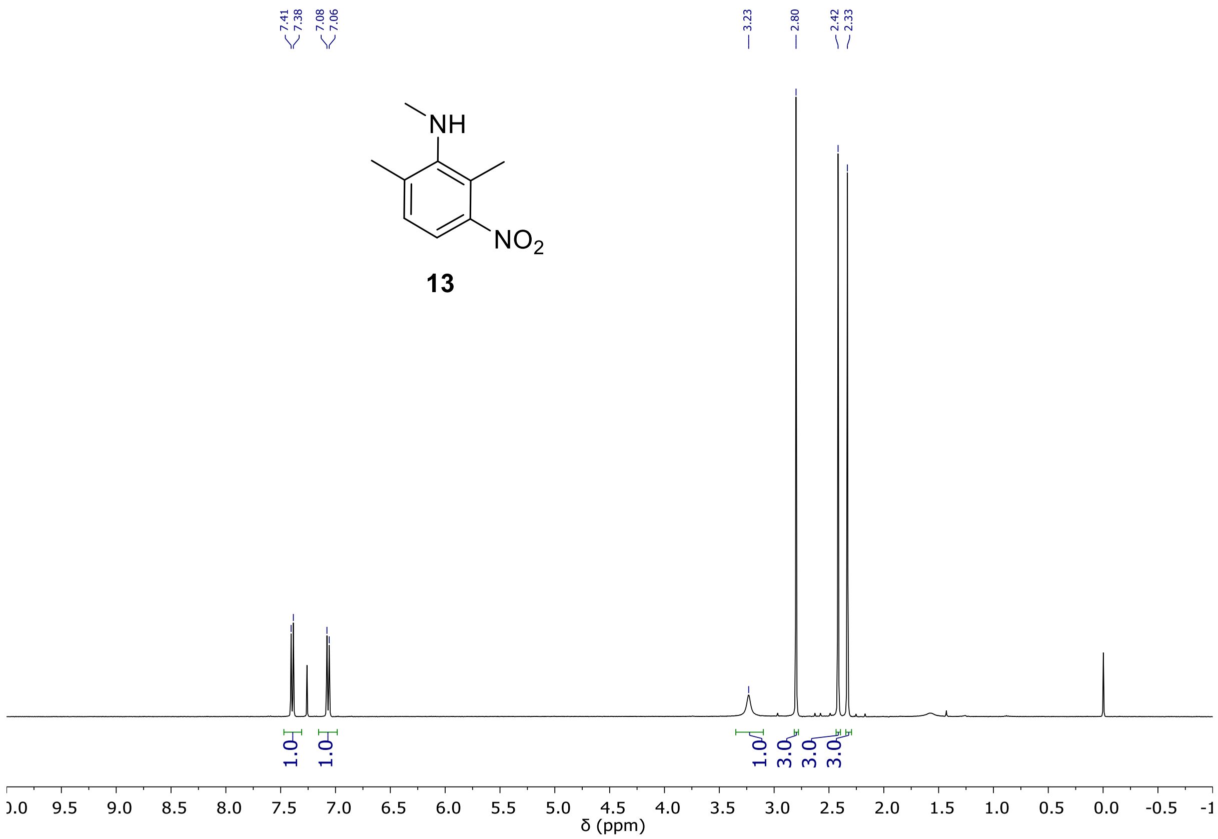

Figure S9. ^{1}H NMR spectra of **13** ($CDCl_3$, 400 MHz).

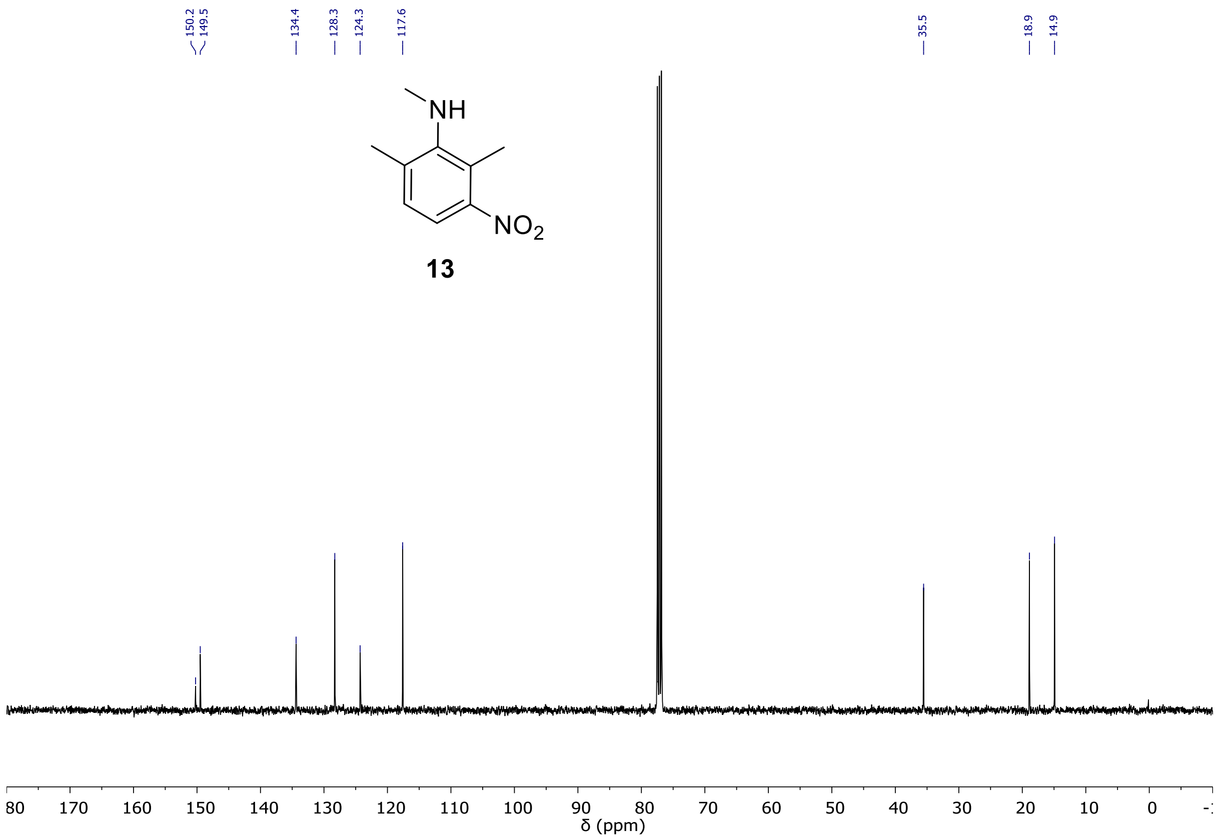

Figure S10. $^{13}C\{^{1}H\}$ NMR spectra of **13** ($CDCl_3$, 100 MHz).

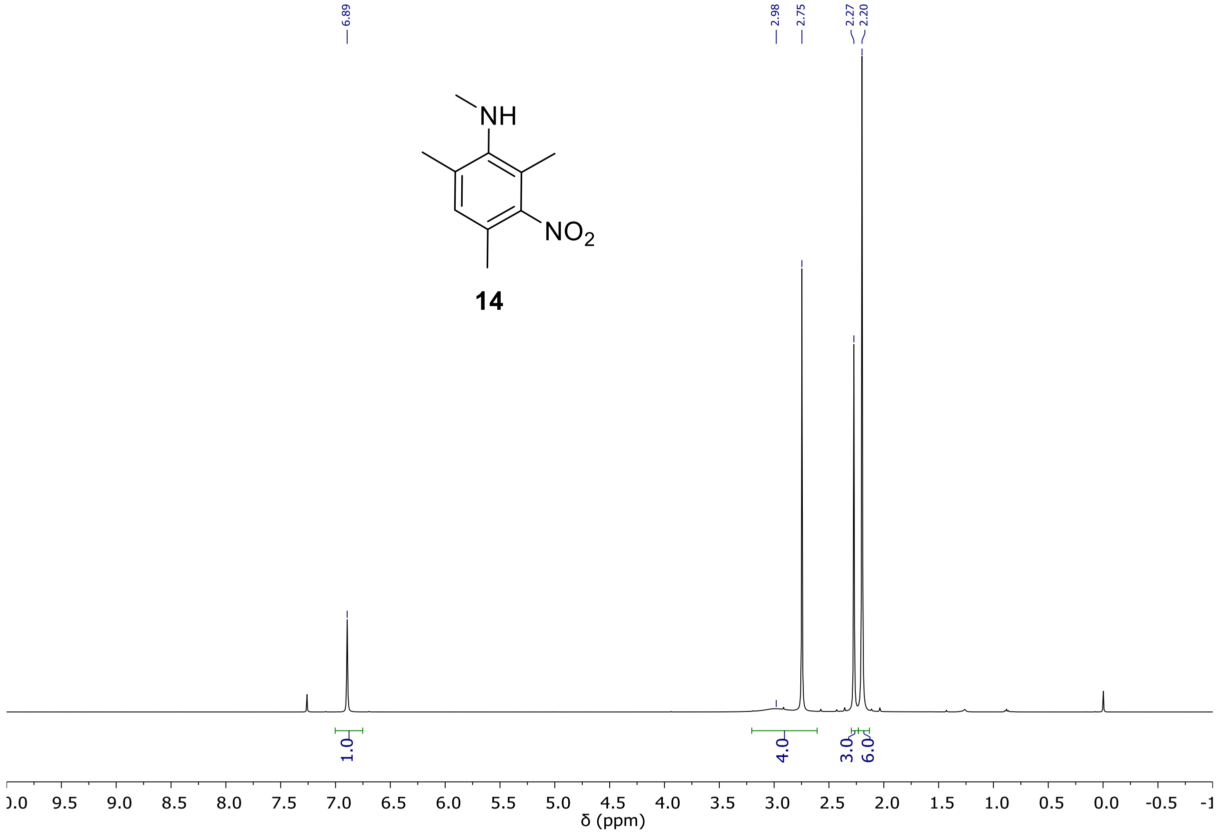

Figure S11. ^{1}H NMR spectra of **14** ($CDCl_3$, 400 MHz).

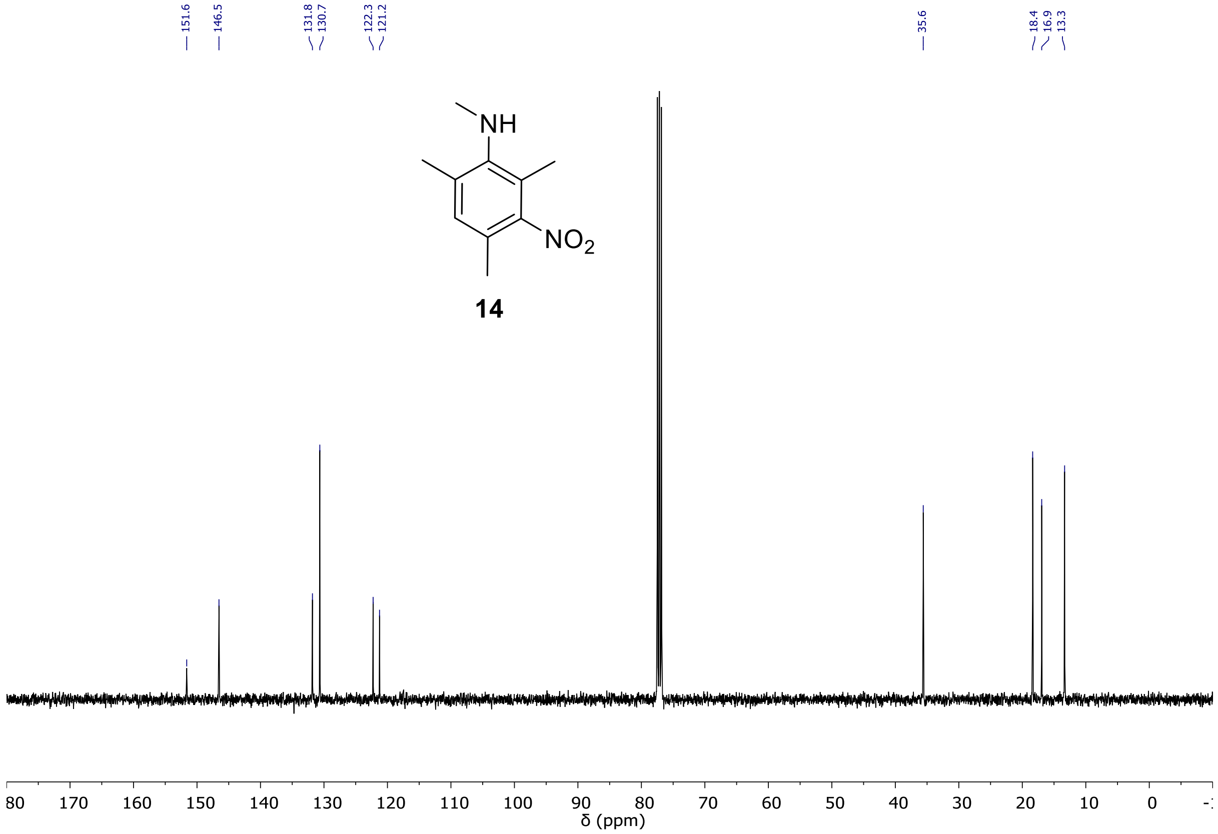

Figure S12. $^{13}C\{^{1}H\}$ NMR spectra of **14** ($CDCl_3$, 100 MHz).

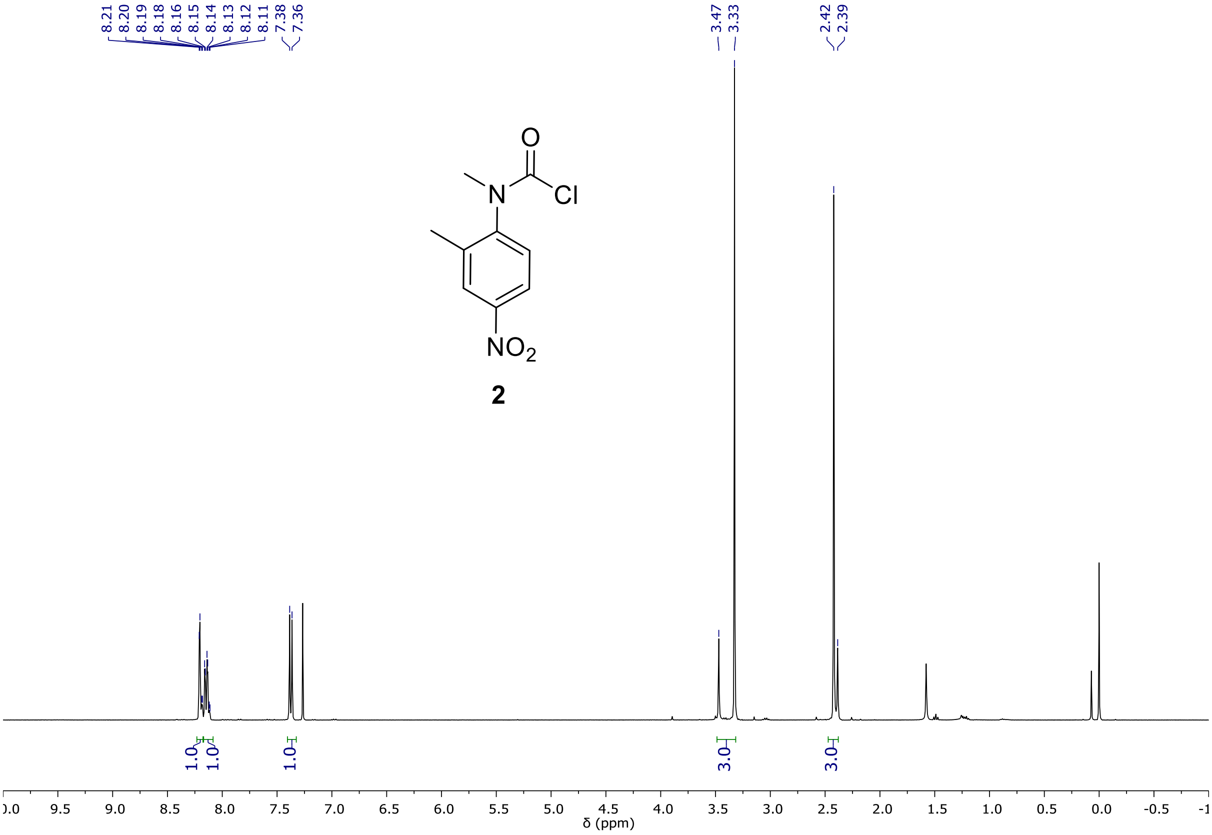

Figure S13. ^{1}H NMR spectra of **2** ($CDCl_3$, 400 MHz).

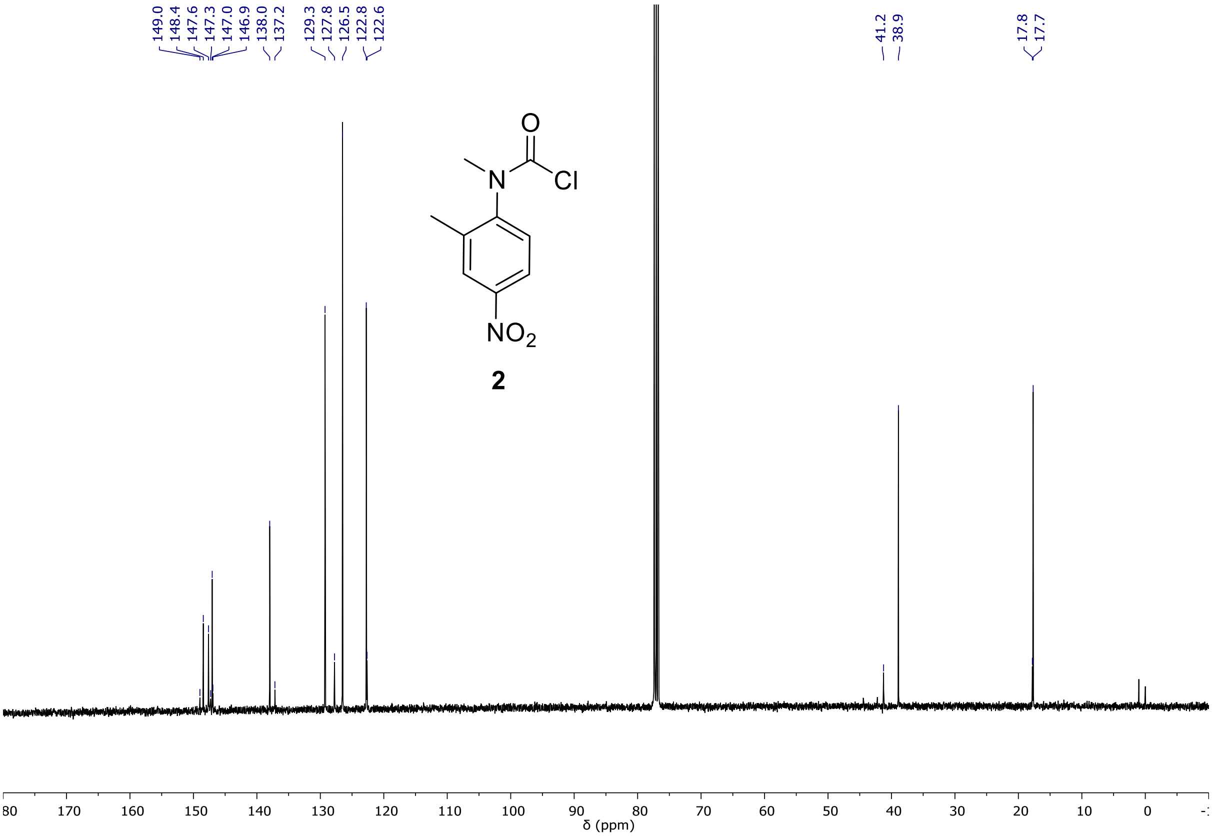

Figure S14. $^{13}C\{^{1}H\}$ NMR spectra of **2** ($CDCl_3$, 100 MHz).

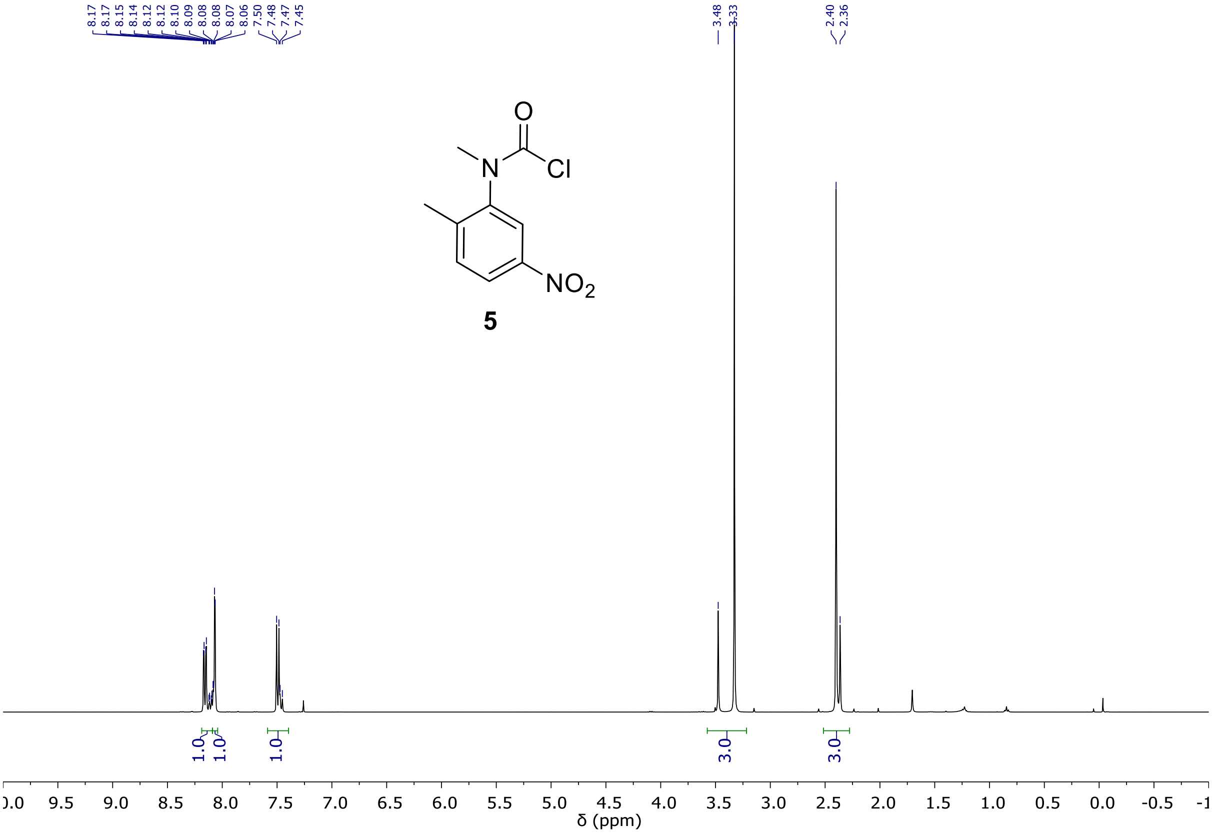

Figure S15. ^{1}H NMR spectra of **5** ($CDCl_3$, 400 MHz).

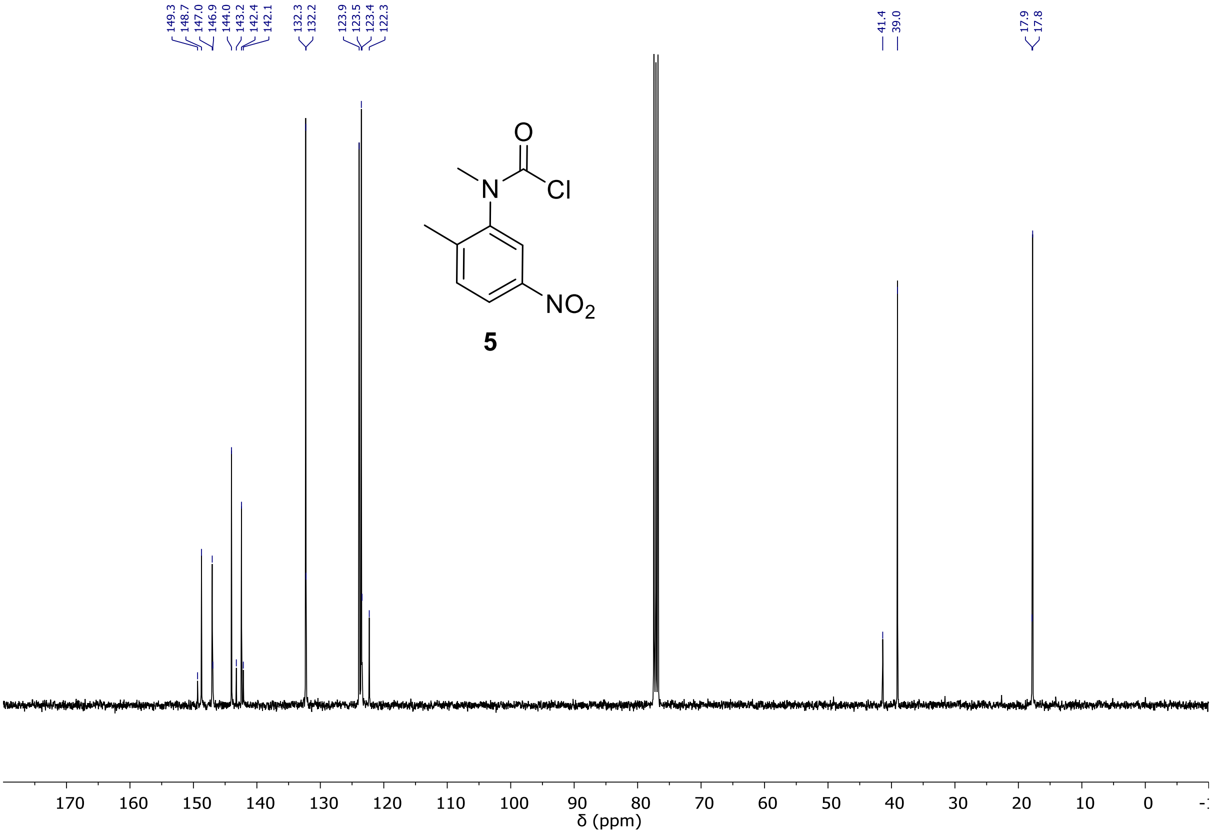

Figure S16. $^{13}C\{^{1}H\}$ NMR spectra of **5** ($CDCl_3$, 100 MHz).

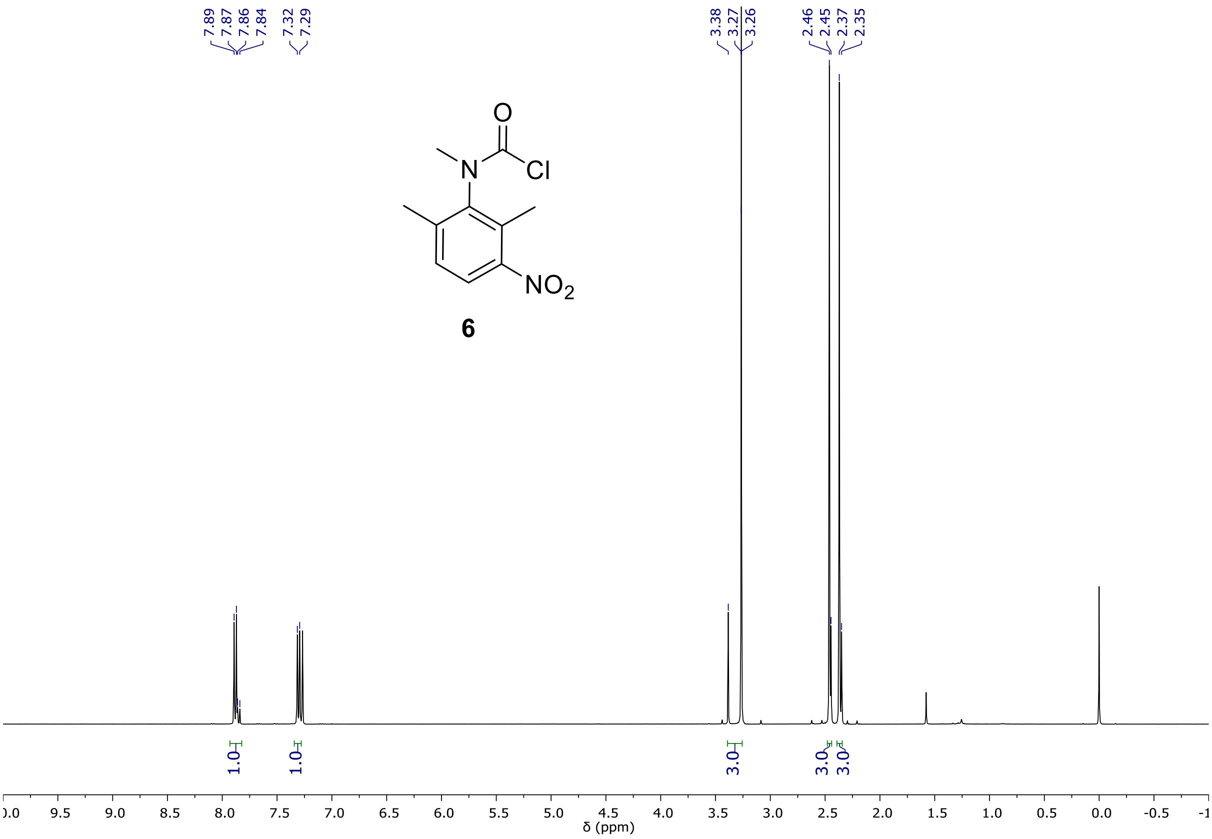

Figure S17. ^{1}H NMR spectra of **6** ($CDCl_3$, 400 MHz).

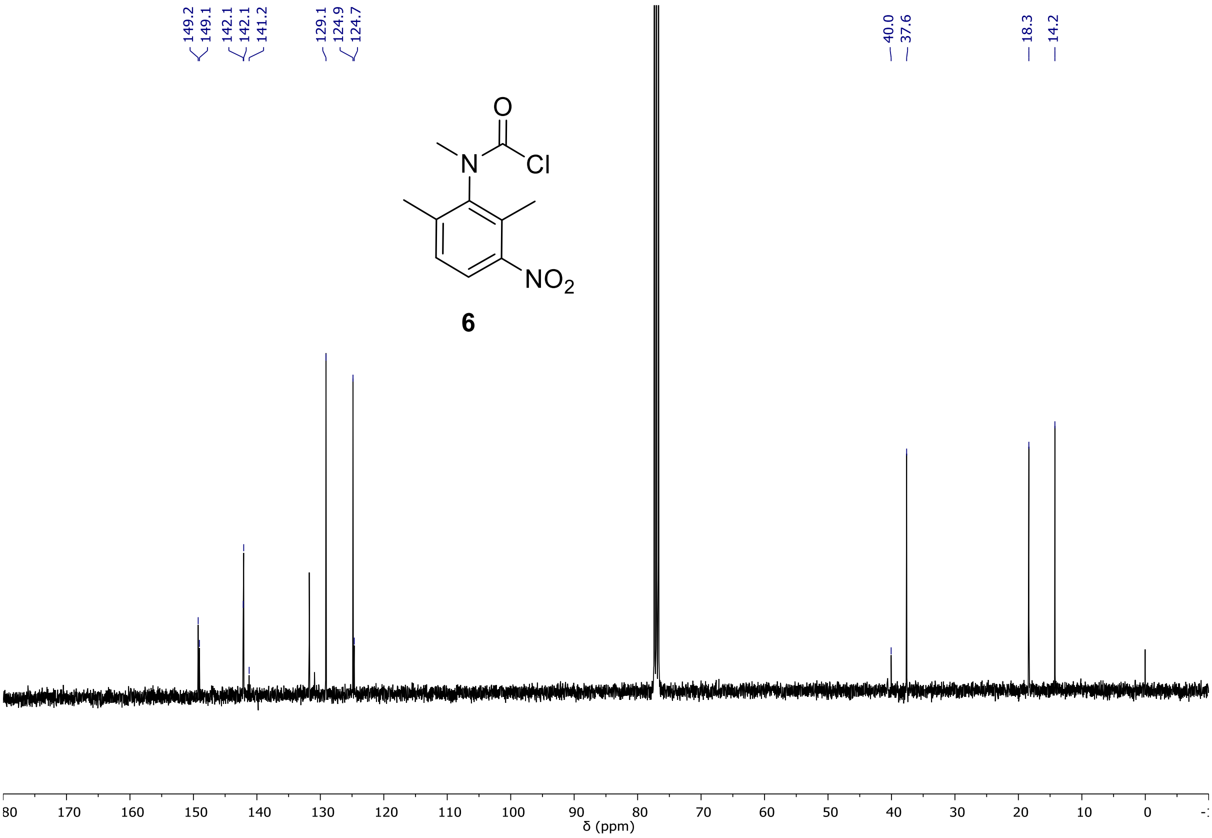

Figure S18. $^{13}C\{^1H\}$ NMR spectra of **6** ($CDCl_3$, 100 MHz).

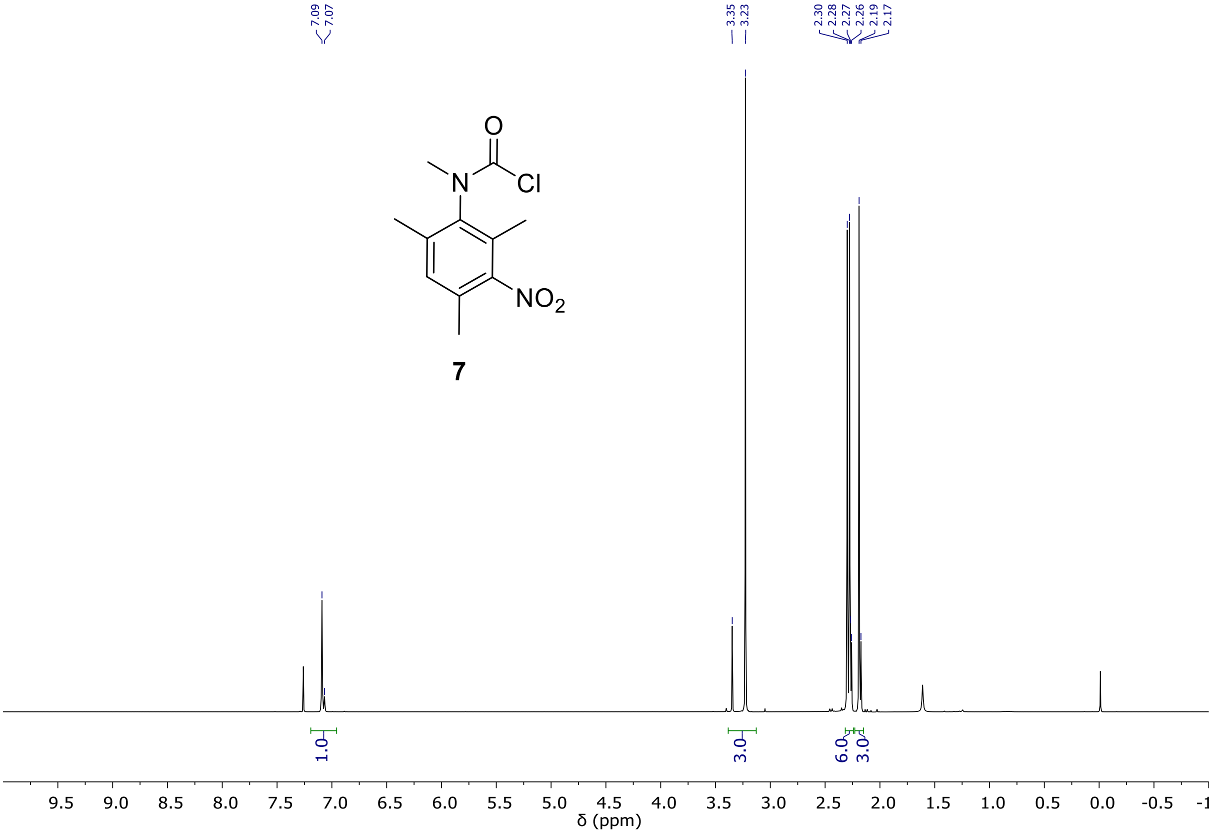

Figure S19. ^{1}H NMR spectra of **7** ($CDCl_3$, 400 MHz).

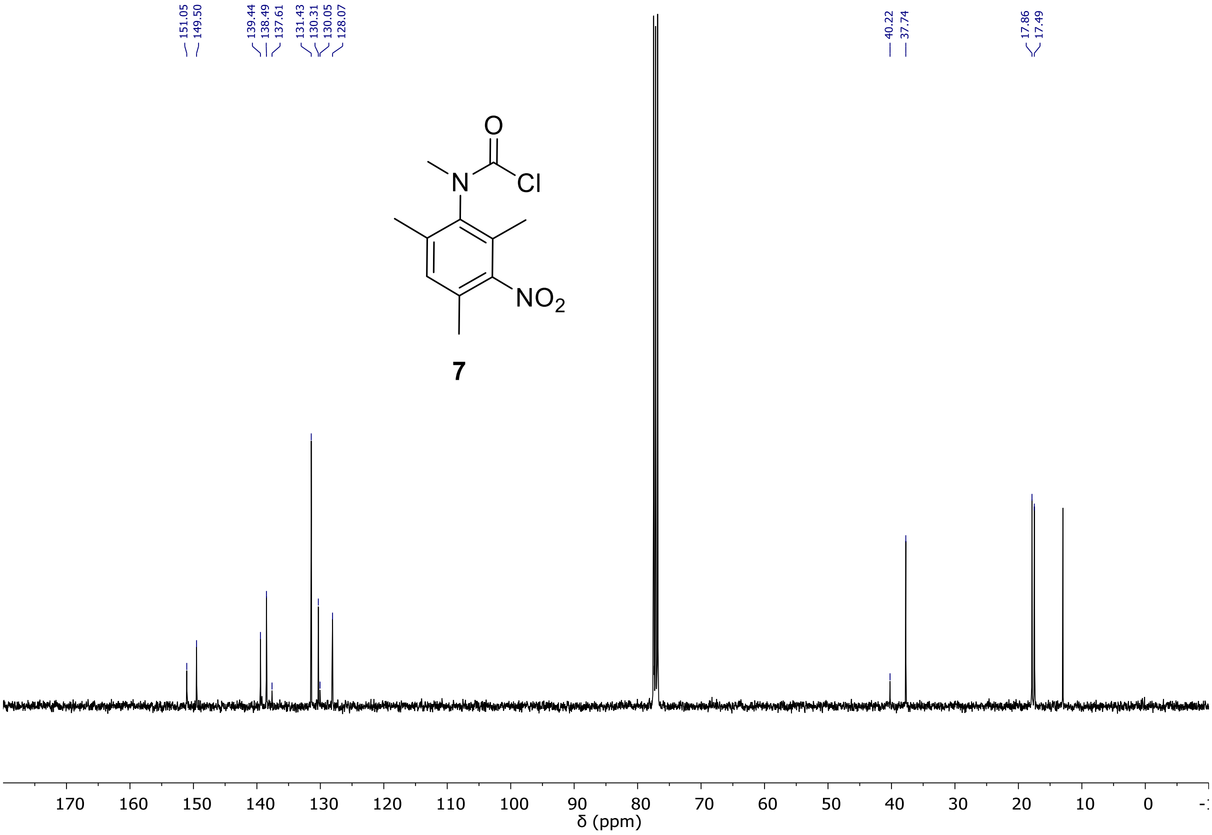

Figure S20. $^{13}C\{^{1}H\}$ NMR spectra of **7** ($CDCl_3$, 100 MHz).

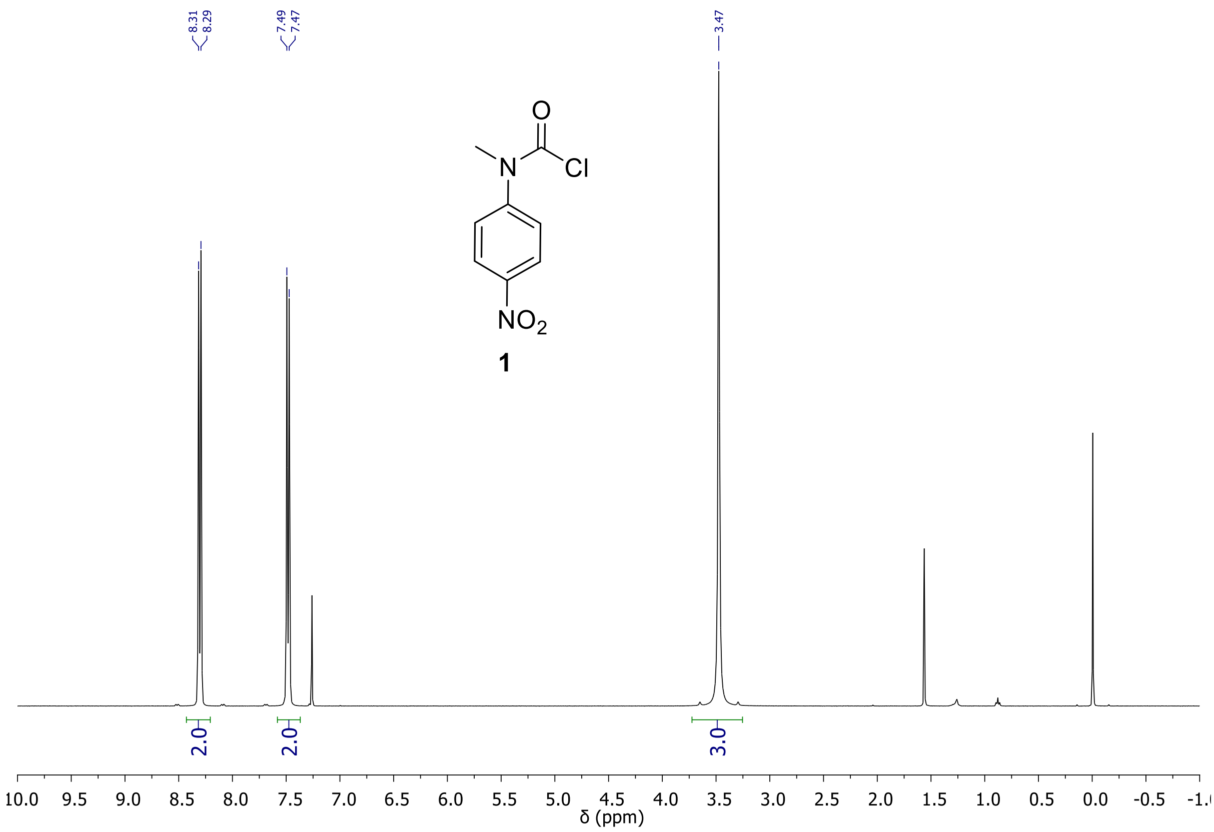

Figure S21. ^{1}H NMR spectra of **1** ($CDCl_3$, 400 MHz).

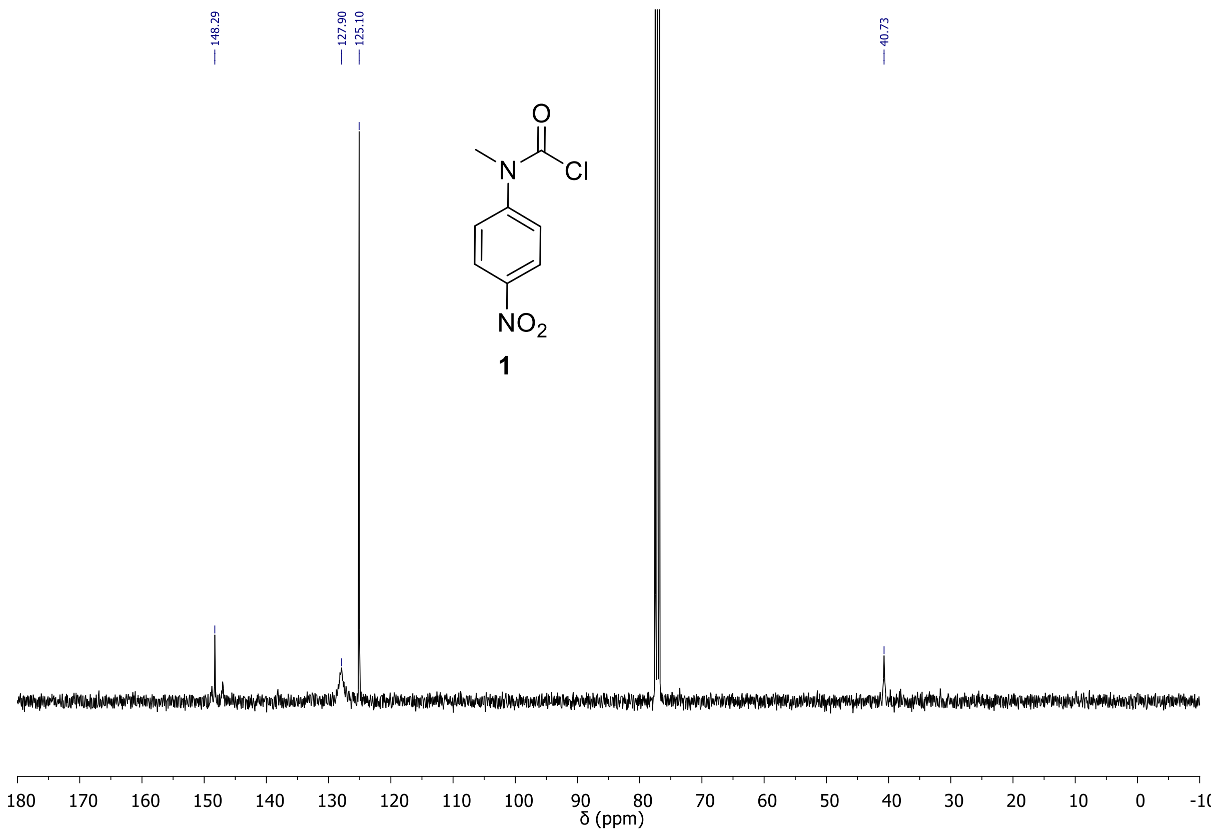

Figure S22. $^{13}C\{^{1}H\}$ NMR spectra of **1** ($CDCl_3$, 100 MHz).

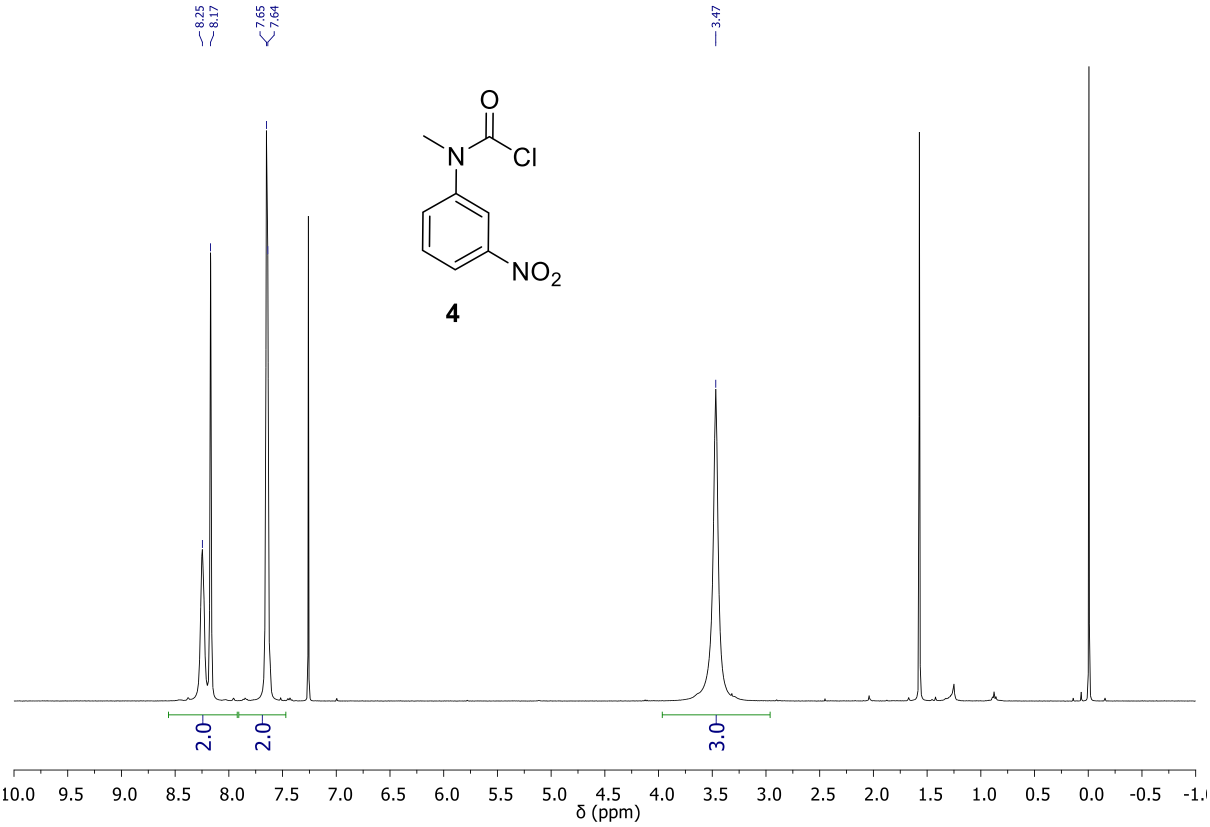

Figure S23. ^{1}H NMR spectra of **4** ($CDCl_3$, 400 MHz).

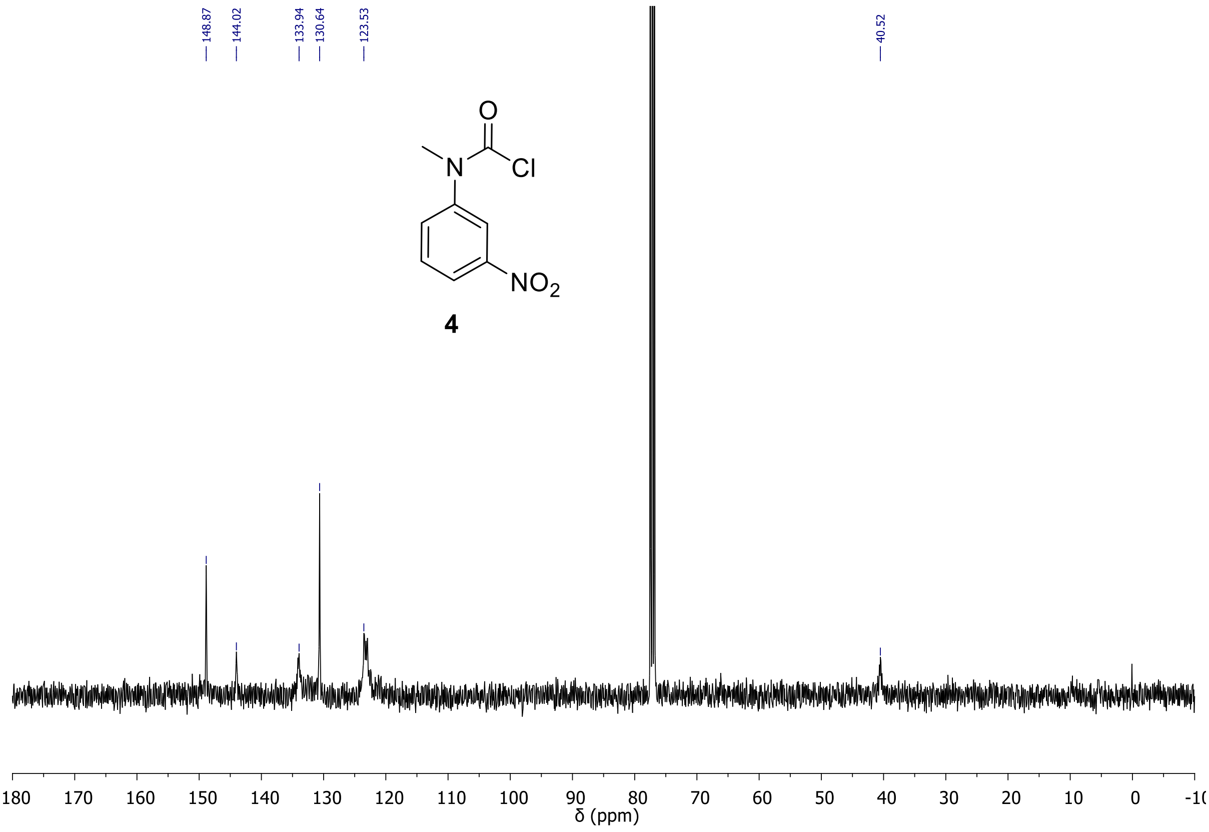

Figure S24. $^{13}C\{^{1}H\}$ NMR spectra of **4** ($CDCl_3$, 100 MHz).

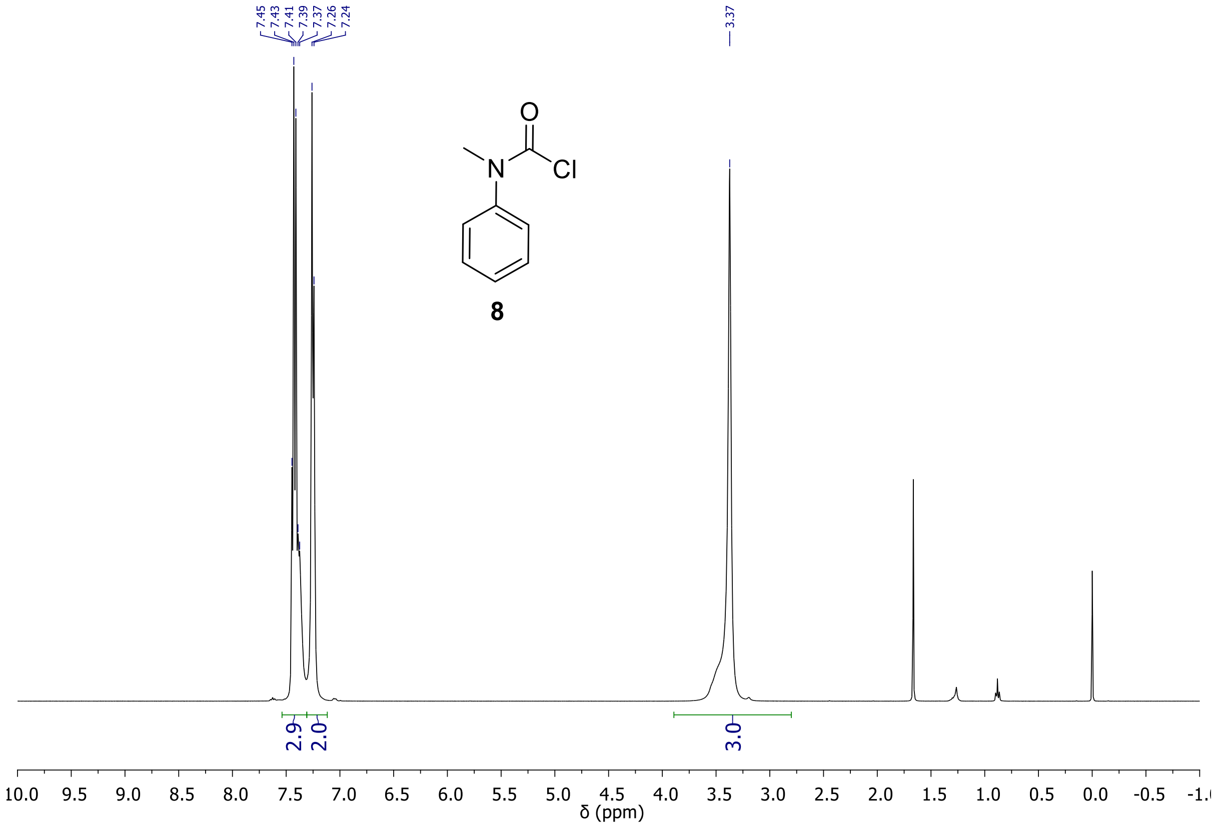

Figure S25. ^{1}H NMR spectra of **8** ($CDCl_3$, 400 MHz).

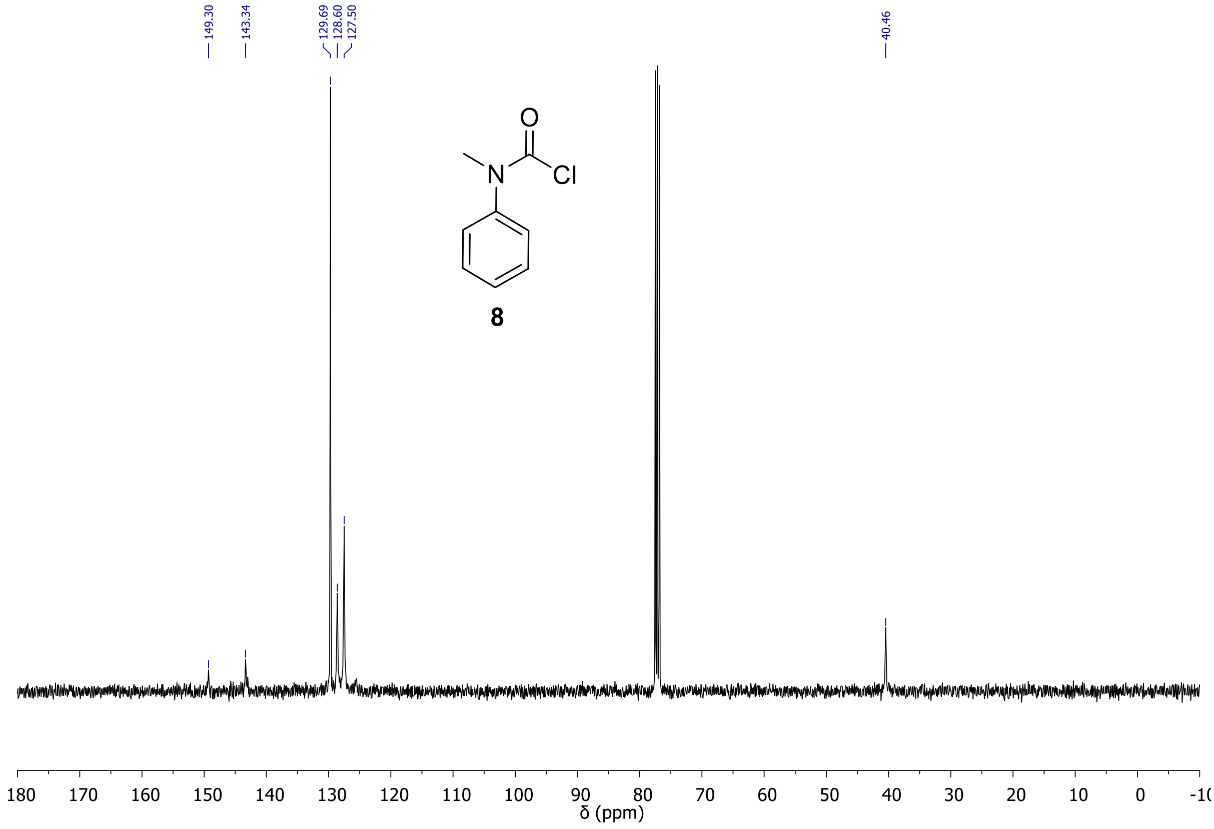

Figure S26. $^{13}C\{^{1}H\}$ NMR spectra of **8** ($CDCl_3$, 100 MHz).

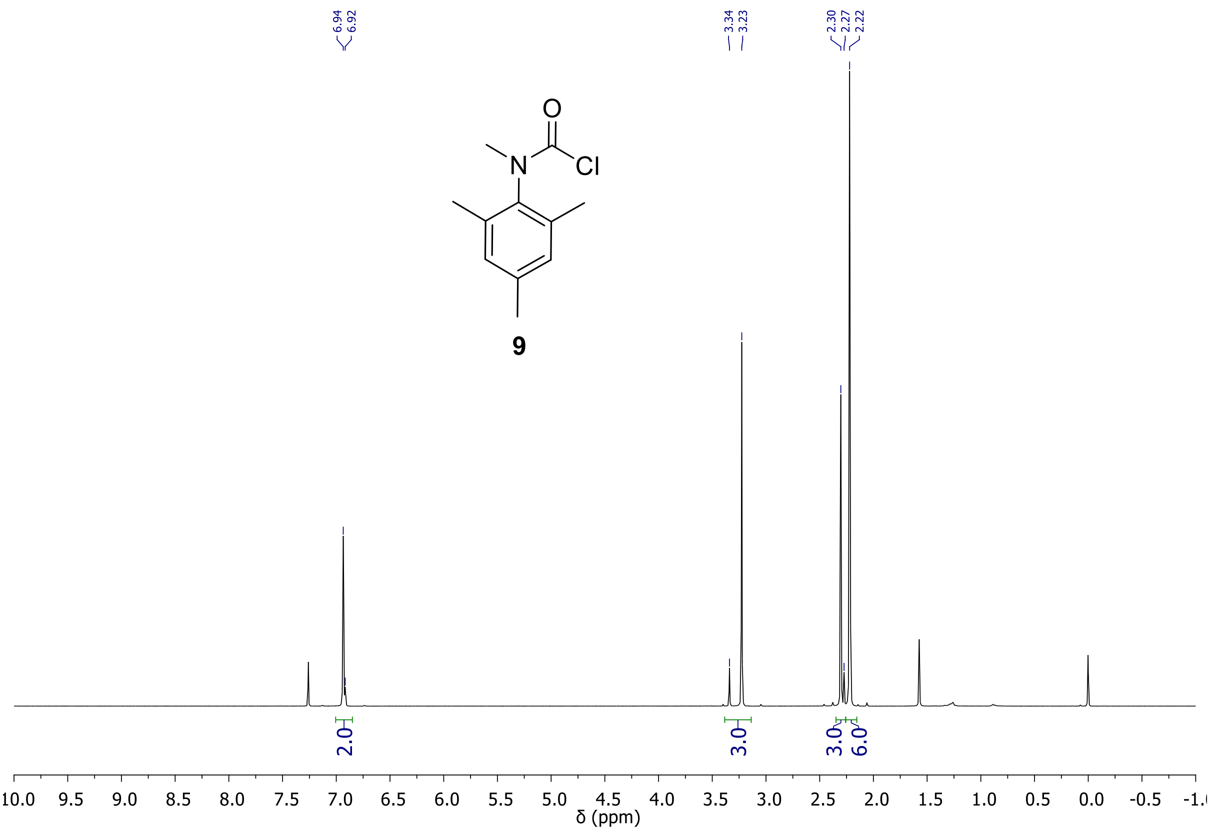

Figure S27. ^{1}H NMR spectra of **9** ($CDCl_3$, 400 MHz).

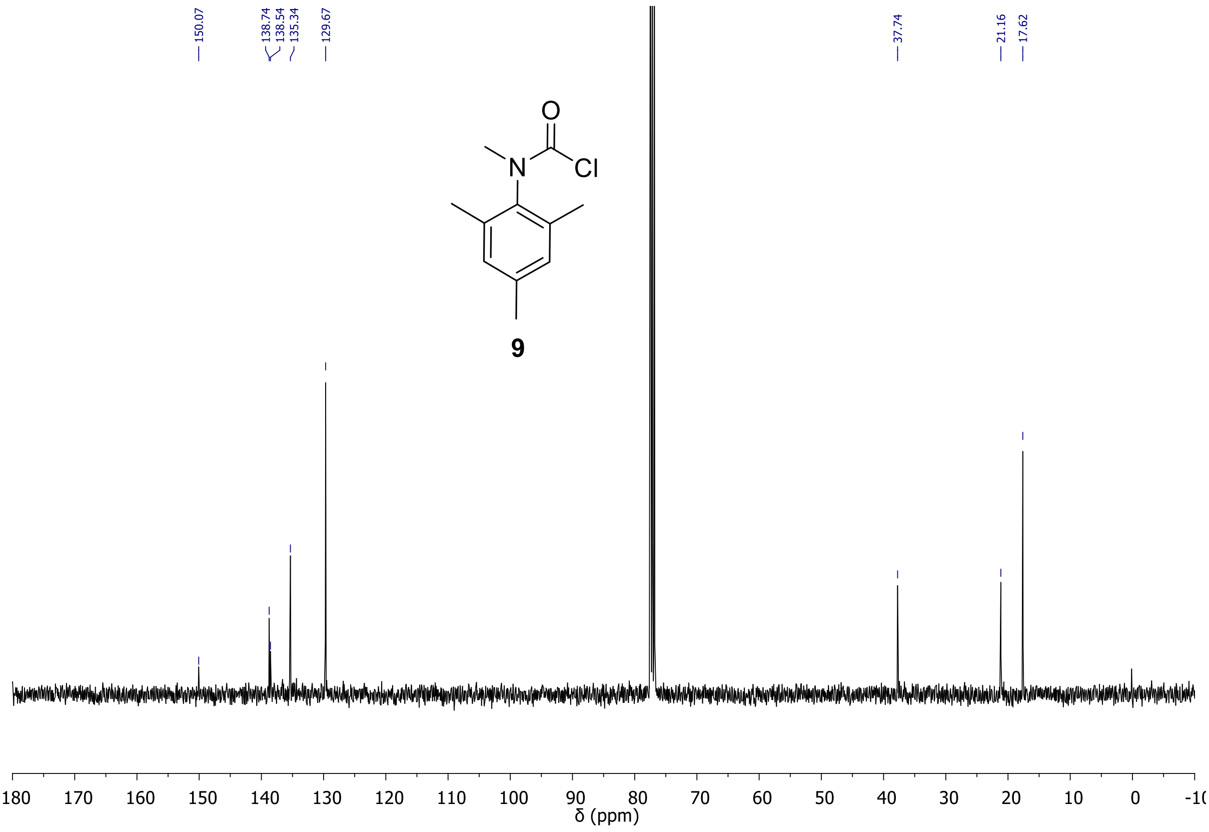

Figure S28. $^{13}C\{^{1}H\}$ NMR spectra of **9** ($CDCl_3$, 100 MHz).

NMR Characterization of self-immolative systems 15-23

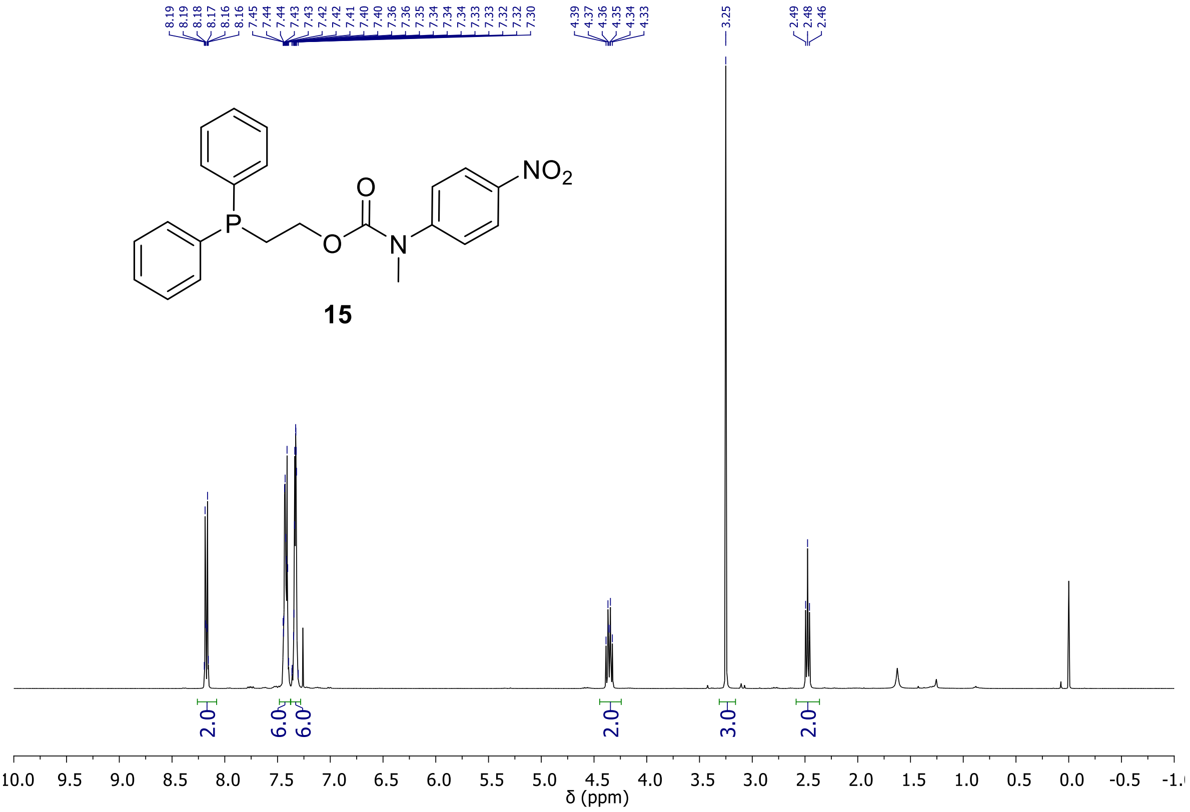

Figure S29. ^{1}H NMR spectra of **15** ($CDCl_3$, 400 MHz).

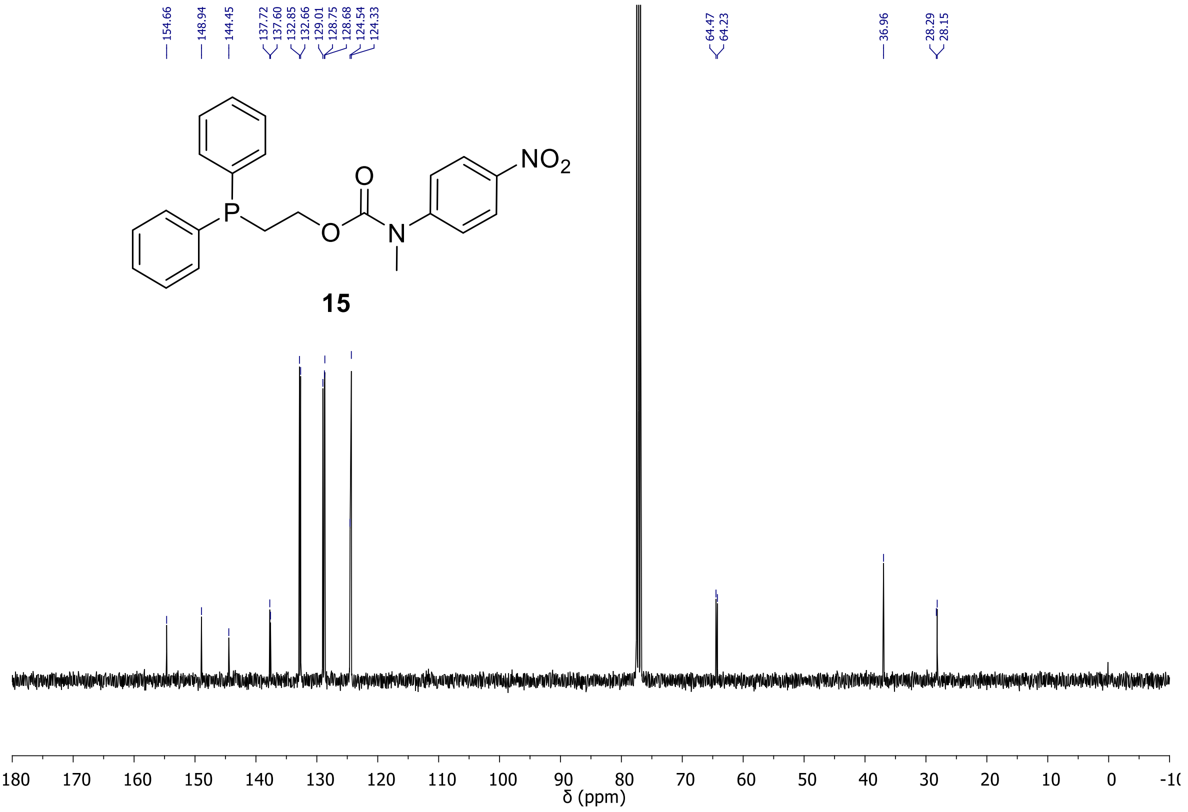

Figure S30. $^{13}C\{^{1}H\}$ NMR spectra of **15** ($CDCl_3$, 100 MHz).

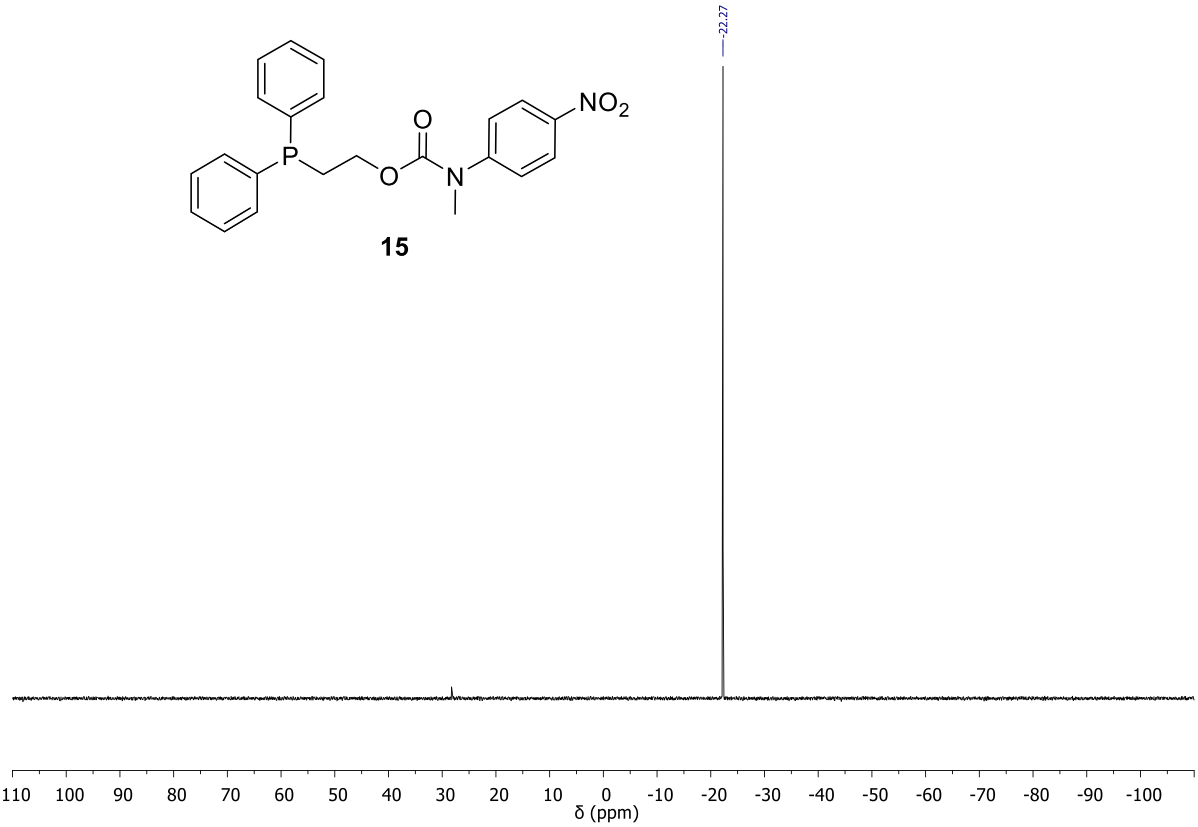

Figure S31. $^{31}P\{^{1}H\}$ NMR spectra of **15** ($CDCl_3$, 162 MHz).

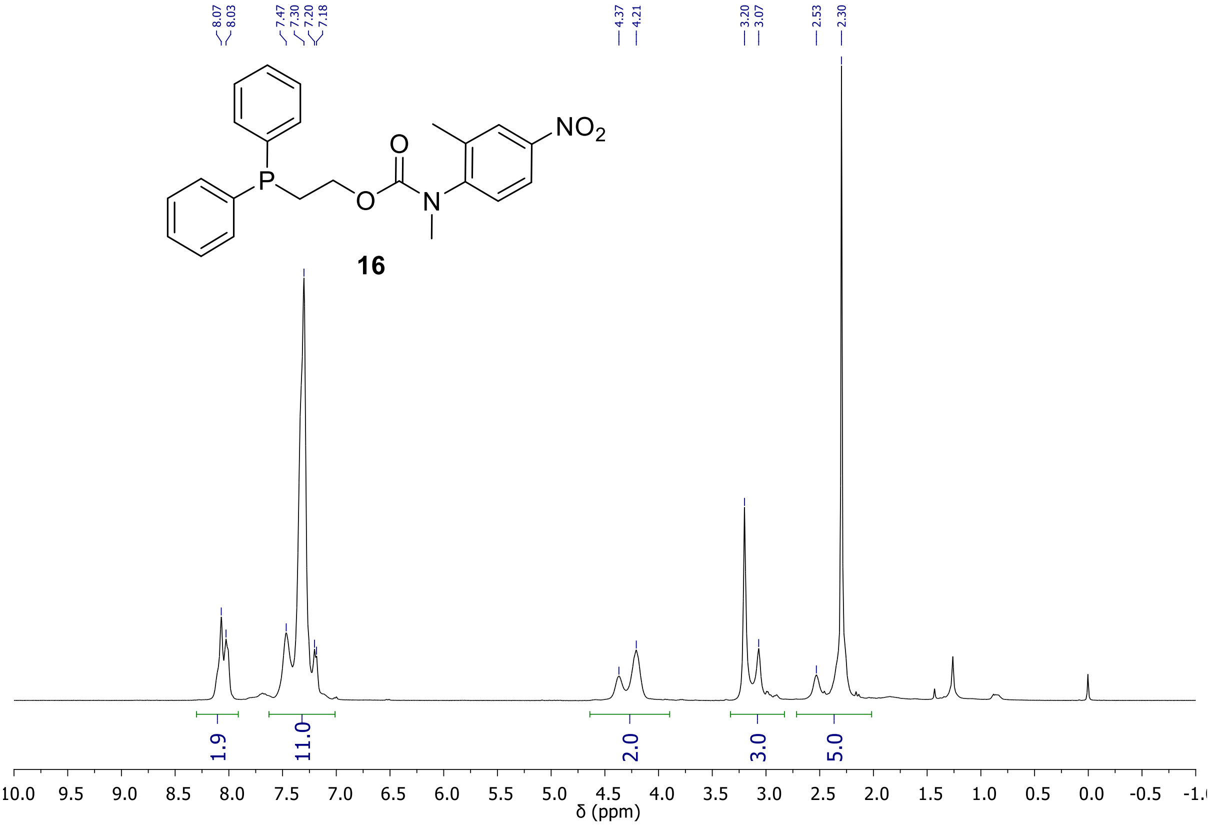

Figure S32. ^{1}H NMR spectra of **16** ($CDCl_3$, 400 MHz).

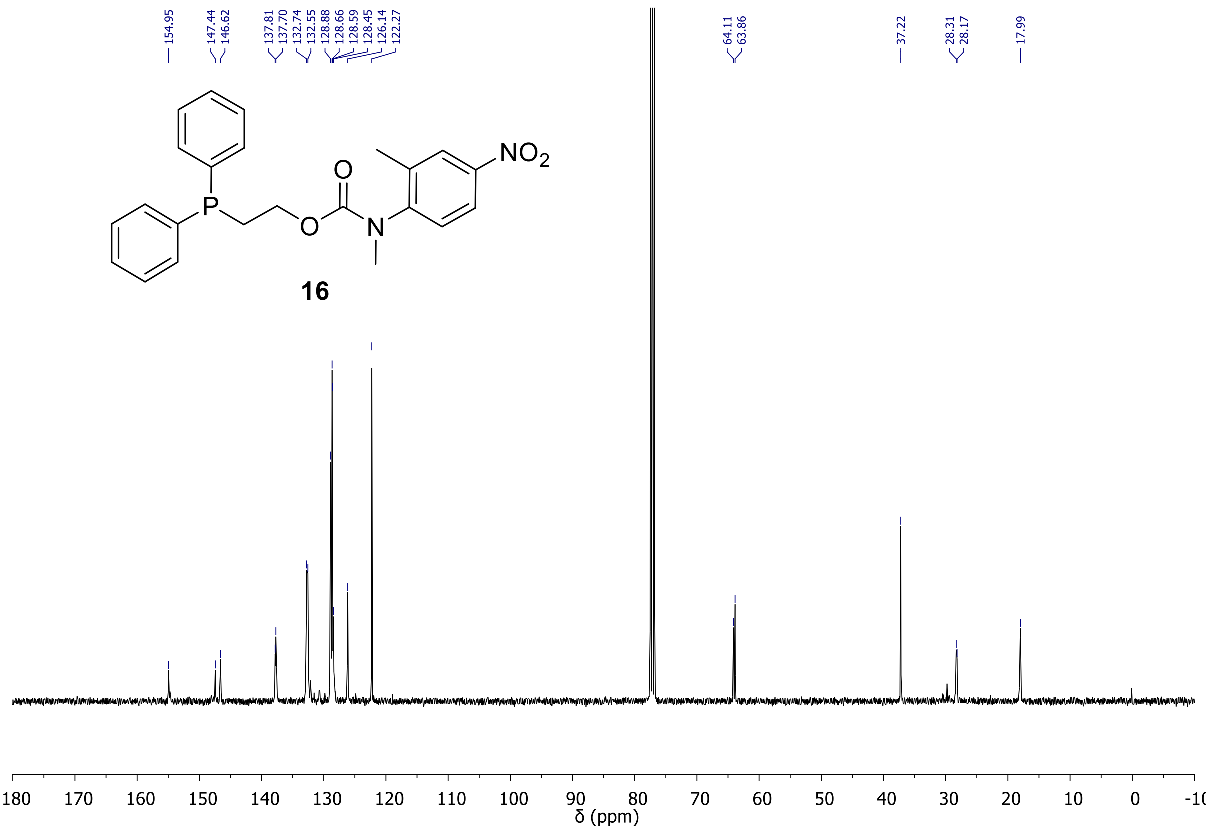

Figure S33. $^{13}C\{^{1}H\}$ NMR spectra of **16** ($CDCl_3$, 100 MHz).

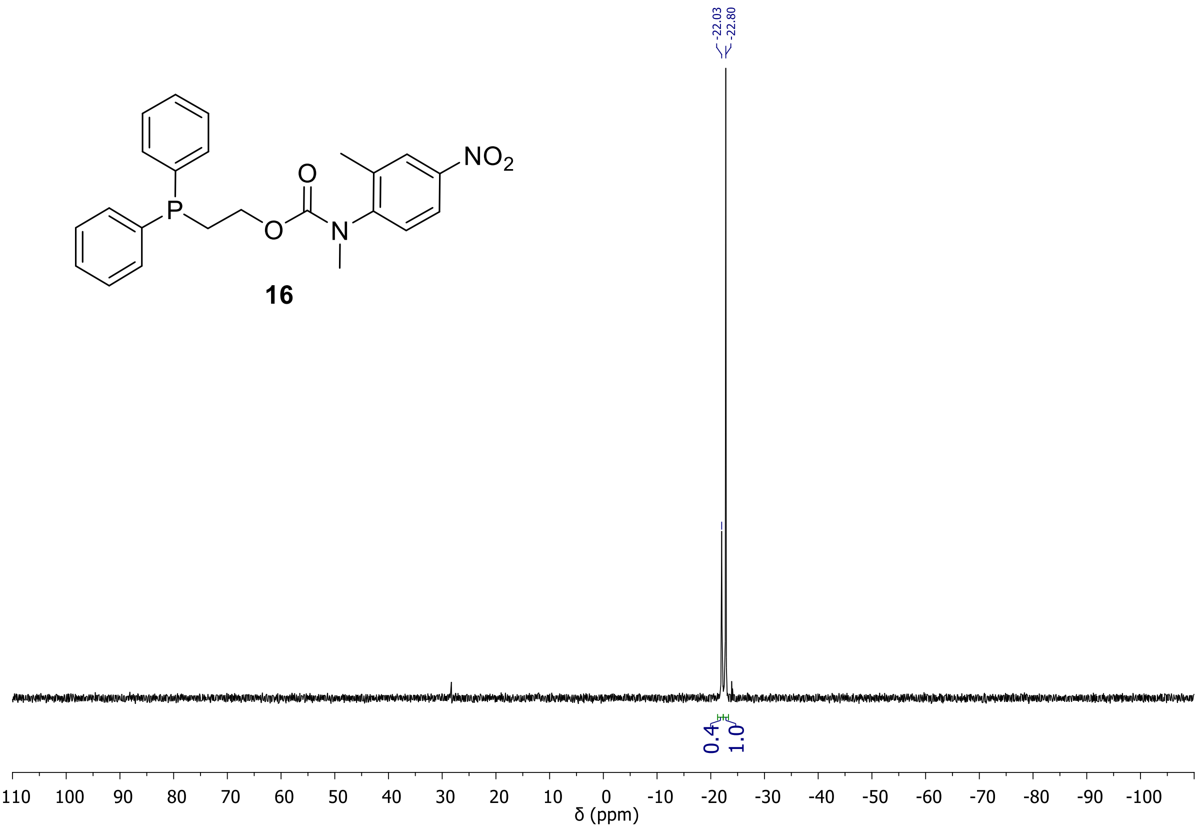

Figure S34. $^{31}P\{^{1}H\}$ NMR spectra of **16** ($CDCl_3$, 162 MHz).

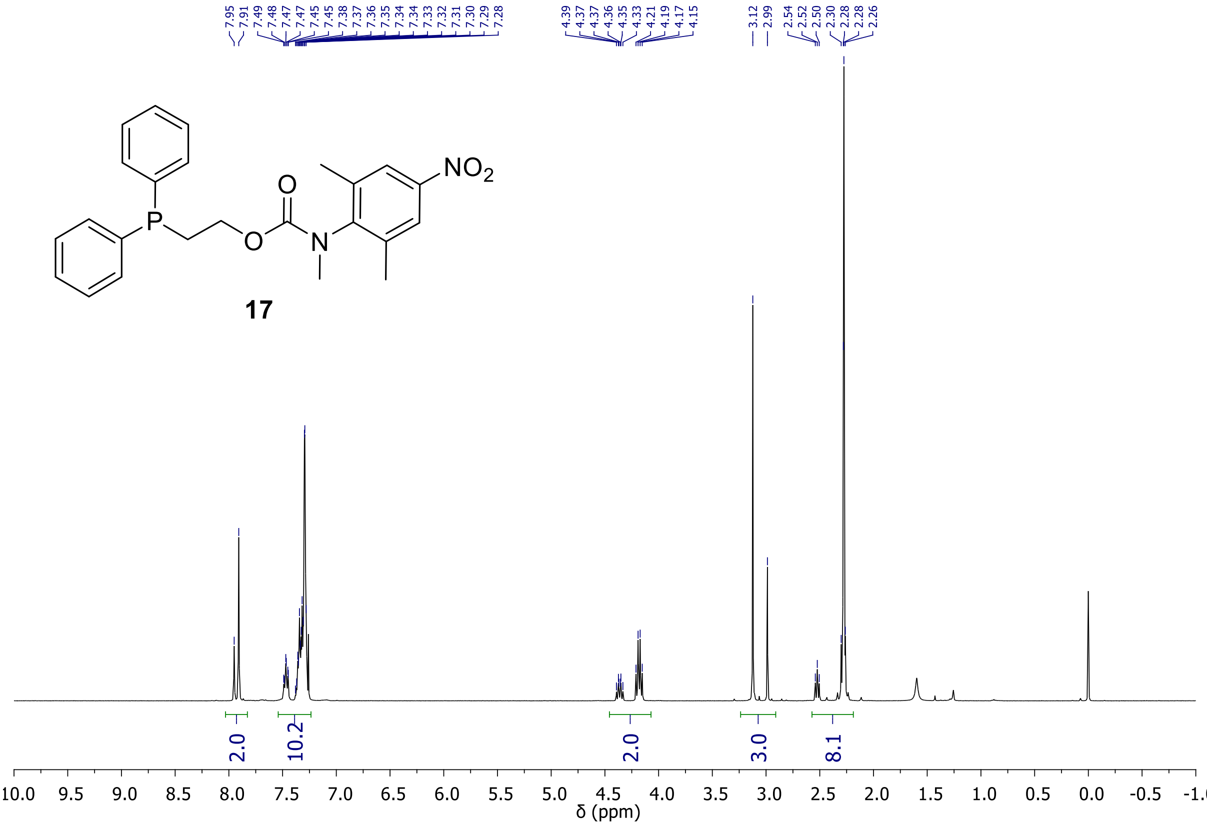

Figure S35. ^{1}H NMR spectra of **17** ($CDCl_3$, 400 MHz).

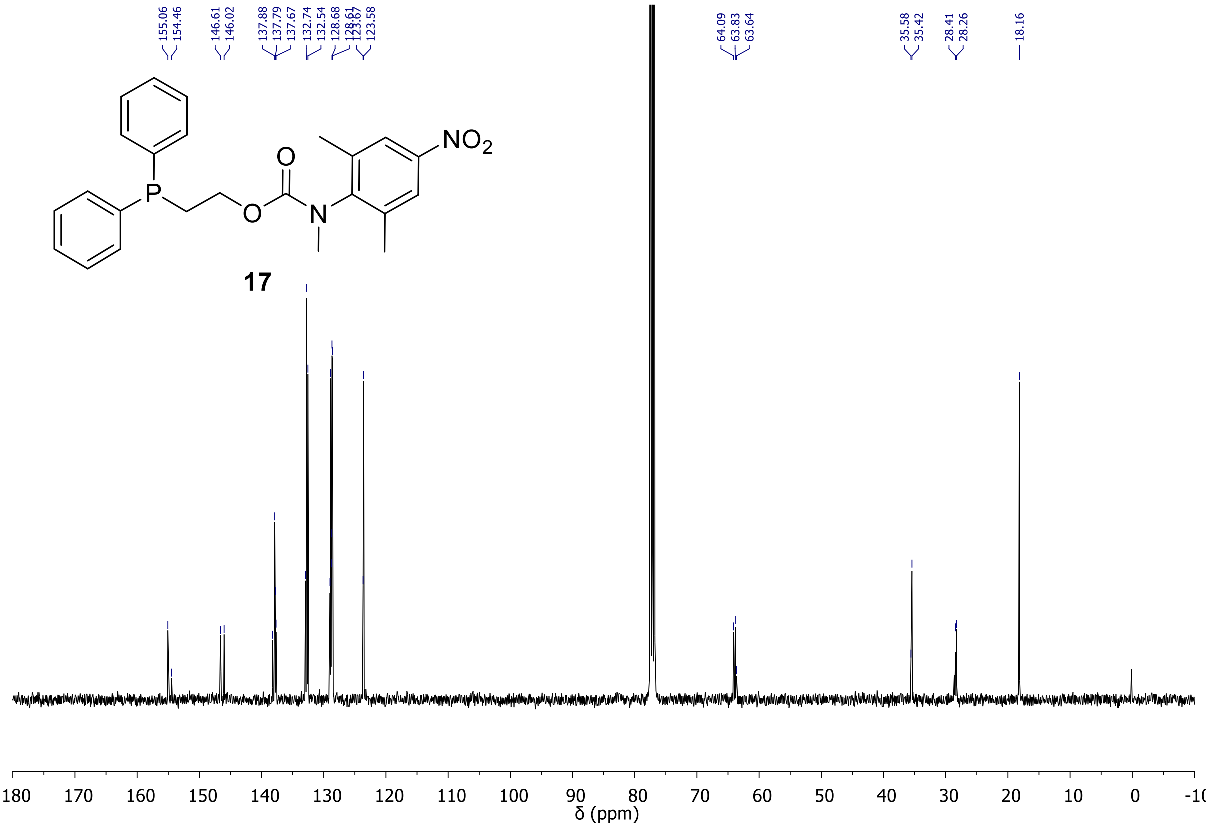

Figure S36. $^{13}C\{^{1}H\}$ NMR spectra of **17** ($CDCl_3$, 100 MHz).

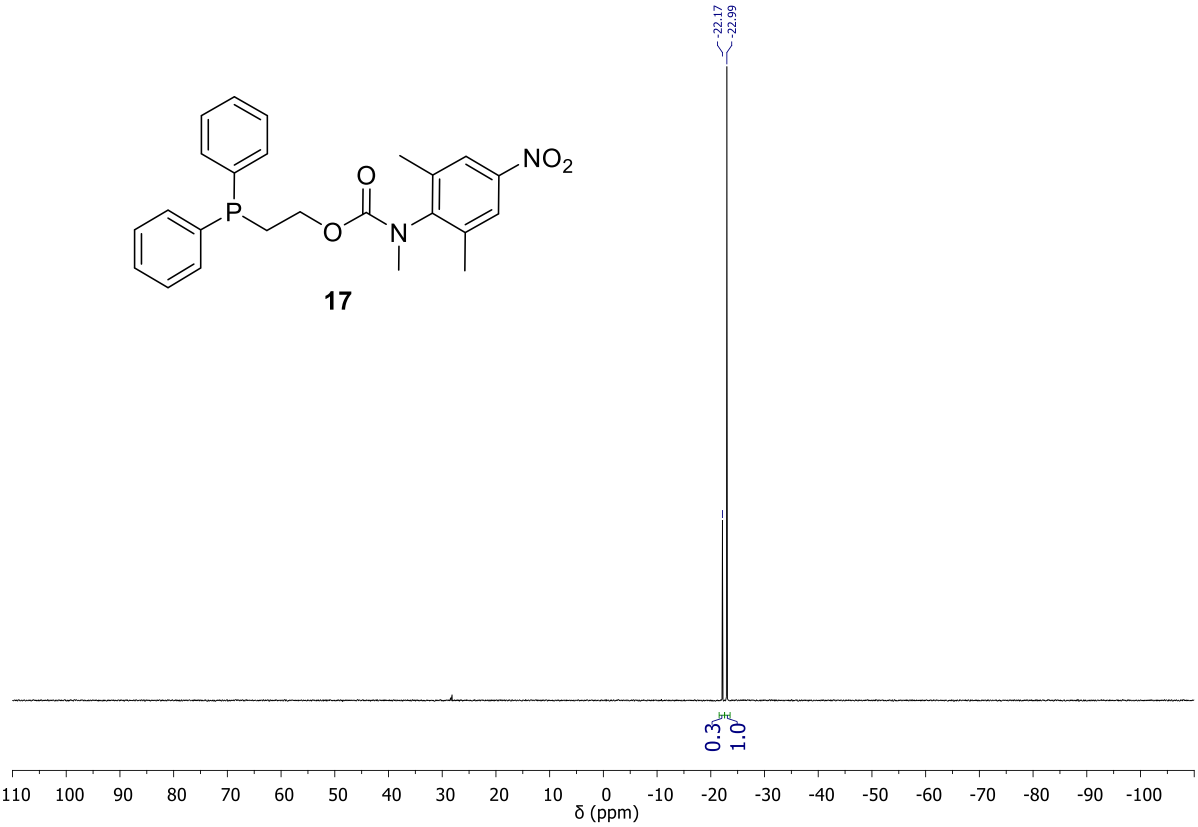

Figure S37. $^{31}P\{^{1}H\}$ NMR spectra of **17** ($CDCl_3$, 162 MHz).

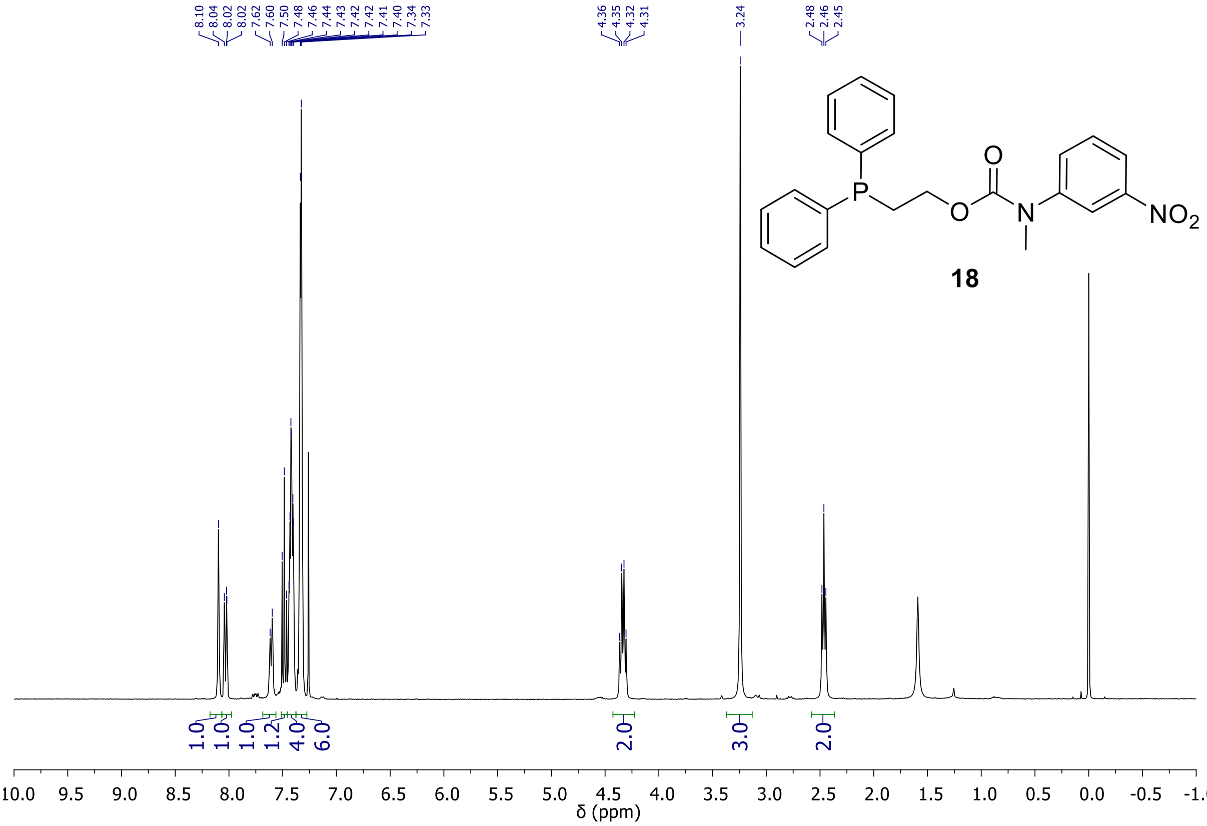

Figure S38. ^{1}H NMR spectra of **18** ($CDCl_3$, 400 MHz).

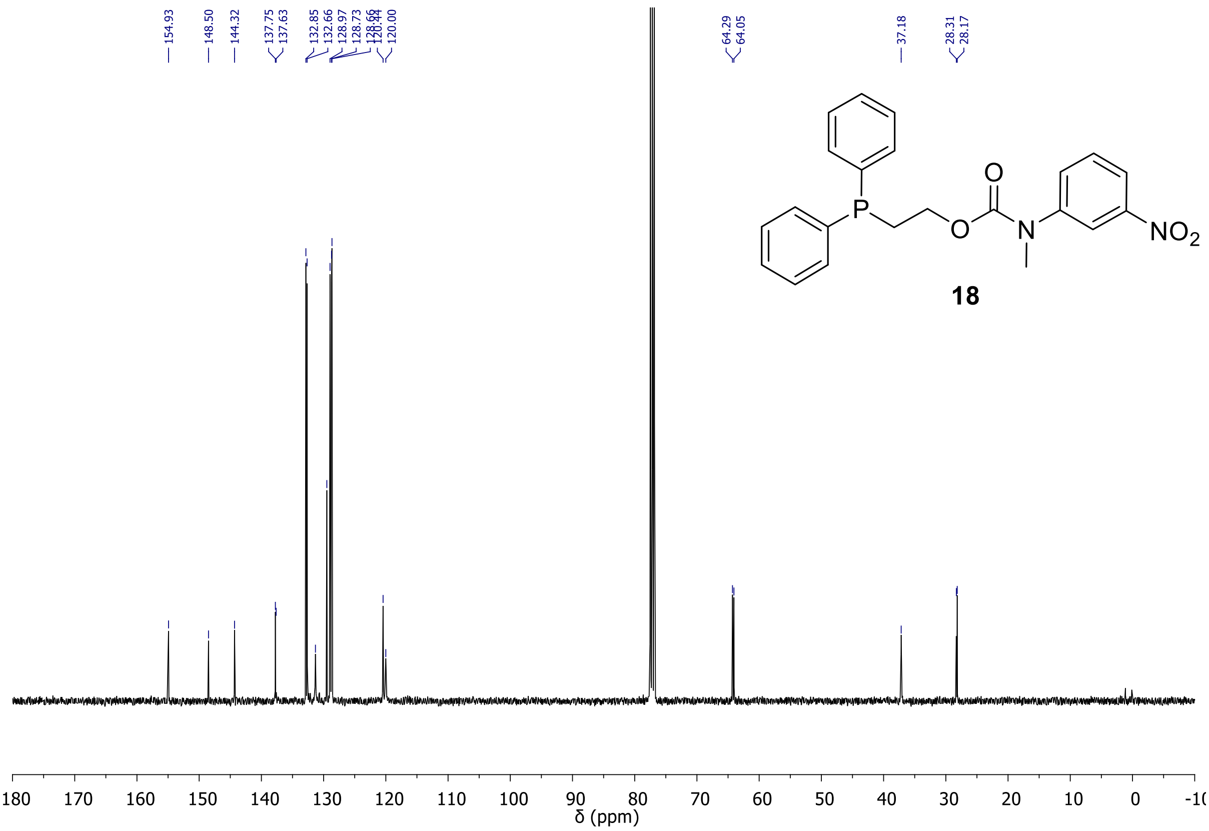

Figure S39. $^{13}C\{^{1}H\}$ NMR spectra of **18** ($CDCl_3$, 100 MHz).

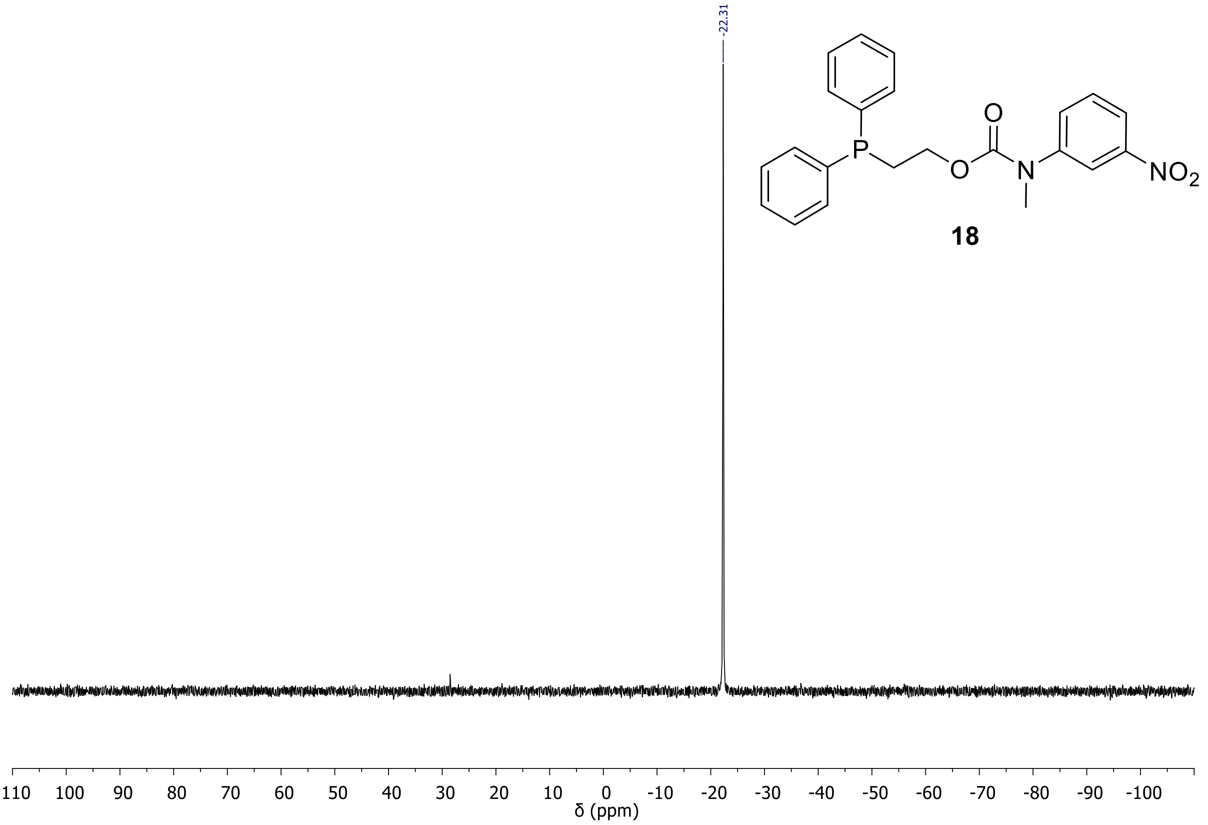

Figure S40. $^{31}P\{^{1}H\}$ NMR spectra of **18** ($CDCl_3$, 162 MHz).

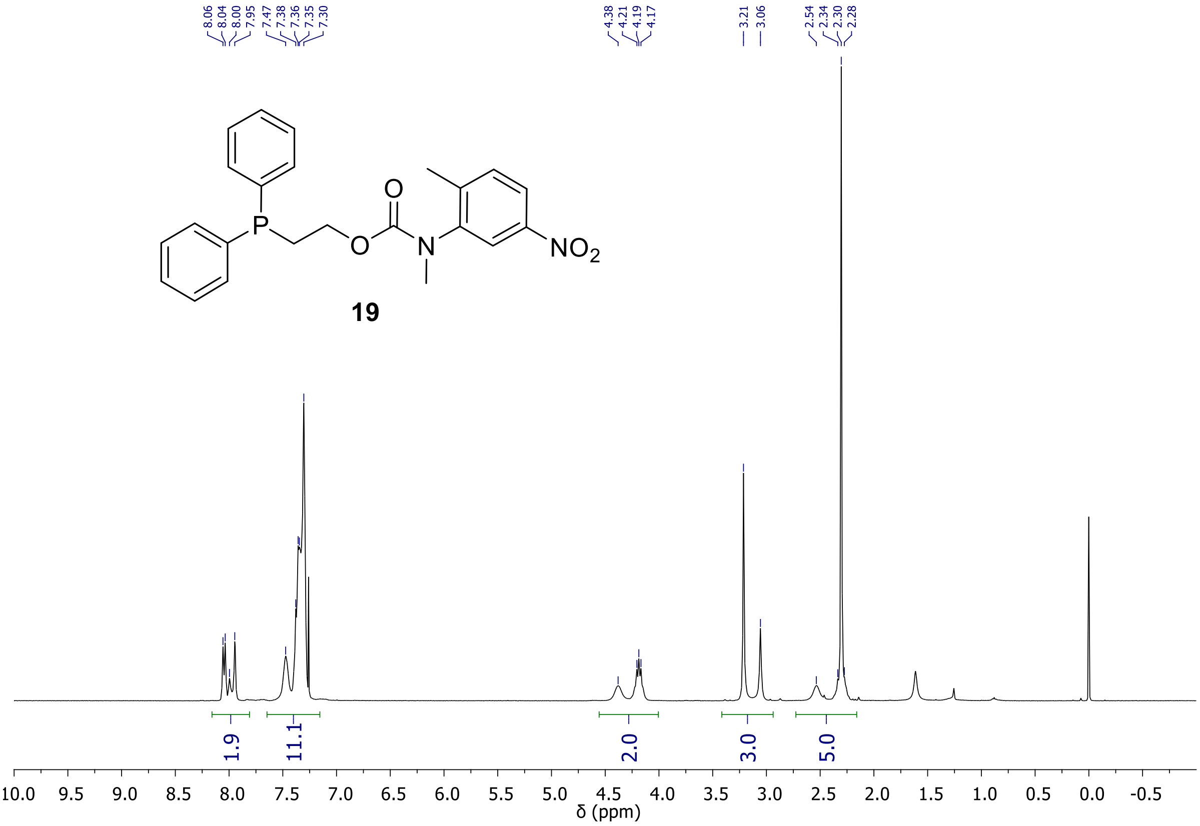

Figure S41. ^{1}H NMR spectra of **19** ($CDCl_3$, 400 MHz).

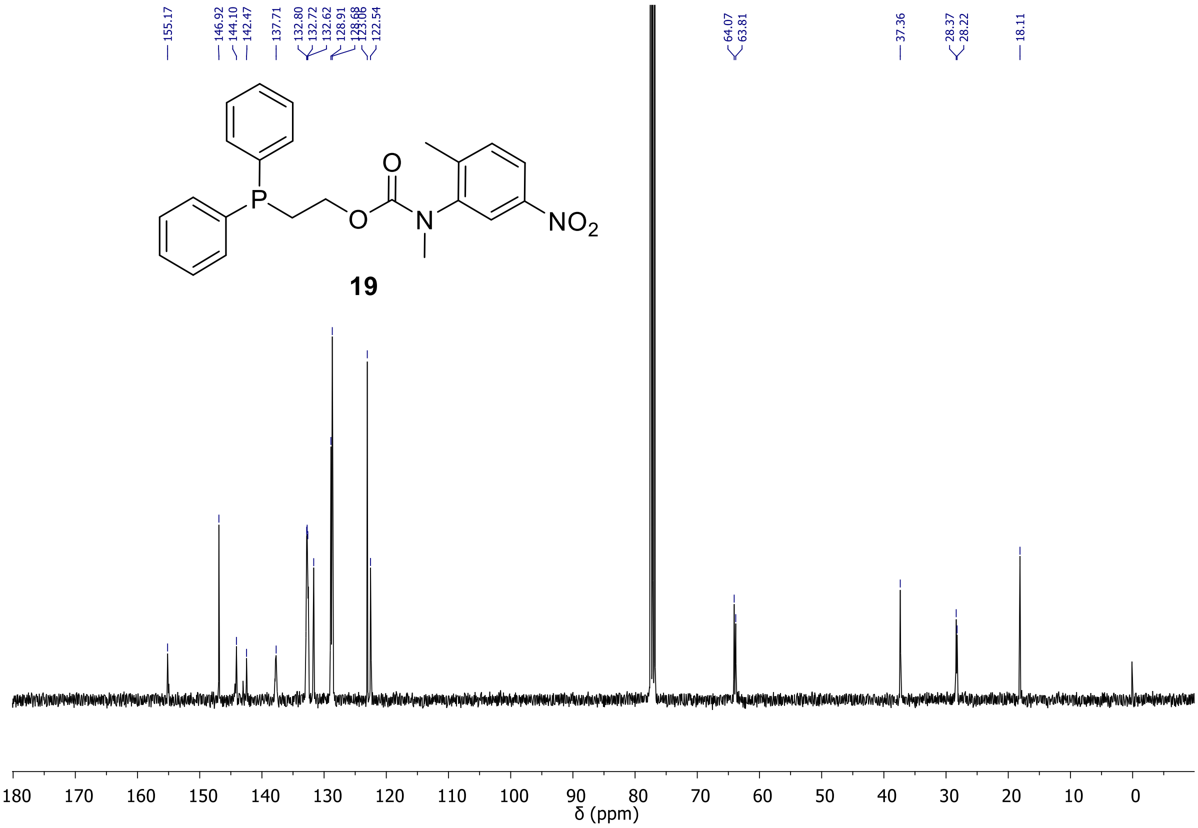

Figure S42. $^{13}C\{^{1}H\}$ NMR spectra of **19** ($CDCl_3$, 100 MHz).

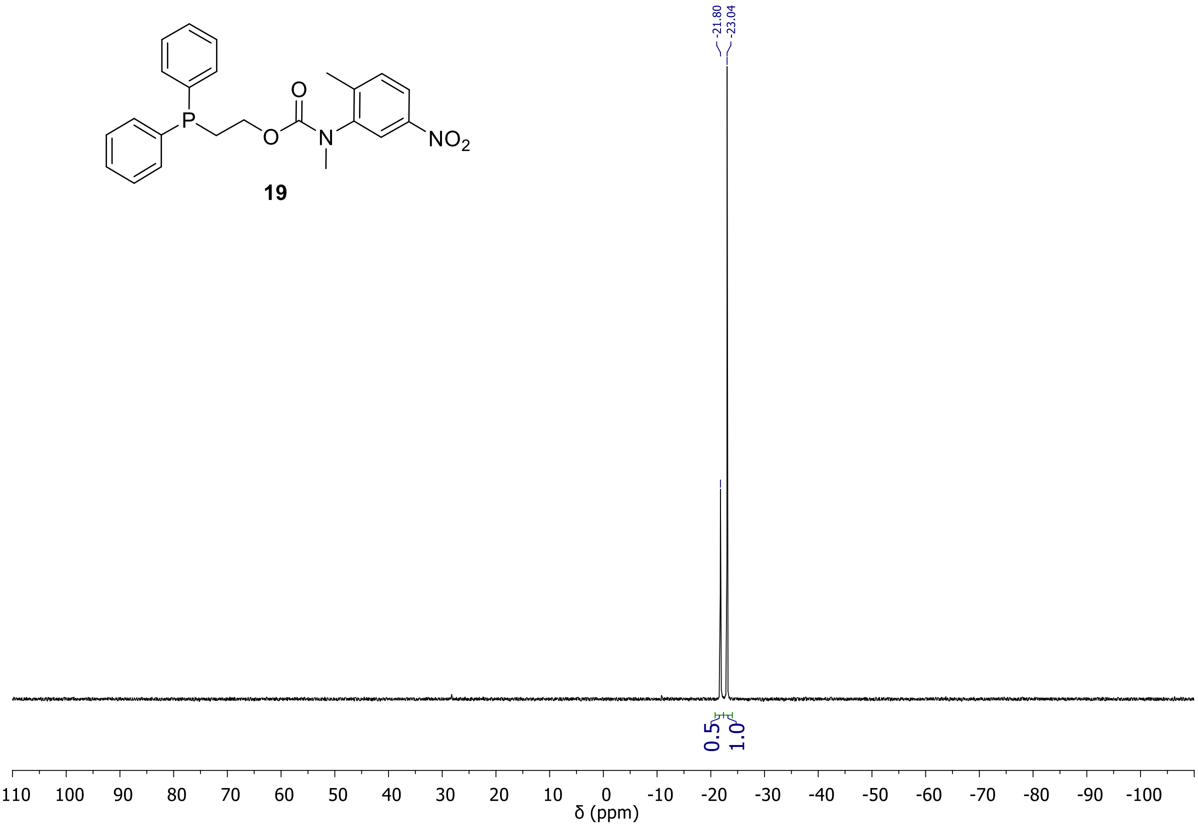

Figure S43. $^{31}P\{^1H\}$ NMR spectra of **19** ($CDCl_3$, 162 MHz).

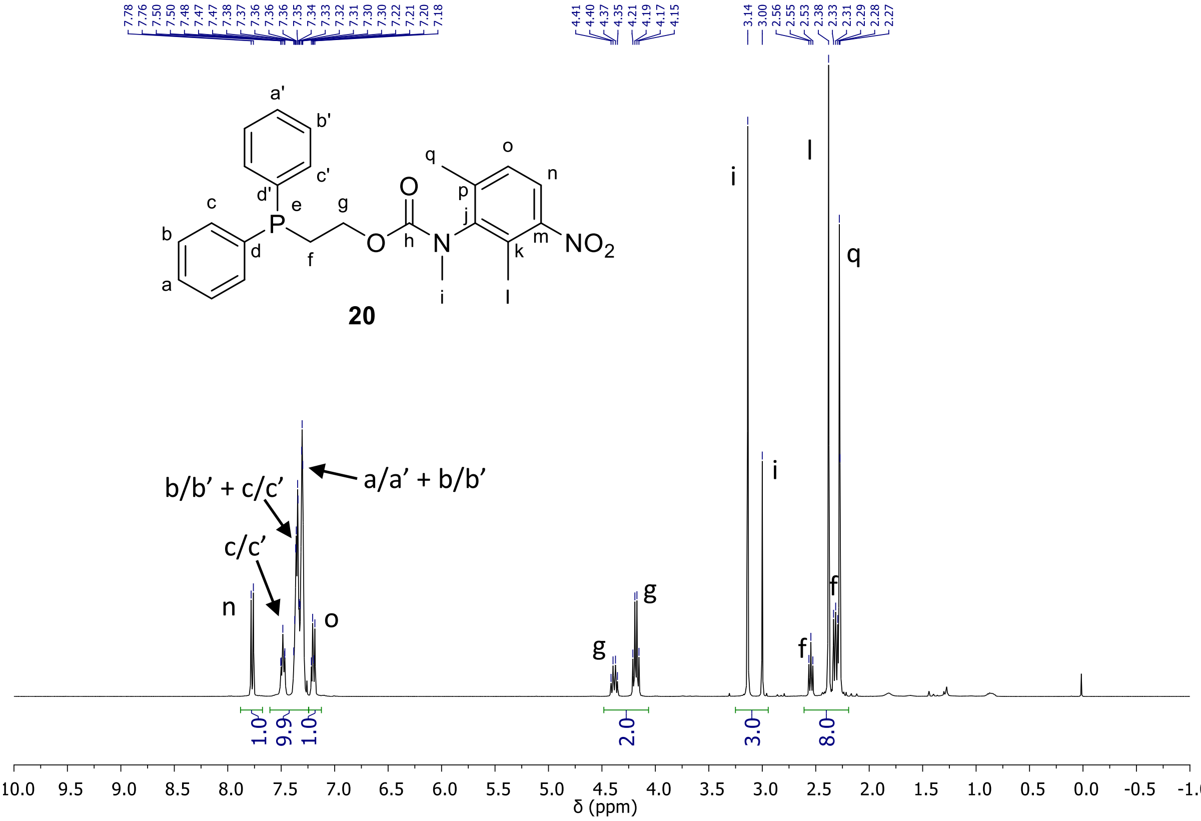

Figure S44. ^1H NMR spectra of **20** ($CDCl_3$, 400 MHz).

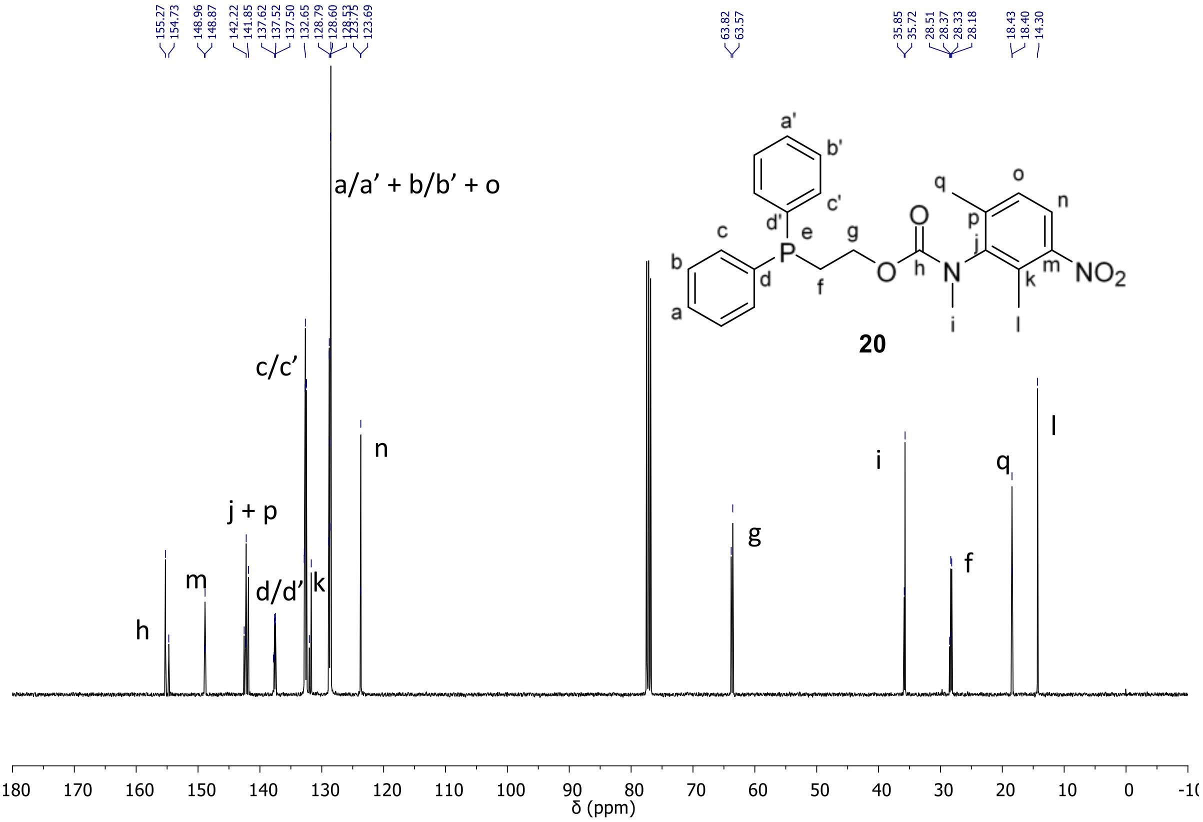

Figure S45. $^{13}C\{^{1}H\}$ NMR spectra of **20** ($CDCl_3$, 100 MHz).

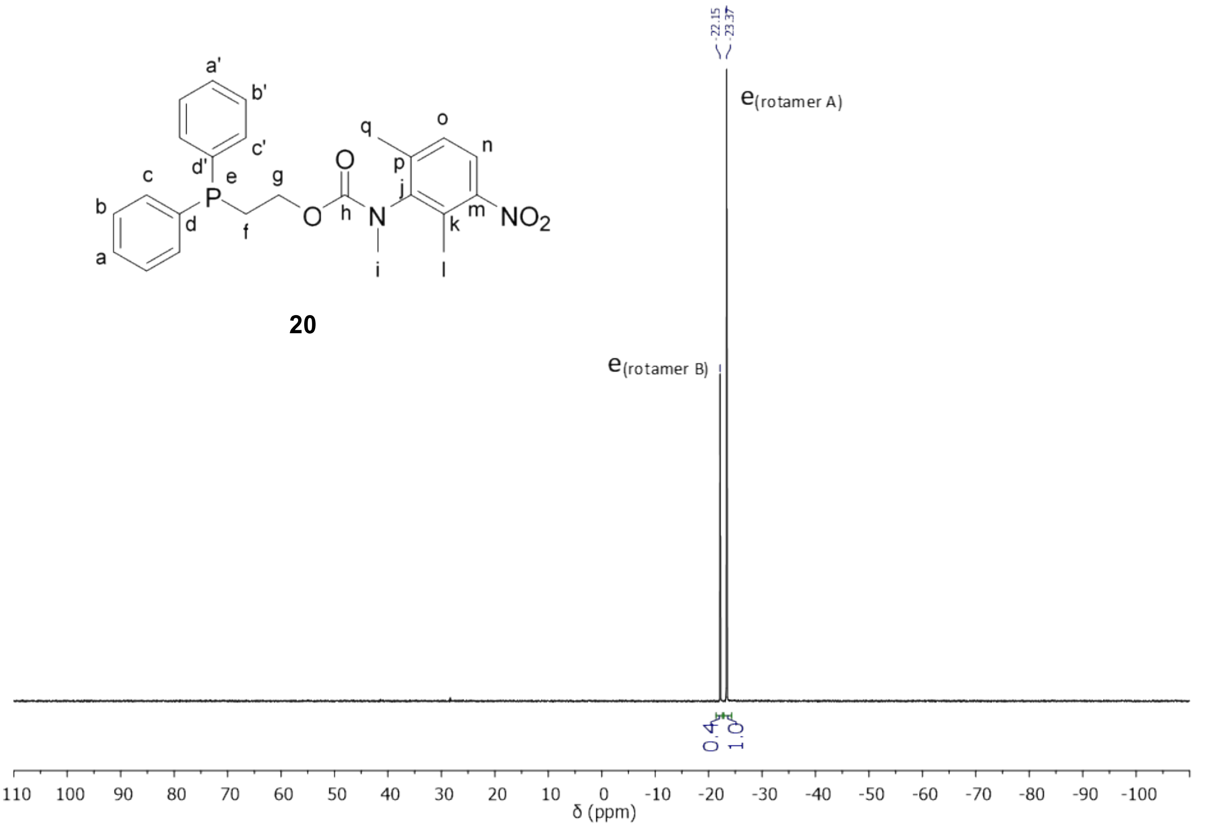

Figure S46. $^{31}P\{^{1}H\}$ NMR spectra of **20** ($CDCl_3$, 162 MHz).

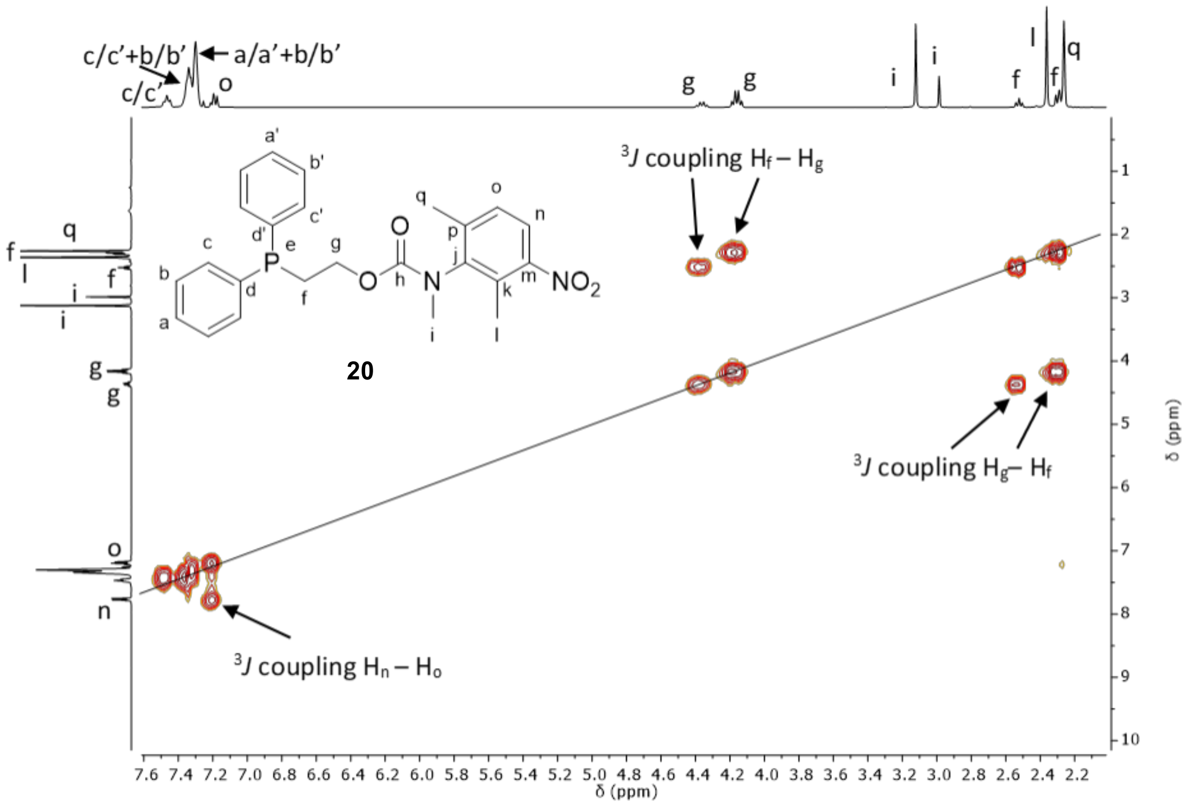

Figure S47. Partial COSY (^1H-^1H) NMR spectra of **20** ($CDCl_3$, 400 MHz).

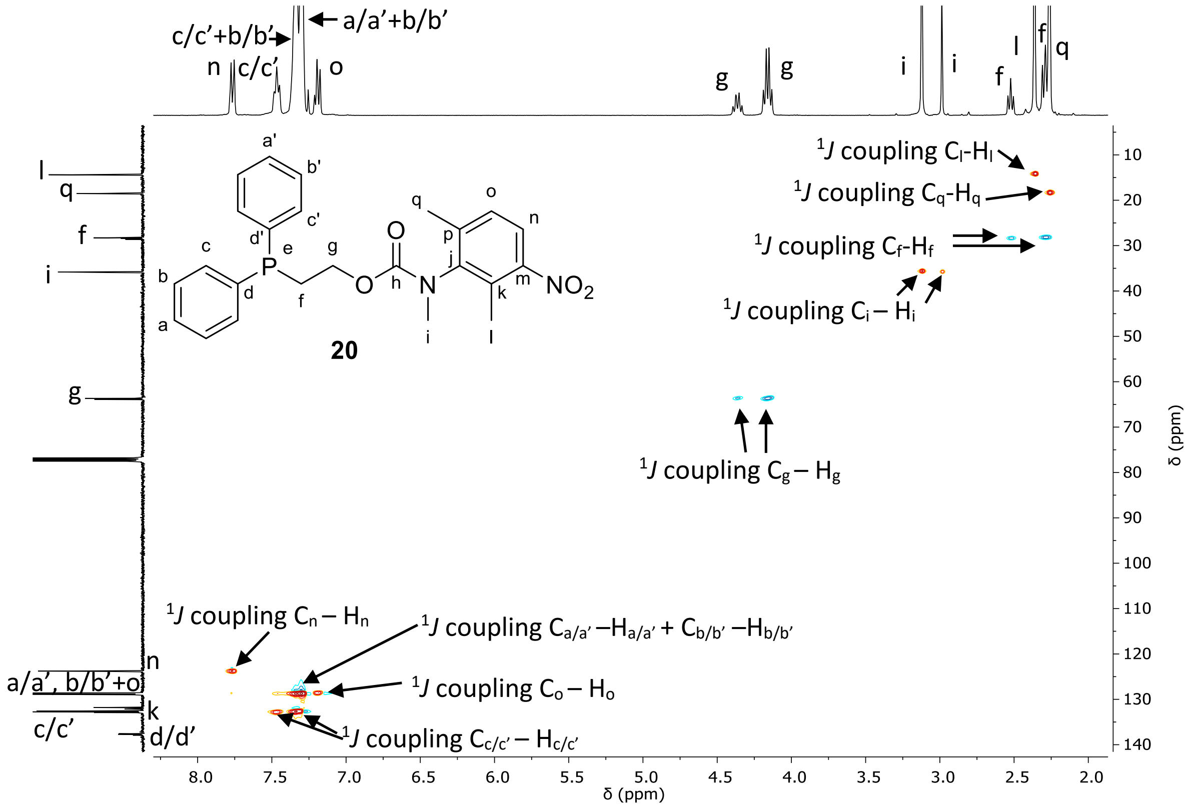

Figure S48. Partial HSQC (^1H-$^{13}C\{^1H\}$) NMR spectra of **20** ($CDCl_3$).

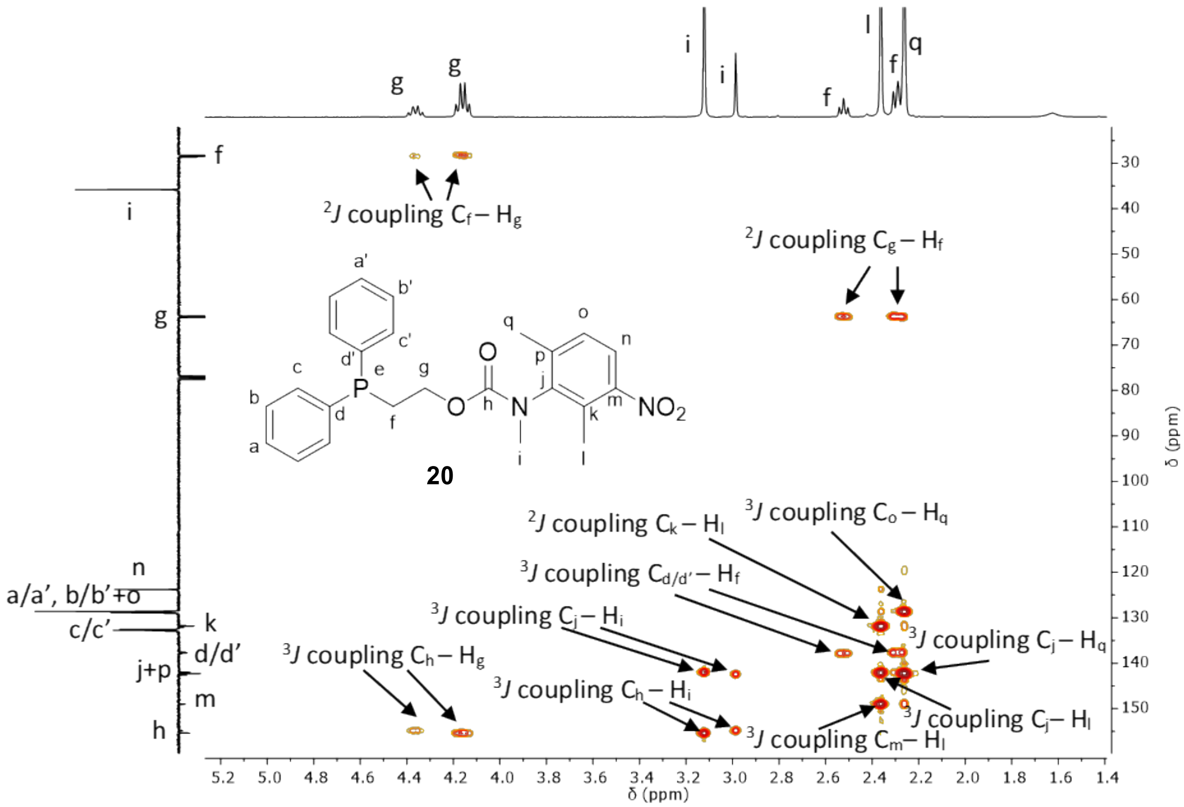

Figure S49. Partial HMBC (^1H-$^{13}C\{^1H\}$) NMR spectra of **20** ($CDCl_3$).

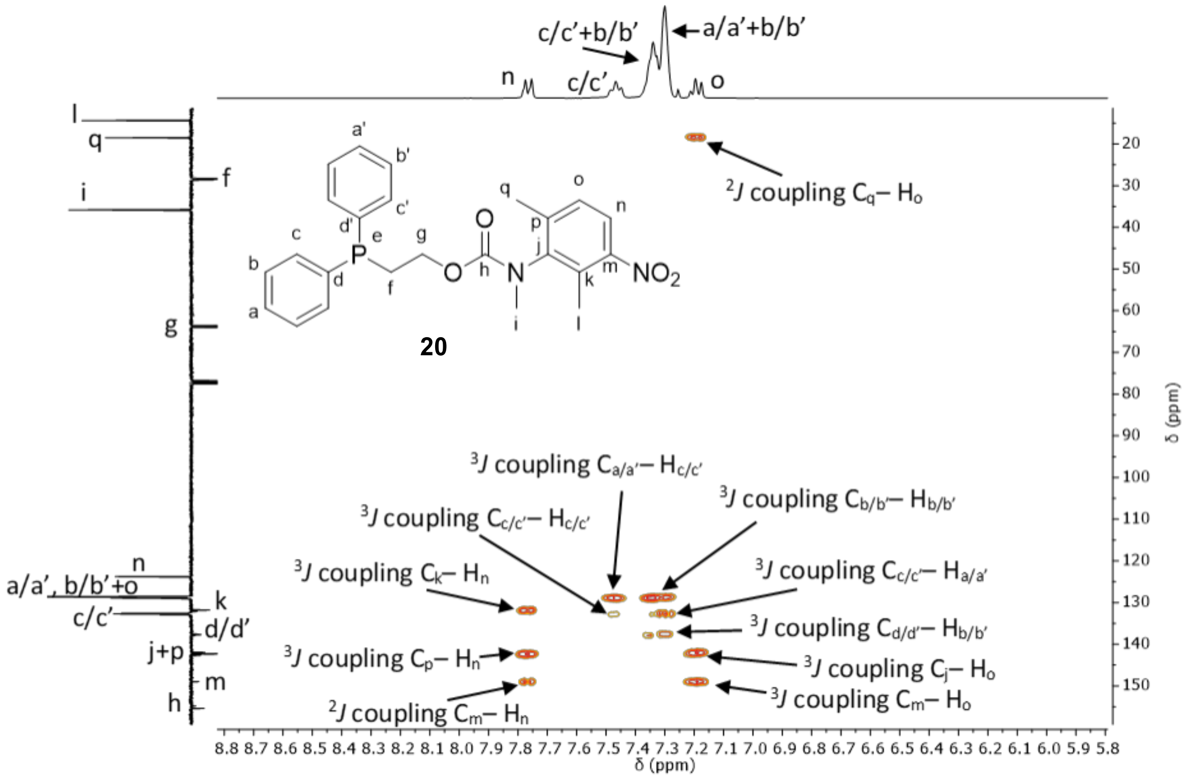

Figure S50. Partial HMBC (^1H-$^{13}C\{^1H\}$) NMR spectra of **20** ($CDCl_3$).

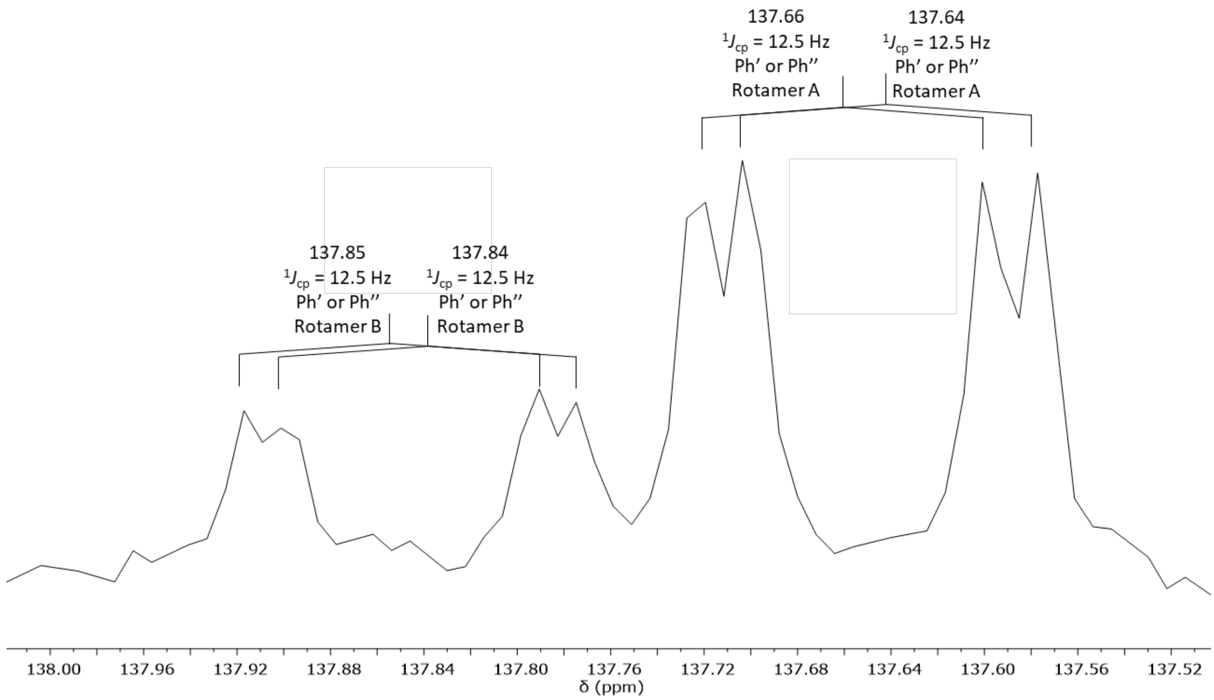

Figure S51. Enlargement of the $^{13}C\{^1H\}$ NMR spectra of **20** in the area of 137.50-138.02 ppm.

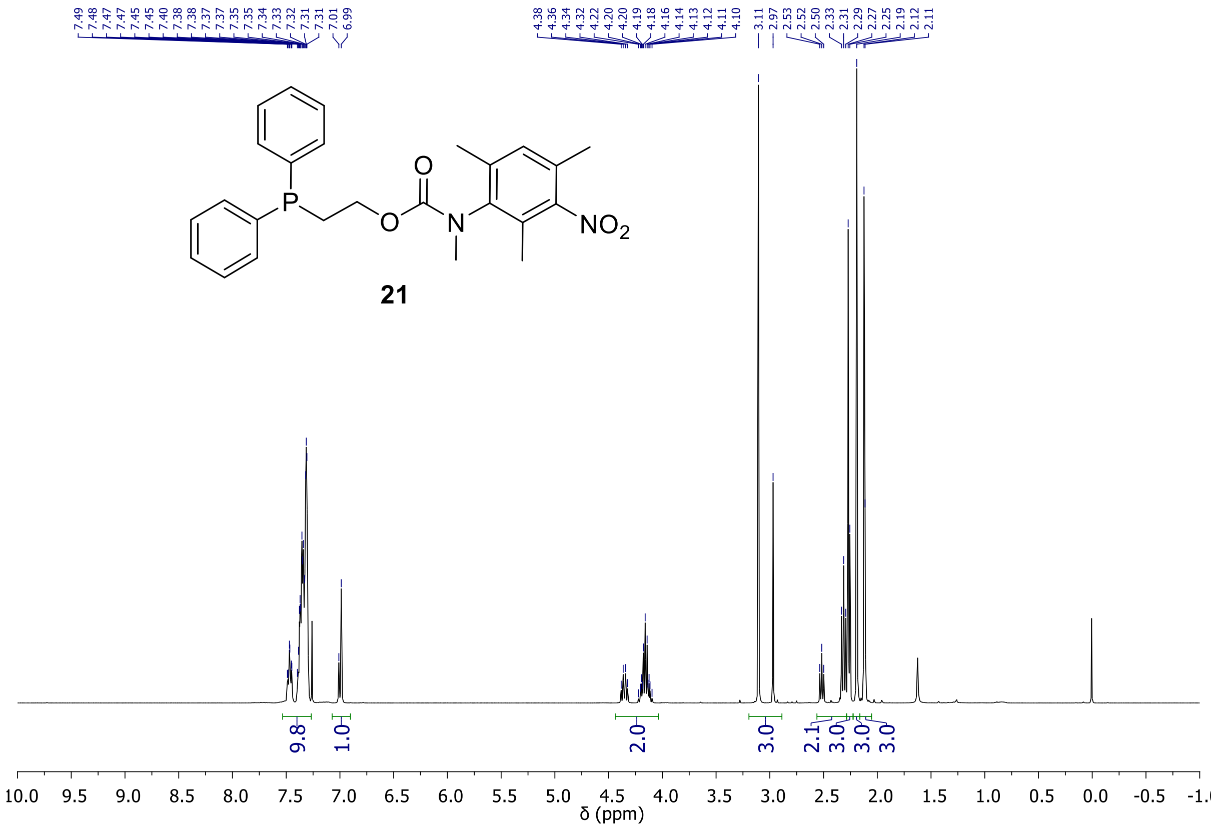

Figure S52. ^1H NMR spectra of **21** ($CDCl_3$, 400 MHz).

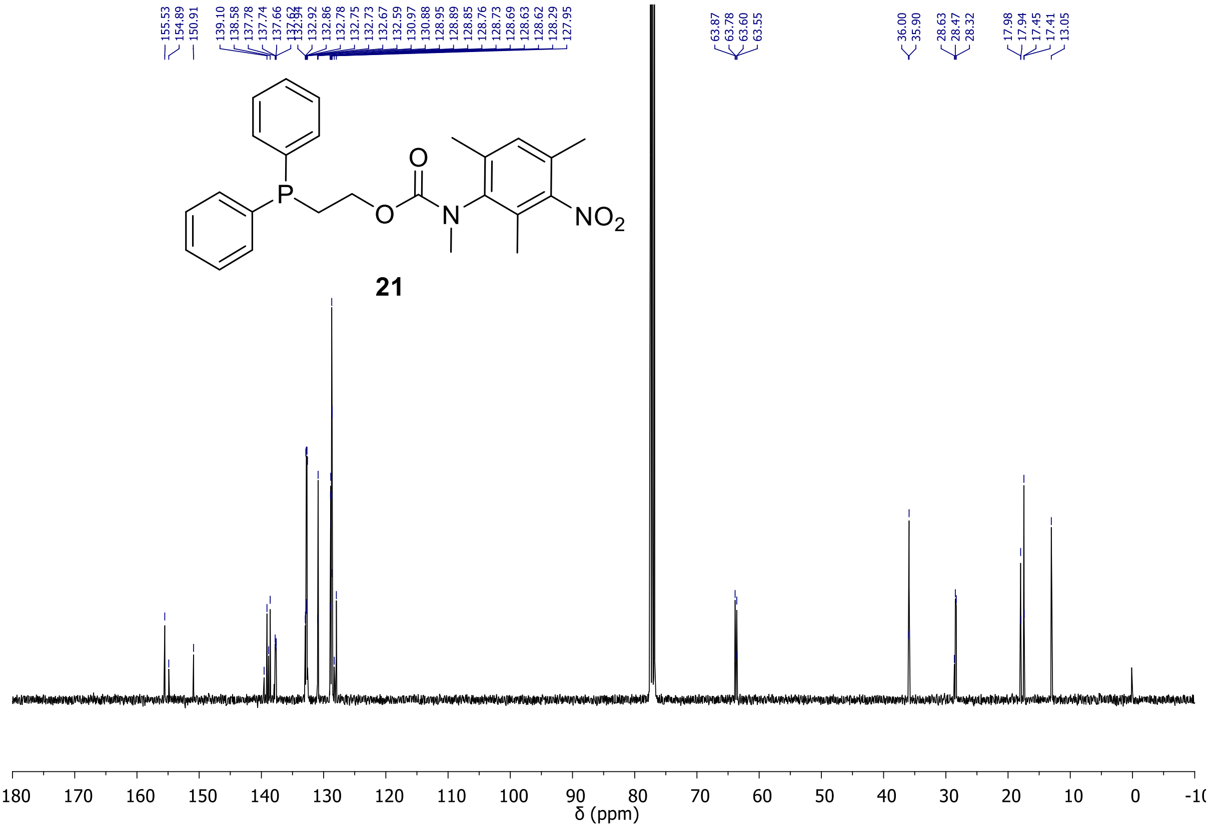

Figure S53. $^{13}C\{^{1}H\}$ NMR spectra of **21** ($CDCl_3$, 100 MHz).

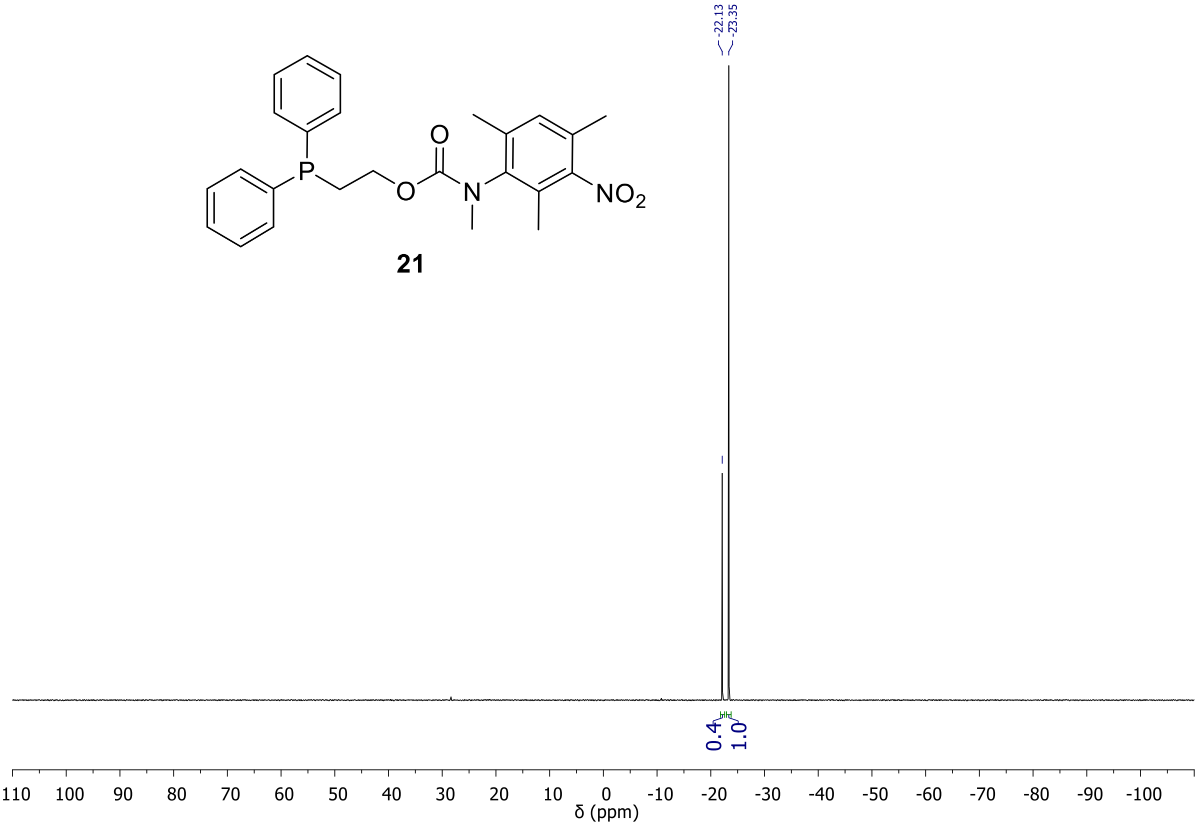

Figure S54. $^{31}P\{^{1}H\}$ NMR spectra of **21** ($CDCl_3$, 162 MHz).

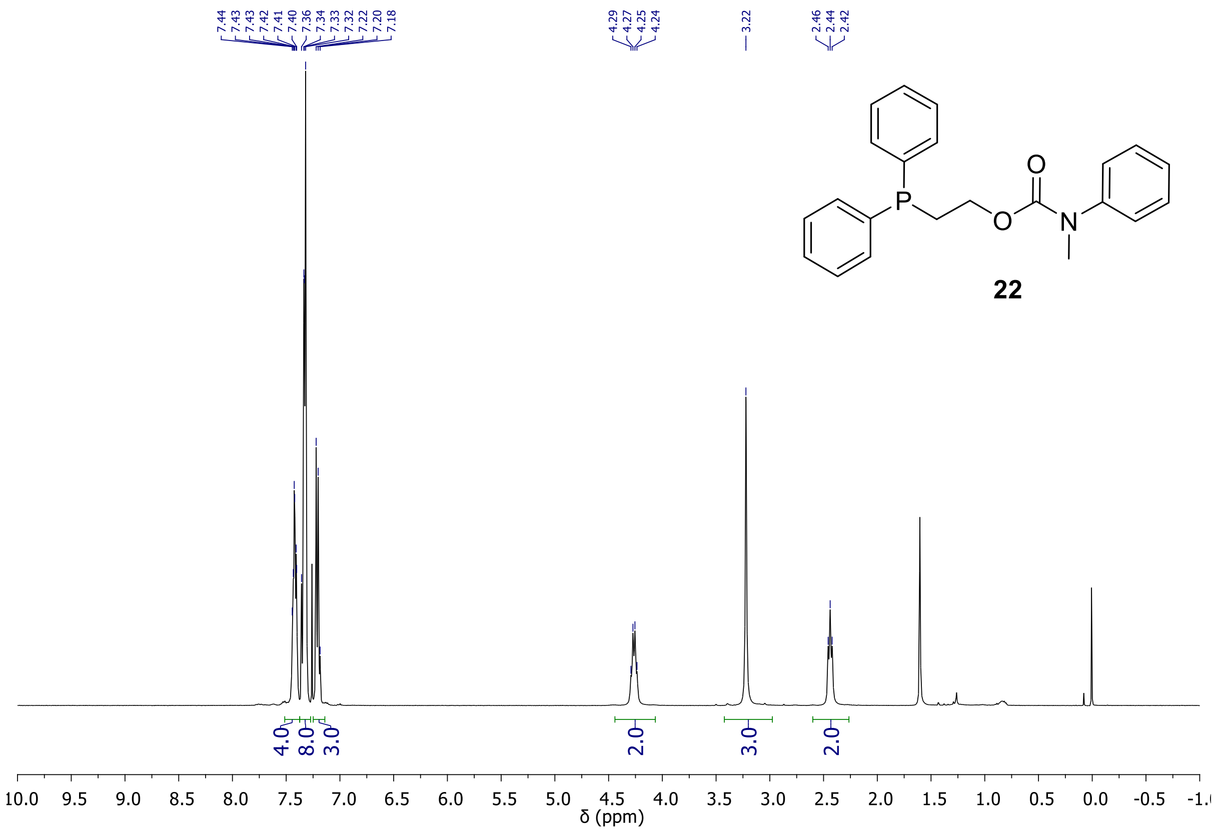

Figure S55. ^{1}H NMR spectra of **22** ($CDCl_3$, 400 MHz).

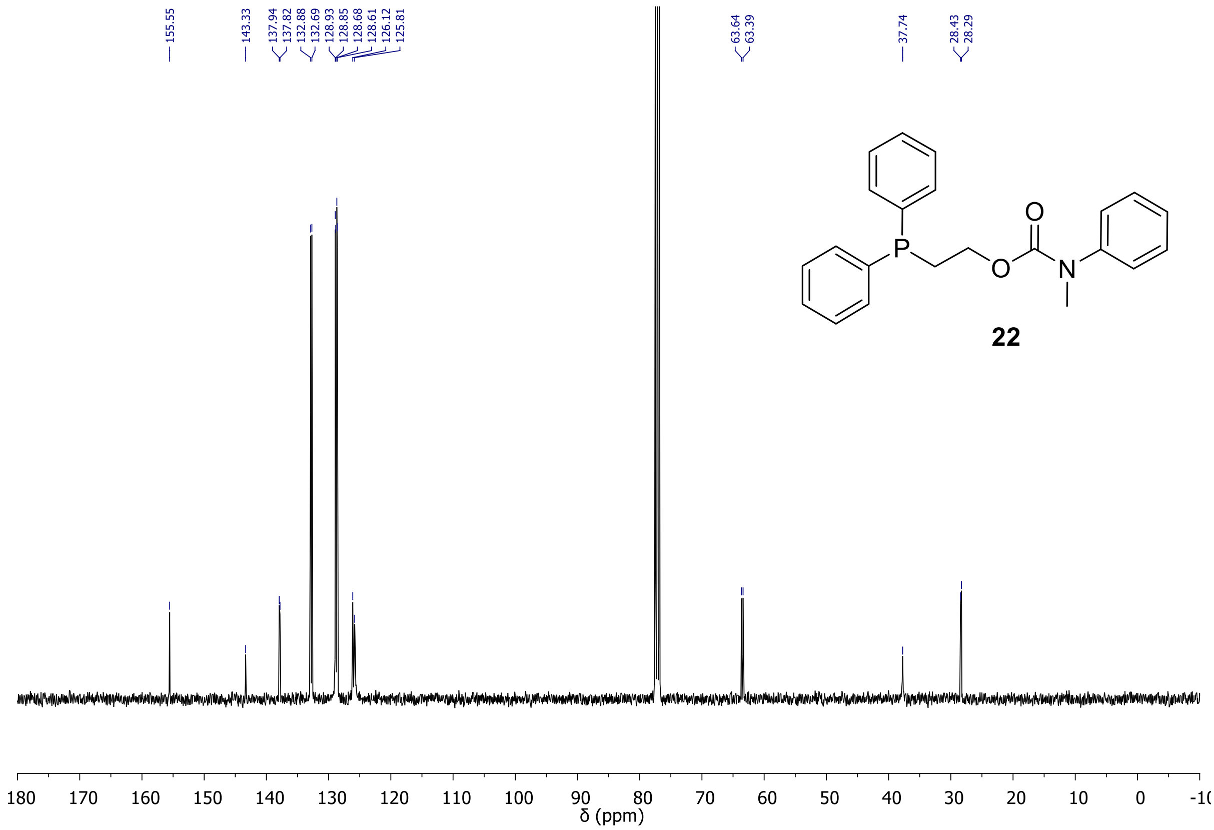

Figure S56. $^{13}C\{^{1}H\}$ NMR spectra of **22** ($CDCl_3$, 100 MHz).

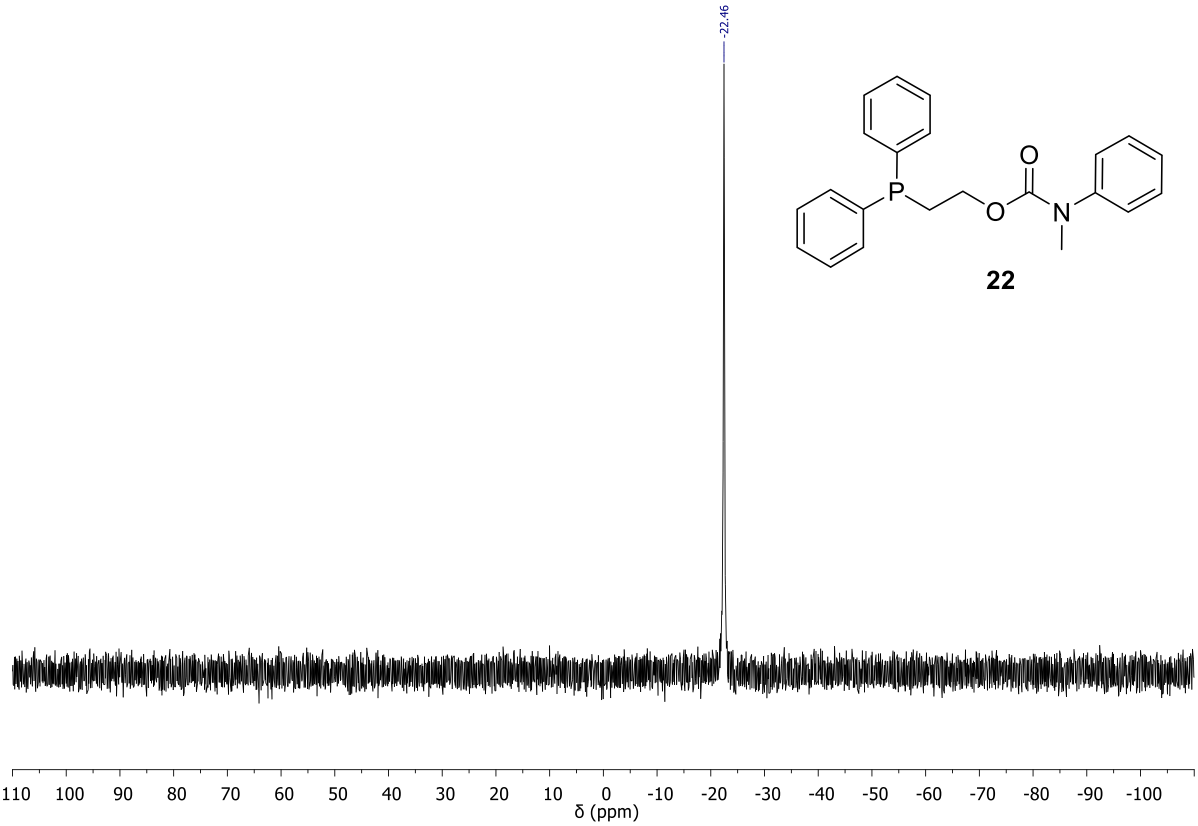

Figure S57. $^{31}P\{^{1}H\}$ NMR spectra of **22** ($CDCl_3$, 162 MHz).

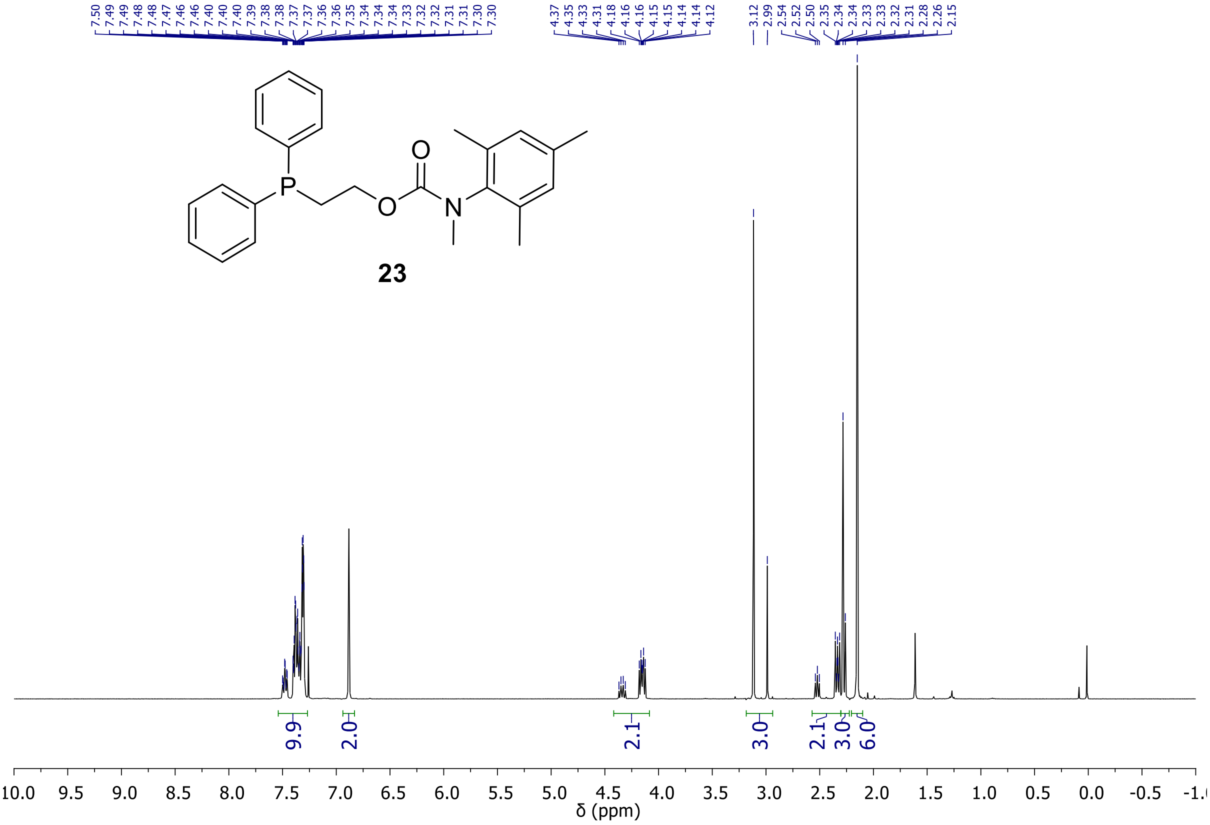

Figure S58. ^{1}H NMR spectra of **23** ($CDCl_3$, 400 MHz).

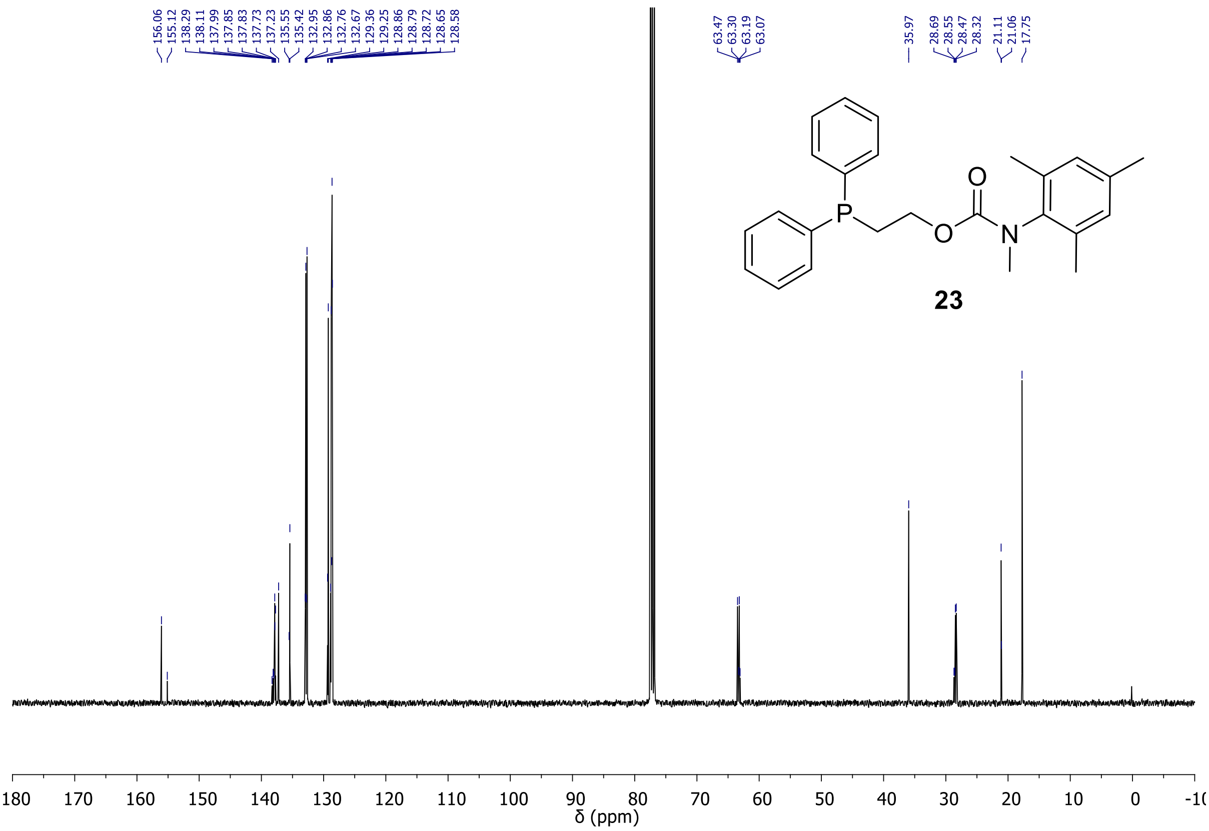

Figure S59. $^{13}C\{^{1}H\}$ NMR spectra of **23** ($CDCl_3$, 100 MHz).

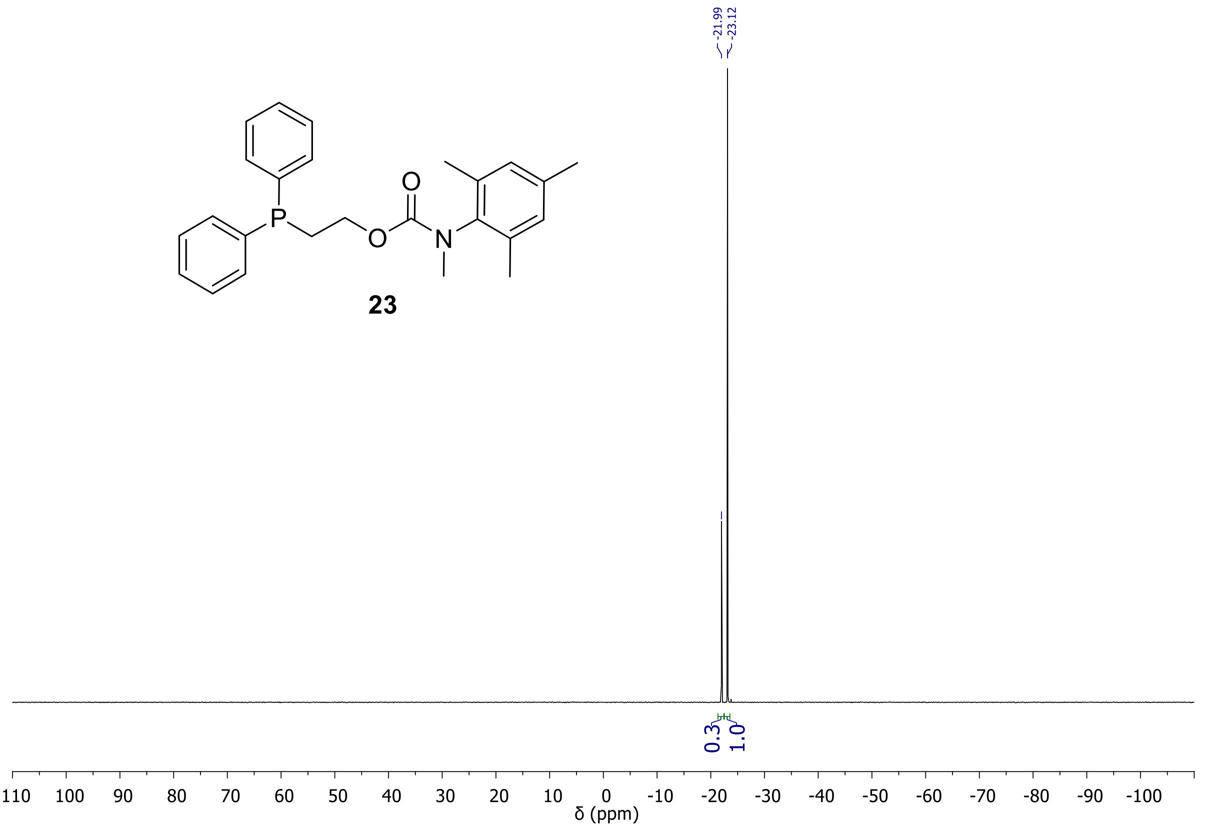

Figure S60. $^{31}P\{^{1}H\}$ NMR spectra of **23** ($CDCl_3$, 162 MHz).

Variable Temperature ^{1}H and ^{31}P{^{1}H} NMR of self-immolative systems 16, 17, 19 and 20

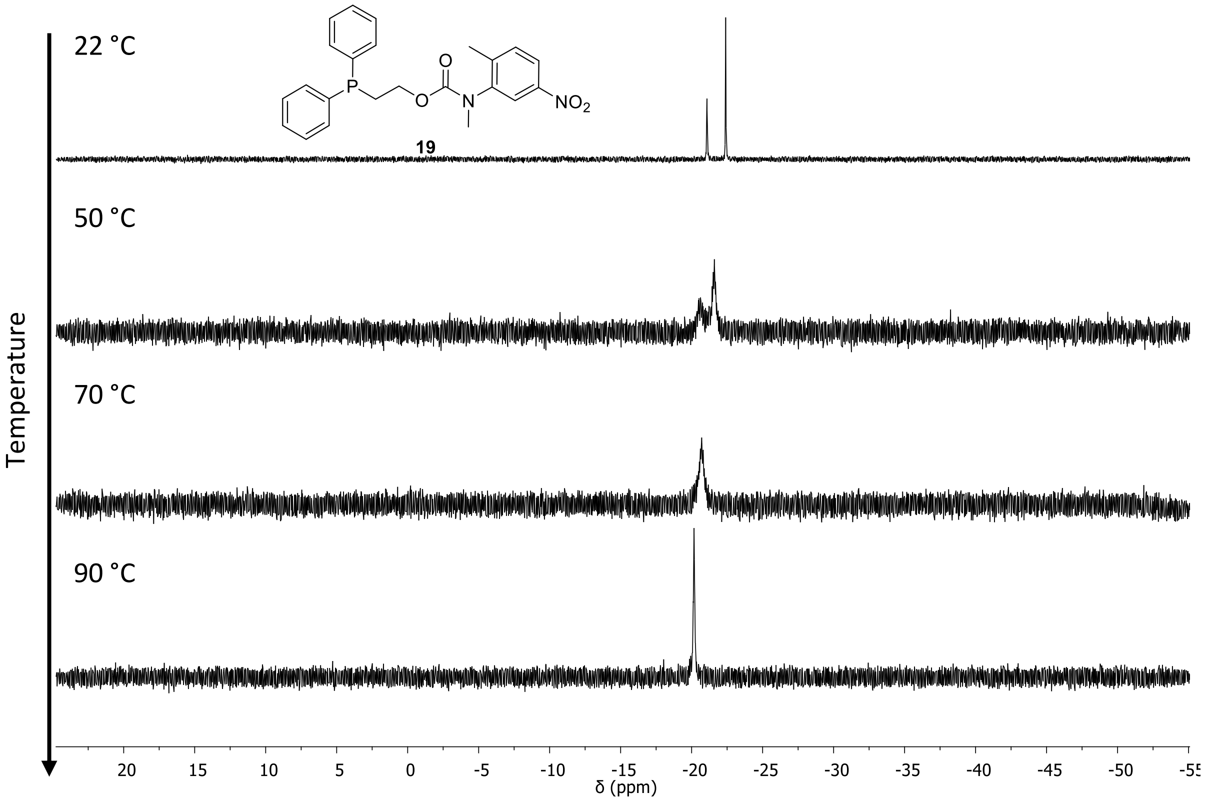

Figure S61. Stacked VT- ^{31}P{^{1}H} NMR spectra of **19** upon heating (DMSO-d_6, 203 MHz).

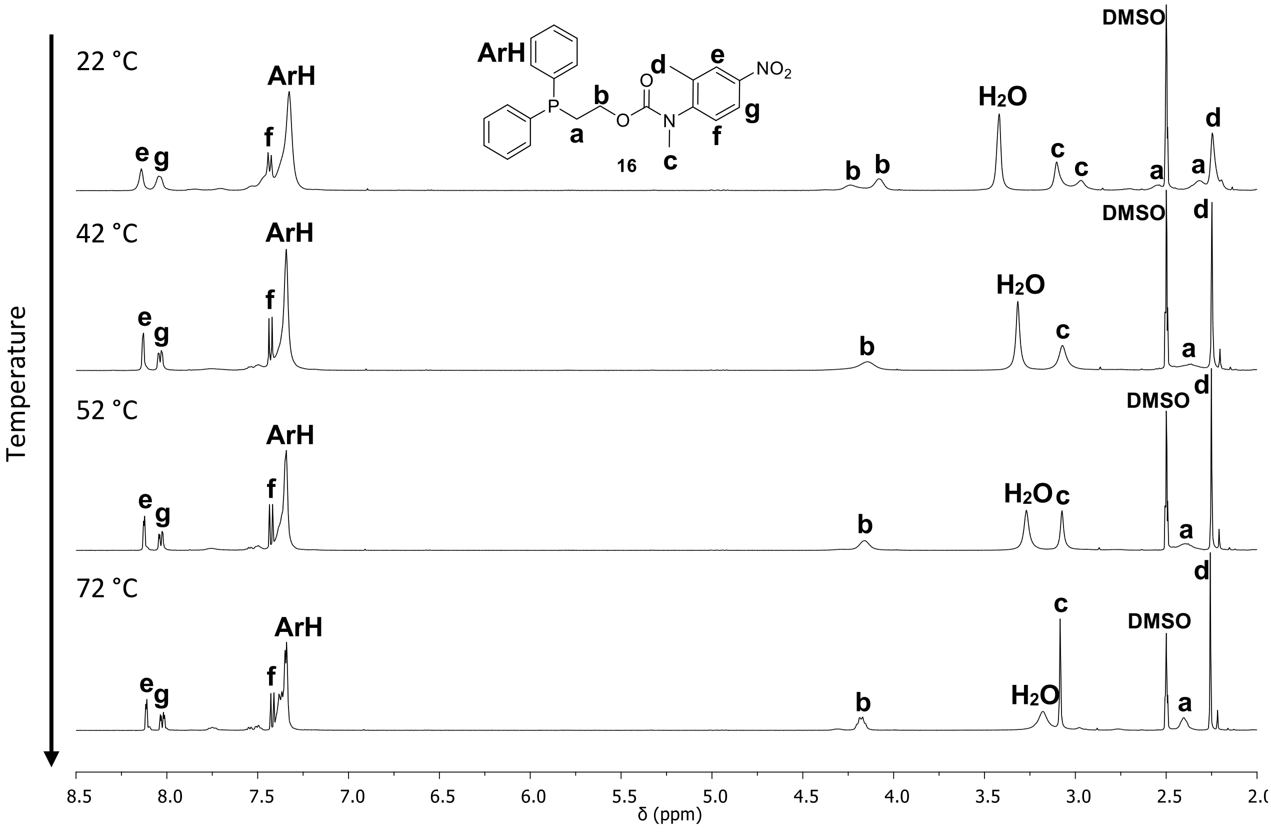

Figure S62. Stacked VT- ^{1}H NMR spectra of **16** upon heating (DMSO-d_6, 500 MHz).

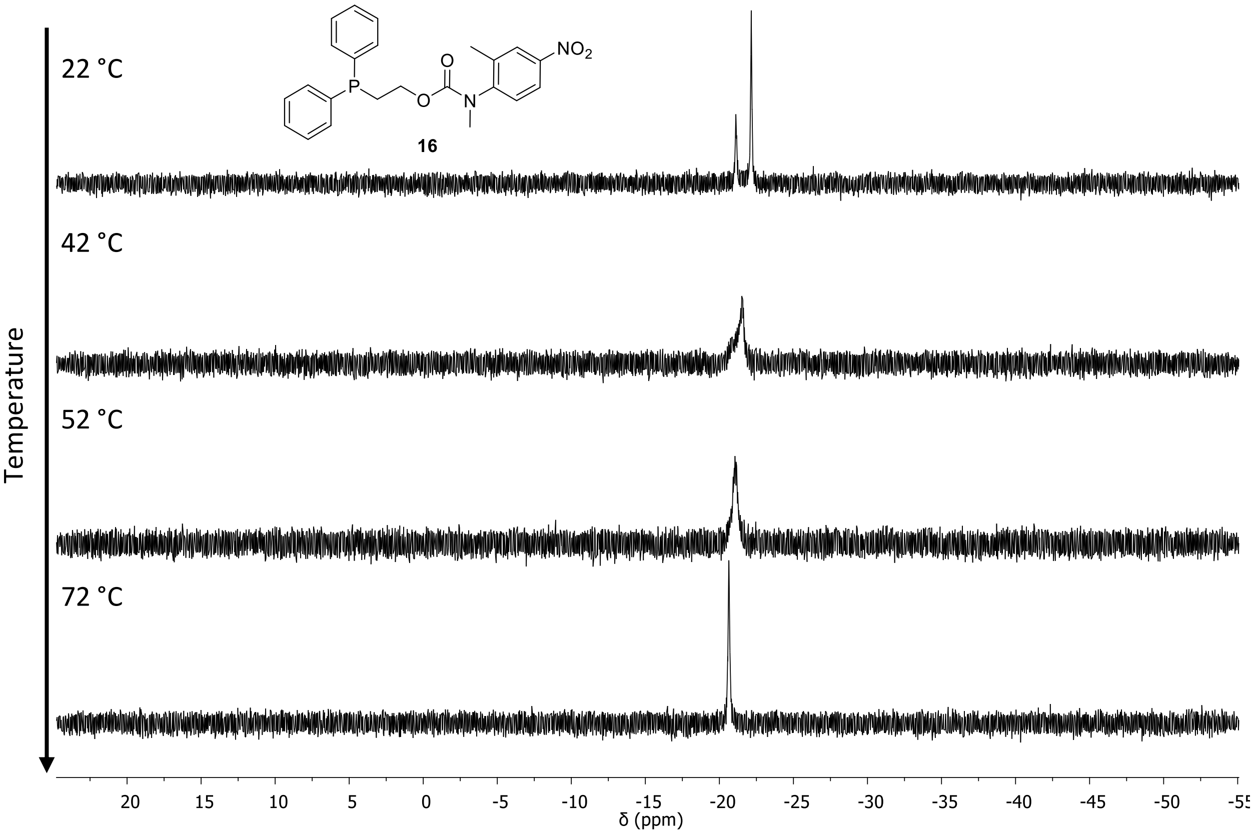

Figure S63. Stacked VT- $^{31}P\{^{1}H\}$ NMR spectra of **16** upon heating (DMSO-d_6, 203 MHz).

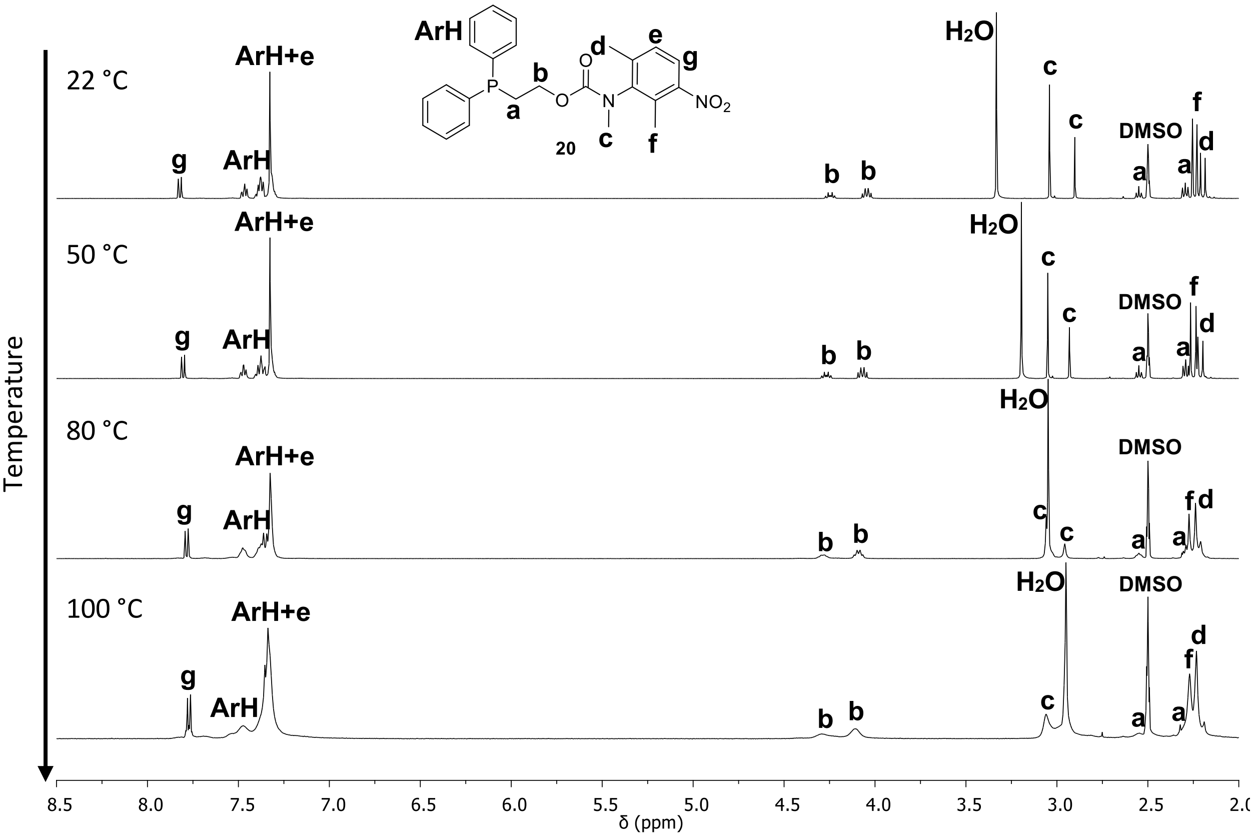

Figure S64. Stacked VT- ^{1}H NMR spectra of **20** upon heating (DMSO-d_6, 500 MHz).

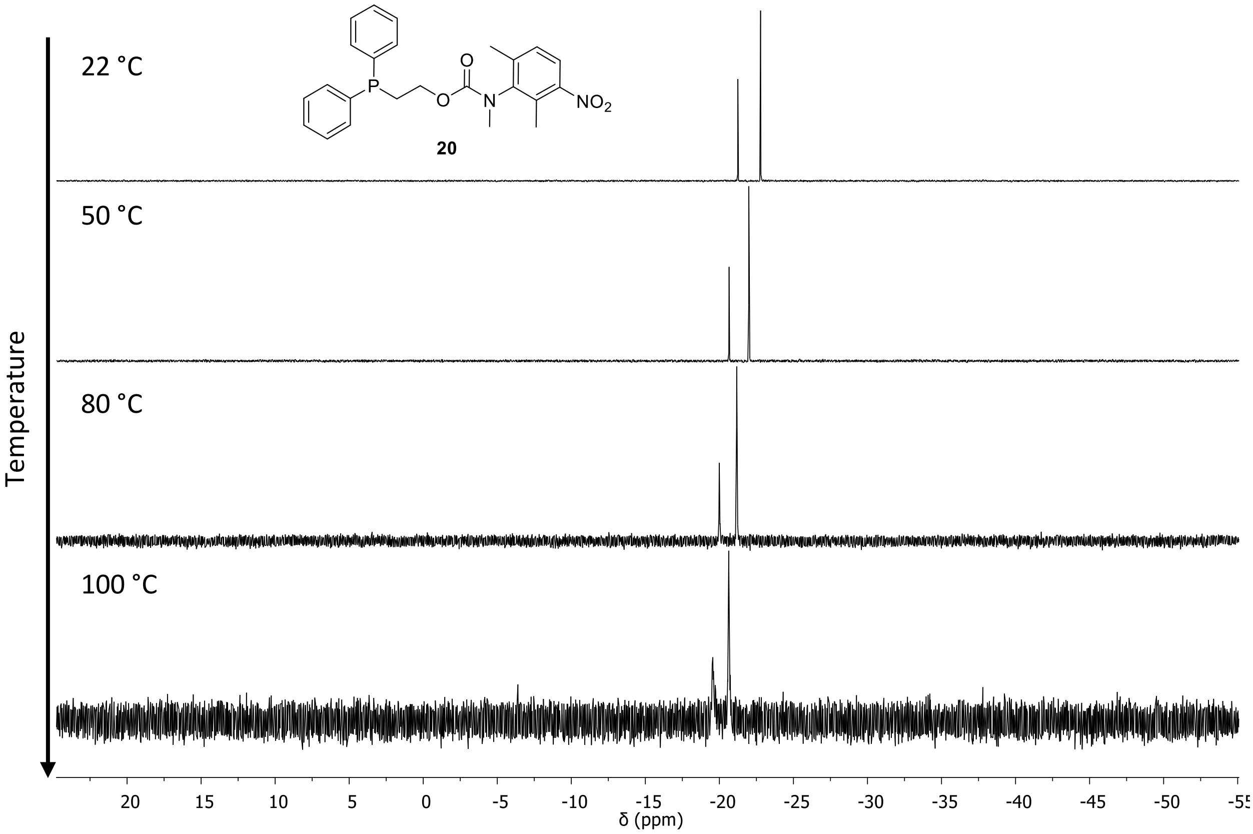

Figure S65. Stacked VT- $^{31}P\{^{1}H\}$ NMR spectra of **20** upon heating (DMSO-d_6, 203 MHz).

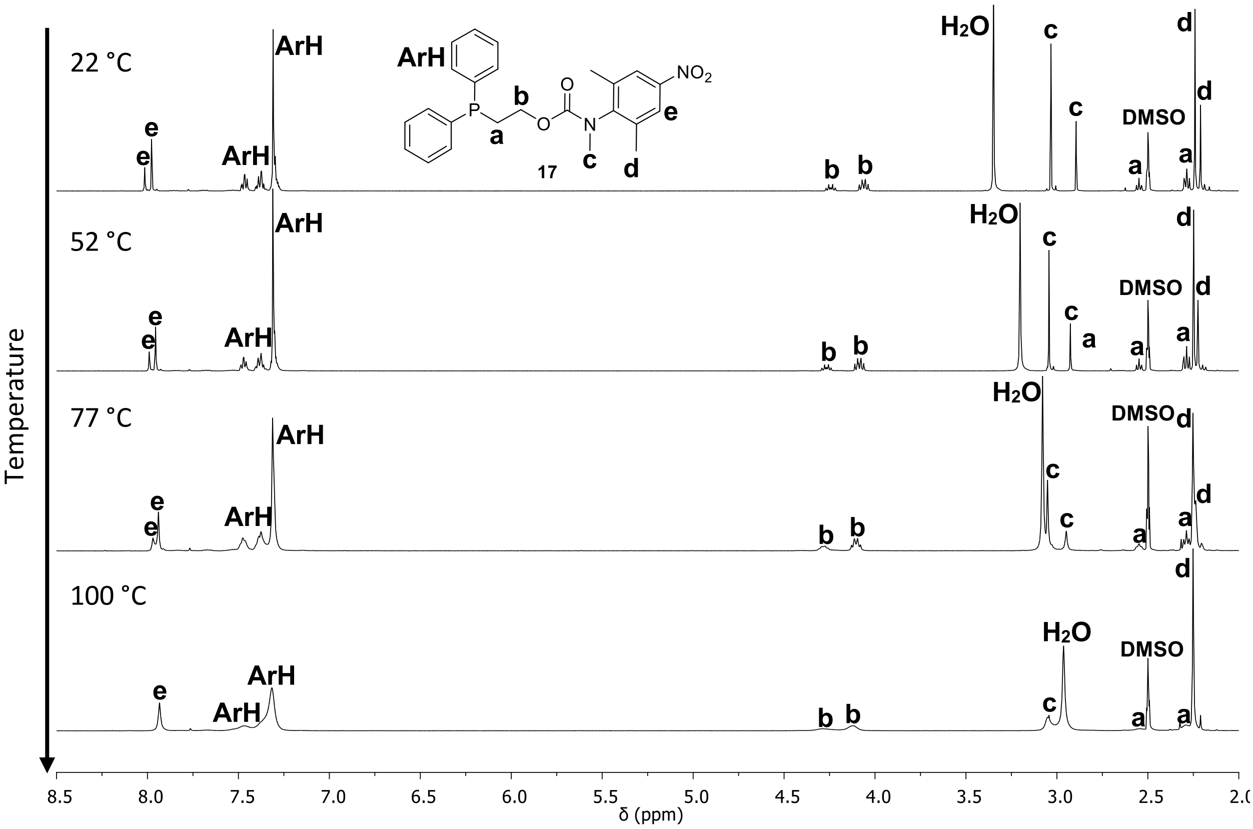

Figure S66. Stacked VT- ^{1}H NMR spectra of **17** upon heating (DMSO-d_6, 500 MHz).

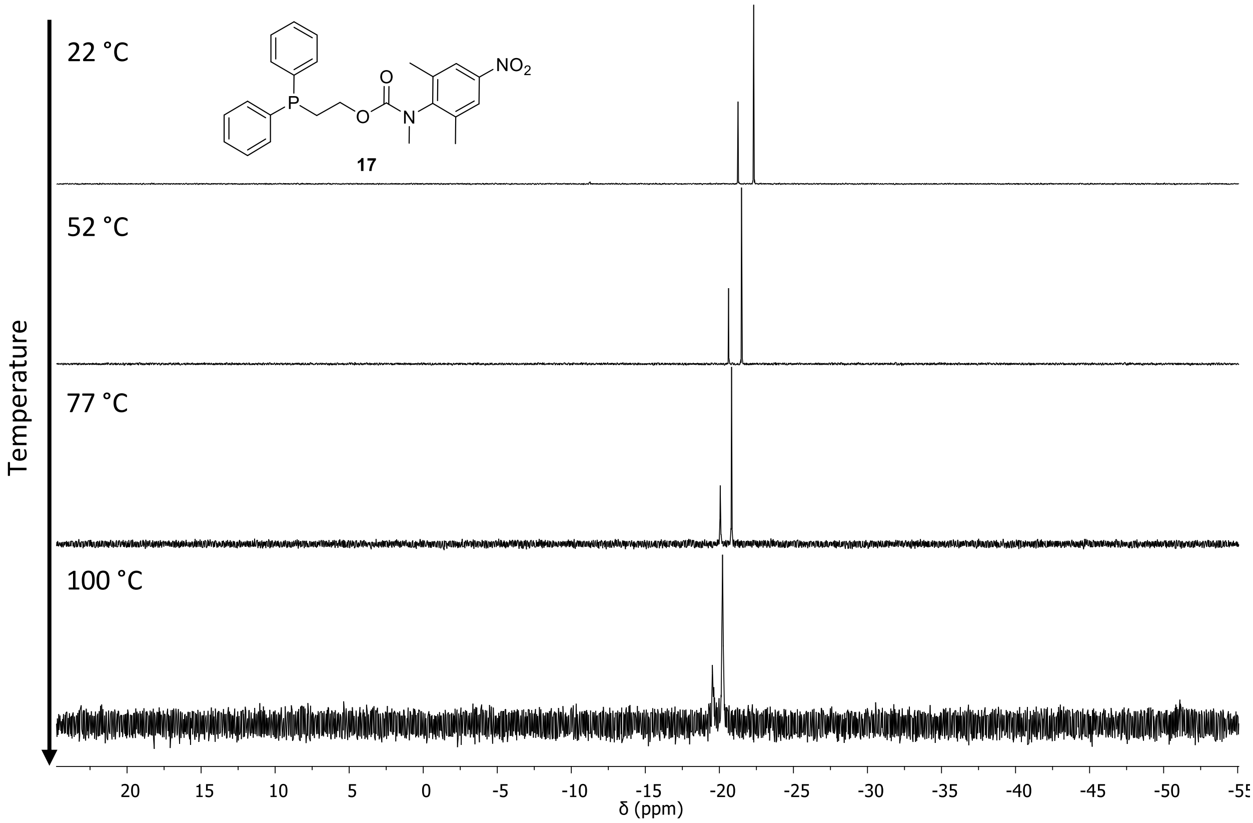

Figure S67. Stacked VT- $^{31}P\{^{1}H\}$ NMR spectra of **17** upon heating (DMSO-d_6, 203 MHz).

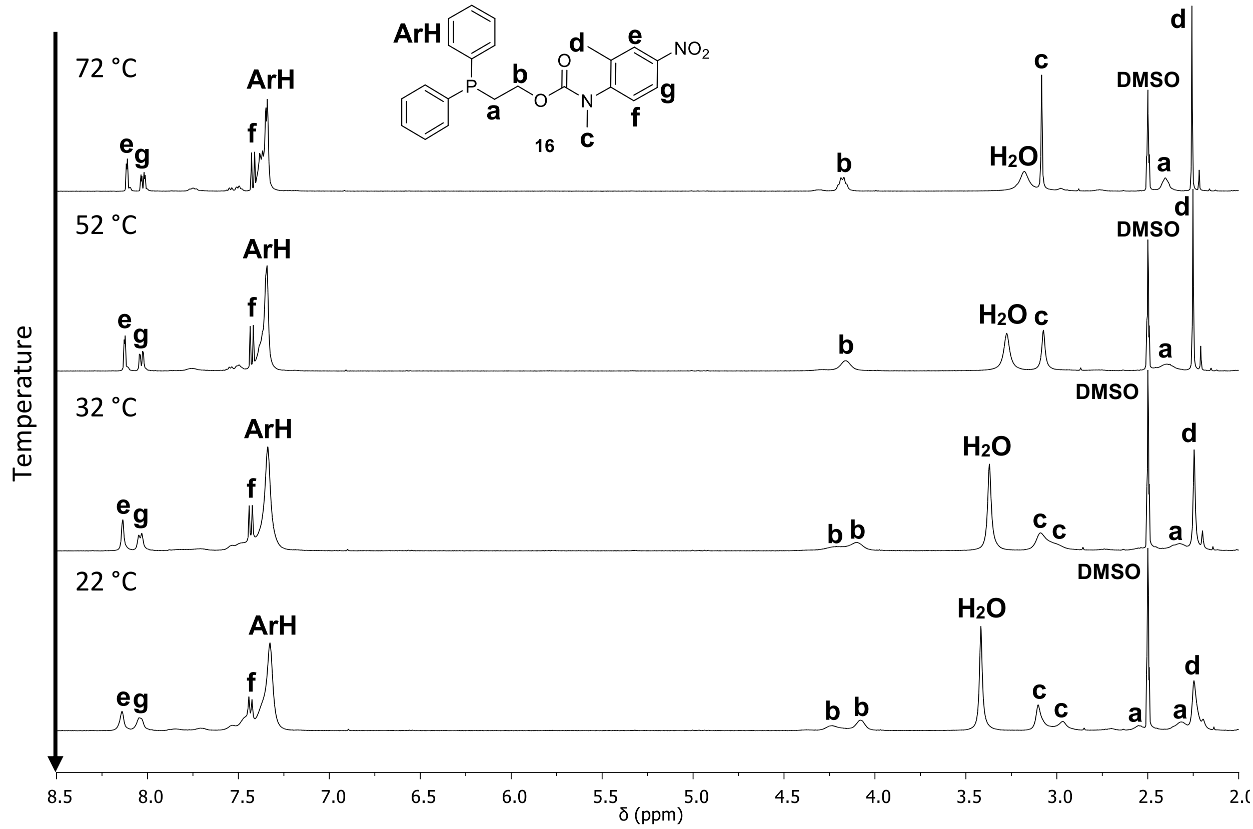

Figure S68. Stacked VT- ^{1}H NMR spectra of **16** upon cooling (DMSO-d_6, 500 MHz).

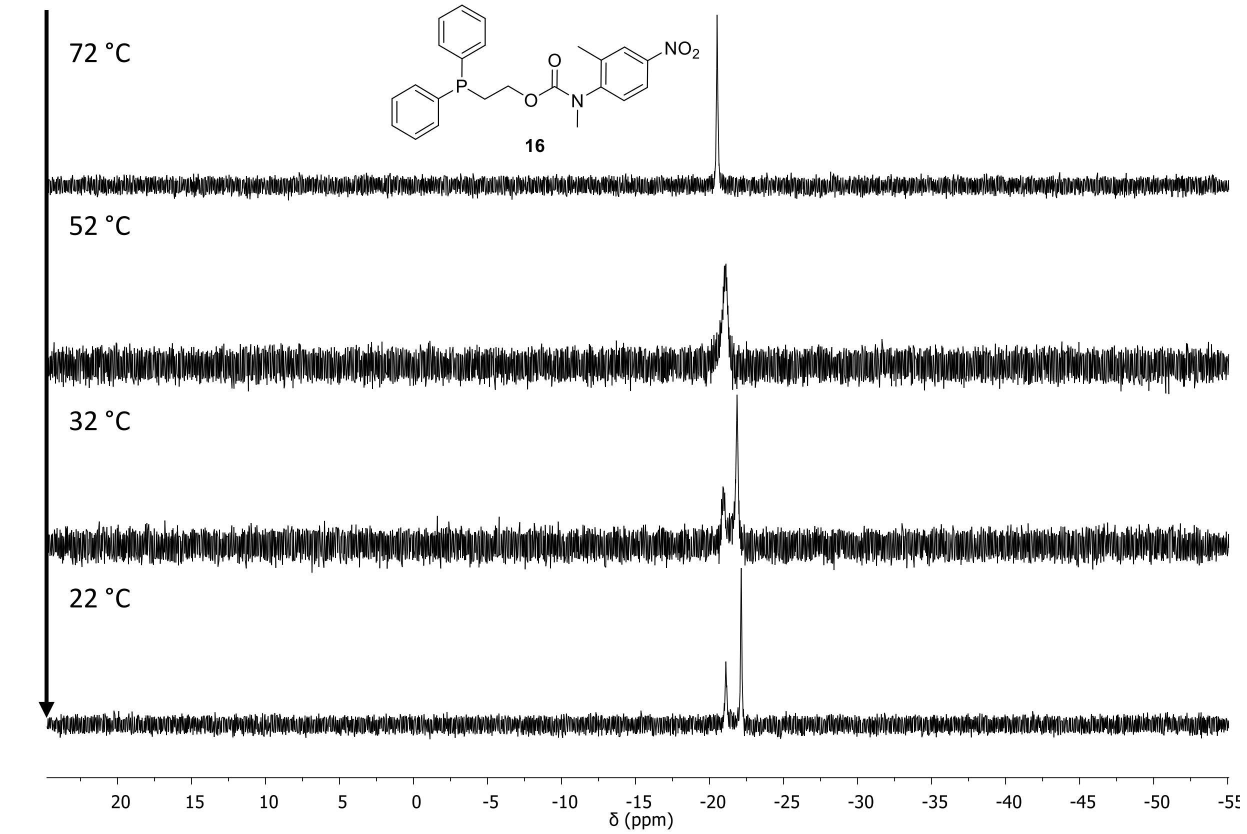

Figure S69. Stacked VT- $^{31}P\{^1H\}$ NMR spectra of **16** upon cooling (DMSO-d_6, 203 MHz).

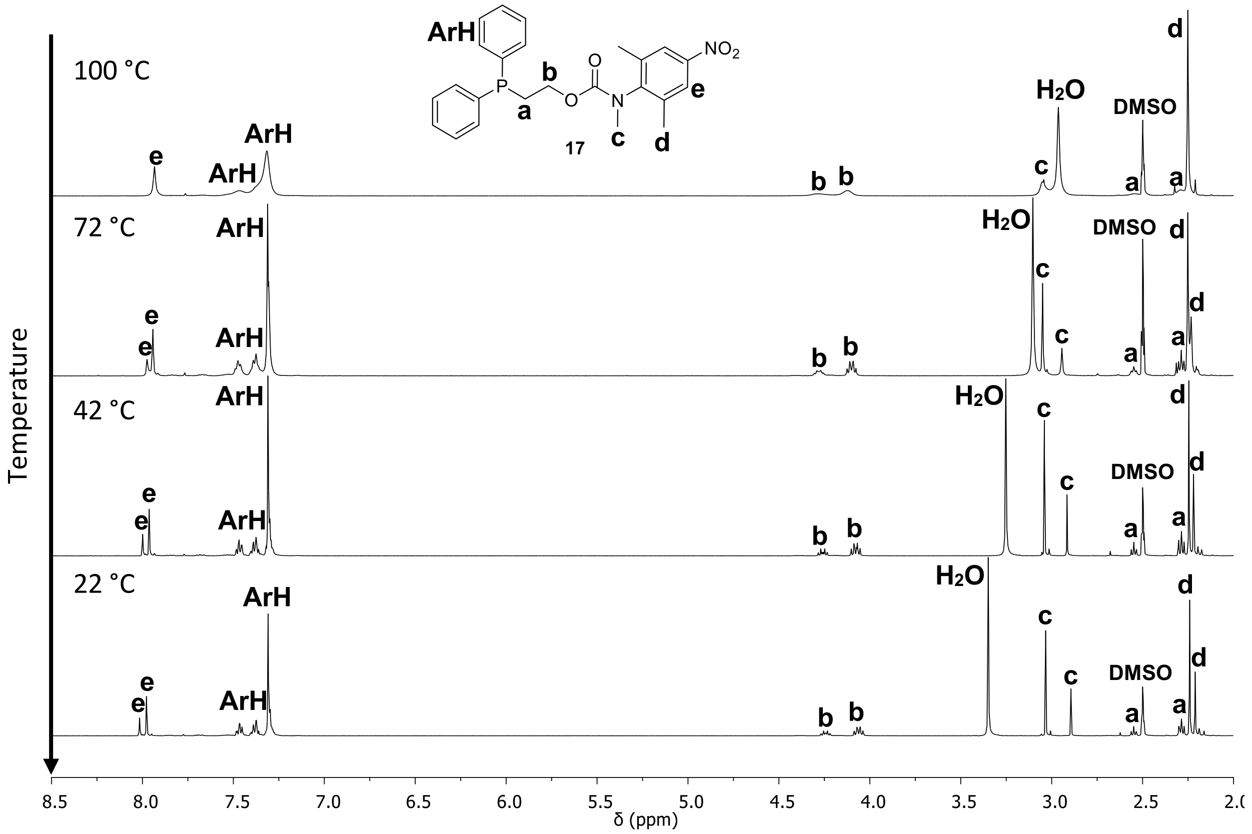

Figure S70. Stacked VT- ^1H NMR spectra of **17** upon cooling (DMSO-d_6, 500 MHz).

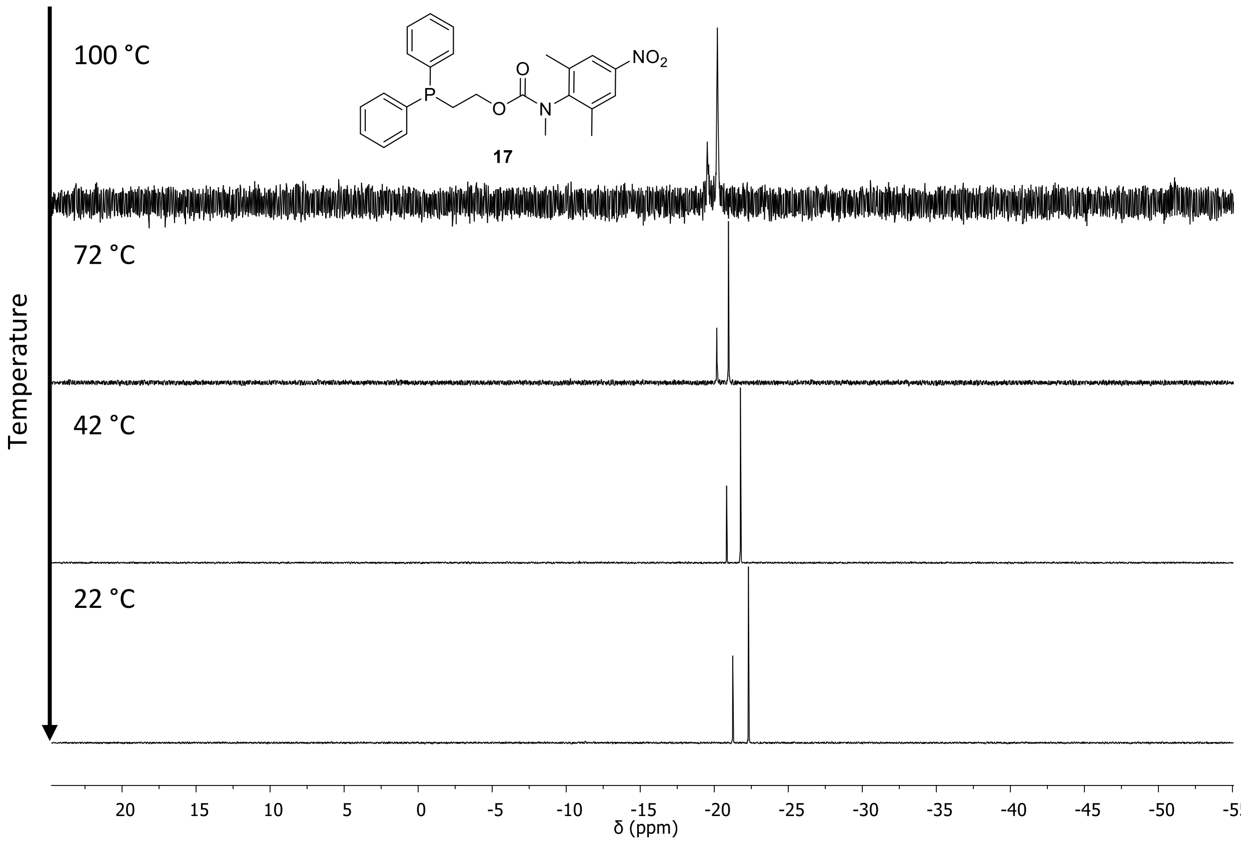

Figure S71. Stacked VT- $^{31}P\{^{1}H\}$ NMR spectra of **17** upon cooling (DMSO-d_6, 203 MHz).

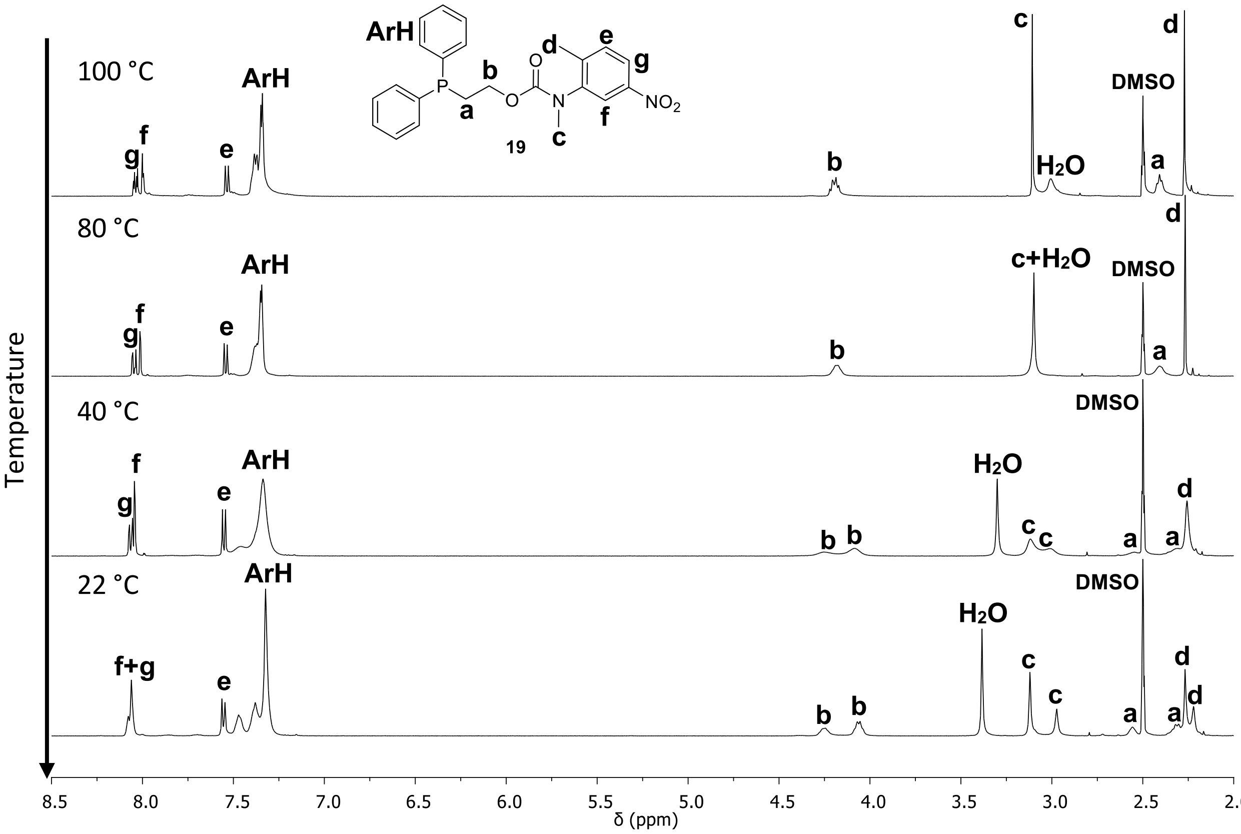

Figure S72. Stacked VT- ^{1}H NMR spectra of **19** upon cooling (DMSO-d_6, 500 MHz).

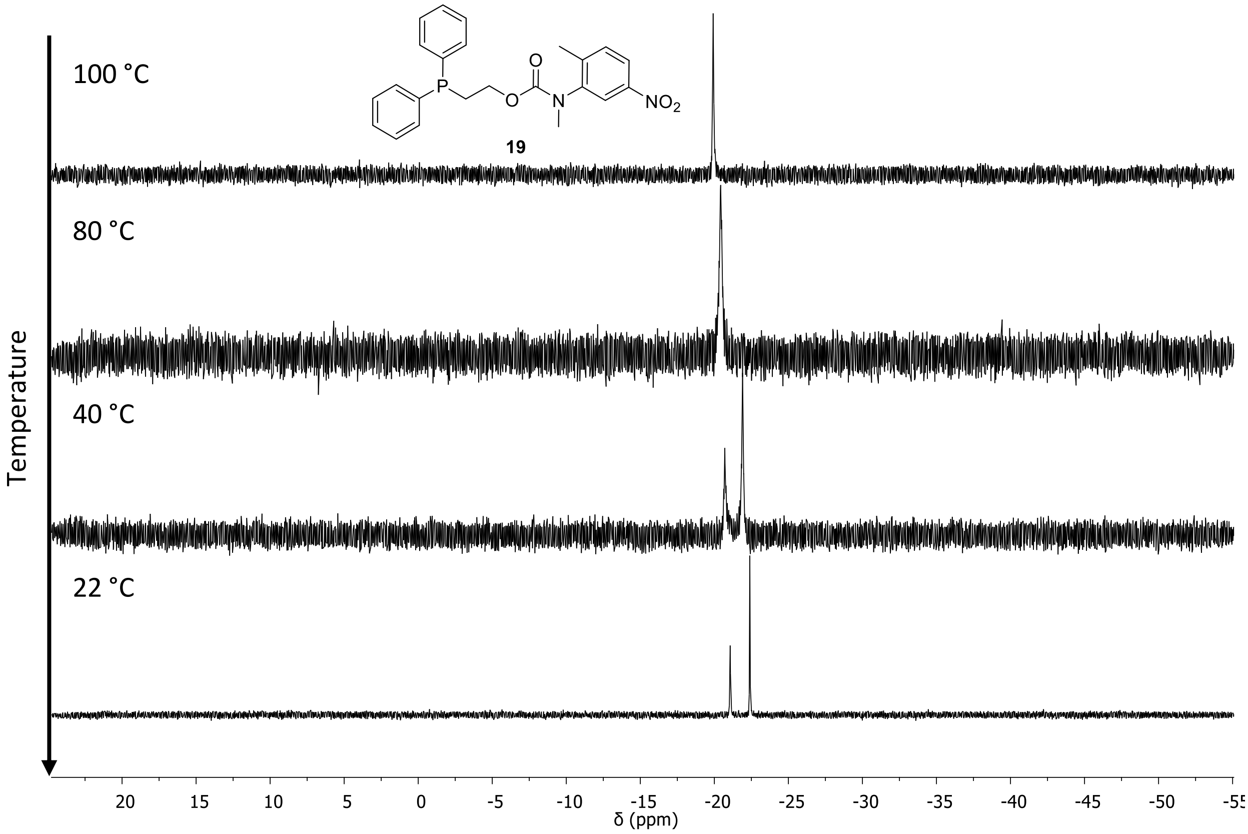

Figure S73. Stacked VT- $^{31}P\{^{1}H\}$ NMR spectra of **19** upon cooling (DMSO-d_6, 203 MHz).

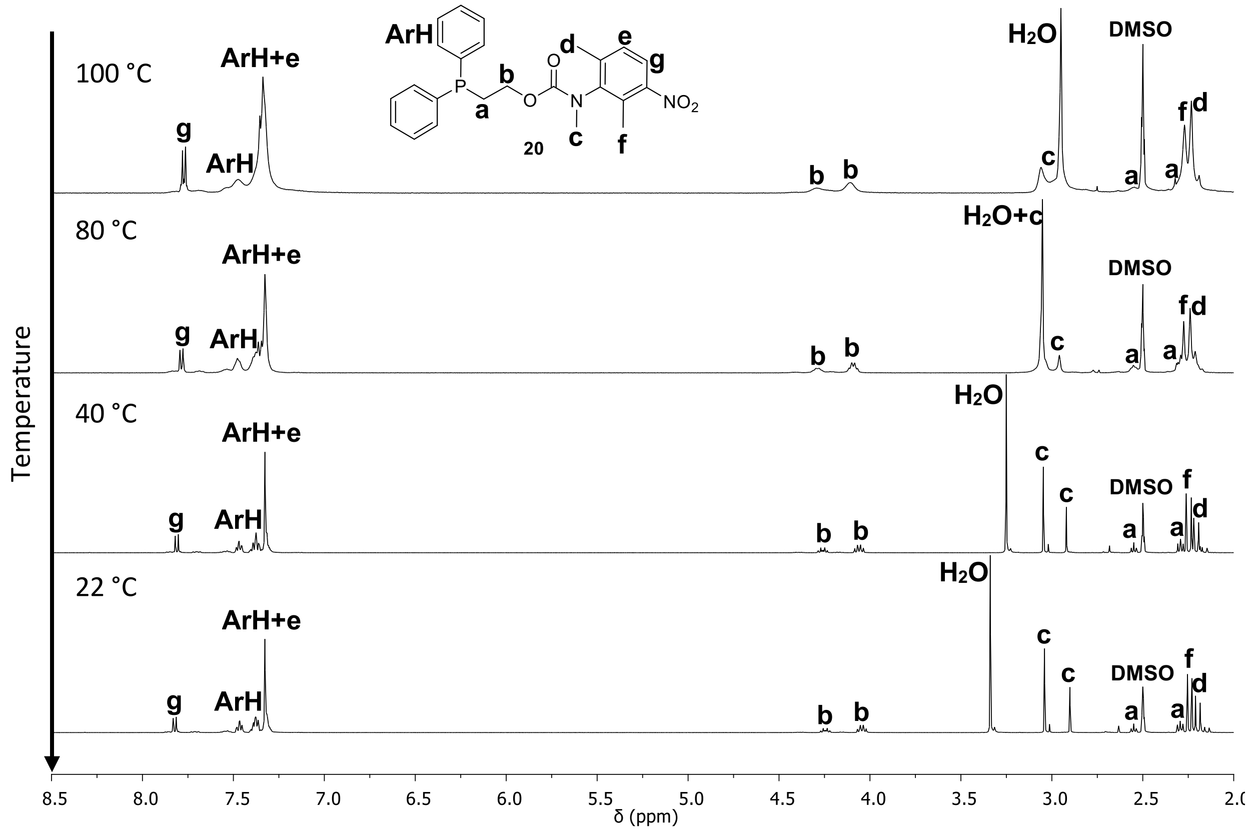

Figure S74. Stacked VT- ^{1}H NMR spectra of **20** upon cooling (DMSO-d_6, 500 MHz).

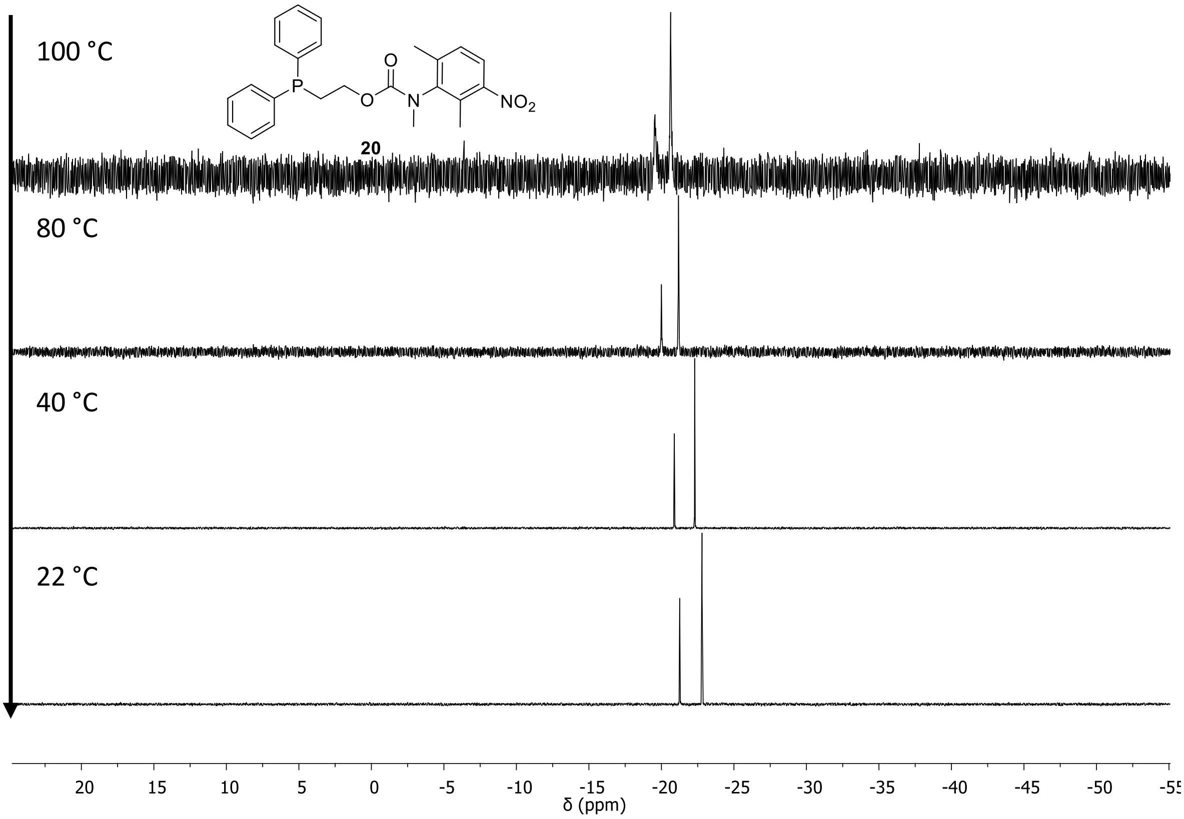

Figure S75. Stacked VT- $^{31}P\{^{1}H\}$ NMR spectra of **20** upon cooling (DMSO-d_6, 203 MHz).

Calculation of rotational barriers for self-immolative systems 16, 17, 19 and 20

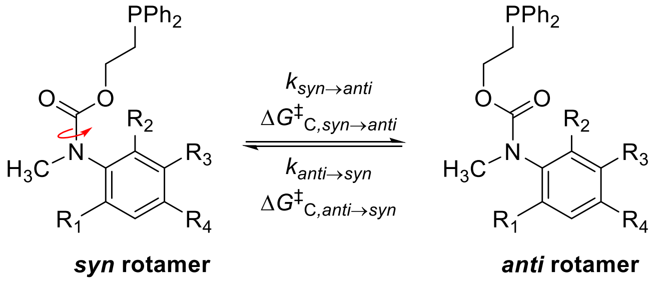

16: $R_1 = CH_3$, $R_2 = R_3 = H$, $R_4 = NO_2$
17: $R_1 = R_2 = CH_3$, $R_3 = H$, $R_4 = NO_2$
19: $R_1 = CH_3$, $R_2 = H$, $R_3 = NO_2$, $R_4 = H$
20: $R_1 = R_2 = CH_3$, $R_3 = NO_2$, $R_4 = H$

In our case, attempts to assign the resolved low temperature signals to *syn* or *anti* rotamer by NMR spectroscopy analysis were unsuccessful. As a consequence, we considered the major isomer as **rotamer A** and the minor isomer as **rotamer B** and used the approximation from Shanan-Atidi and Bar-Eli[1] to calculate the free energy of activation ($\Delta G^{\ddagger}$) for the rotational equilibrium of the different self-immolative systems **16, 17, 19 and 20**.

For unequal populations $P_A \neq P_B$:

At the coalescence: $$P_A - P_B = \Delta P = \left[\frac{X^2 - 2}{3}\right]^{3/2}\frac{1}{X}$$ Equation 1

Leading to: $$X^6 - 6X^4 - [12 - 27(\Delta P)^2]X^2 - 8 = 0$$ Equation 2

ΔP: population difference ($P_A - P_B$); $X = 2\pi\tau_C\Delta\nu$, with τ_C: lifetime at the coalescence [s] and $\Delta\nu$: maximum peak separation [Hz].

The reaction rates are k_A and k_B which obey $k_A P_A = k_B P_B$, *with* $k_A = 1/\tau_A$ and $k_B = 1/\tau_B$, leading to:

$$k_A = \frac{1}{2\tau_C}(1 - \Delta P) \quad \text{and} \quad k_B = \frac{1}{2\tau_C}(1 + \Delta P)$$ Equation 3

The value of the free energy of activation for both **rotamer A** ($\Delta G^{\ddagger}_A$) and **rotamer B** ($\Delta G^{\ddagger}_B$) are deduced using Eyring's expressions:

$$\Delta G^{\ddagger}_A = 4.57\ T_C\left[10.62 + \log\frac{X}{2\pi(1 - \Delta P)} + \log\frac{T_C}{\Delta\nu}\right] \text{cal.mol}^{-1}$$ Equation 4

$$\Delta G^{\ddagger}_B = 4.57\ T_C\left[10.62 + \log\frac{X}{2\pi(1 + \Delta P)} + \log\frac{T_C}{\Delta\nu}\right] \text{cal.mol}^{-1}$$ Equation 5

Energetic difference between the two rotamers:

$$\Delta G = RT_C \ln\frac{P_A}{P_B} = RT_C \ln\frac{1 + \Delta P}{1 - \Delta P} \quad \text{J.mol}^{-1}$$ Equation 6

T_C: coalescence temperature [K]; 1 cal.mol^{-1} = 4.184 J.mol^{-1}.

Table S1. Characteristic values determined for the rotational equilibrium of the different self-immolative systems **16**, **17**, **19** and **20** using VT- ^{1}H NMR spectroscopic analysis.

	SIE 16	SIE 17	SIE 19	SIE 20
**Δv CH_3N (Hz)**	53.2	55.6	58.8	56.0
**T_c (K)**	310.15	> 373.15	323.15	> 373.15
**P_A**	0.620	0.690	0.617	0.650
**P_B**	0.380	0.310	0.383	0.350
**ΔP**	0.240	0.380	0.234	0.300
**X**	1.951777	2.149811	1.942568	2.040267
**τ_C (s)**	0.00584	0.00615	0.00526	0.00580
**k_A (s^{-1})**	65.08	50.38	72.84	60.36
**k_B (s^{-1})**	106.18	112.13	117.35	112.10
**$\Delta G^{\ddagger}_A$ (kJ.mol^{-1})**	65.22	> 79.83	67.76	> 79.27
**$\Delta G^{\ddagger}_B$ (kJ.mol^{-1})**	63.96	> 77.35	66.48	> 77.35
**ΔG (kJ.mol^{-1})**	1.26	-	1.28	-

Degradation study in solution for the self-immolative systems 15-23

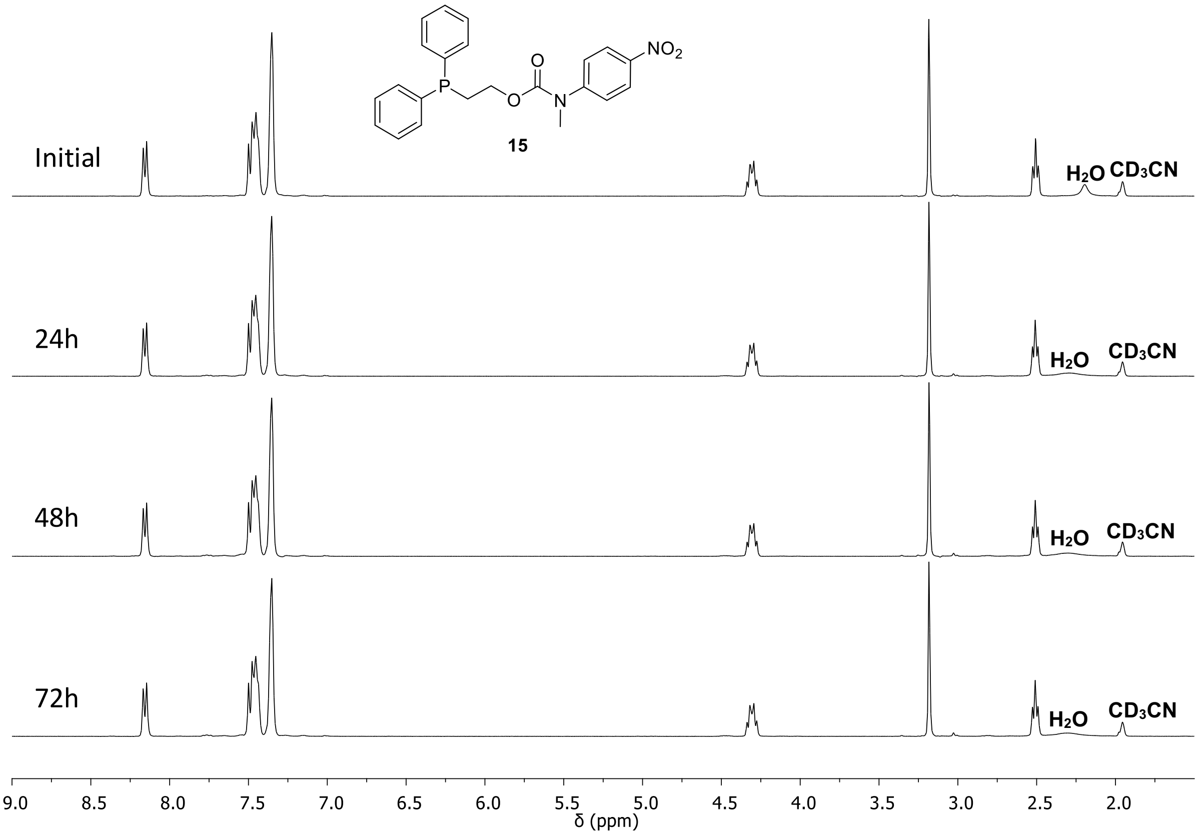

Figure S76. Stacked ^{1}H NMR spectra of **15** over 72 hours at room temperature in solution (0.1 mol.L^{-1}) (MeCN-d_3, 400 MHz).

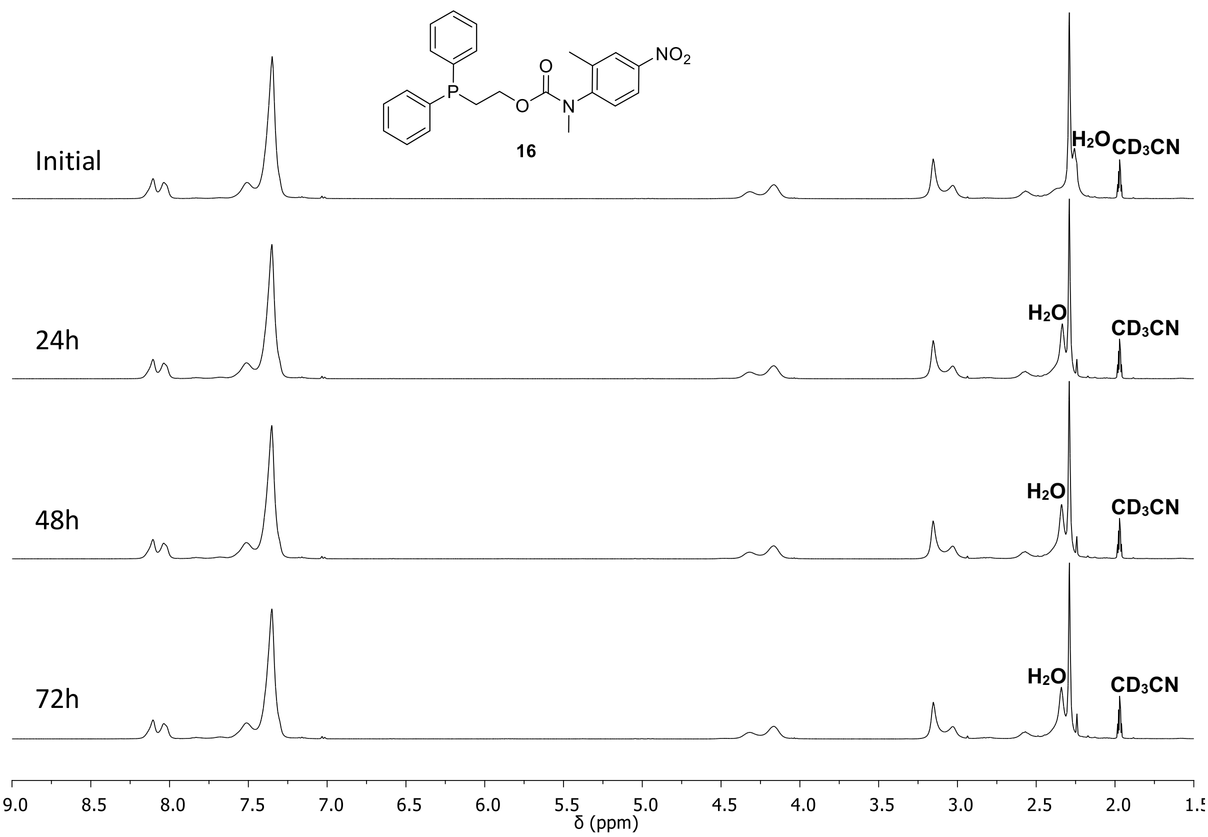

Figure S77. Stacked ^{1}H NMR spectra of **16** over 72 hours at room temperature in solution (0.1 mol.L^{-1}) (MeCN-d_3, 400 MHz).

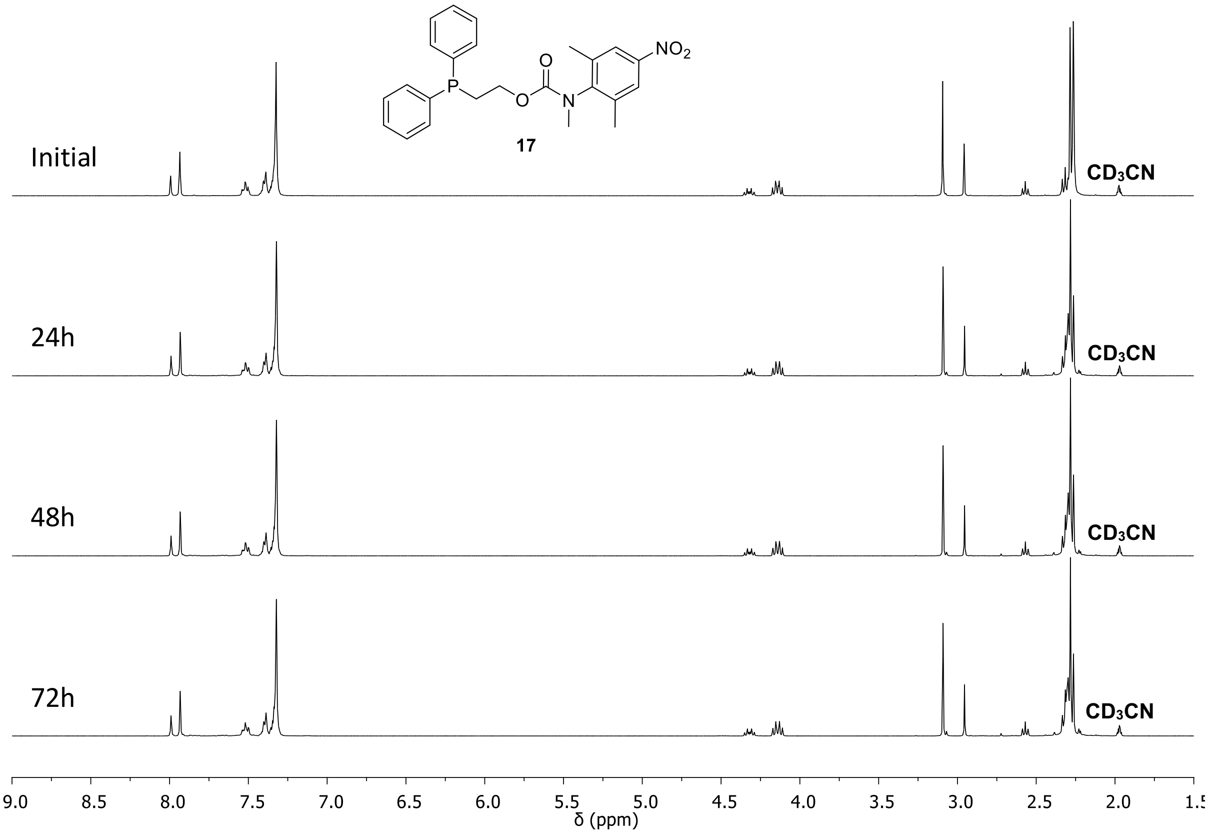

Figure S78. Stacked ^{1}H NMR spectra of **17** over 72 hours at room temperature in solution (0.1 mol.L^{-1}) (MeCN-d_3, 400 MHz).

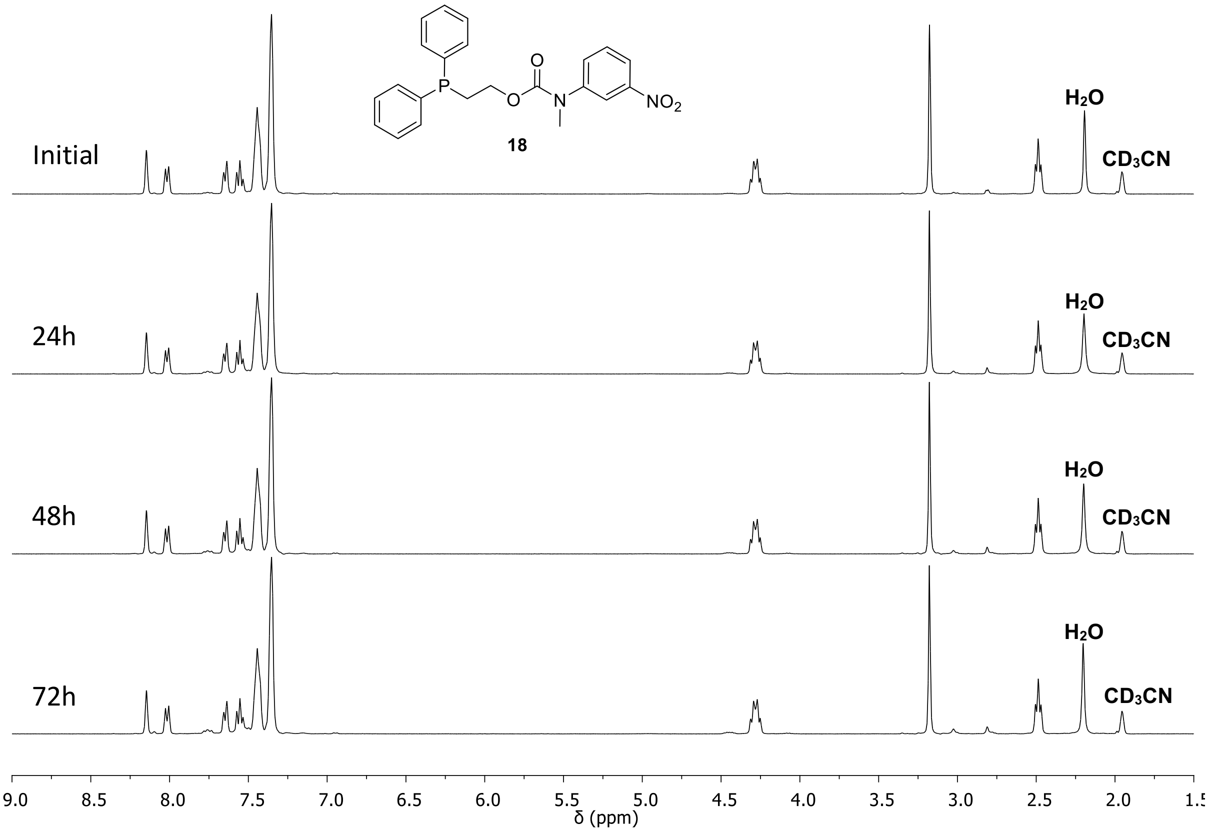

Figure S79. Stacked ^{1}H NMR spectra of **18** over 72 hours at room temperature in solution (0.1 mol.L^{-1}) (MeCN-d_3, 400 MHz).

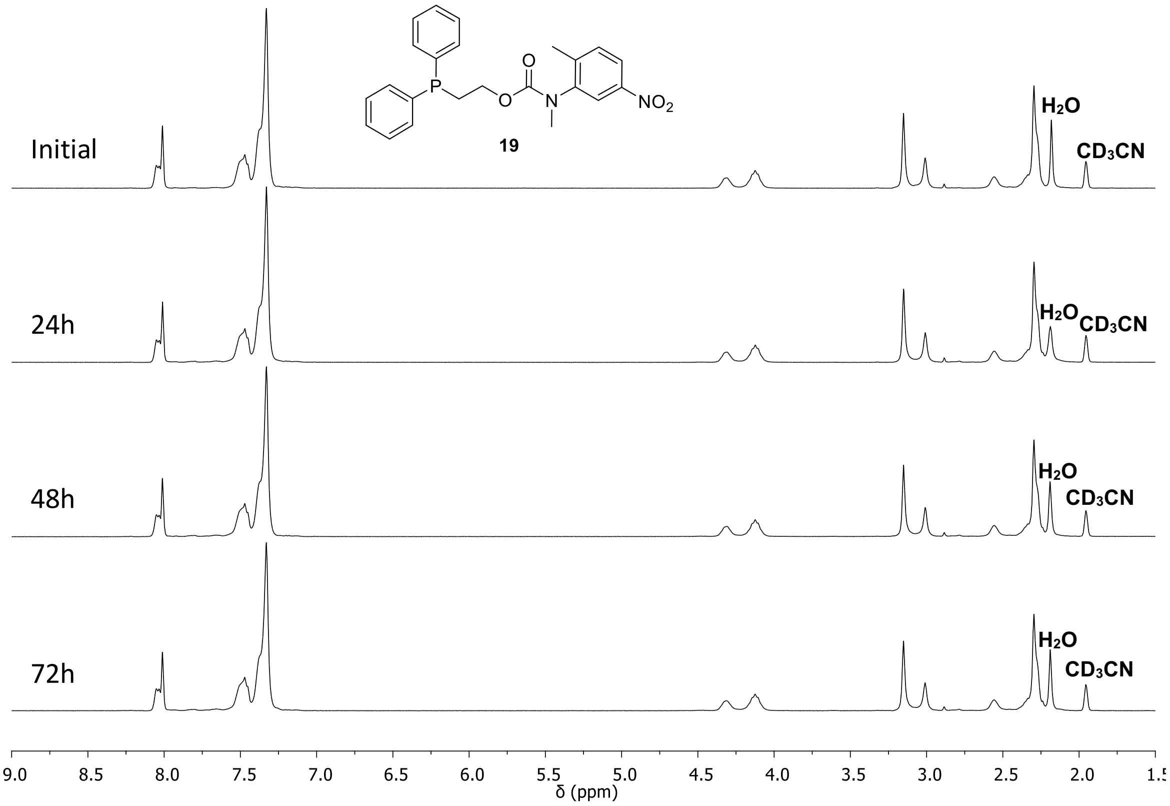

Figure S80. Stacked ^{1}H NMR spectra of **19** over 72 hours at room temperature in solution (0.1 mol.L^{-1}) (MeCN-d_3, 400 MHz).

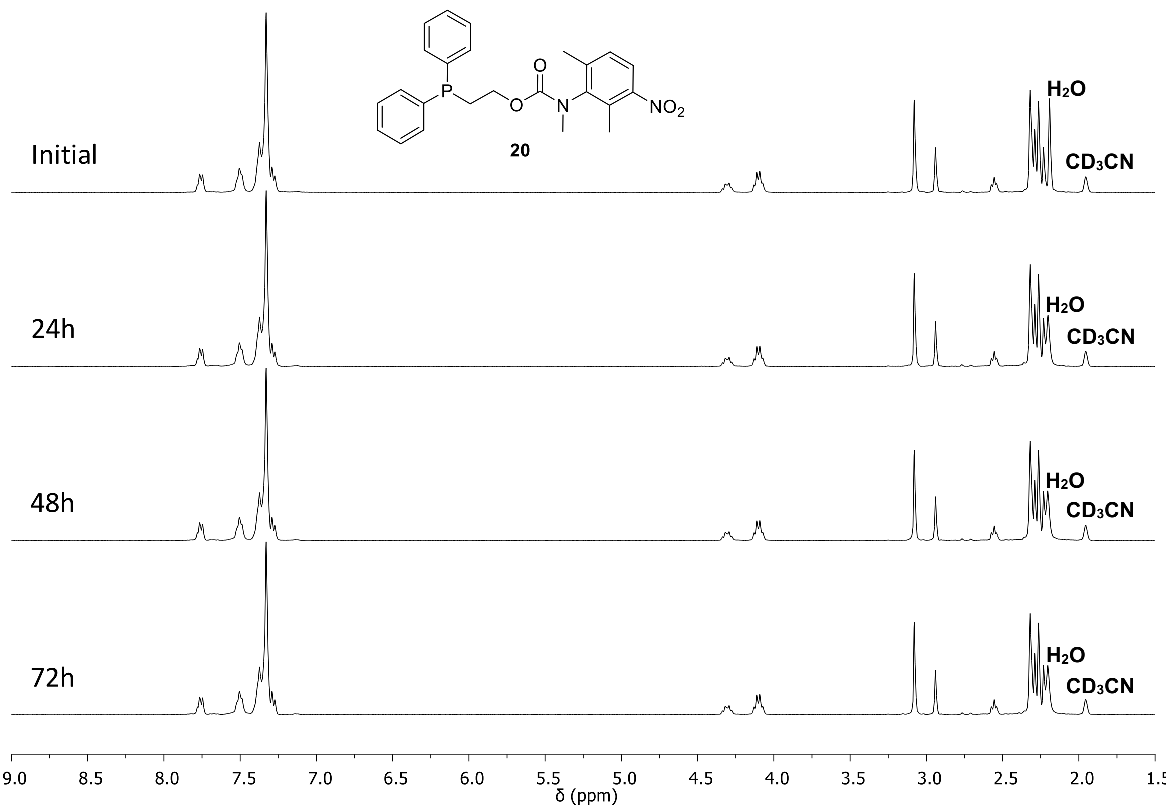

Figure S81. Stacked ^{1}H NMR spectra of **20** over 72 hours at room temperature in solution (0.1 mol.L^{-1}) (MeCN-d_3, 400 MHz).

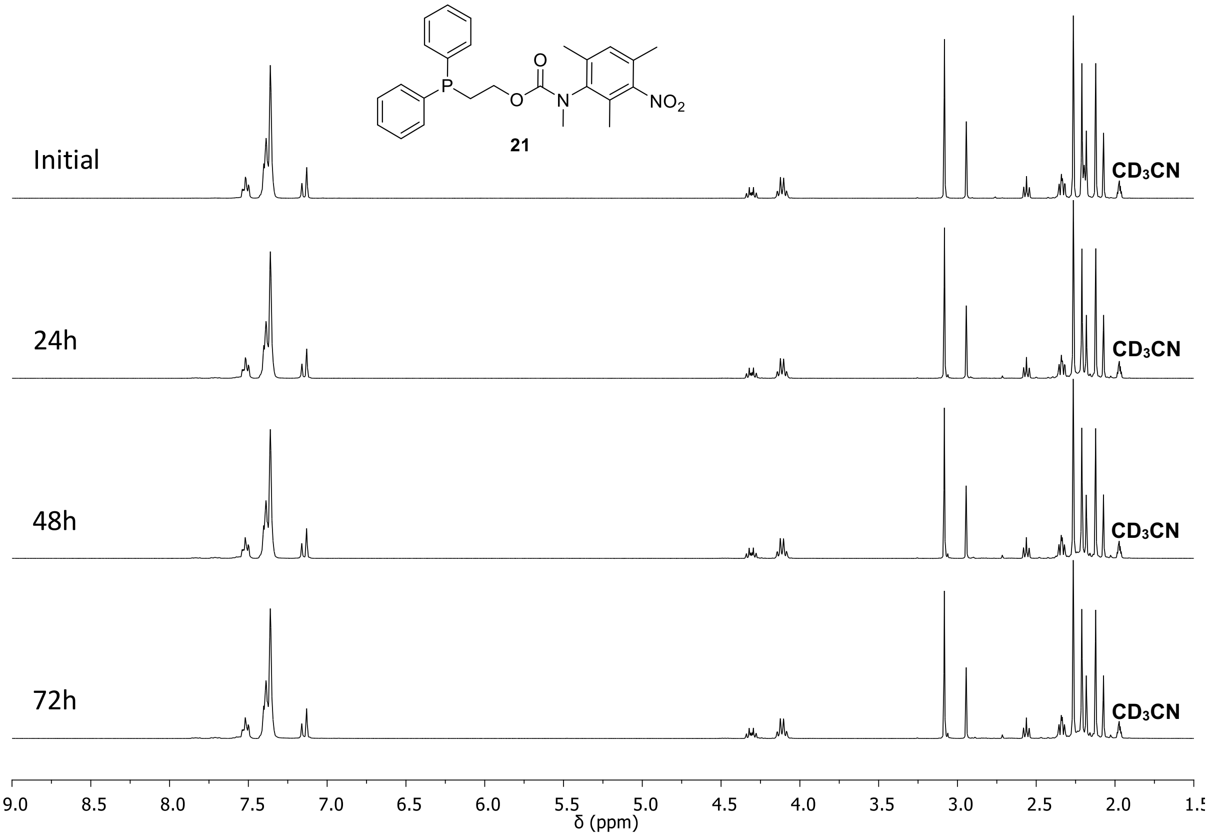

Figure S82. Stacked ^{1}H NMR spectra of **21** over 72 hours at room temperature in solution (0.1 mol.L^{-1}) (MeCN-d_3, 400 MHz).

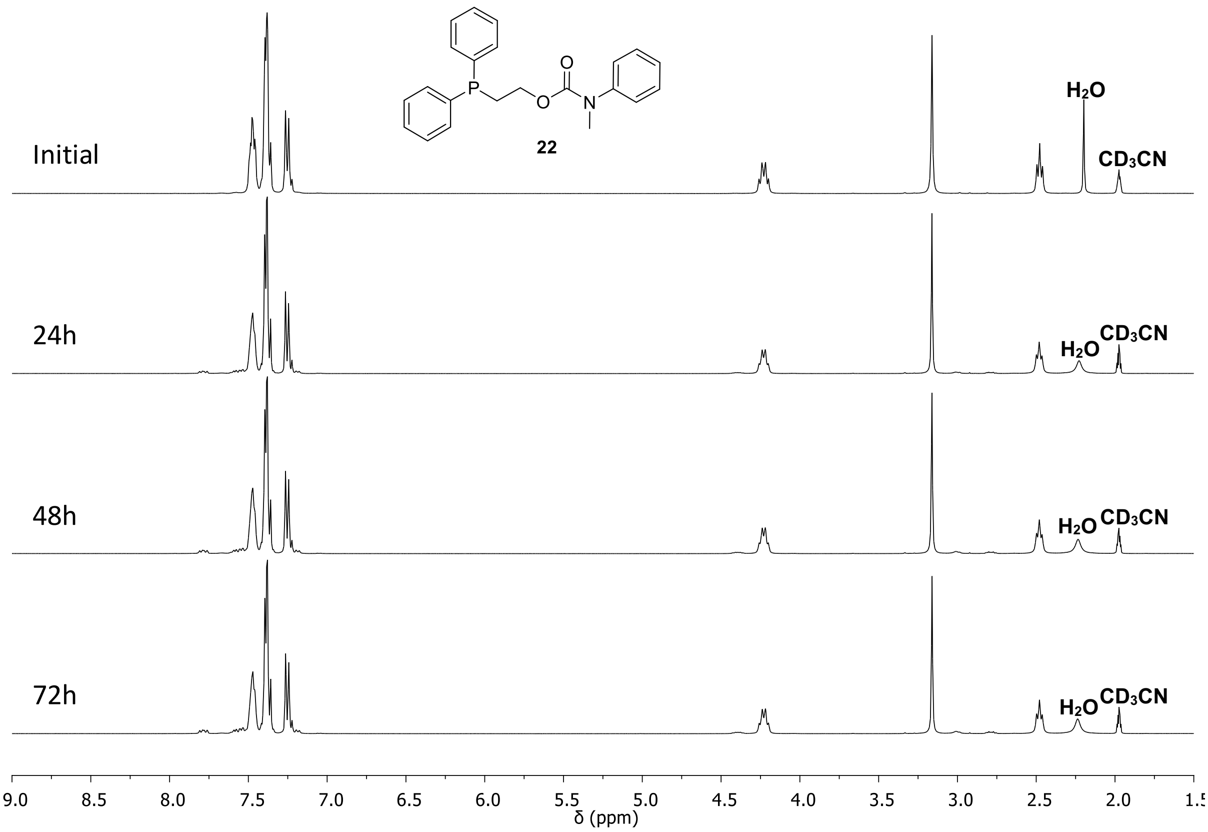

Figure S83. Stacked ^{1}H NMR spectra of **22** over 72 hours at room temperature in solution (0.1 mol.L^{-1}) (MeCN-d_3, 400 MHz).

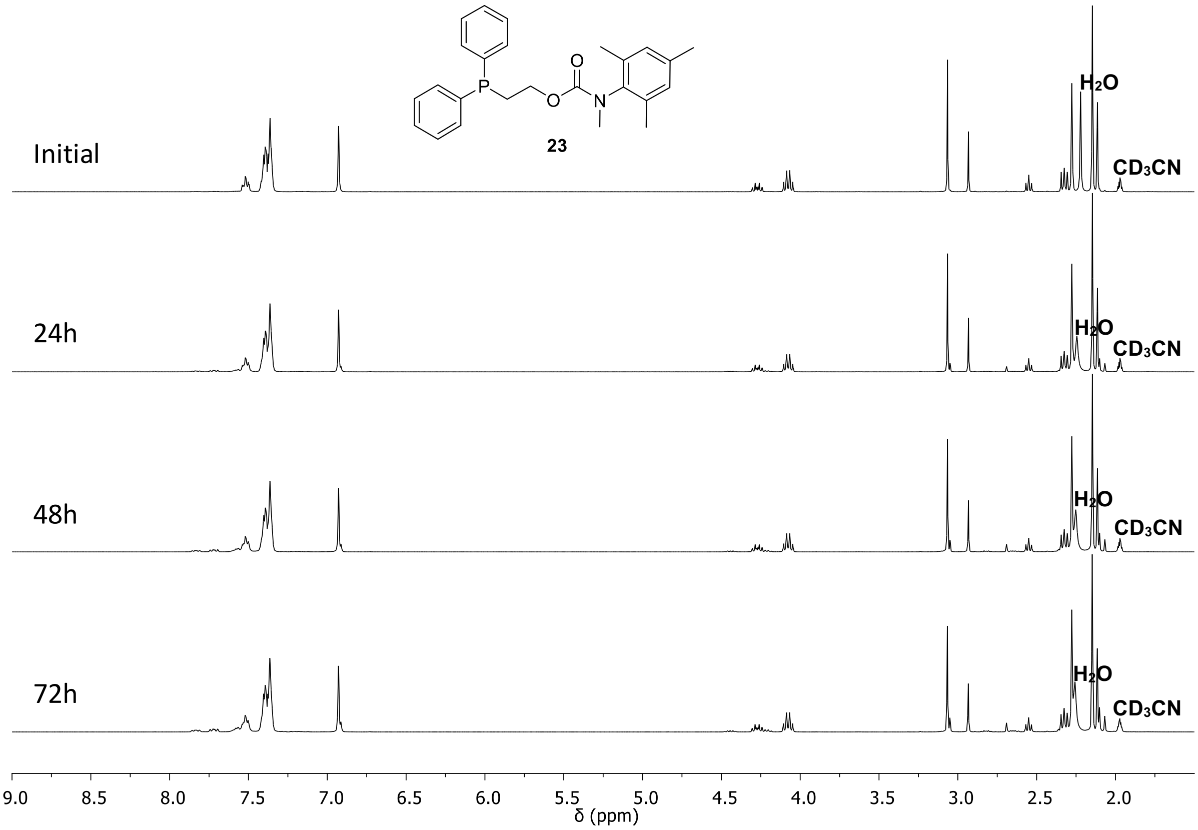

Figure S84. Stacked ^{1}H NMR spectra of **23** over 72 hours at room temperature in solution (0.1 mol.L^{-1}) (MeCN-d_3, 400 MHz).

Degradation study for the neat self-immolative systems 15-23

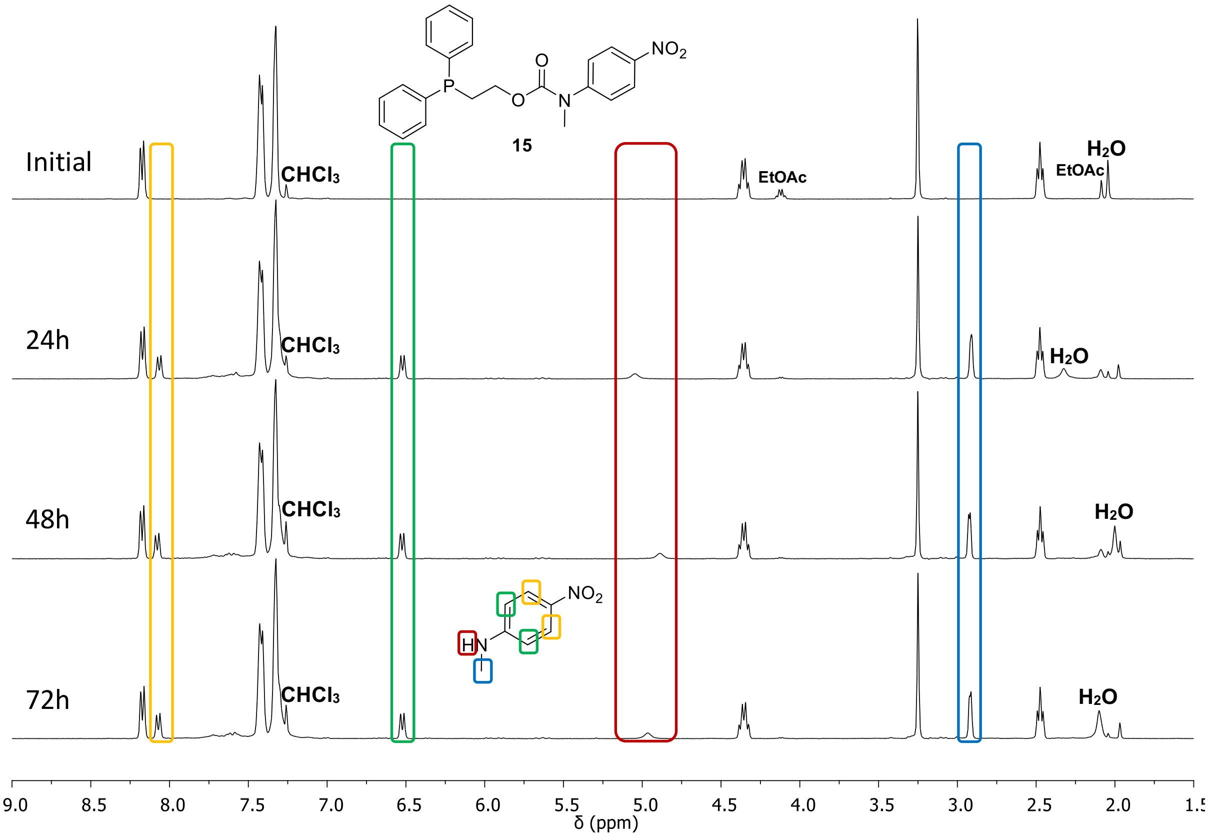

Figure S85. Stacked ^{1}H NMR spectra of **15** ($CDCl_3$, 400 MHz) over 72 hours at room temperature under *vacuum*, as a neat liquid.

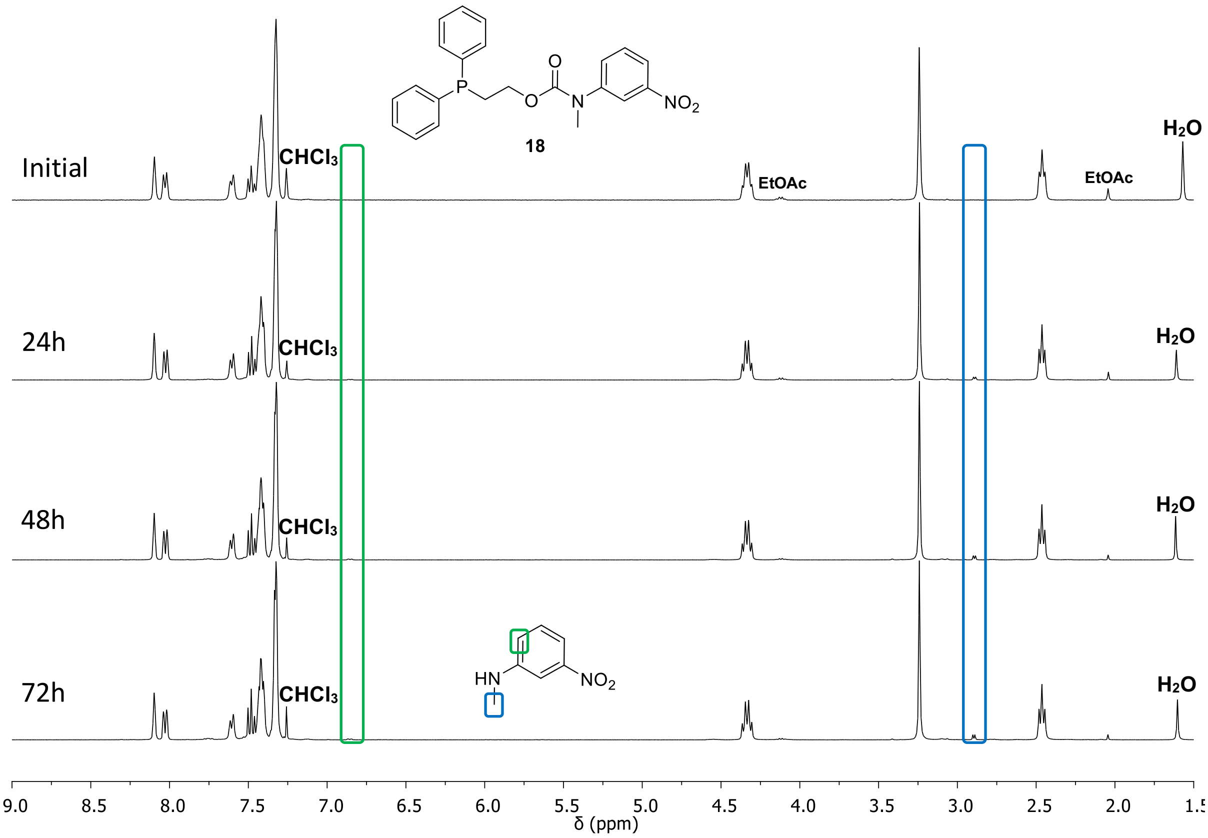

Figure S86. Stacked ^{1}H NMR spectra of **18** ($CDCl_3$, 400 MHz) over 72 hours at room temperature under *vacuum*, as a neat liquid.

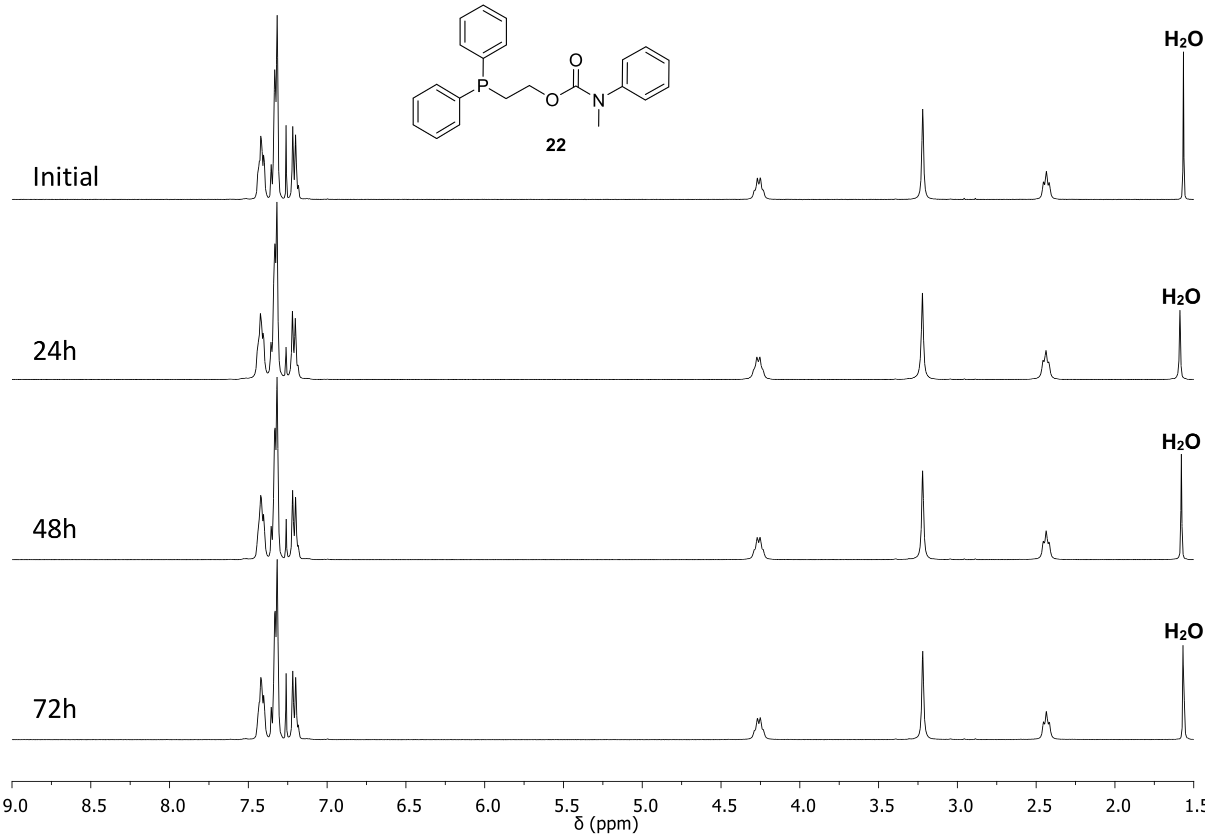

Figure S87. Stacked ^{1}H NMR spectra of **22** ($CDCl_3$, 400 MHz) over 72 hours at room temperature under *vacuum*, as a neat solid.

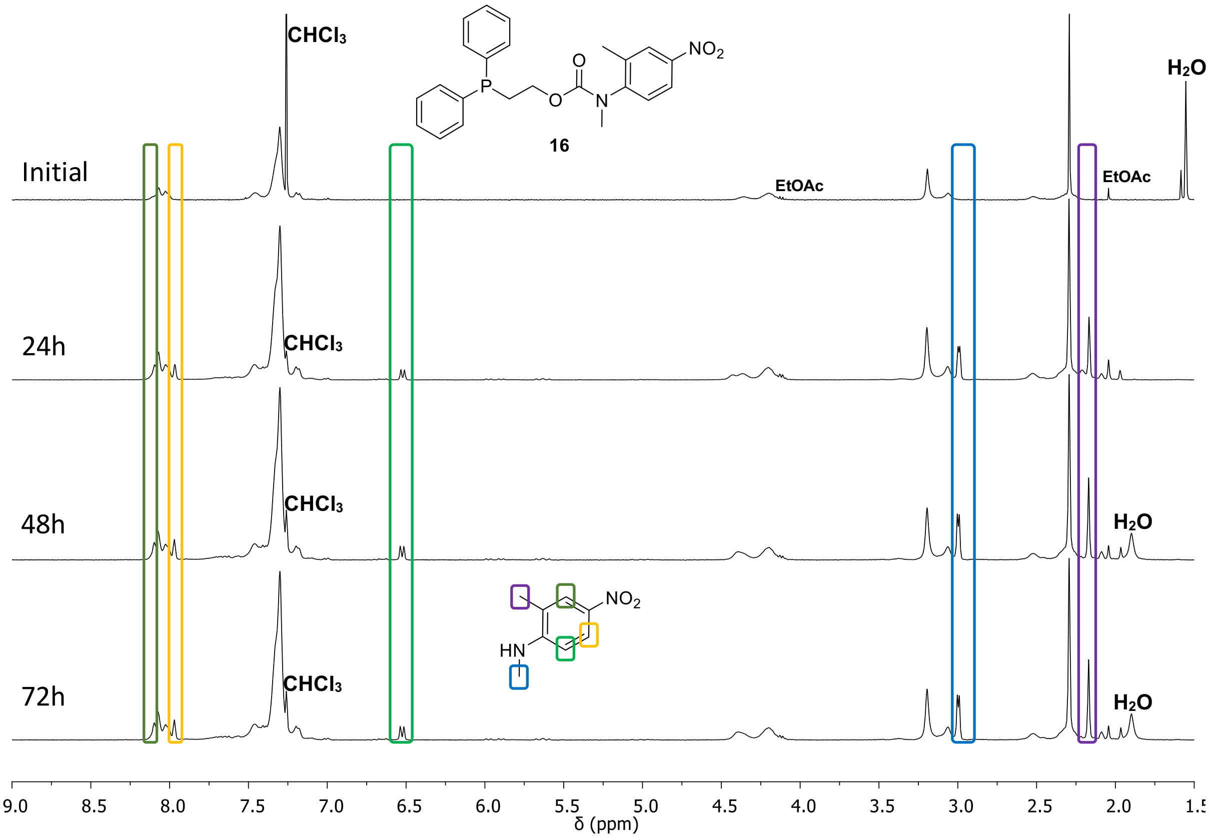

Figure S88. Stacked ^{1}H NMR spectra of **16** ($CDCl_3$, 400 MHz) over 72 hours at room temperature under *vacuum*, as a neat liquid.

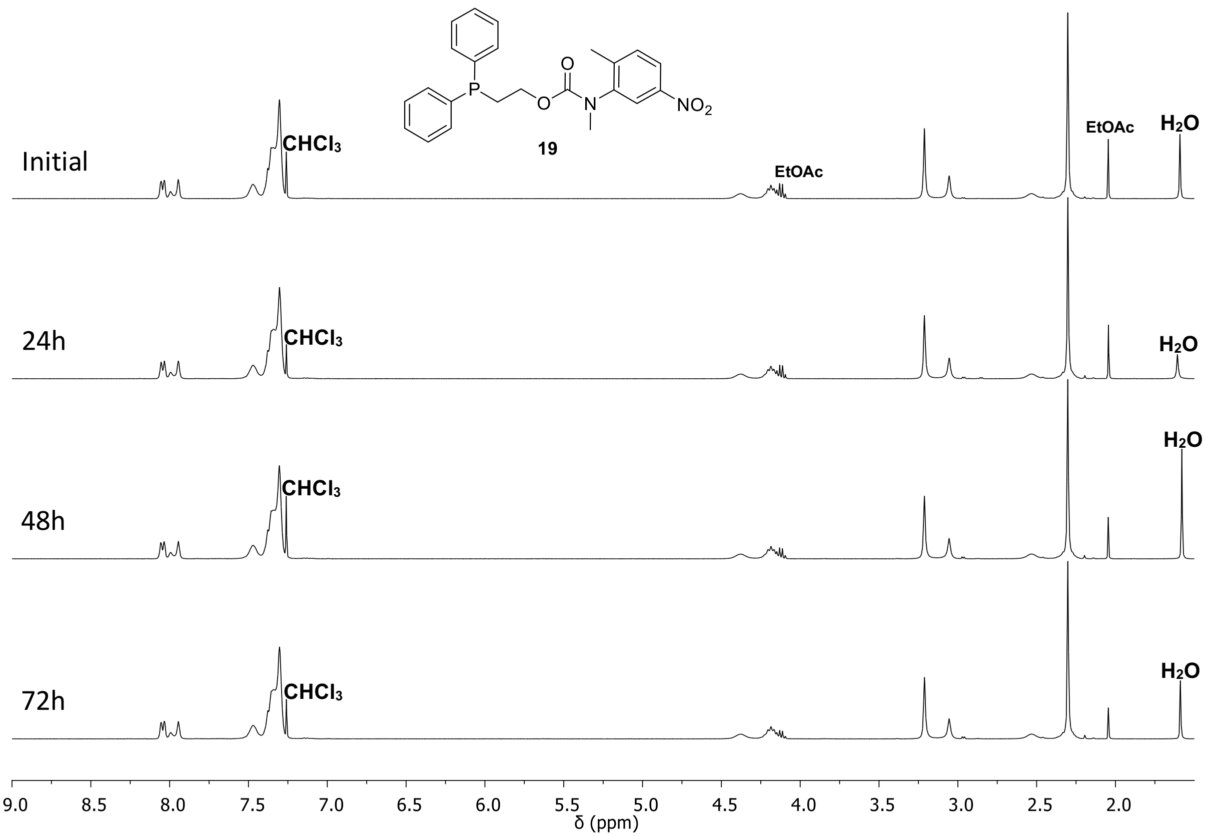

Figure S89. Stacked ^{1}H NMR spectra of **19** ($CDCl_3$, 400 MHz) over 72 hours at room temperature under *vacuum*, as a neat liquid.

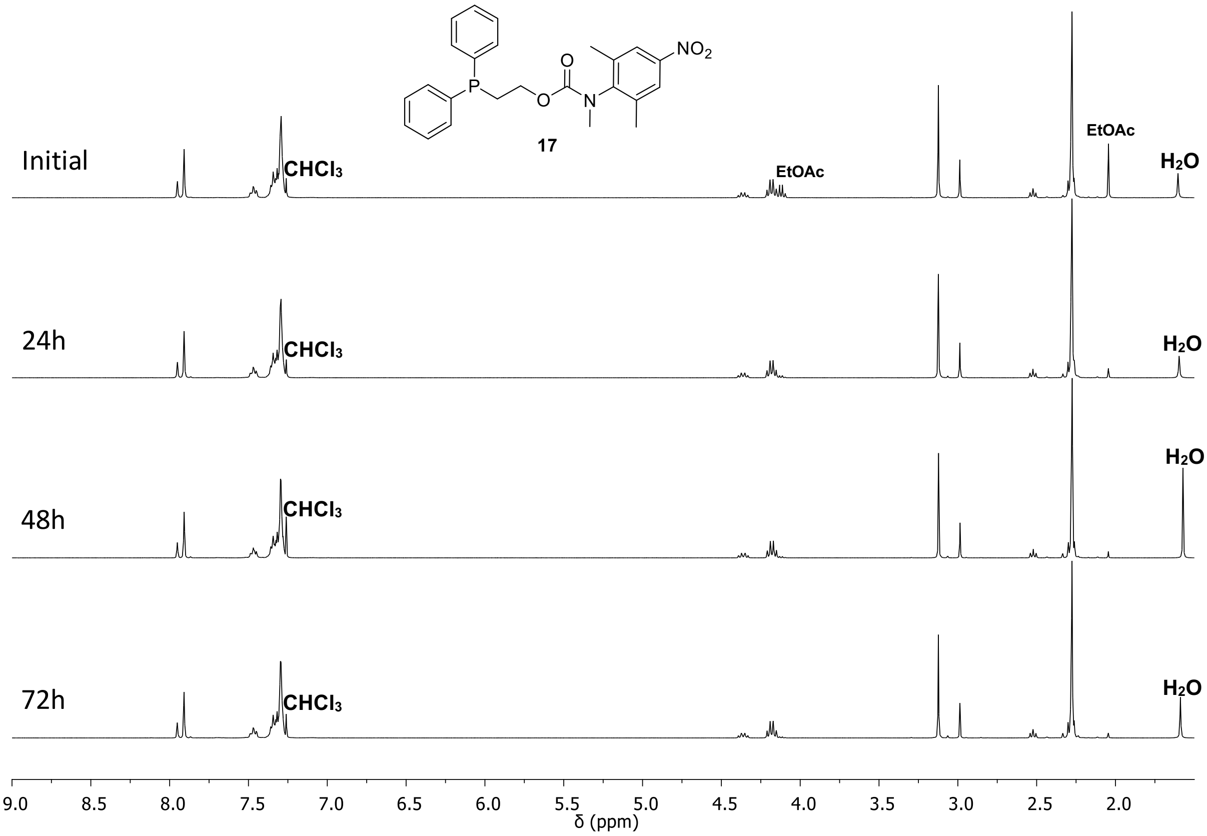

Figure S90. Stacked ^{1}H NMR spectra of **17** ($CDCl_3$, 400 MHz) over 72 hours at room temperature under *vacuum*, as a neat liquid.

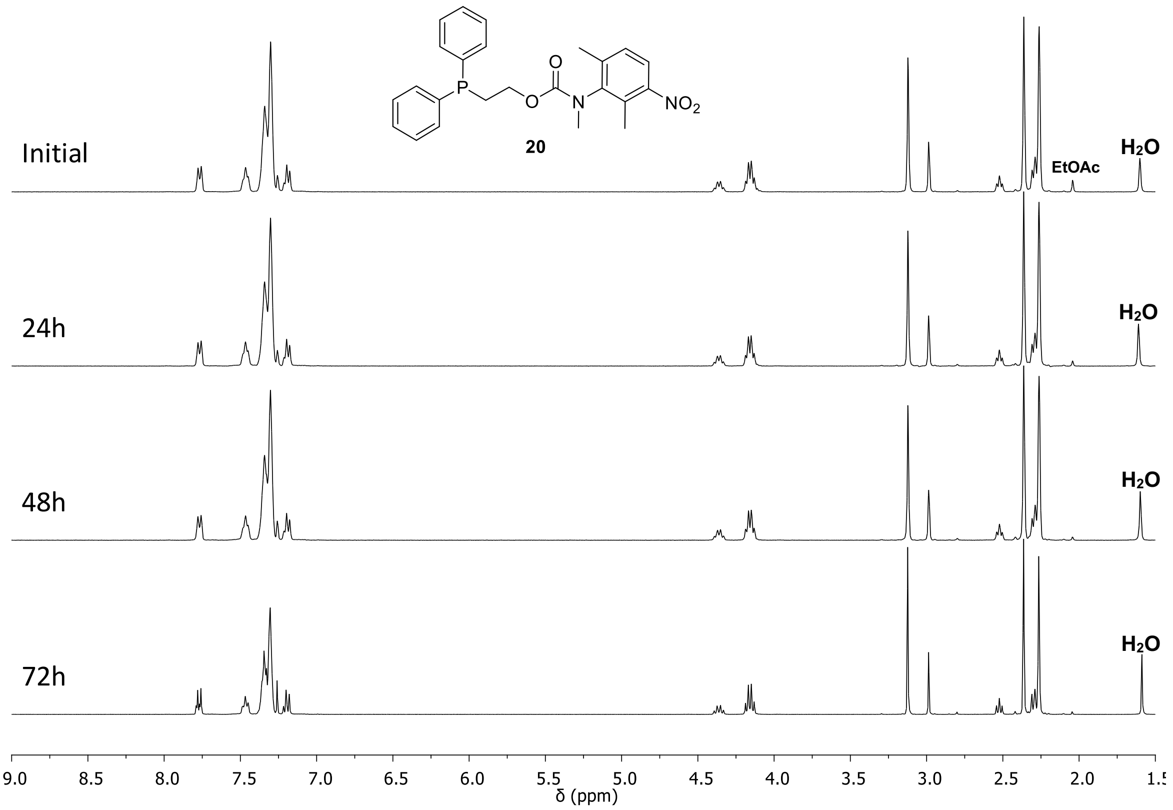

Figure S91. Stacked ^{1}H NMR spectra of **20** ($CDCl_3$, 400 MHz) over 72 hours at room temperature under *vacuum*, as a neat liquid.

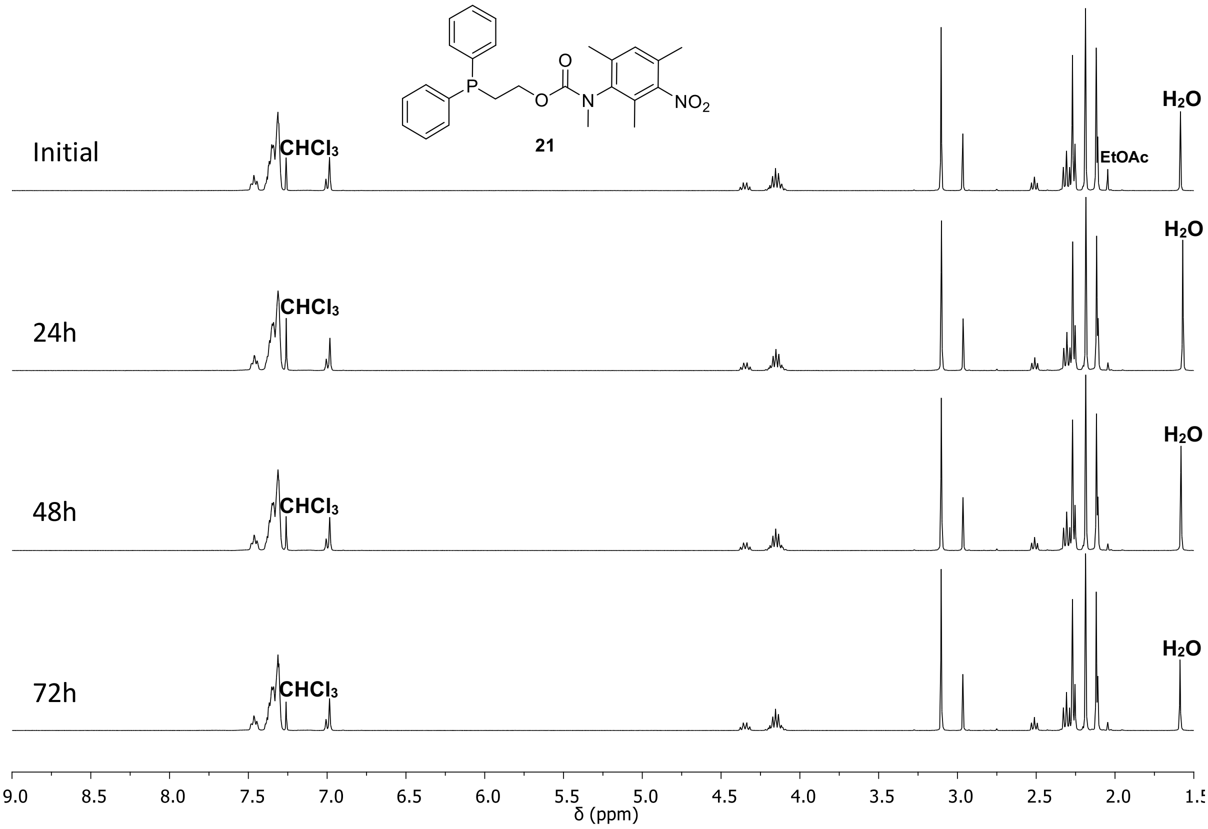

Figure S92. Stacked ^{1}H NMR spectra of **21** ($CDCl_3$, 400 MHz) over 72 hours at room temperature under *vacuum*, as a neat liquid.

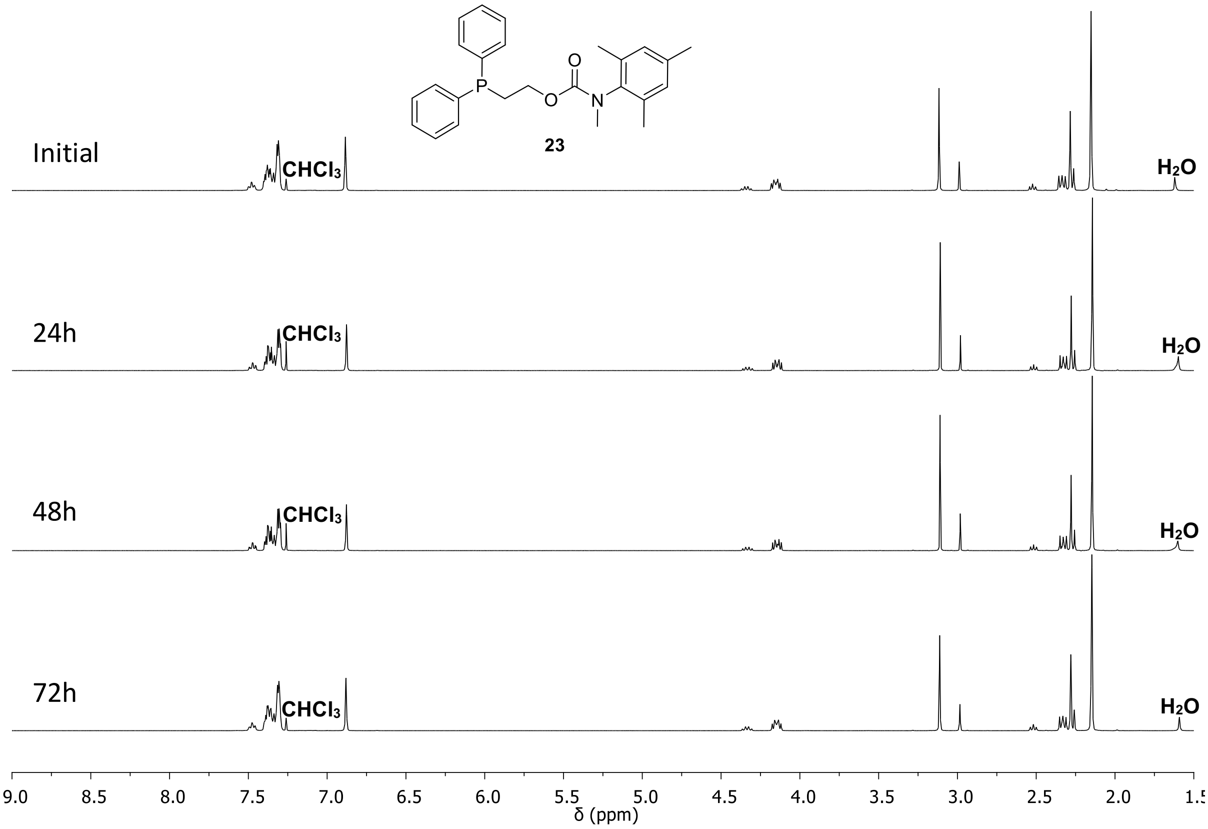

Figure S93. Stacked ^{1}H NMR spectra of **23** ($CDCl_3$, 400 MHz) over 72 hours at room temperature under *vacuum*, as a neat solid.

NMR Characterization of crystalline model carbamate compounds 24-28

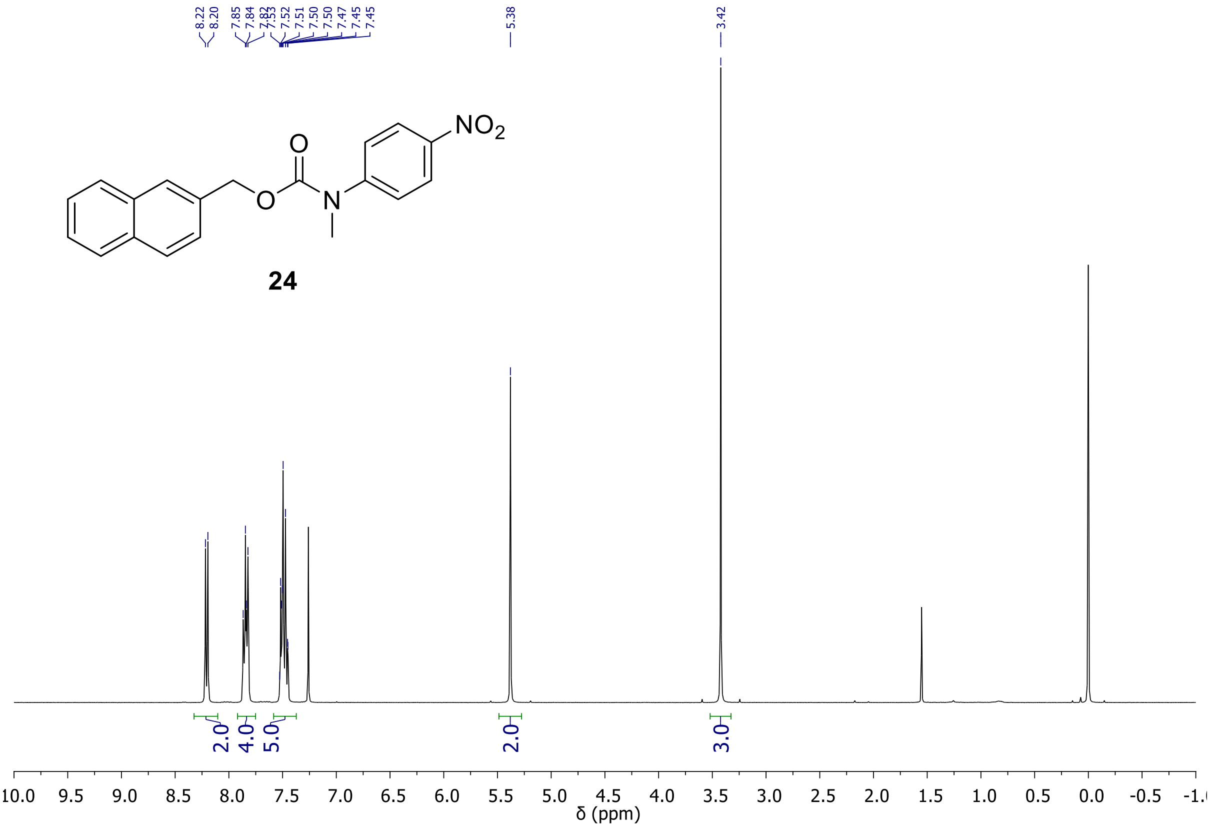

Figure S94. ^{1}H NMR spectra of **24** ($CDCl_3$, 400 MHz).

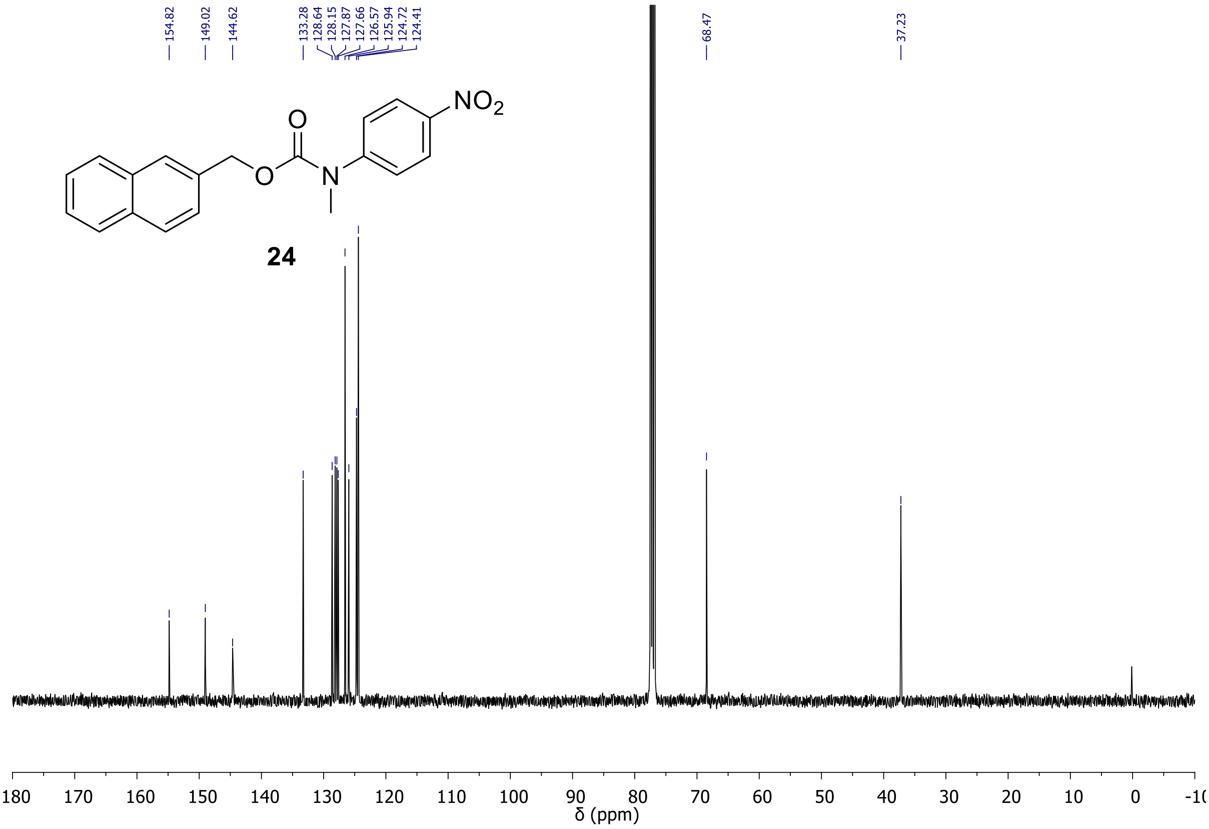

Figure S95. $^{13}C\{^{1}H\}$ NMR spectra of **24** ($CDCl_3$, 100 MHz).

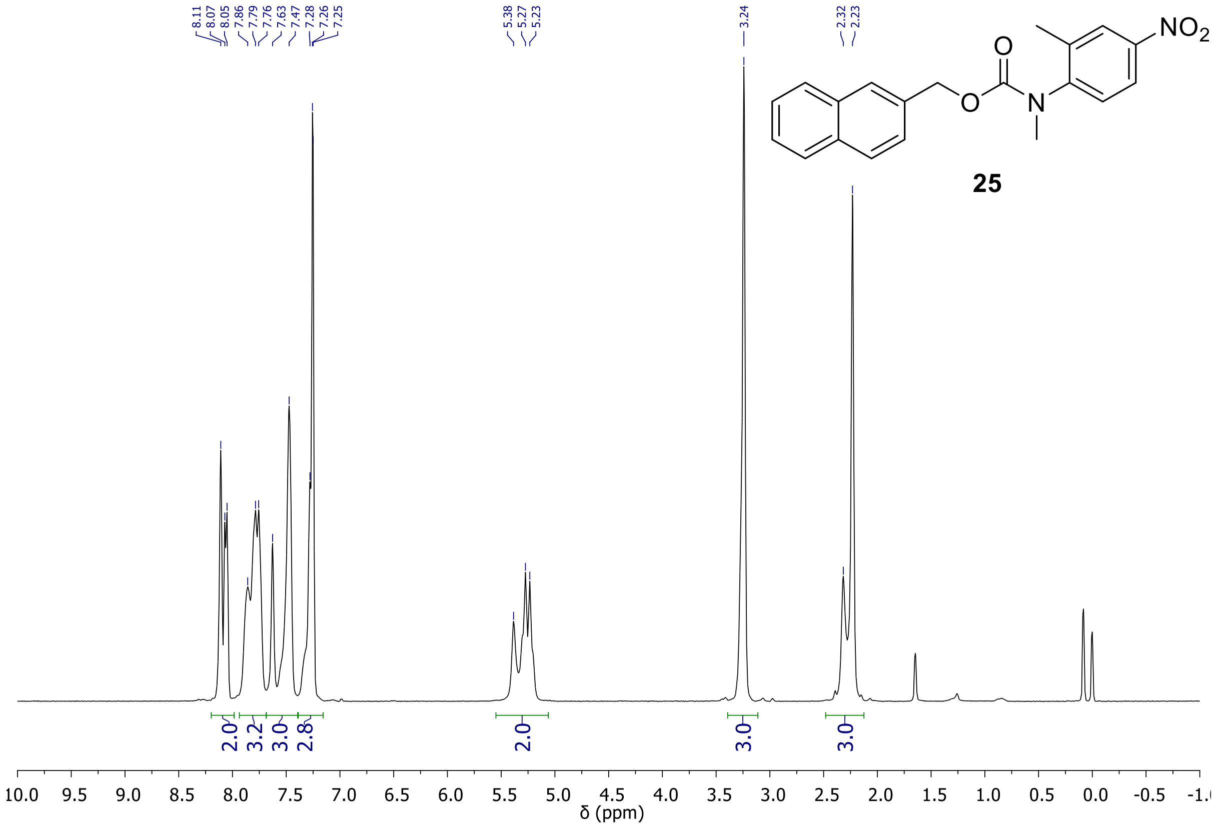

Figure S96. ^{1}H NMR spectra of **25** ($CDCl_3$, 400 MHz).

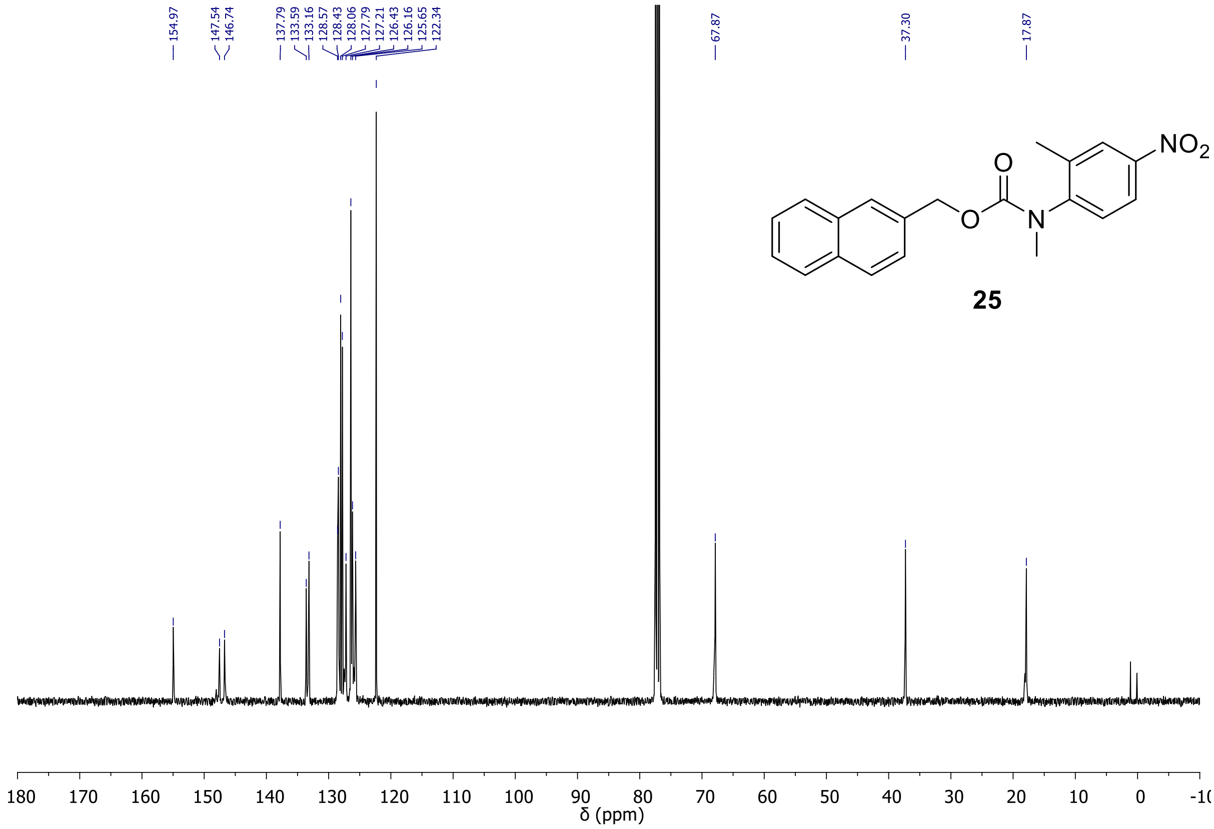

Figure S97. $^{13}C\{^{1}H\}$ NMR spectra of **25** ($CDCl_3$, 100 MHz).

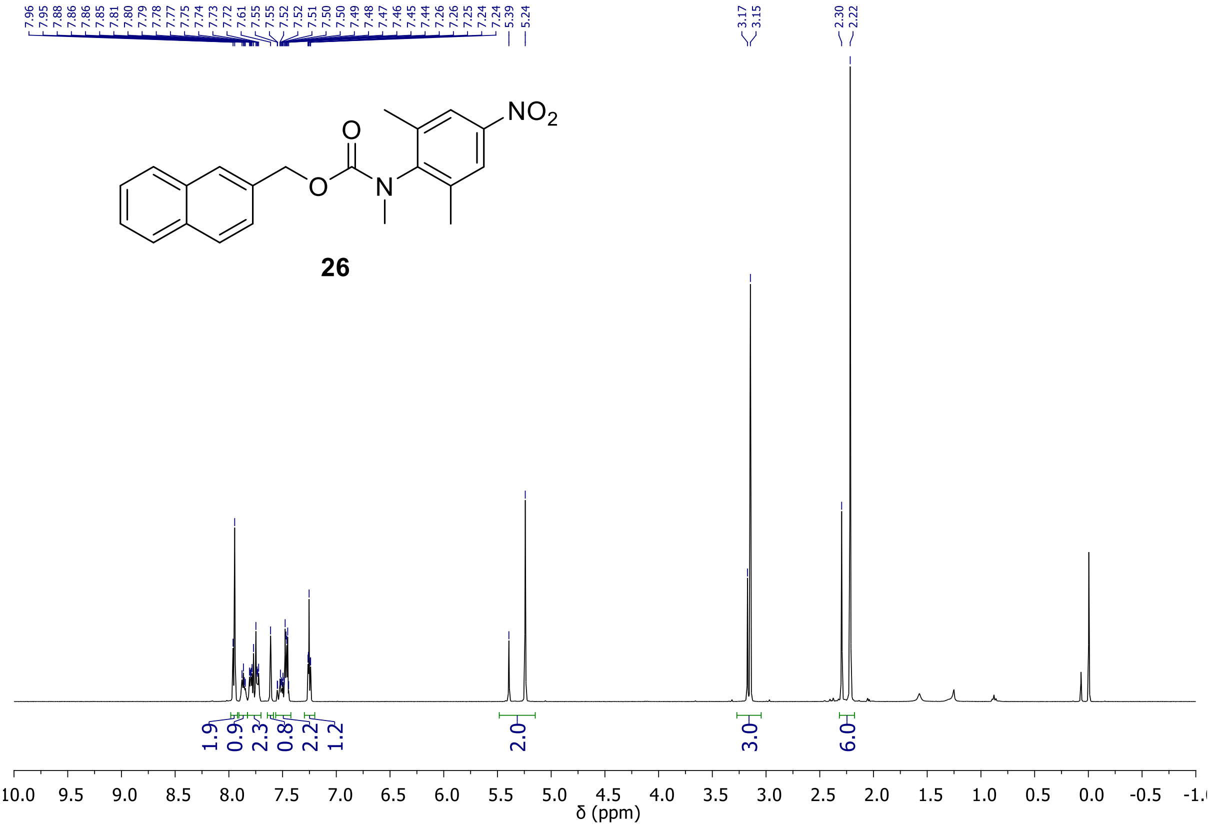

Figure S98. ^{1}H NMR spectra of **26** ($CDCl_3$, 400 MHz).

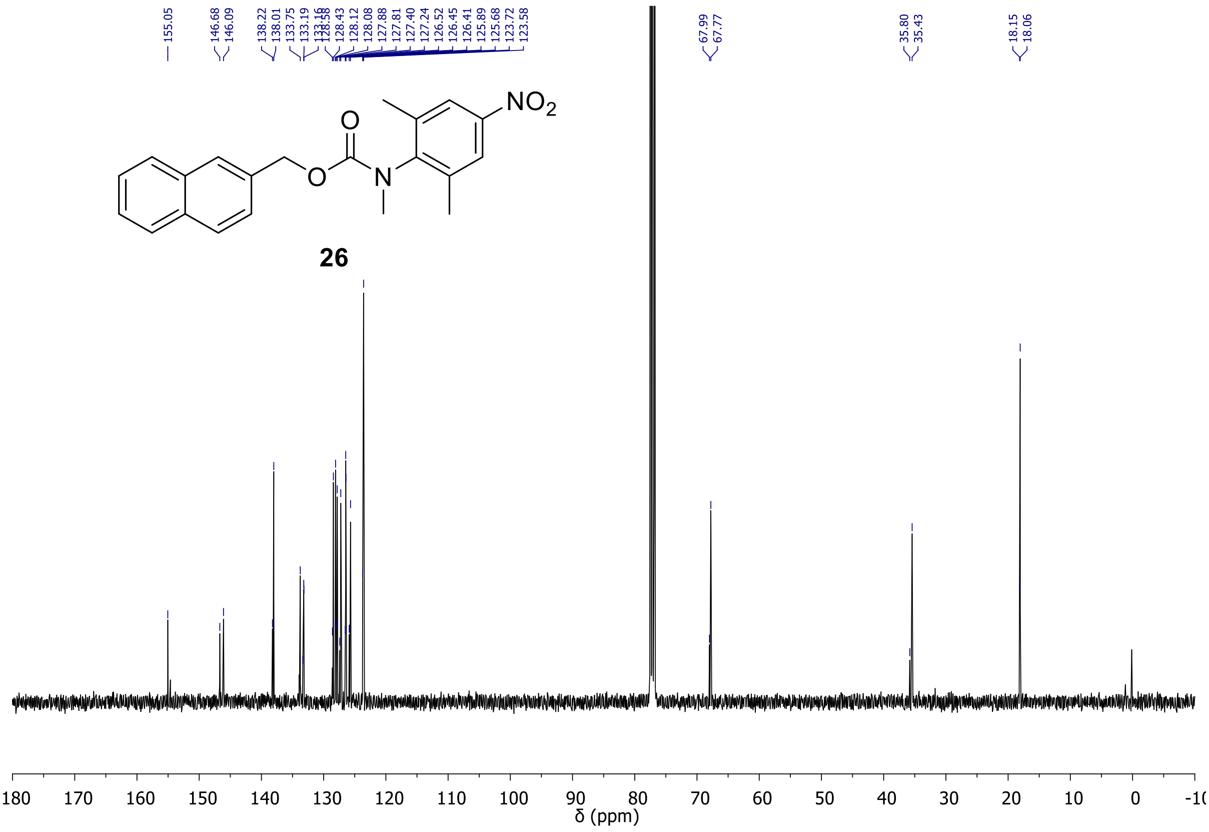

Figure S99. $^{13}C\{^1H\}$ NMR spectra of **26** ($CDCl_3$, 100 MHz).

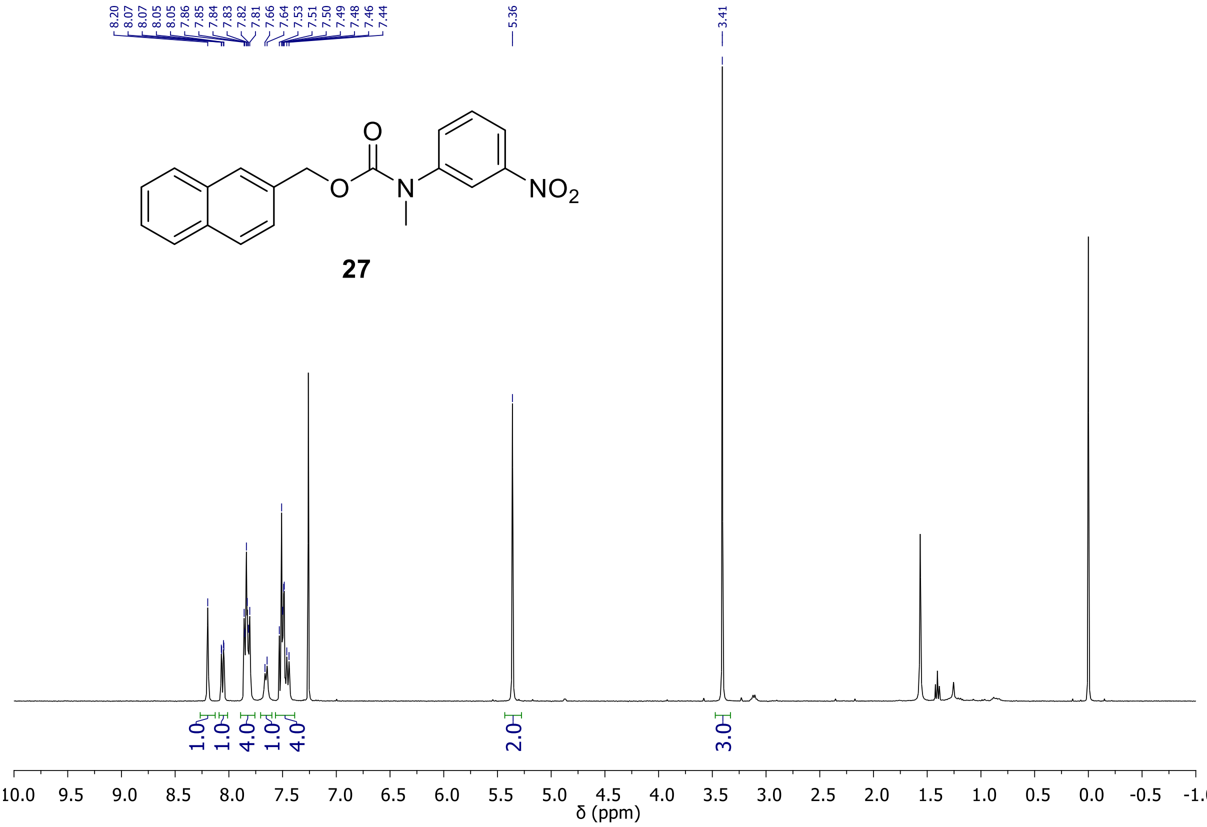

Figure S100. ^{1}H NMR spectra of **27** ($CDCl_3$, 400 MHz).

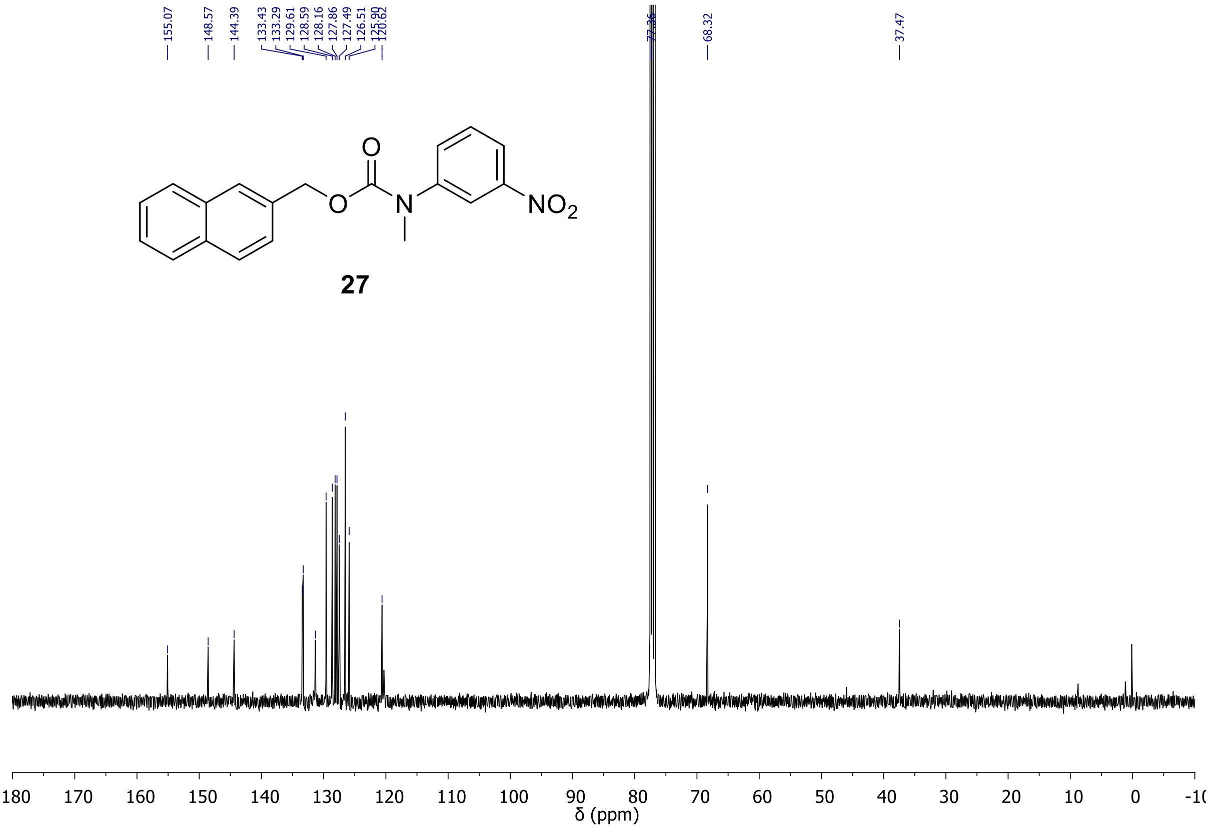

Figure S101. $^{13}C\{^{1}H\}$ NMR spectra of **27** ($CDCl_3$, 100 MHz).

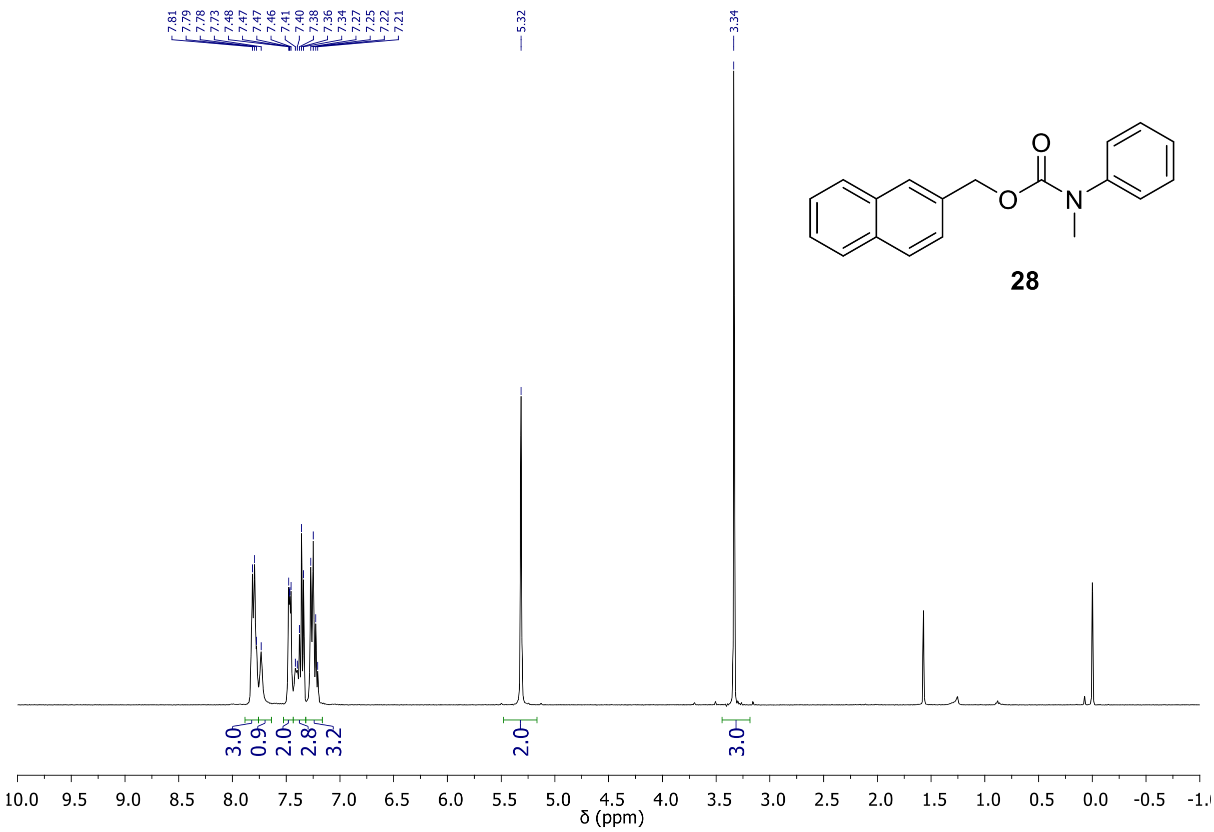

Figure S102. ^{1}H NMR spectra of **28** ($CDCl_3$, 400 MHz).

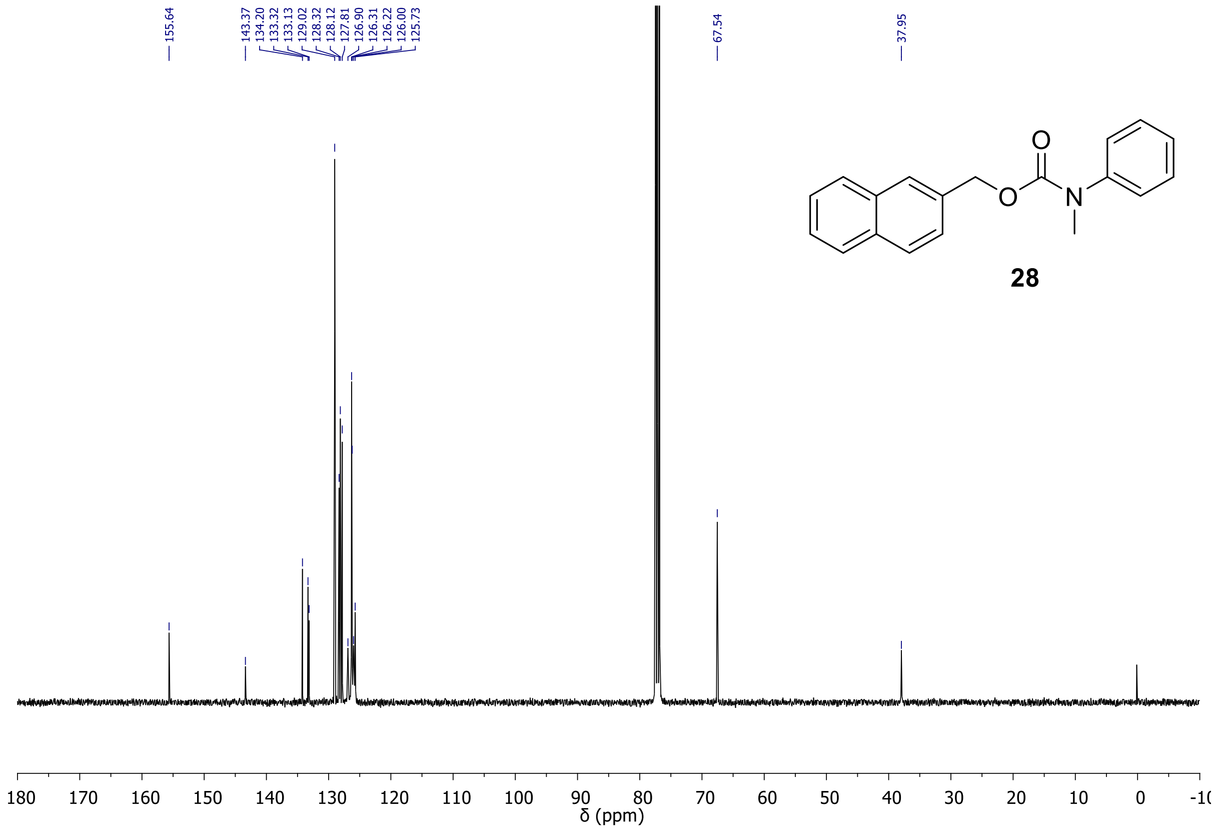

Figure S103. $^{13}C\{^{1}H\}$ NMR spectra of **28** ($CDCl_3$, 100 MHz).

X-ray characterization of crystalline compounds 22-28

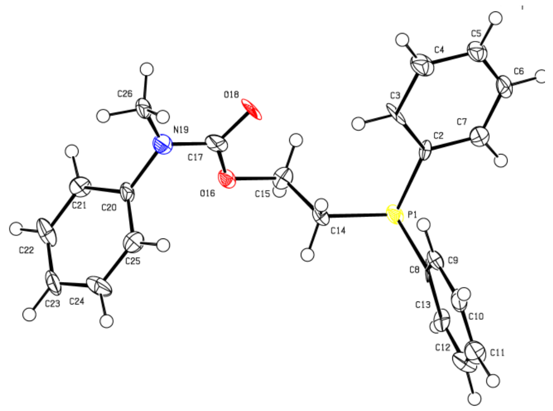

Figure S104. The solid-state structure of **22** obtained from single-crystal X-ray diffraction analysis. The thermal ellipsoids are shown at 50 % probability.

Table S2. Crystallographic data of **22**.

Formula	$C_{22}H_{22}NO_2P$
*M*r	363.38
Crystal system	orthorhombic
Space group	*P n a 2₁*
Z	4
a /Å	16.86700(2)
b/Å	15.46610(2)
c /Å	7.28360(2)
α / °	90
β / °	90
γ / °	90
V / Å^3	1900.05(1)
D_{calc} / g cm^{-3}	1.270
Crystal habit	Colourless block
Crystal dimensions /mm	0.10 x 0.35 x 0.45
Radiation	Cu K_α (1.54184 Å)
T /K	100
μ /mm^{-1}	1.401
R(*F*), *Rw*(*F*)	7.48, 10.46
CCDC cif deposition number	CCDC 1980913

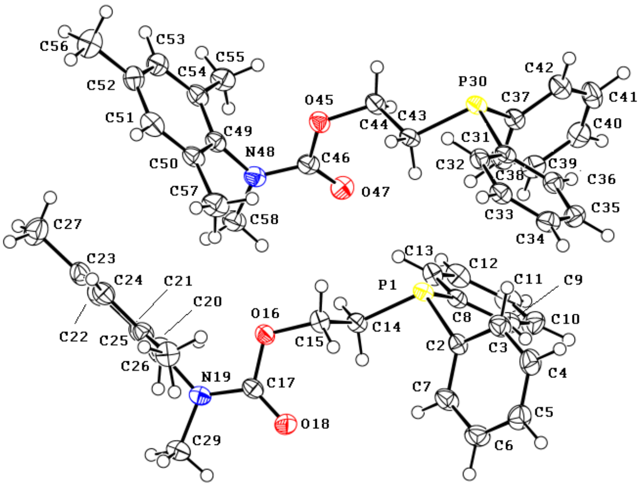

Figure S105. The solid-state structure of **23** obtained from single-crystal X-ray diffraction analysis. The thermal ellipsoids are shown at 50 % probability.

Table S3. Crystallographic data of **23**.

Formula	$C_{25}\ H_{28}\ N_1\ O_2\ P_1$
M_r	405.48
Crystal system	triclinic
Space group	*P* -1
Z	4
a /Å	10.05838(2)
b/Å	13.64351(2)
c /Å	16.41625(2)
α / °	77.189(3)
β / °	84.474(3)
γ / °	89.116(3)
V / Å^3	2186.50(3)
D_{calc} / g cm^{-3}	1.232
Crystal habit	Colourless rod
Crystal dimensions /mm	0.019 × 0.044 × 0.132
Radiation	Cu K_α (1.54184 Å)
T /K	100
μ /mm^{-1}	1.267
R(*F*), *Rw*(*F*)	5.71, 7.65
CCDC cif deposition number	CCDC 1962710

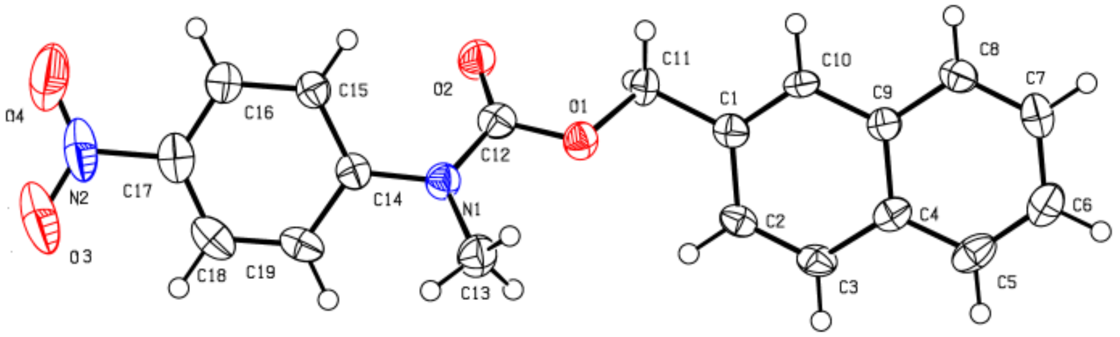

Figure S106. The solid-state structure of **24** obtained from single-crystal X-ray diffraction analysis. The thermal ellipsoids are shown at 50 % probability.

Table S4. Crystallographic data of **24**.

Formula	$C_{19}H_{16}N_2O_4$
M_r	336.34
Crystal system	monoclinic
Space group	*I c*
Z	4
a /Å	7.4743(4)
b/Å	11.6565(4)
c /Å	18.9804(10)
β / °	101.236(5)
V / Å^3	1621.96(13)
D_{calc} / g cm^{-3}	1.377
Crystal habit	Colourless block
Crystal dimensions /mm	0.25 × 0.03 × 0.03
Radiation	Mo K_α (0.71073 Å)
T /K	150
μ /mm^{-1}	0.095
R(*F*), *Rw*(*F*)	6.26, 7.21
CCDC cif deposition number	CCDC 1908144

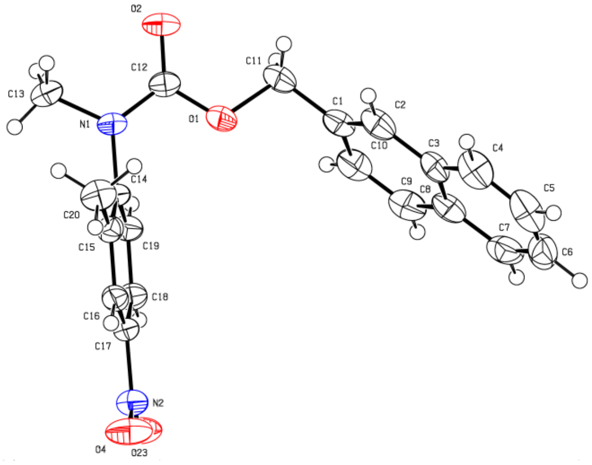

Figure S107. The solid-state structure of **25** obtained from single-crystal X-ray diffraction analysis. The thermal ellipsoids are shown at 50 % probability.

Table S5. Crystallographic data of **25**.

Formula	$C_{20}\,H_{18}\,N_2\,O_4$
M_r	350.37
Crystal system	triclinic
Space group	*P* -1
Z	2
a /Å	7.1068(6)
b/Å	8.0781(6)
c /Å	15.2806(13)
α / °	96.560(6)
β / °	98.545(7)
γ / °	93.192(6)
V / Å^3	859.47(12)
D_{calc} / g cm^{-3}	1.354
Crystal habit	Colourless block
Crystal dimensions /mm	0.08 × 0.15 × 0.23
Radiation	Mo K_α (0.71073 Å)
T /K	150
μ /mm^{-1}	0.096
R(*F*), *Rw*(*F*)	7.30, 8.15
CCDC cif deposition number	CCDC 1908143

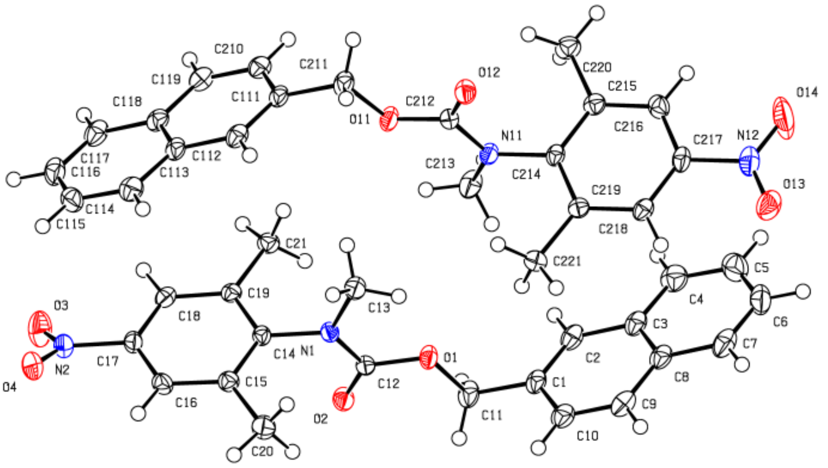

Figure S108. The solid-state structure of **26** obtained from single-crystal X-ray diffraction analysis. The thermal ellipsoids are shown at 50 % probability.

Table S6. Crystallographic data of **26**.

Formula	$C_{21} H_{20} N_2 O_4$
M_r	364.39
Crystal system	triclinic
Space group	*P* -1
Z	4
a /Å	12.0396(1)
b/Å	12.2822(2)
c /Å	13.6060(2)
α / °	65.669(2)
β / °	81.736(1)
γ / °	88.075(1)
V / Å^3	1813.49(5)
D_{calc} / g cm^{-3}	1.335
Crystal habit	Colourless block
Crystal dimensions /mm	0.12 × 0.32 × 0.40
Radiation	Cu K_{α} (1.54184 Å)
T /K	100
μ /mm^{-1}	0.764
R(*F*), *Rw*(*F*)	3.98, 4.20
CCDC cif deposition number	CCDC 1908145

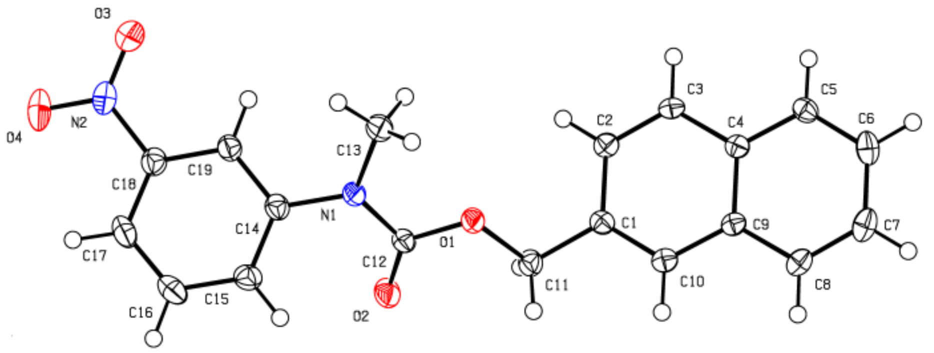

Figure S109. The solid-state structure of **27** obtained from single-crystal X-ray diffraction analysis. The thermal ellipsoids are shown at 50 % probability.

Table S7. Crystallographic data of **27**.

Formula	$C_{19}\,H_{16}\,N_2\,O_4$
M_r	336.34
Crystal system	monoclinic
Space group	$P\,2_1$
Z	2
a /Å	6.3270(1)
b/Å	7.2854(1)
c /Å	16.9650(2)
α / °	90
β / °	94.129(1)
γ / °	90
V / Å^3	779.967(19)
D_{calc} / g cm^{-3}	1.432
Crystal habit	Colourless block
Crystal dimensions /mm	0.05 × 0.16 × 0.24
Radiation	Cu K_α (1.54184 Å)
T /K	100
μ /mm^{-1}	0.841
$R(F)$, $Rw(F)$	3.92, 5.13
CCDC cif deposition number	CCDC 1908146

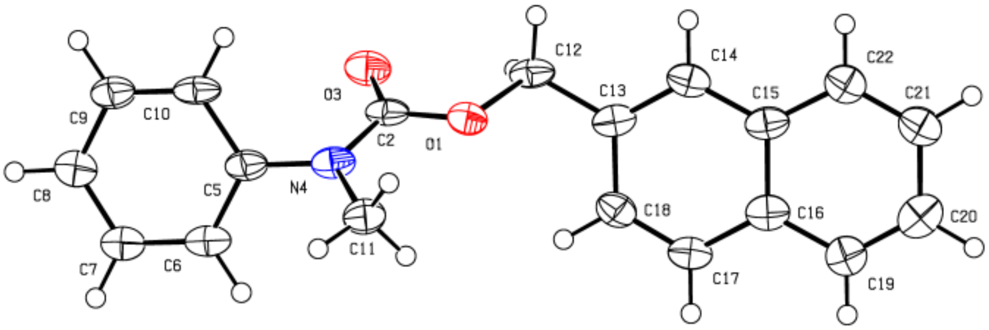

Figure S110. The solid-state structure of **28** obtained from single-crystal X-ray diffraction analysis. The thermal ellipsoids are shown at 50 % probability.

Table S8. Crystallographic data of **28**.

Formula	$C_{19} H_{17} N O_2$
M_r	291.34
Crystal system	monoclinic
Space group	*P* c
Z	2
a /Å	16.46267(4)
b/Å	5.75787(2)
c /Å	7.77104(3)
α / °	90
β / °	102.141(5)
γ / °	90
V / Å^3	720.141(14)
D_{calc} / g cm^{-3}	1.344
Crystal habit	Colourless block
Crystal dimensions /mm	0.03 x 0.06 x 0.14
Radiation	Cu $K_α$ (1.54184 Å)
T /K	100
μ /mm^{-1}	0.694
R(*F*), *Rw*(*F*)	7.14, 9.32
CCDC cif deposition number	CCDC 1980598

Alkylation data for self-immolative system 15-23.

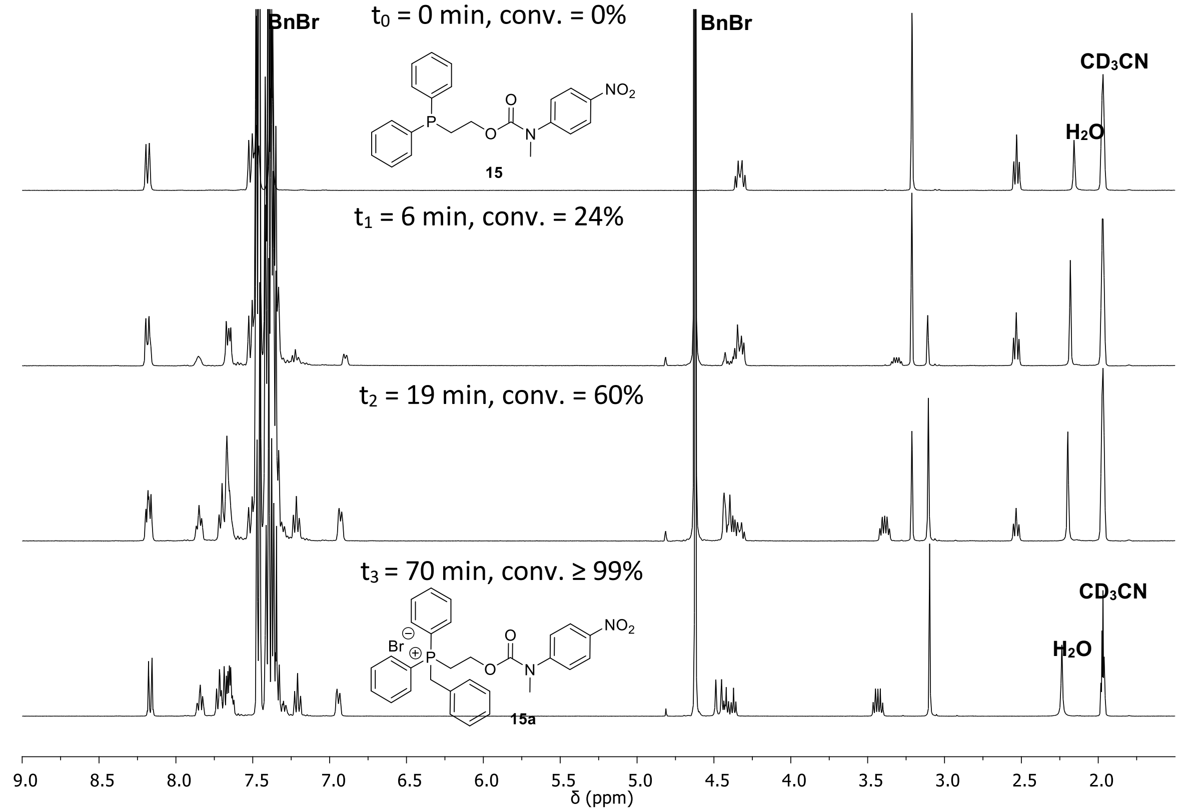

Figure S111. ^{1}H NMR spectra recorded overtime following the addition of 10 equivalents of BnBr to a solution of **15** (MeCN-d_3, 400 MHz).

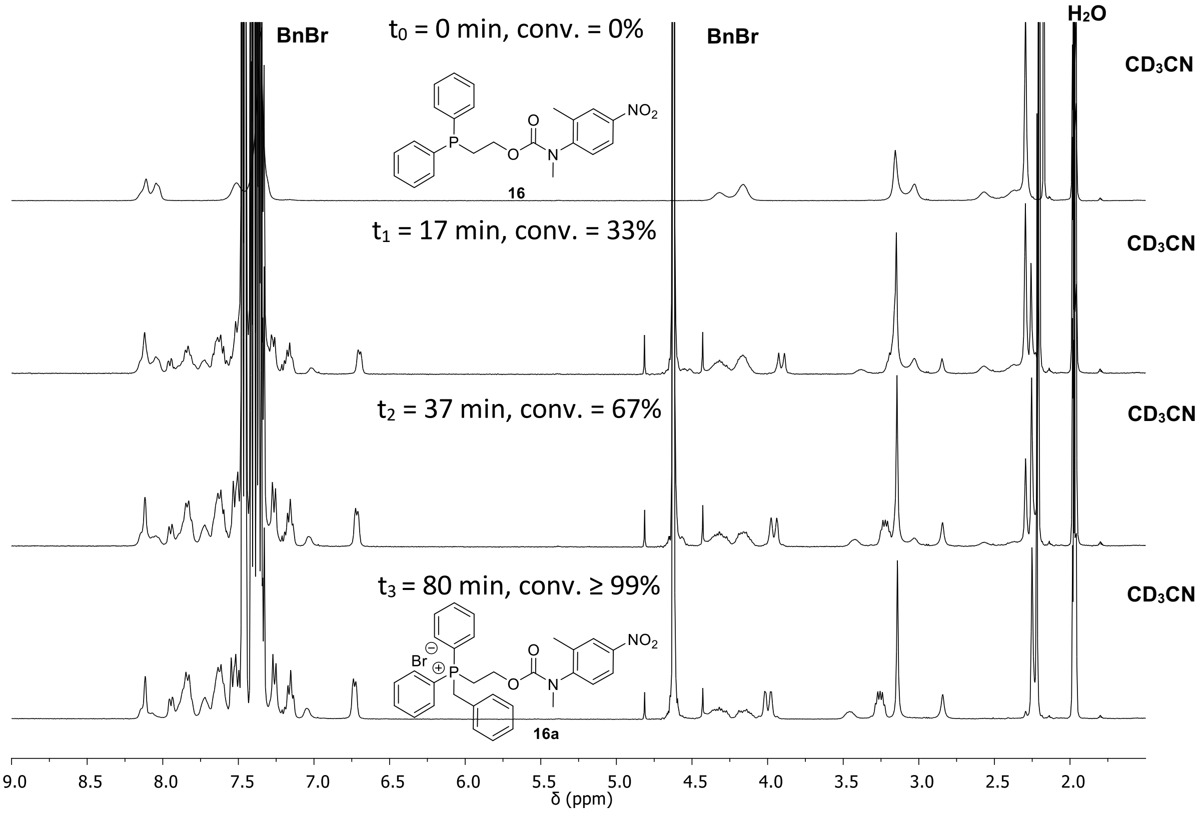

Figure S112. ^{1}H NMR spectra recorded overtime following the addition of 10 equivalents of BnBr to a solution of **16** (MeCN-d_3, 400 MHz).

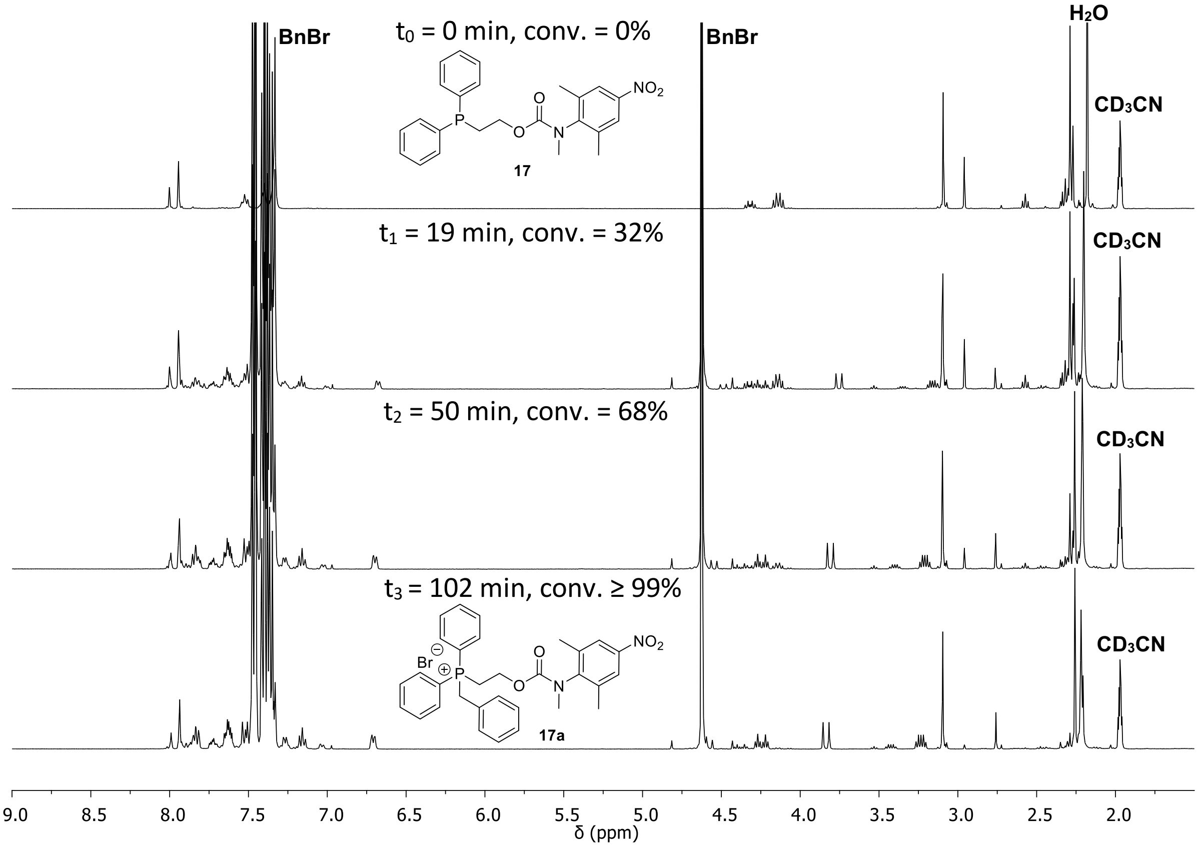

Figure S113. ^{1}H NMR spectra recorded overtime following the addition of 10 equivalents of BnBr to a solution of **17** (MeCN-d_3, 400 MHz).

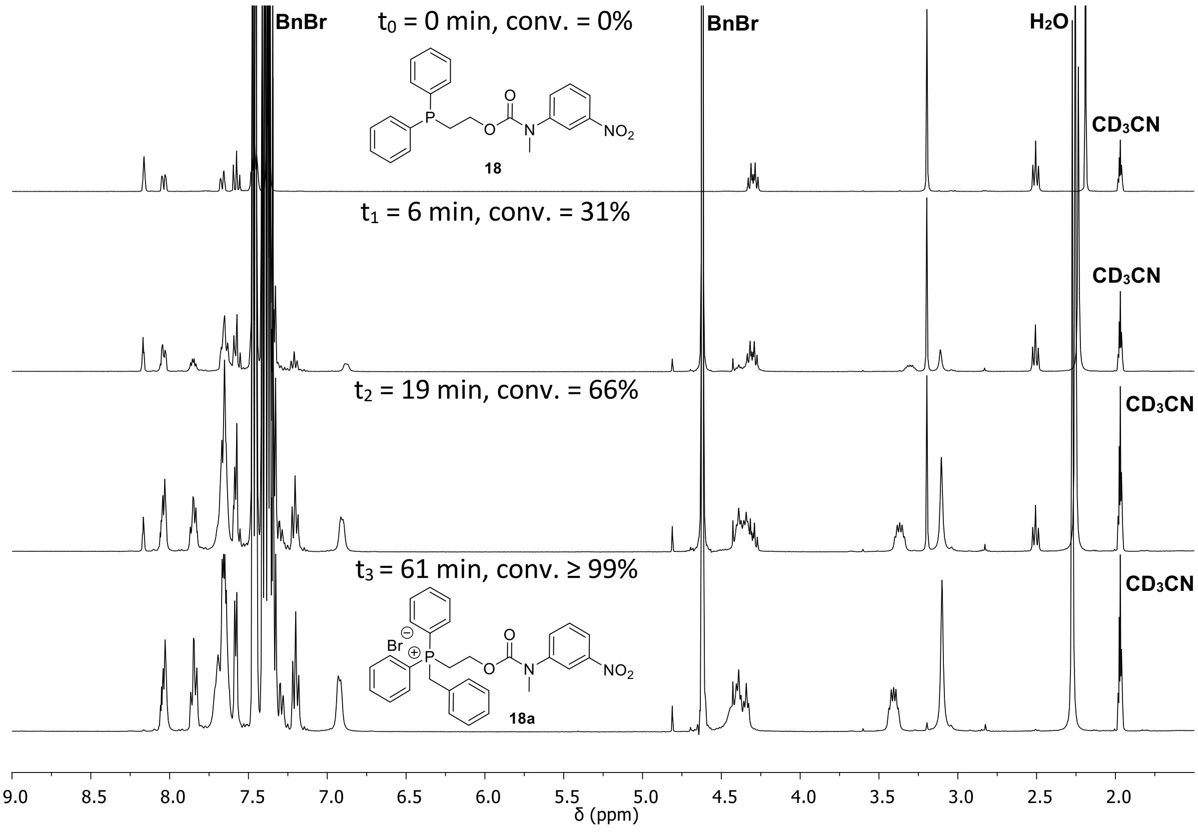

Figure S114. ^{1}H NMR spectra recorded overtime following the addition of 10 equivalents of BnBr to a solution of **18** (MeCN-d_3, 400 MHz).

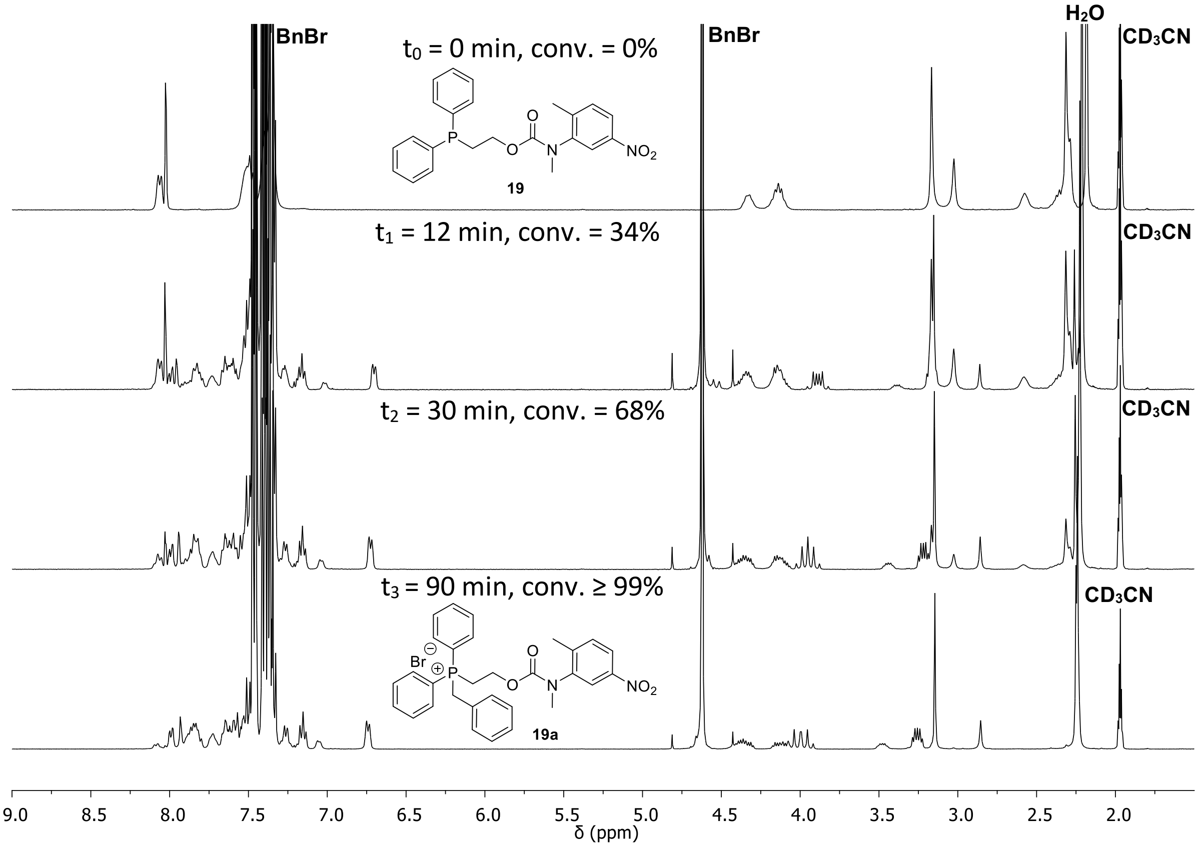

Figure S115. ^{1}H NMR spectra recorded overtime following the addition of 10 equivalents of BnBr to a solution of **19** (MeCN-d_3, 400 MHz).

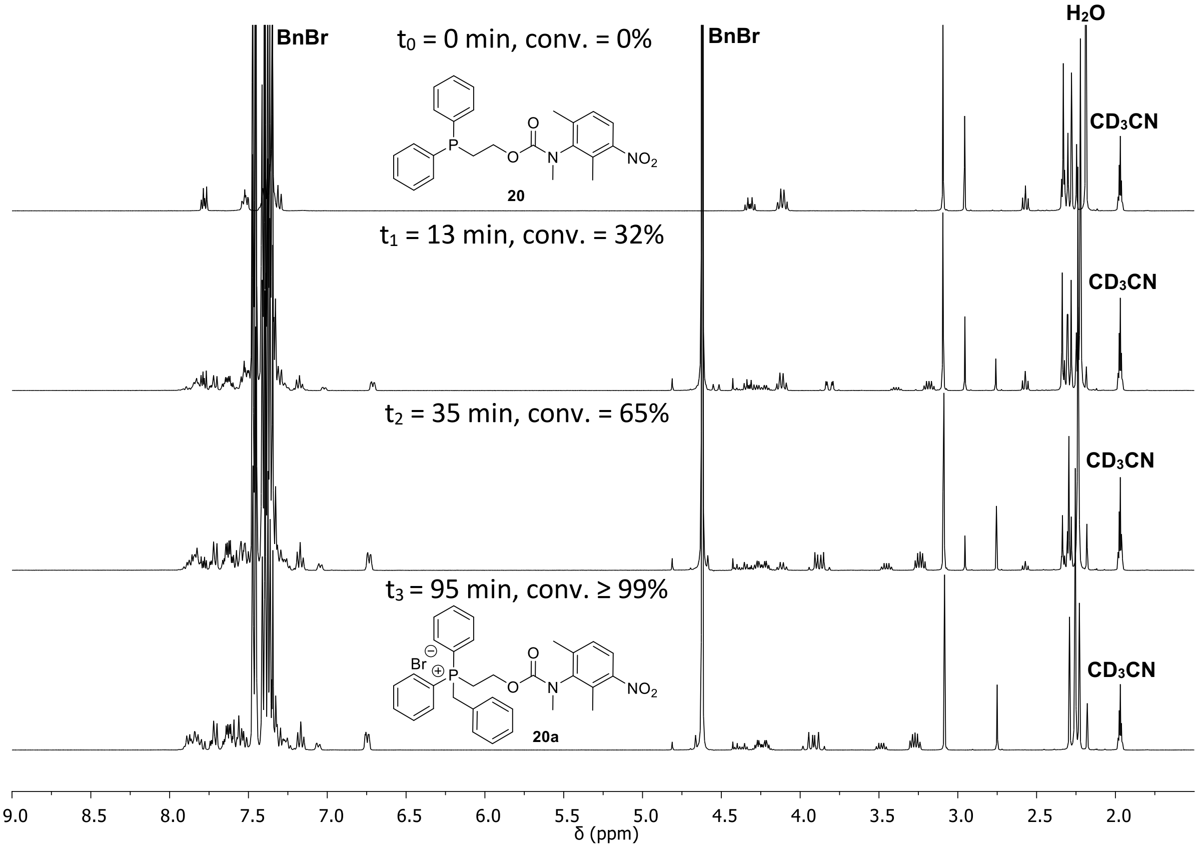

Figure S116. ^{1}H NMR spectra recorded overtime following the addition of 10 equivalents of BnBr to a solution of **20** (MeCN-d_3, 400 MHz)

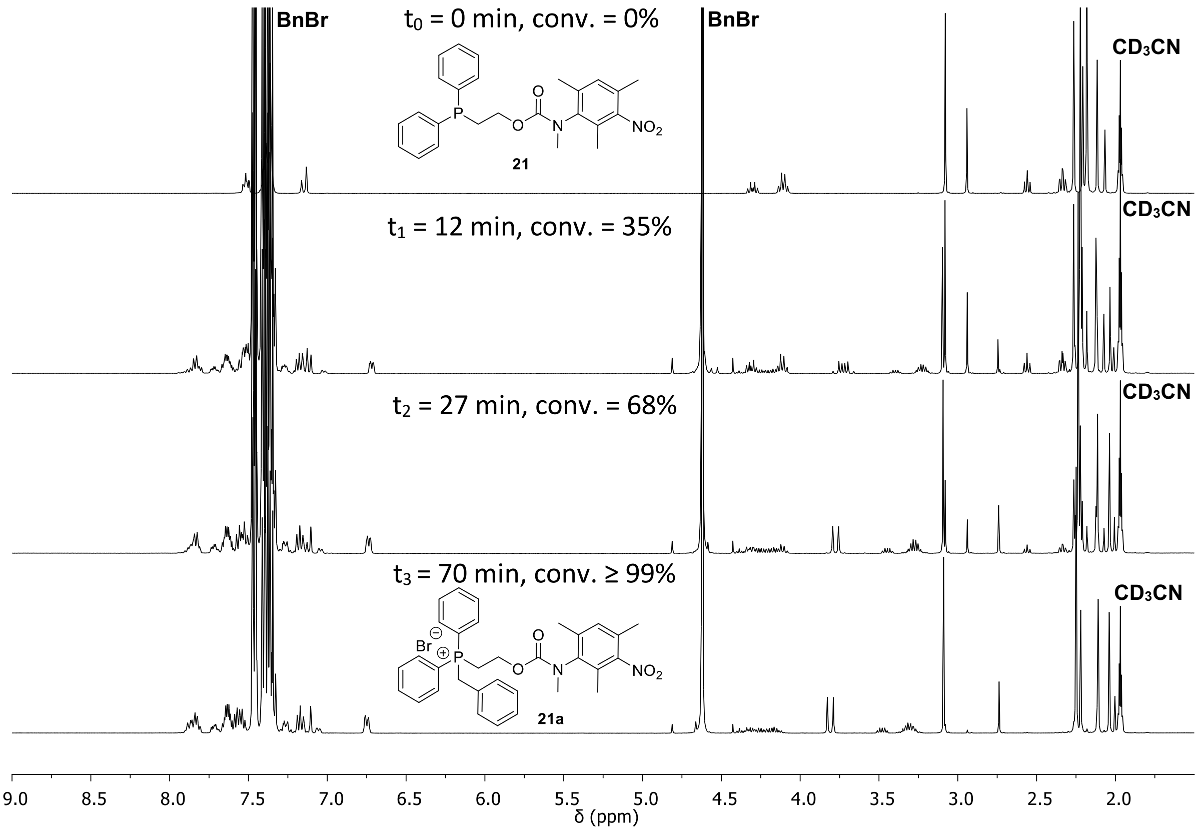

Figure S117. ^{1}H NMR spectra recorded overtime following the addition of 10 equivalents of BnBr to a solution of **21** (MeCN-d_3, 400 MHz).

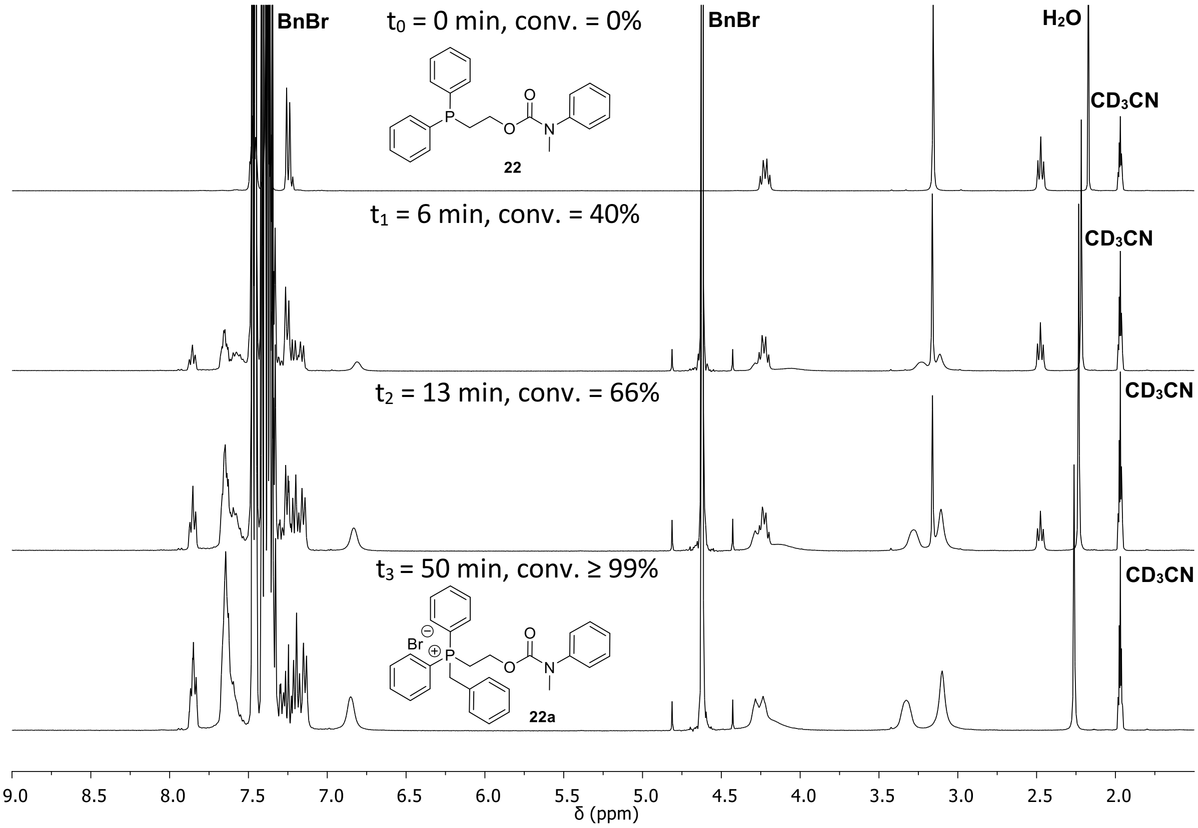

Figure S118. ^{1}H NMR spectra recorded overtime following the addition of 10 equivalents of BnBr to a solution of **22** (MeCN-d_3, 400 MHz).

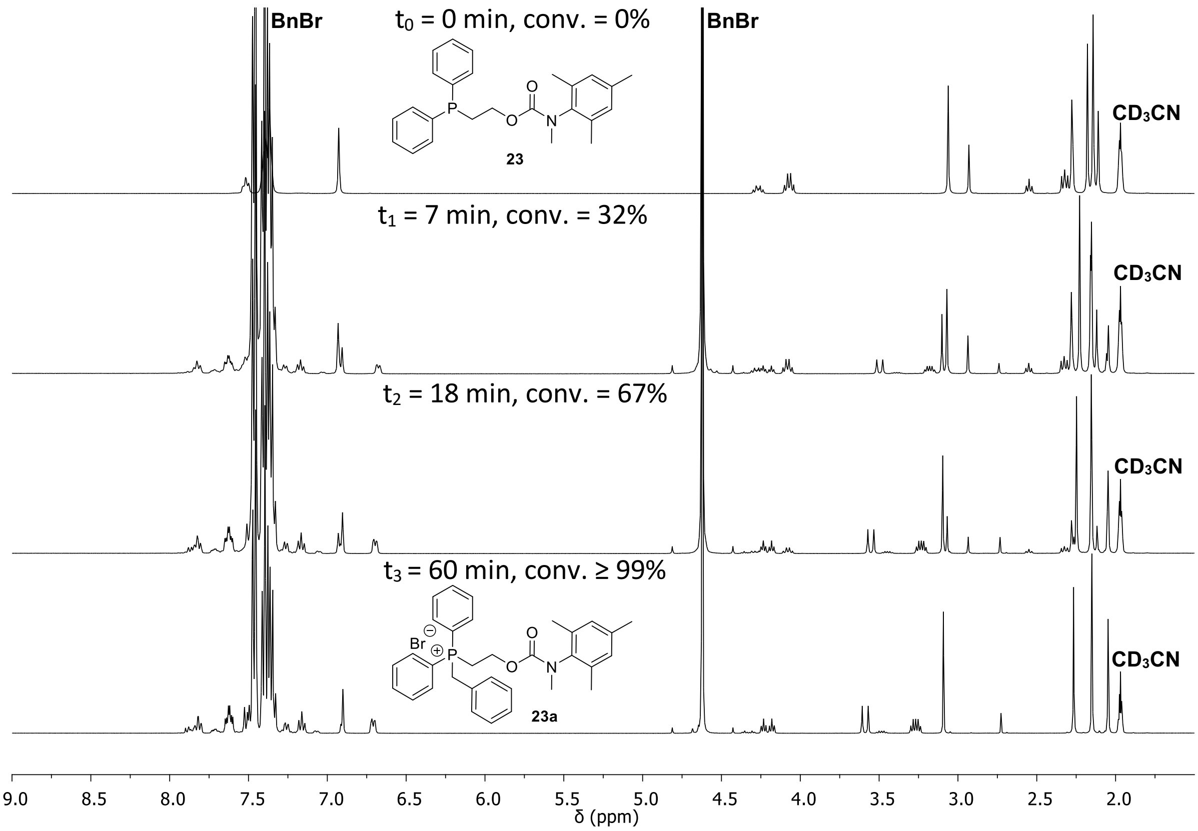

Figure S119. ^{1}H NMR spectra recorded overtime following the addition of 10 equivalents of BnBr to a solution of **23** (MeCN-d_3, 400 MHz).

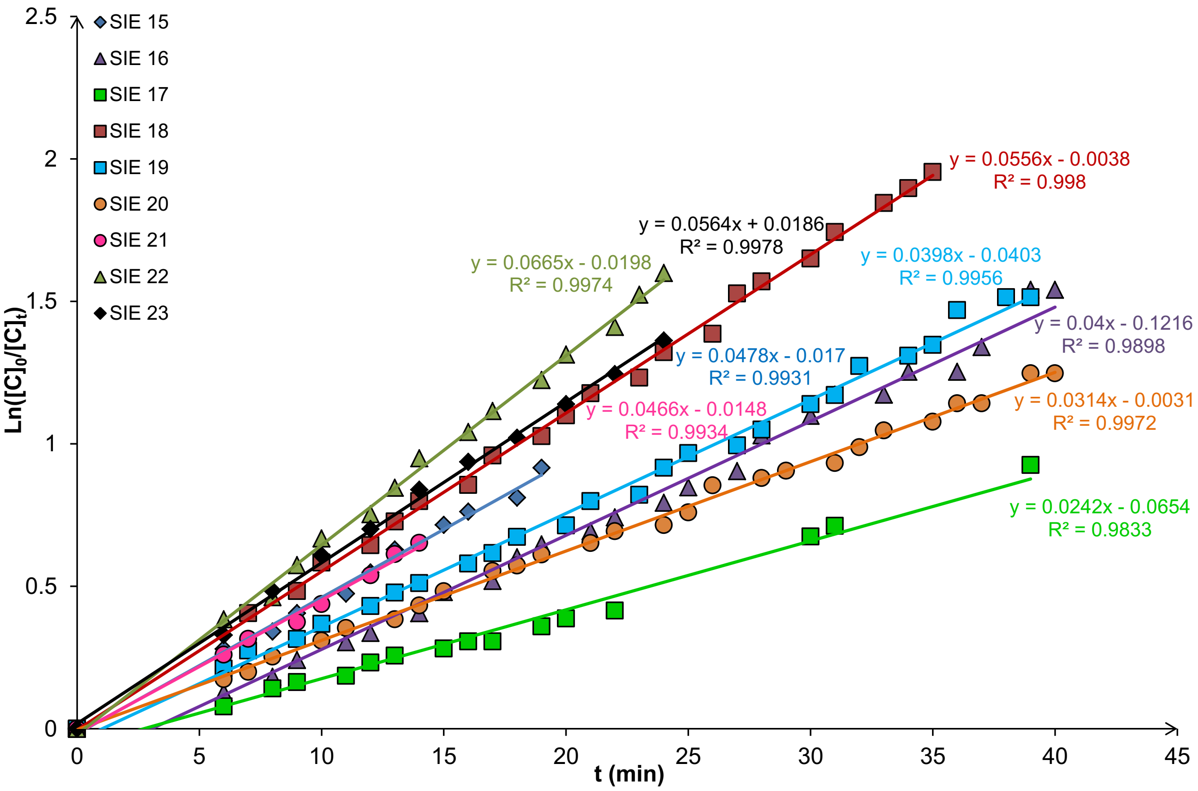

Figure S120. Kinetic plot of ln($[C]_0/[C]_t$) versus reaction time for the alkylation of the self-immolative systems **15-23** in MeCN-d_3 at 20 °C.

β-elimination data for alkylated self-immolative systems 15a-23a

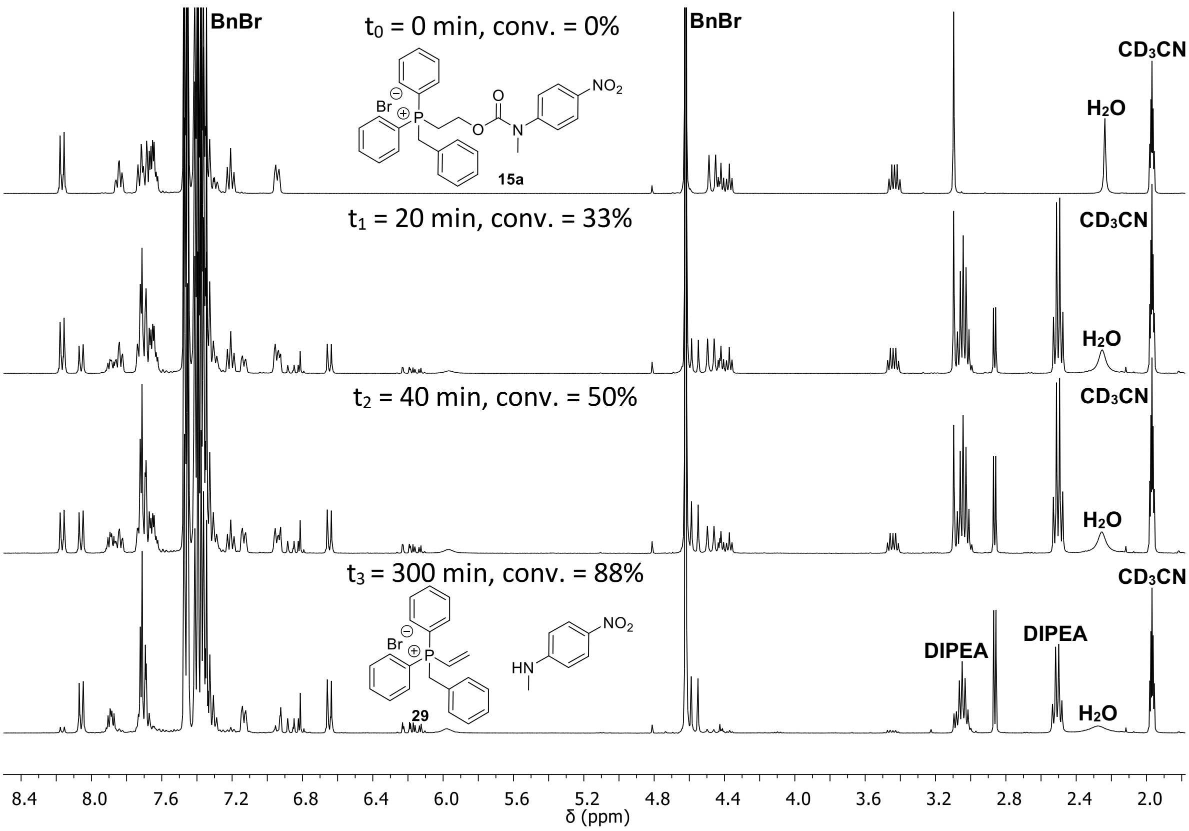

Figure S121. ^{1}H NMR spectra recorded overtime following the addition of 2 equivalents of DIPEA to a solution of alkylated **15** (MeCN-d_3, 400 MHz).

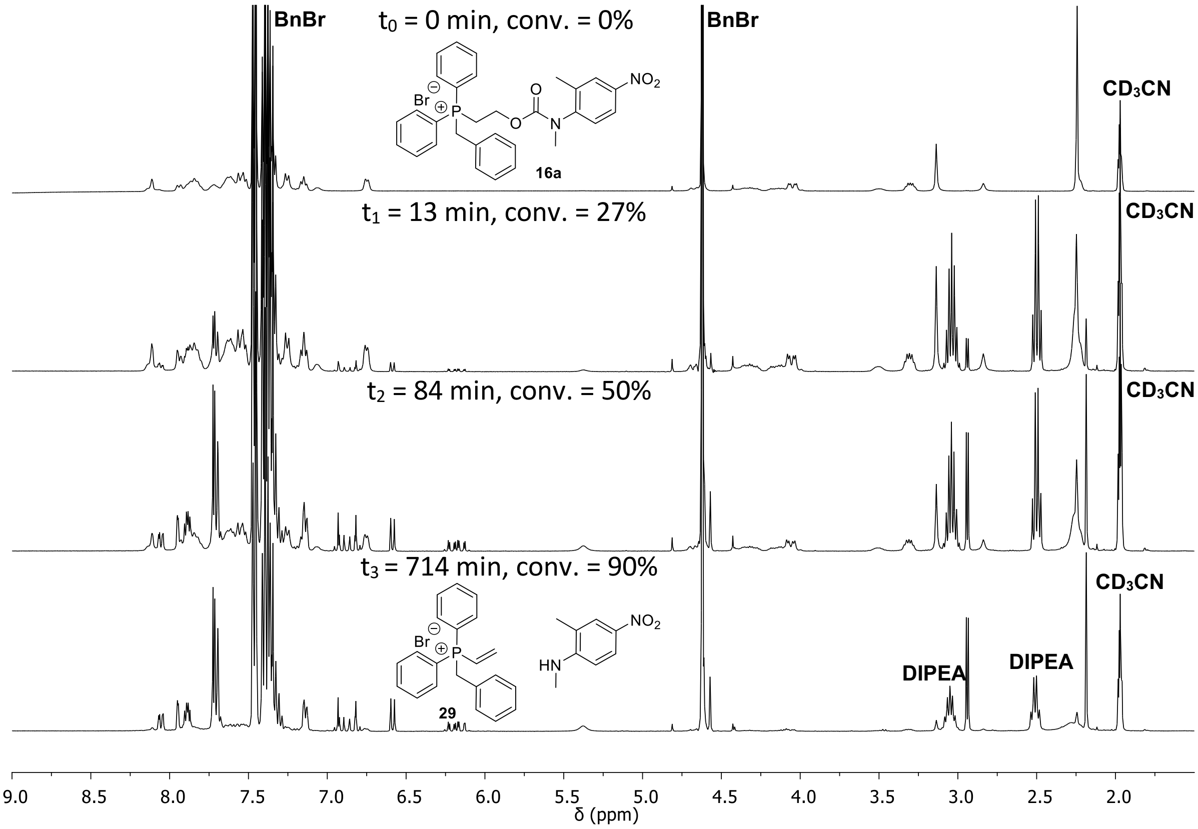

Figure S122. ^{1}H NMR spectra recorded overtime following the addition of 2 equivalents of DIPEA to a solution of alkylated **16** (MeCN-d_3, 400 MHz).

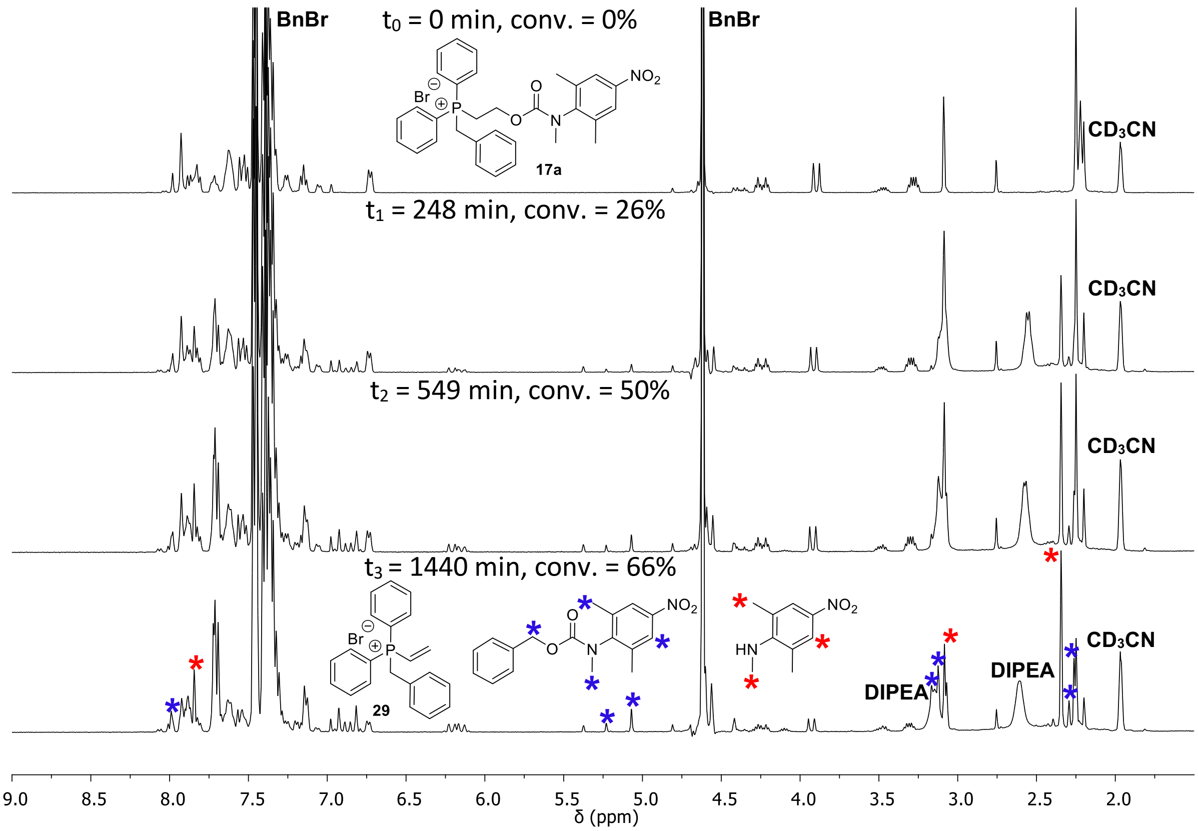

Figure S123. ^{1}H NMR spectra recorded overtime following the addition of 2 equivalents of DIPEA to a solution of alkylated **17** (MeCN-d_3, 400 MHz).

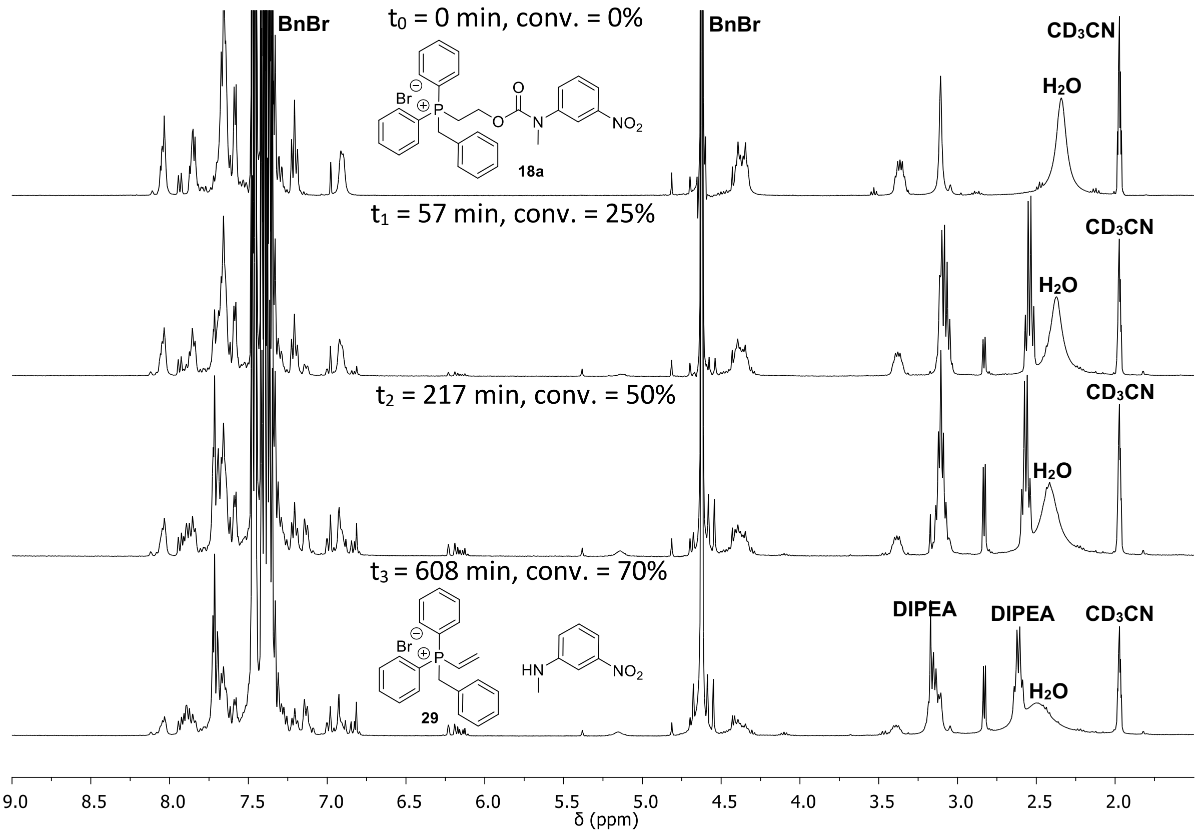

Figure S124. ^{1}H NMR spectra recorded overtime following the addition of 2 equivalents of DIPEA to a solution of alkylated **18** (MeCN-d_3, 400 MHz).

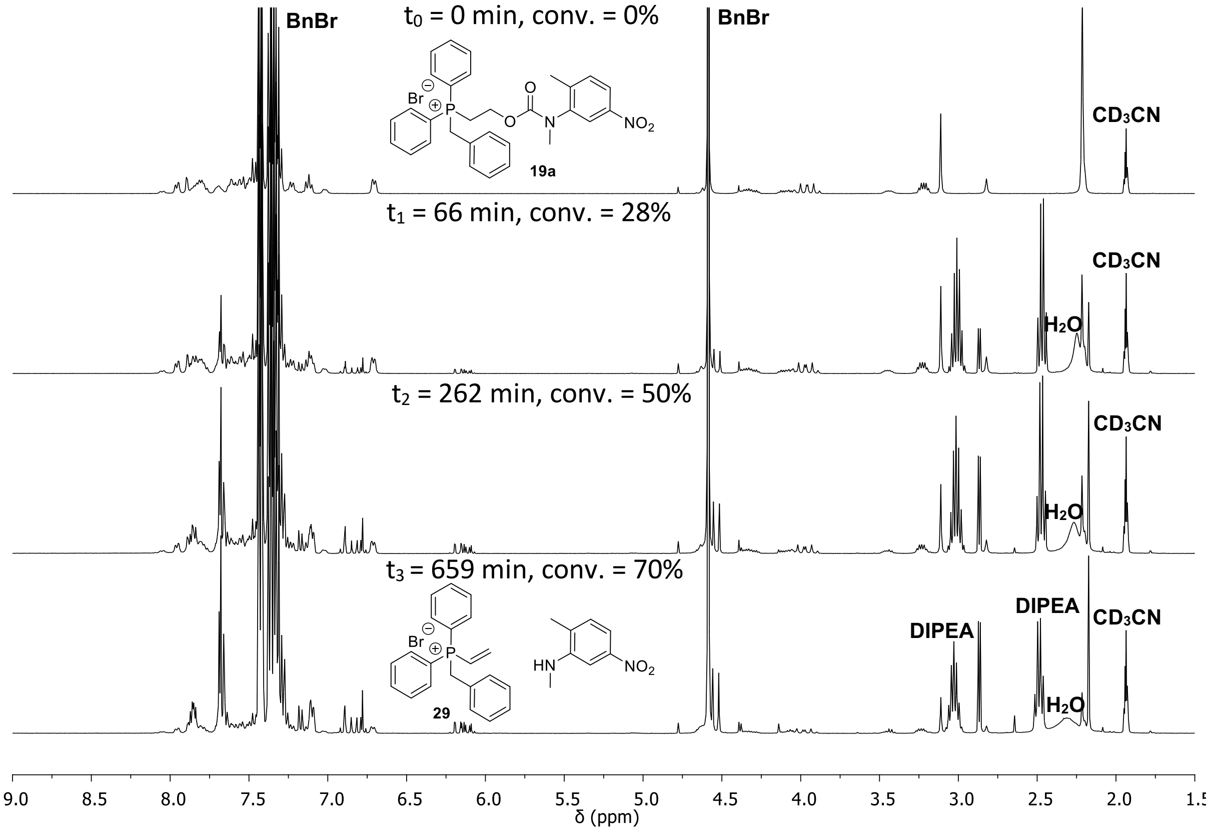

Figure S125. ^{1}H NMR spectra recorded overtime following the addition of 2 equivalents of DIPEA to a solution of alkylated **19** (MeCN-d_3, 400 MHz).

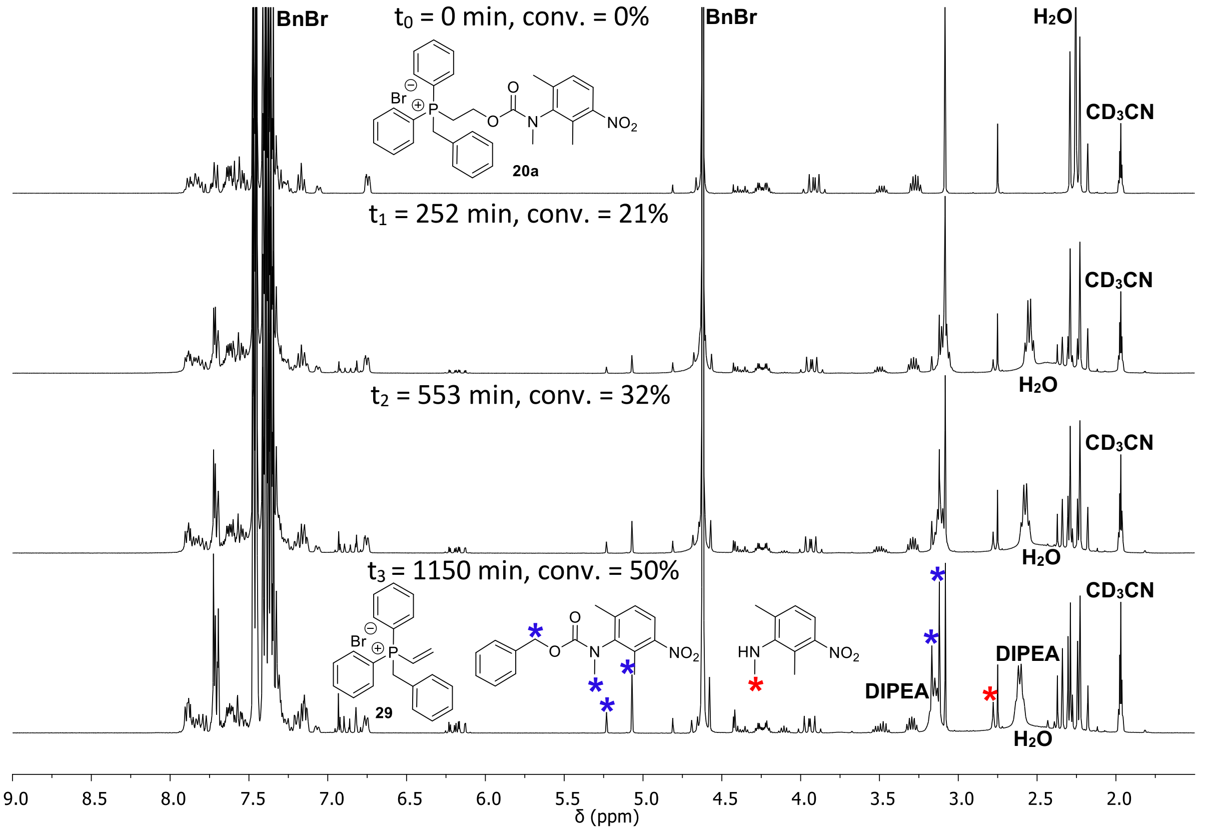

Figure S126. ^{1}H NMR spectra recorded overtime following the addition of 2 equivalents of DIPEA to a solution of alkylated **20** (MeCN-d_3, 400 MHz).

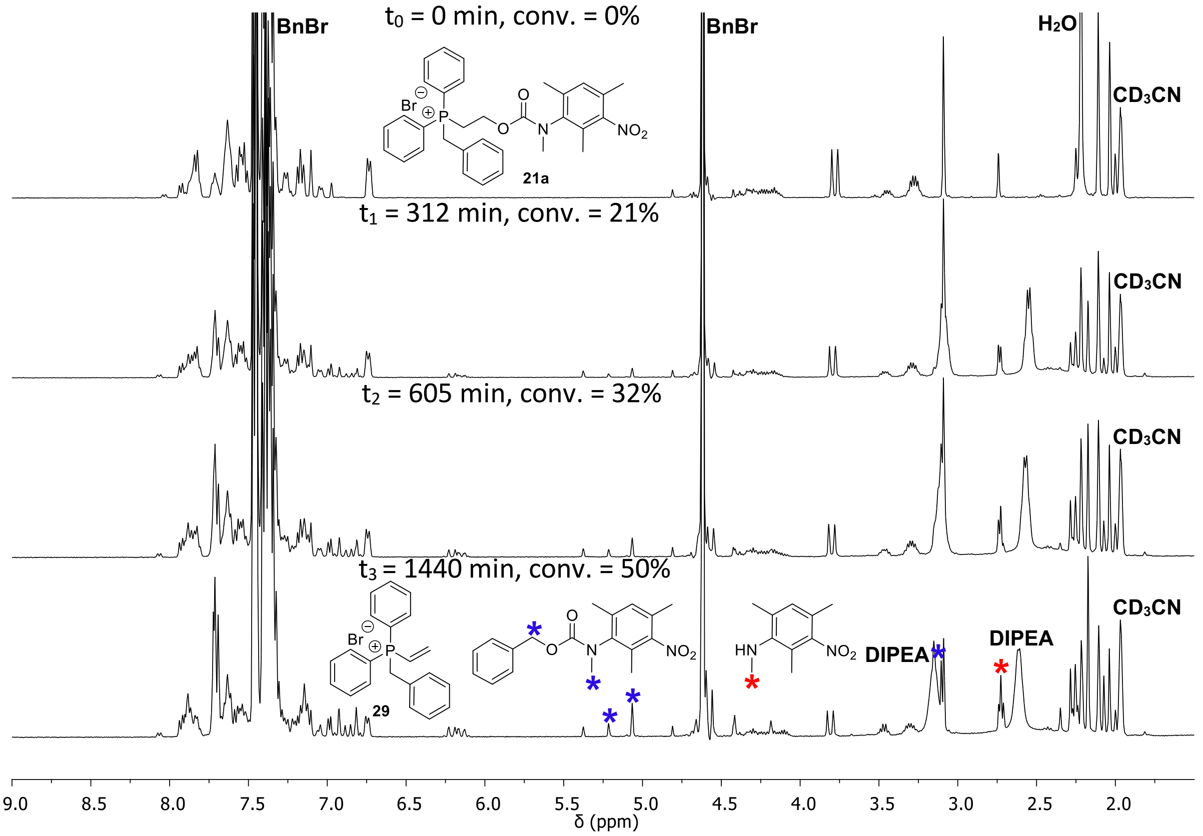

Figure S127. ^{1}H NMR spectra recorded overtime following the addition of 2 equivalents of DIPEA to a solution of alkylated **21** (MeCN-d_3, 400 MHz).

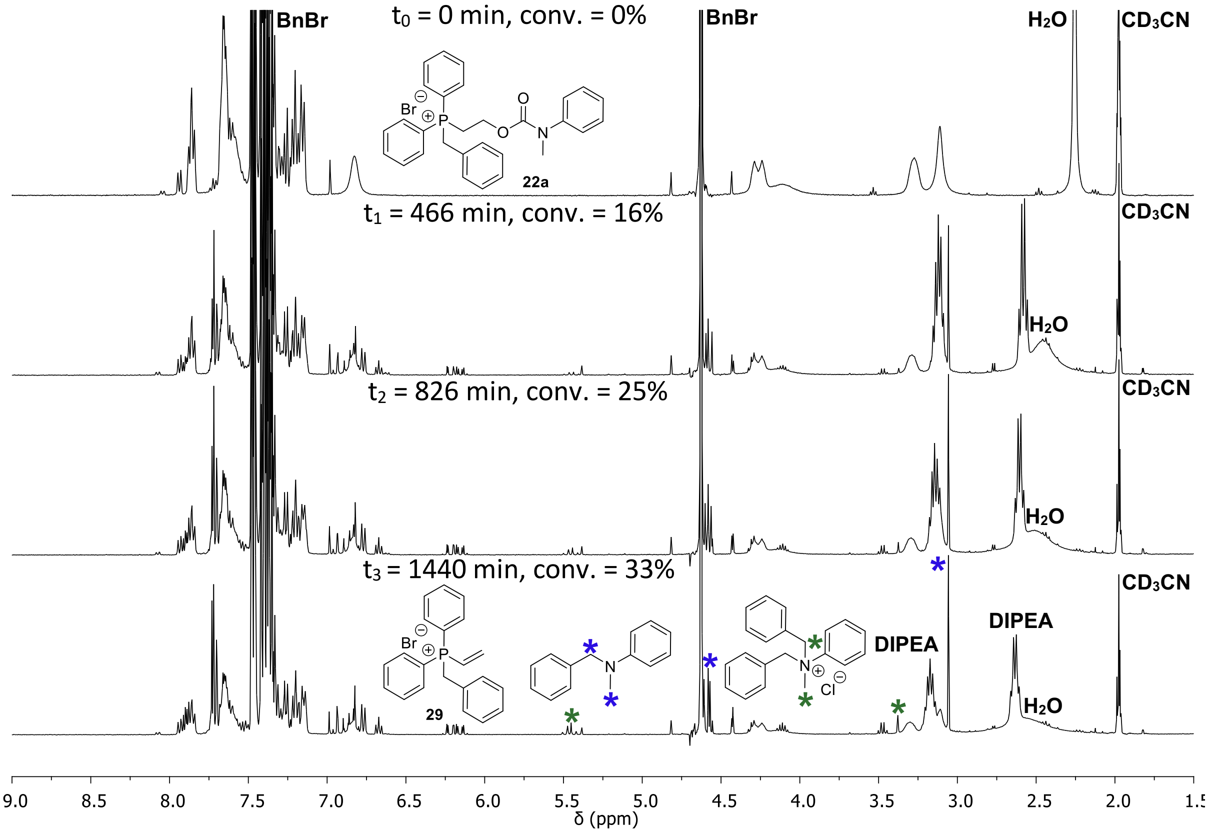

Figure S128. ^{1}H NMR spectra recorded overtime following the addition of 2 equivalents of DIPEA to a solution of alkylated **22** (MeCN-d_3, 400 MHz).

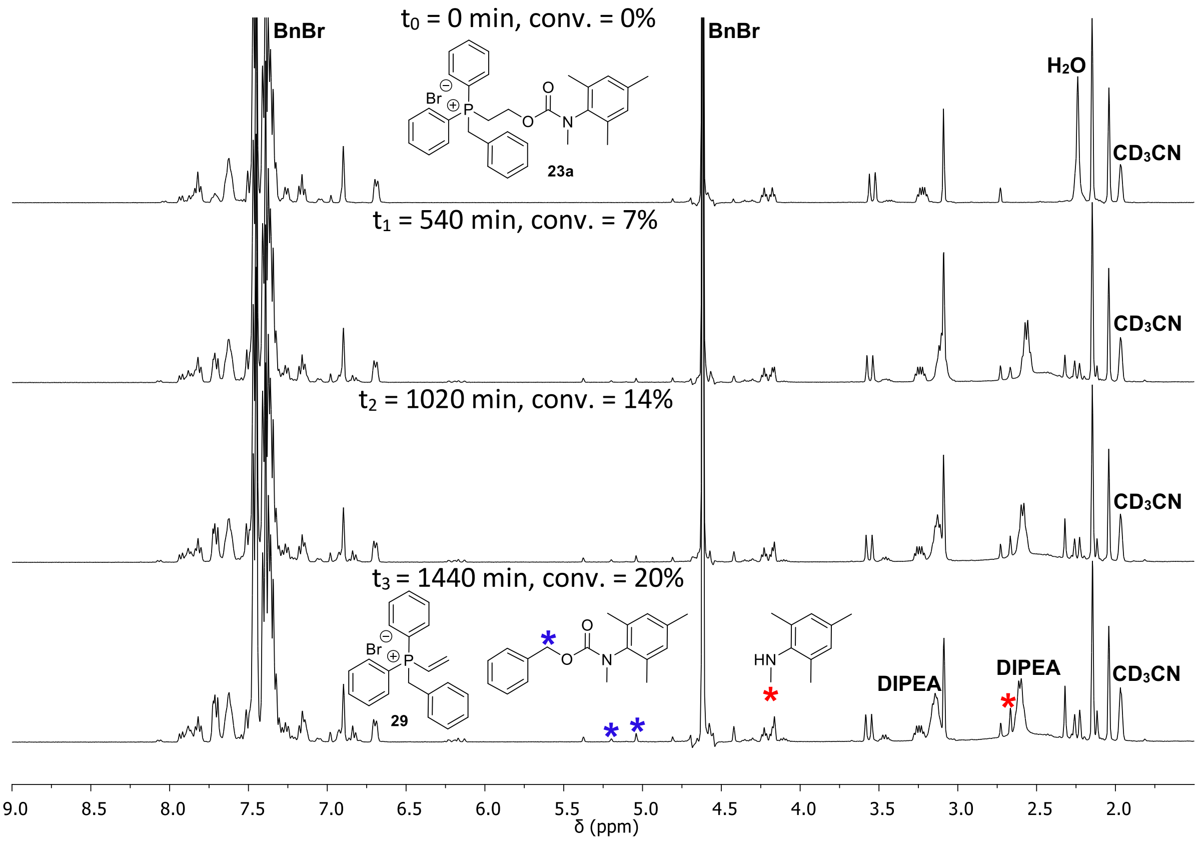

Figure S129. ^{1}H NMR spectra recorded overtime following the addition of 2 equivalents of DIPEA to a solution of alkylated **23** (MeCN-d_3, 400 MHz).

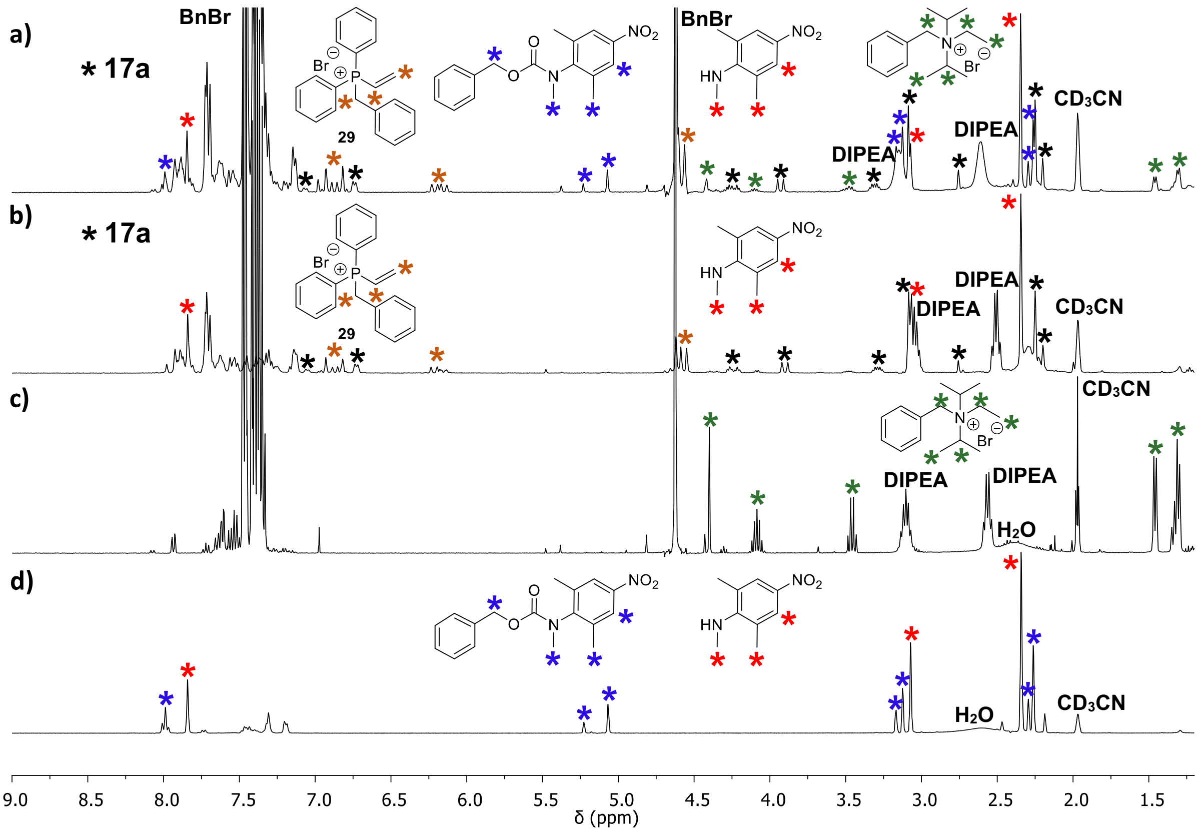

Figure S130. ^{1}H NMR spectra obtained **a)** 550 min after addition of 2 equivalents of DIPEA to a solution of alkylated **17 (17a)** and 9 equivalents of BnBr, **b)** 550 min after addition of 2 equivalents of DIPEA to a solution of **17a** without excess of BnBr, **c)** 5 days after addition of 5 equivalents of BnBr to a solution of DIPEA, **d)** for a mixture of *N*,2,6-trimethyl-4-nitroaniline and benzyl (2,6-dimethyl-4-nitrophenyl)(methyl)carbamate (MeCN-d_3, 400 MHz).

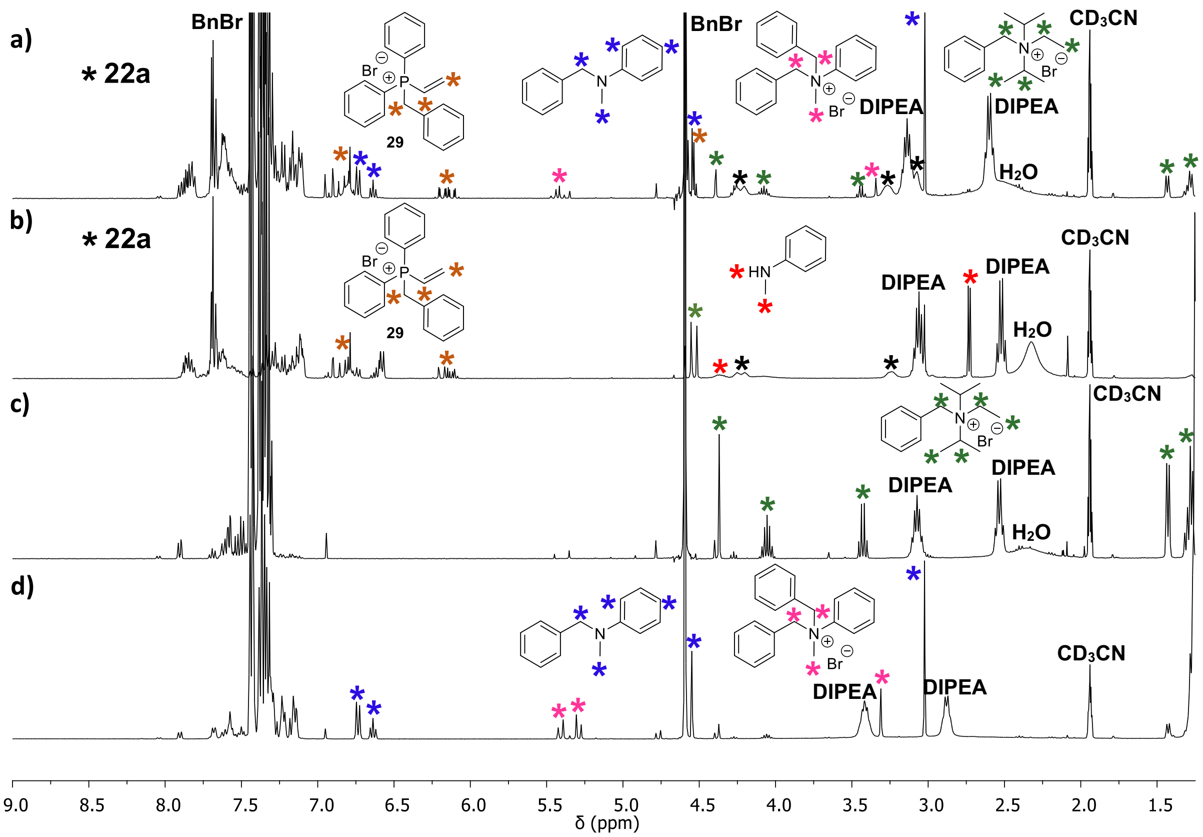

Figure S131. ^{1}H NMR spectra obtained **a)** 1440 min after addition of 2 equivalents of DIPEA to a solution of alkylated **22 (22a)** and 9 equivalents of BnBr, **b)** 1440 min after addition of 2 equivalents of DIPEA to a solution of **22a** without excess of BnBr, **c)** 5 days after addition of 5 equivalents of BnBr to a solution of DIPEA, **d)** 1440 min after addition of 10 equivalents of BnBr to a solution of *N*-methylaniline and 2 equivalents of DIPEA (MeCN-d_3, 400 MHz).

Solvent screening for self-immolative system 19

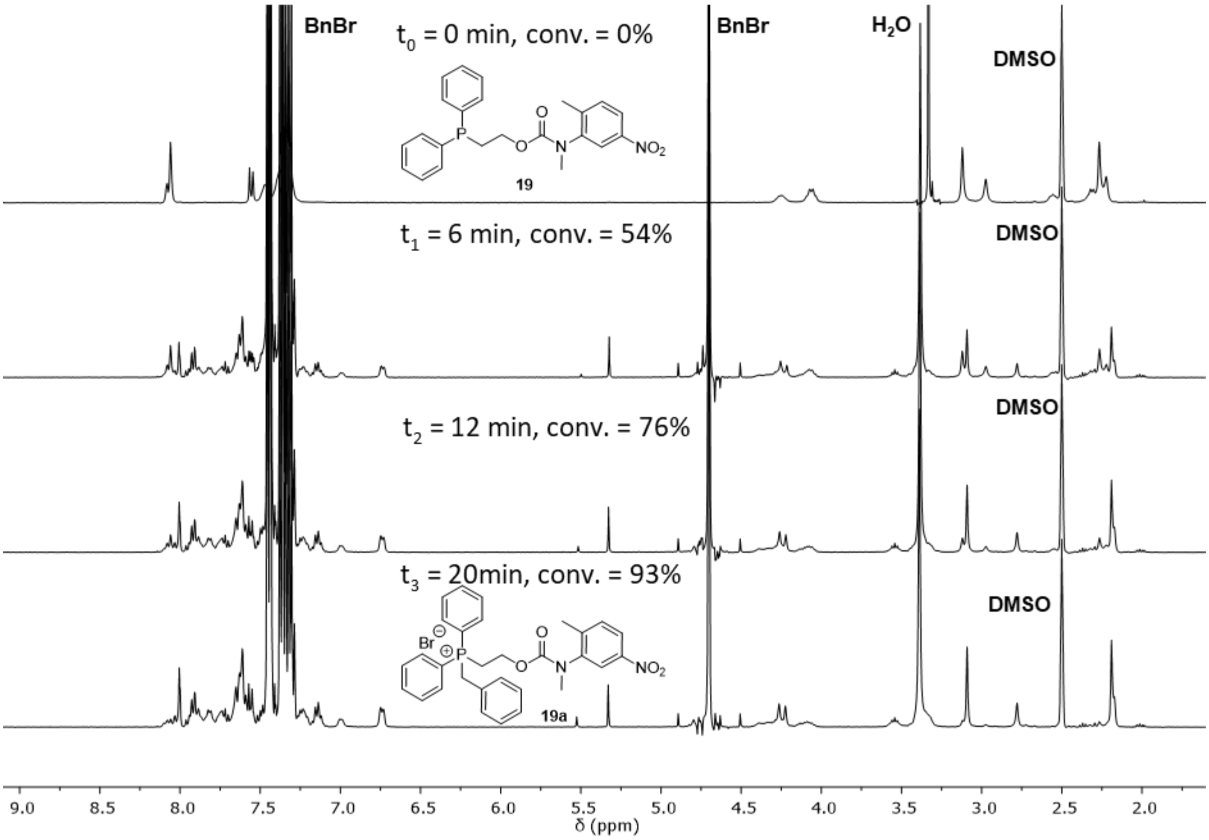

Figure S132. ^{1}H NMR spectra recorded overtime following the addition of 10 equivalents of BnBr to a solution of **19** (DMSO-d_6, 400 MHz).

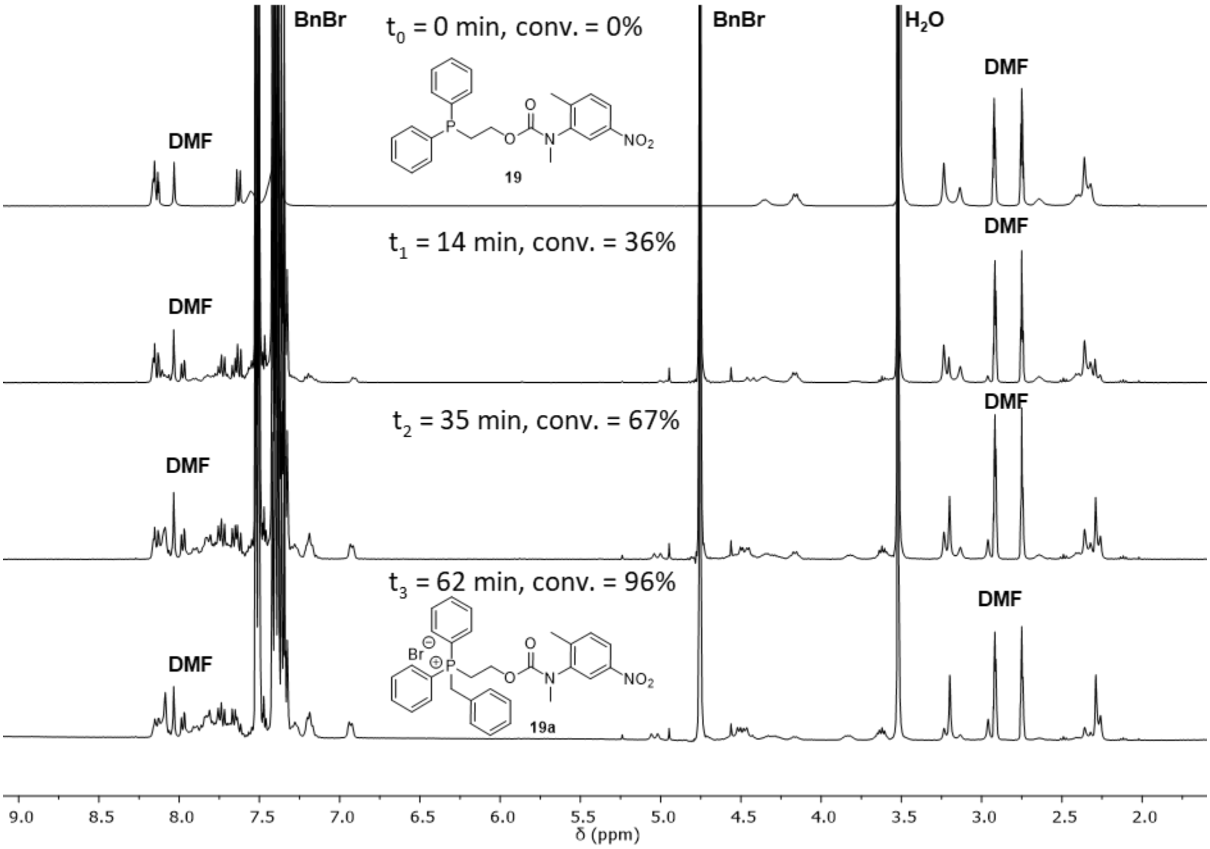

Figure S133. ^{1}H NMR spectra recorded overtime following the addition of 10 equivalents of BnBr to a solution of **19** (DMF-d_7, 400 MHz).

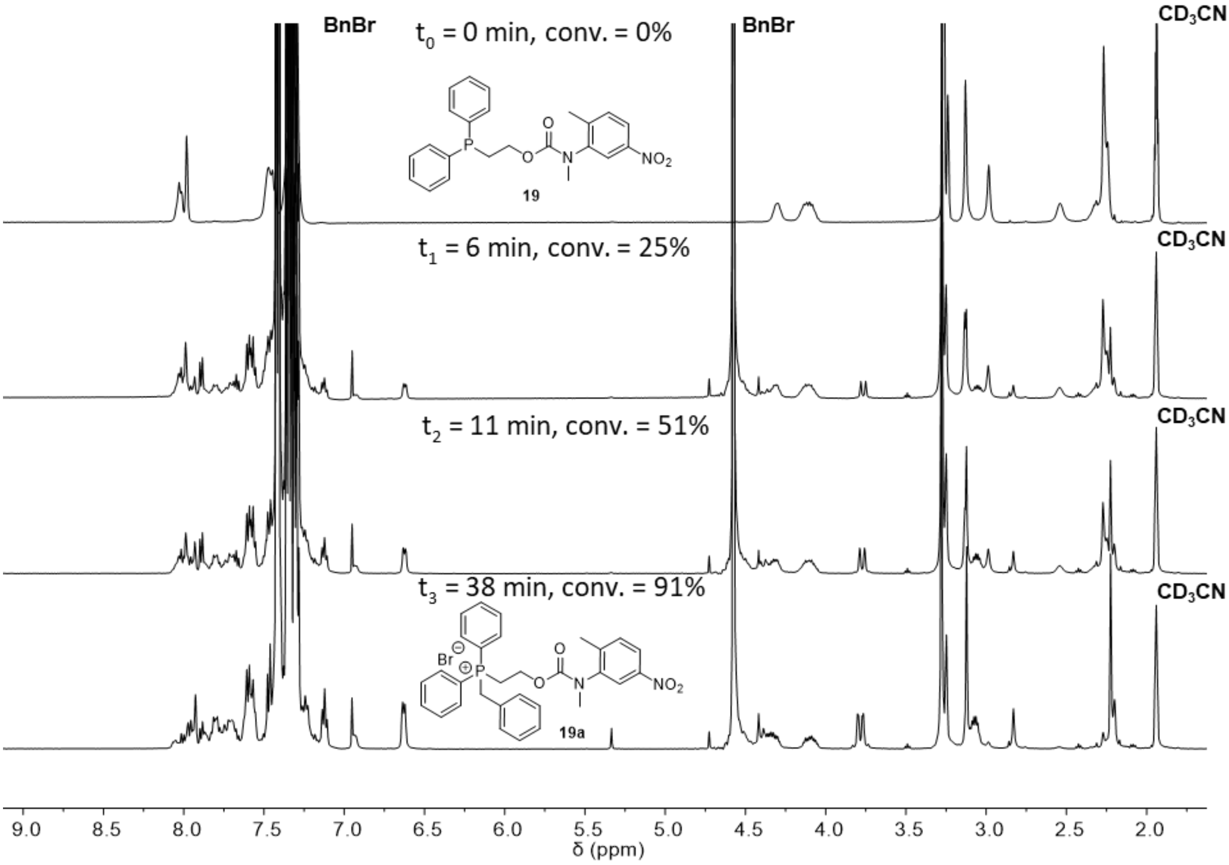

Figure S134. ^{1}H NMR spectra recorded overtime following the addition of 10 equivalents of BnBr to a solution of **19** (MeCN-d_3 with 10 % MeOD, 400 MHz).

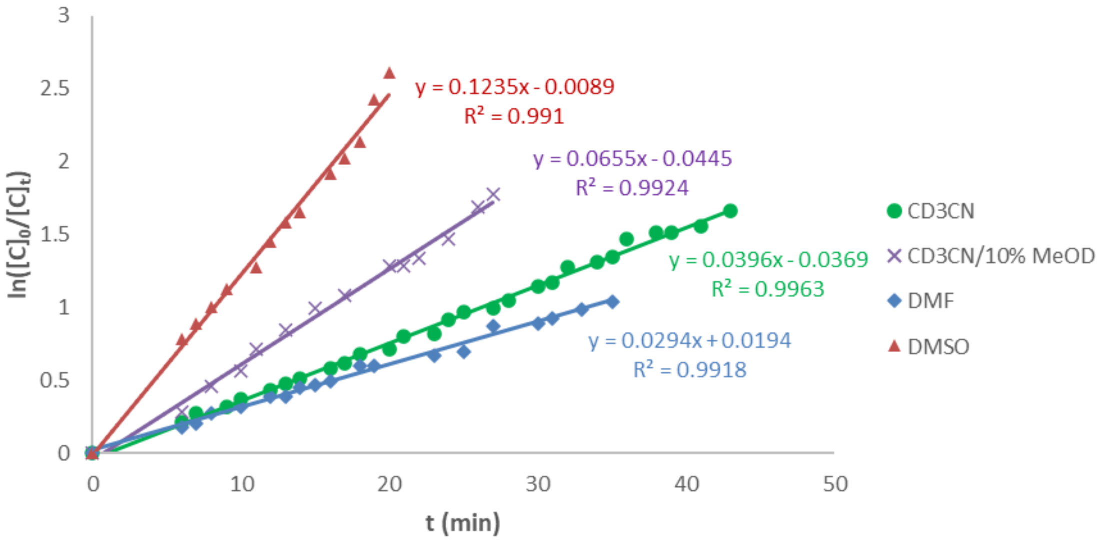

Figure S135. Kinetic plot of ln($[C]_0/[C]_t$) versus reaction time for the alkylation of **19** in MeCN-d_3, MeCN-d_3 with 10% MeOD, DMF-d_7 and DMSO-d_6.

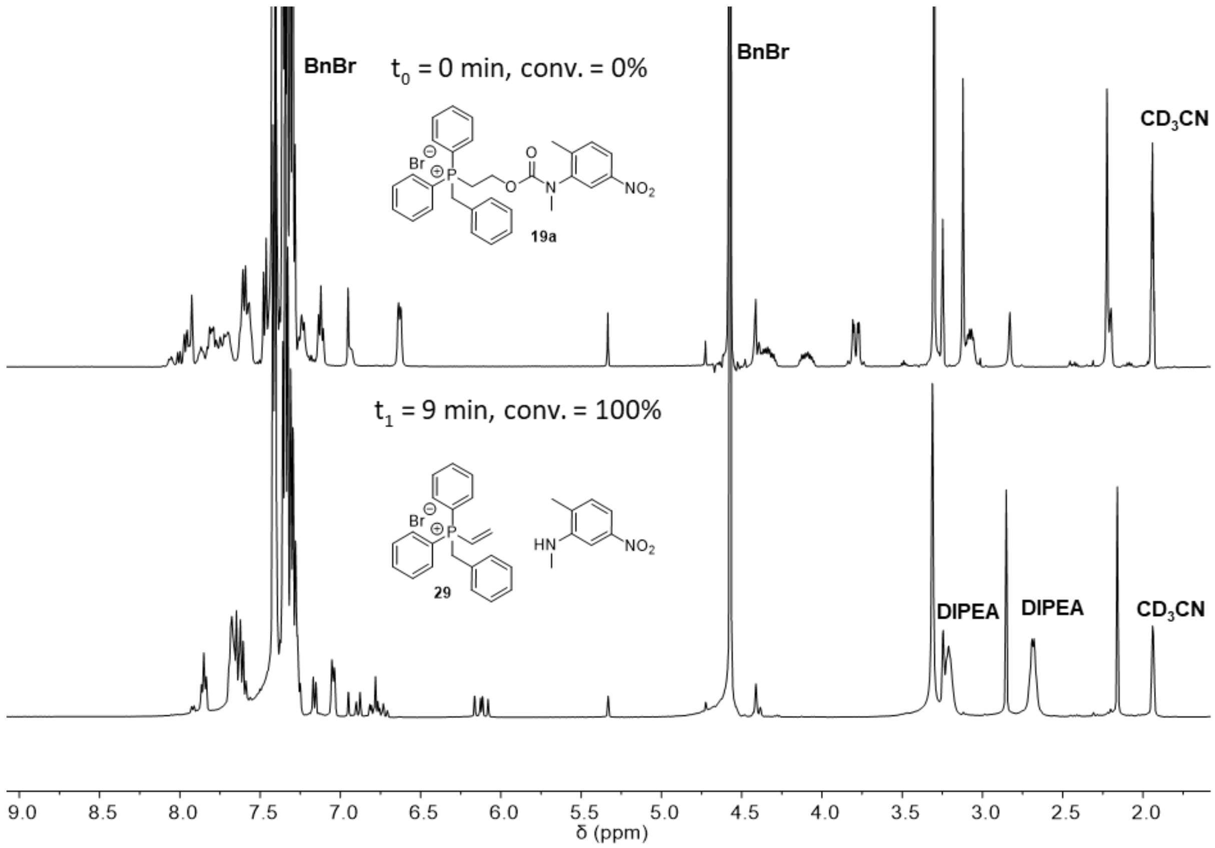

Figure S136. ^{1}H NMR spectra recorded overtime following the addition of 2 equivalents of DIPEA to a solution of alkylated **19a** (MeCN-d_3 with 10 % MeOD, 400 MHz).

One-pot alkylation/elimination data for the self-immolative systems 18-20

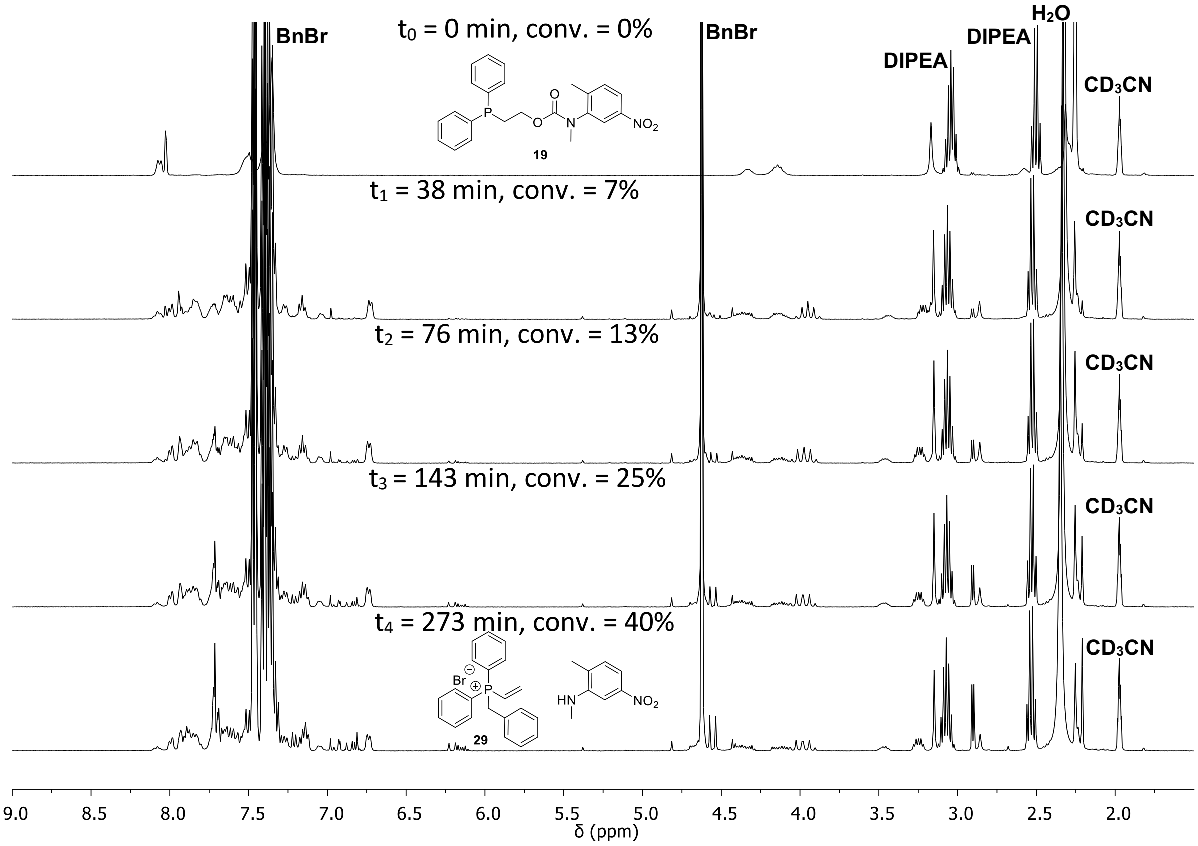

Figure S137. ^{1}H NMR spectra recorded overtime following the addition of 10 equivalents of BnBr to a solution of **19** and 2 equivalents of DIPEA in MeCN-d_3.

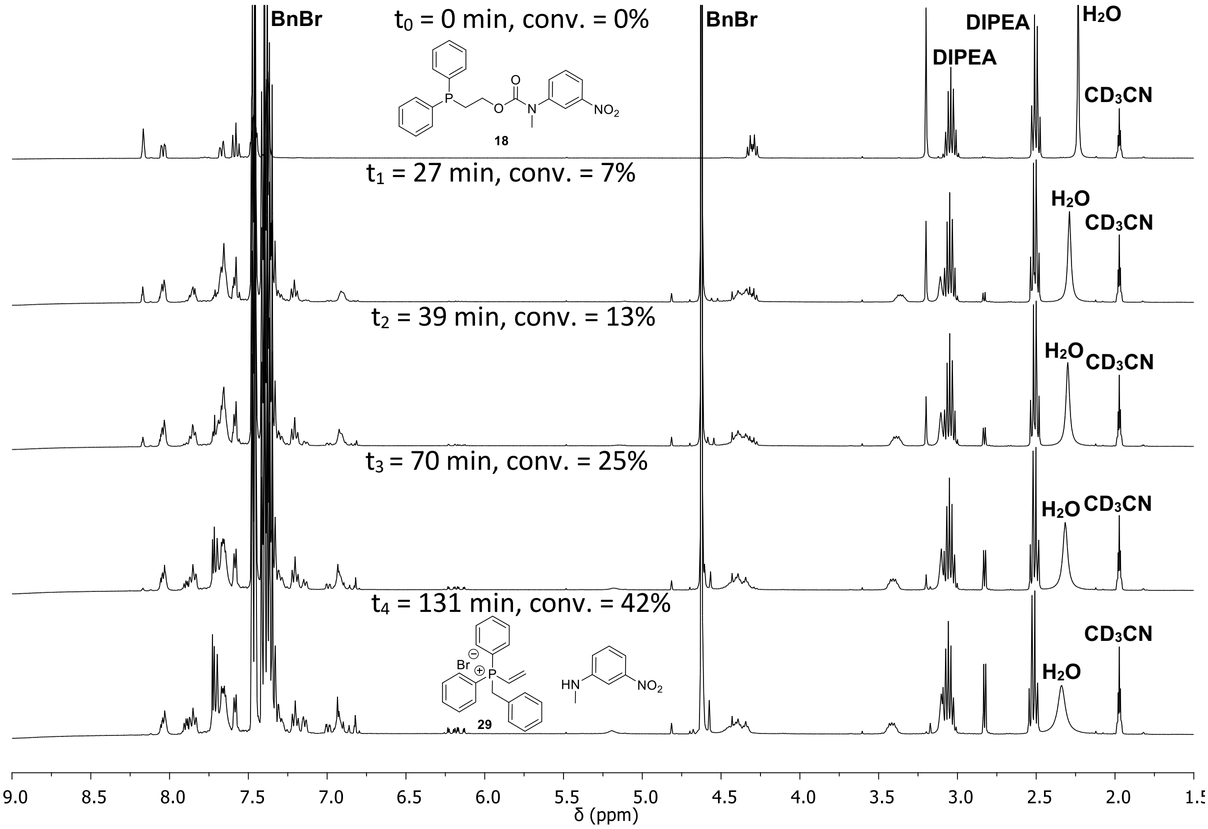

Figure S138. ^{1}H NMR spectra recorded overtime following the addition of 10 equivalents of BnBr to a solution of **18** and 2 equivalents of DIPEA in MeCN-d_3.

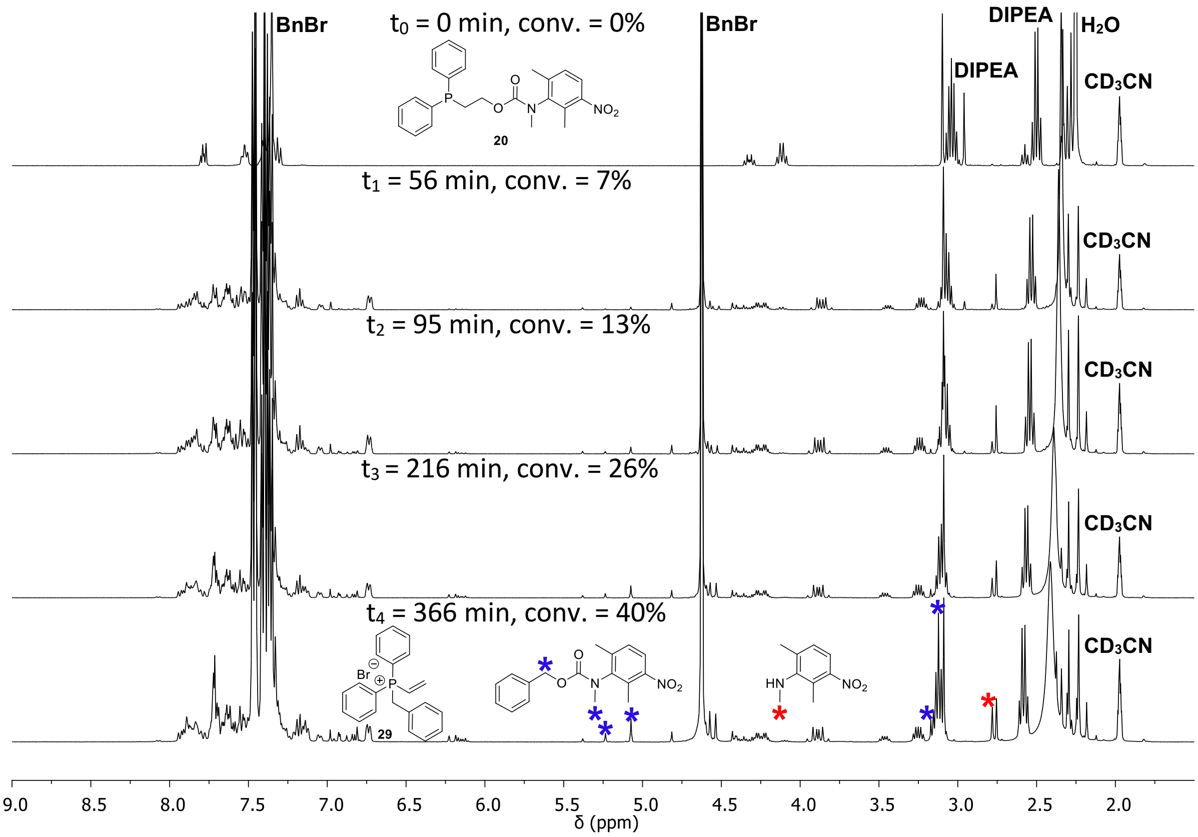

Figure S139. ^{1}H NMR spectra recorded overtime following the addition of 10 equivalents of BnBr to a solution of **20** and 2 equivalents of DIPEA in MeCN-d_3.

Table S9. Data obtained for the "one pot" reaction following the addition of 10 equivalents of BnBr to a solution of **18** and 2 equivalents of DIPEA in MeCN-d_3 at 20 °C calculated using ^{1}H NMR spectroscopy.

t(min)	SIE 18 (Area/1H)	SIE 18 (%)	Alkylated SIE 18 (Area/1H)	Alkylated SIE 18 (%)	Reporter group (Area/1H)	Reporter group (%)
0	0.55	100	0.00	0	0.00	0
6	0.45	80	0.11	20	0.00	0
7	0.42	77	0.13	23	0.00	0
8	0.40	71	0.16	28	0.00	1
10	0.37	67	0.18	32	0.00	1
11	0.35	64	0.20	35	0.01	1
13	0.34	60	0.21	38	0.01	2
14	0.31	57	0.23	42	0.01	2
15	0.29	53	0.25	44	0.01	2
17	0.28	50	0.26	47	0.02	3
18	0.26	47	0.27	49	0.02	4
20	0.25	45	0.28	51	0.02	4
21	0.23	42	0.29	53	0.02	4
22	0.22	40	0.30	55	0.03	5
24	0.21	37	0.32	57	0.03	6
25	0.19	35	0.32	58	0.04	7
27	0.18	33	0.33	60	0.04	7

28	0.17	31	0.34	61	0.05	8
29	0.16	29	0.34	62	0.05	9
31	0.15	28	0.35	63	0.05	9
32	0.14	25	0.36	64	0.06	10
34	0.13	24	0.36	65	0.06	11
35	0.13	23	0.36	65	0.06	12
36	0.12	22	0.36	66	0.07	12
38	0.11	21	0.37	67	0.07	13
39	0.11	20	0.37	67	0.07	13
40	0.10	18	0.37	68	0.08	14
42	0.09	17	0.37	69	0.08	15
43	0.09	16	0.38	68	0.09	16
45	0.08	15	0.38	69	0.09	16
46	0.08	14	0.38	70	0.09	16
47	0.07	13	0.38	69	0.10	18
49	0.07	13	0.38	70	0.10	18
50	0.06	12	0.38	69	0.10	19
52	0.06	11	0.38	69	0.11	20
53	0.06	10	0.38	69	0.11	20
59	0.04	8	0.38	70	0.12	22
61	0.04	7	0.38	70	0.12	23
62	0.04	7	0.38	69	0.13	24
63	0.04	7	0.38	69	0.13	24
65	0.04	7	0.38	68	0.14	25
66	0.03	6	0.37	69	0.14	25
68	0.03	6	0.37	69	0.14	26
69	0.03	6	0.37	68	0.14	26
70	0.03	5	0.37	68	0.14	27
72	0.03	5	0.37	67	0.15	28
75	0.02	4	0.36	67	0.15	29
76	0.02	4	0.36	67	0.16	29
78	0.02	4	0.36	67	0.16	30
79	0.02	3	0.36	66	0.16	31
81	0.02	3	0.36	66	0.16	31
82	0.02	3	0.36	66	0.17	31
85	0.02	3	0.35	66	0.17	31
86	0.01	3	0.35	65	0.17	32
88	0.01	3	0.35	65	0.17	33
89	0.01	3	0.34	64	0.18	33
91	0.01	3	0.34	64	0.18	34
92	0.01	2	0.34	64	0.18	34
95	0.01	2	0.34	64	0.18	34
97	0.01	2	0.34	63	0.18	35
98	0.01	1	0.34	64	0.18	35
107	0.01	1	0.32	61	0.20	38
116	0.00	1	0.31	60	0.20	39

131	0.00	0	0.30	58	0.21	42
146	0.00	0	0.29	56	0.22	44
161	0.00	0	0.28	55	0.23	45
177	0.00	0	0.27	52	0.24	48
192	0.00	0	0.26	51	0.25	49
206	0.00	0	0.26	50	0.25	50
221	0.00	0	0.25	50	0.25	50
237	0.00	0	0.25	49	0.25	51
252	0.00	0	0.24	48	0.26	52
267	0.00	0	0.23	47	0.26	53
282	0.00	0	0.23	46	0.26	54
298	0.00	0	0.22	45	0.27	55

Table S10. Data obtained for the "one pot" reaction following the addition of 10 equivalents of BnBr to a solution of **19** and 2 equivalents of DIPEA in MeCN-d_3 at 20 °C calculated using ^{1}H NMR spectroscopy.

t(min)	SIE 19 (Area/2H)	SIE 19 (%)	Alkylated SIE 19 (Area/2H)	Alkylated SIE 19 (%)	Reporter group (Area/2H)	Reporter group (%)
0	0.87	100	0.00	0	0.00	0
7	0.70	77	0.21	23	0.00	0
8	0.64	73	0.24	27	0.00	0
10	0.61	69	0.28	31	0.00	0
11	0.58	65	0.31	35	0.00	0
13	0.57	63	0.33	36	0.01	1
14	0.52	58	0.36	40	0.01	1
15	0.51	56	0.39	43	0.01	1
17	0.48	53	0.42	46	0.01	1
18	0.48	51	0.42	45	0.04	4
20	0.46	49	0.43	46	0.04	4
21	0.44	47	0.45	48	0.04	4
22	0.43	46	0.47	50	0.04	4
24	0.41	43	0.50	53	0.04	4
25	0.37	40	0.52	56	0.04	4
27	0.37	39	0.52	55	0.05	5
28	0.36	38	0.54	57	0.05	5
29	0.34	36	0.55	59	0.05	5
31	0.31	34	0.56	61	0.05	5
32	0.30	32	0.57	61	0.06	6
34	0.29	32	0.57	62	0.06	7
35	0.28	30	0.59	63	0.06	6
36	0.27	29	0.60	65	0.06	6
38	0.26	28	0.61	65	0.07	7
39	0.26	27	0.62	65	0.07	7
41	0.25	26	0.63	66	0.08	8
42	0.24	25	0.63	66	0.09	9

43	0.21	22	0.65	68	0.09	9
45	0.21	22	0.65	68	0.09	9
46	0.20	21	0.66	69	0.09	9
48	0.18	19	0.67	71	0.09	10
49	0.18	19	0.67	71	0.09	10
51	0.18	19	0.67	71	0.09	10
52	0.18	19	0.68	72	0.09	9
53	0.18	19	0.68	72	0.09	9
56	0.16	17	0.68	73	0.09	10
58	0.16	17	0.68	73	0.09	10
59	0.14	15	0.69	75	0.09	10
60	0.13	14	0.70	76	0.09	10
62	0.13	14	0.70	75	0.10	11
63	0.13	14	0.70	75	0.10	11
65	0.13	14	0.70	75	0.10	11
66	0.12	13	0.70	76	0.10	11
67	0.12	13	0.70	76	0.10	11
69	0.11	12	0.70	77	0.10	11
70	0.10	11	0.70	77	0.11	12
72	0.10	11	0.70	77	0.11	12
73	0.10	11	0.71	77	0.11	12
74	0.10	11	0.71	77	0.11	12
76	0.09	10	0.71	77	0.12	13
77	0.09	10	0.71	76	0.13	14
79	0.08	9	0.71	77	0.13	14
80	0.08	9	0.71	77	0.13	14
81	0.08	9	0.71	77	0.13	14
83	0.08	9	0.70	77	0.13	14
84	0.08	9	0.70	76	0.14	15
86	0.07	8	0.70	77	0.14	15
87	0.07	8	0.70	77	0.14	15
88	0.07	8	0.70	77	0.14	15
90	0.07	8	0.70	77	0.14	15
91	0.07	8	0.70	76	0.15	16
93	0.07	8	0.70	76	0.15	16
94	0.07	8	0.70	76	0.15	16
95	0.07	8	0.70	76	0.15	16
97	0.06	7	0.70	76	0.16	17
98	0.05	5	0.70	77	0.16	18
100	0.05	5	0.70	77	0.16	18
101	0.05	6	0.69	77	0.16	18
102	0.05	6	0.69	77	0.16	18
104	0.05	6	0.69	77	0.16	18
105	0.05	6	0.69	77	0.16	18
107	0.05	6	0.69	77	0.16	18
108	0.04	4	0.69	77	0.17	19

109	0.03	3	0.69	78	0.17	19
111	0.03	3	0.69	78	0.17	19
112	0.03	3	0.68	76	0.18	20
114	0.03	3	0.68	76	0.18	20
115	0.03	3	0.68	76	0.18	20
117	0.03	3	0.68	76	0.18	20
118	0.03	3	0.68	76	0.19	21
119	0.02	2	0.68	76	0.19	21
121	0.02	2	0.68	76	0.19	21
122	0.02	2	0.68	76	0.20	22
124	0.02	2	0.68	76	0.20	22
125	0.01	1	0.68	76	0.20	22
126	0.01	1	0.68	76	0.20	22
128	0.01	1	0.68	76	0.20	22
129	0.01	1	0.67	76	0.20	23
131	0.00	0	0.67	76	0.21	24
132	0.00	0	0.67	76	0.21	24
133	0.00	0	0.67	76	0.21	24
135	0.00	0	0.67	76	0.21	24
136	0.00	0	0.67	75	0.22	25
138	0.00	0	0.67	75	0.22	25
139	0.00	0	0.67	75	0.22	25
140	0.00	0	0.67	75	0.22	25
142	0.00	0	0.67	75	0.22	25
143	0.00	0	0.66	75	0.22	25
145	0.00	0	0.66	74	0.23	26
146	0.00	0	0.66	74	0.23	26
156	0.00	0	0.66	73	0.24	27
187	0.00	0	0.60	68	0.28	32
216	0.00	0	0.58	65	0.31	35
273	0.00	0	0.53	60	0.35	40

Table S11. Data obtained for the "one pot" reaction following the addition of 10 equivalents of BnBr to a solution of **20** and 2 equivalents of DIPEA in MeCN-d_3 at 20 °C calculated using ^{1}H NMR spectroscopy.

t(min)	SIE 20 (Area/2H)	SIE 20 (%)	Alkylated SIE 20 (Area/2H)	Alkylated SIE 20 (%)	Reporter group (Area/2H)	Reporter group (%)
0	0.79	100	0.00	0	0.00	0
7	0.62	78	0.17	22	0.00	0
8	0.59	75	0.20	25	0.00	0
10	0.56	72	0.22	28	0.00	0
11	0.53	68	0.25	32	0.00	0
13	0.52	66	0.27	34	0.00	0
14	0.49	63	0.28	36	0.01	1
16	0.46	58	0.32	41	0.01	1

17	0.44	56	0.34	43	0.01	1
18	0.41	53	0.36	46	0.01	1
20	0.40	50	0.39	49	0.01	1
21	0.39	49	0.39	49	0.02	3
23	0.37	46	0.41	51	0.03	4
24	0.35	44	0.42	52	0.03	4
25	0.34	42	0.44	55	0.03	4
27	0.32	40	0.45	56	0.03	4
28	0.31	39	0.46	58	0.03	4
30	0.29	37	0.48	60	0.03	4
31	0.28	34	0.49	61	0.04	5
32	0.27	33	0.51	62	0.04	5
34	0.25	31	0.51	64	0.04	5
35	0.24	30	0.53	65	0.04	5
37	0.23	29	0.53	66	0.04	5
38	0.22	27	0.54	68	0.04	5
39	0.21	26	0.54	68	0.05	6
41	0.21	25	0.56	69	0.05	6
42	0.19	24	0.56	70	0.05	6
44	0.19	23	0.57	70	0.05	6
45	0.17	22	0.58	72	0.05	6
46	0.17	22	0.58	72	0.05	6
48	0.16	20	0.60	74	0.05	6
49	0.16	19	0.60	74	0.05	6
51	0.15	18	0.60	75	0.05	6
52	0.15	18	0.61	76	0.05	6
53	0.13	16	0.62	78	0.05	6
55	0.13	16	0.63	78	0.05	6
56	0.12	15	0.63	78	0.06	7
58	0.12	15	0.64	78	0.06	7
59	0.12	14	0.64	77	0.07	8
60	0.11	14	0.64	78	0.07	9
62	0.10	13	0.64	79	0.07	9
63	0.10	13	0.64	79	0.07	9
65	0.10	13	0.64	79	0.07	9
66	0.09	11	0.66	81	0.07	9
67	0.09	11	0.66	81	0.07	9
69	0.09	11	0.66	81	0.07	9
70	0.09	10	0.66	80	0.08	10
72	0.08	9	0.66	81	0.08	10
73	0.07	9	0.66	81	0.08	10
74	0.07	9	0.66	81	0.08	10
76	0.07	9	0.66	80	0.09	11
77	0.07	8	0.67	81	0.09	11
79	0.06	7	0.67	82	0.09	11
80	0.06	7	0.68	82	0.09	11

81	0.06	7	0.68	81	0.10	12
83	0.06	7	0.68	81	0.10	12
84	0.05	6	0.68	82	0.10	12
86	0.04	5	0.68	83	0.10	12
87	0.04	5	0.68	83	0.10	12
88	0.04	5	0.68	82	0.11	13
90	0.04	5	0.68	82	0.11	13
91	0.04	5	0.68	82	0.11	13
93	0.04	5	0.68	82	0.11	13
94	0.04	5	0.68	82	0.11	13
95	0.03	4	0.68	83	0.11	13
97	0.03	4	0.68	83	0.11	13
98	0.03	4	0.68	83	0.11	13
100	0.03	4	0.68	83	0.11	13
101	0.03	4	0.68	83	0.11	13
103	0.03	3	0.68	83	0.11	13
104	0.03	3	0.68	82	0.12	15
105	0.03	3	0.68	82	0.12	15
107	0.03	3	0.68	82	0.12	15
108	0.03	3	0.68	82	0.12	15
110	0.03	3	0.68	82	0.12	15
111	0.03	3	0.68	82	0.12	15
112	0.03	3	0.68	82	0.12	15
114	0.02	2	0.68	83	0.12	15
115	0.02	2	0.68	82	0.13	16
117	0.02	2	0.67	82	0.13	16
118	0.02	2	0.67	82	0.13	16
119	0.02	2	0.67	81	0.14	17
121	0.02	2	0.67	81	0.14	17
122	0.02	2	0.67	81	0.14	17
124	0.02	2	0.67	81	0.14	17
125	0.02	2	0.67	81	0.14	17
126	0.02	2	0.67	81	0.14	17
128	0.02	2	0.67	80	0.15	18
129	0.02	2	0.67	80	0.15	18
131	0.02	2	0.67	80	0.15	18
132	0.02	2	0.66	80	0.15	18
133	0.02	2	0.66	80	0.15	18
135	0.02	2	0.66	80	0.15	18
136	0.02	2	0.66	80	0.15	18
138	0.02	2	0.66	80	0.15	18
139	0.02	2	0.66	80	0.15	18
140	0.02	2	0.66	80	0.15	18
142	0.02	2	0.66	80	0.15	18
143	0.02	2	0.66	79	0.16	19
145	0.00	0	0.65	80	0.16	20

146	0.00	0	0.65	80	0.16	20
156	0.00	0	0.65	80	0.16	20
186	0.00	0	0.64	77	0.19	23
216	0.00	0	0.63	74	0.22	26
246	0.00	0	0.57	72	0.22	28
276	0.00	0	0.55	71	0.23	29

UV-visible data for self-immolative system 19

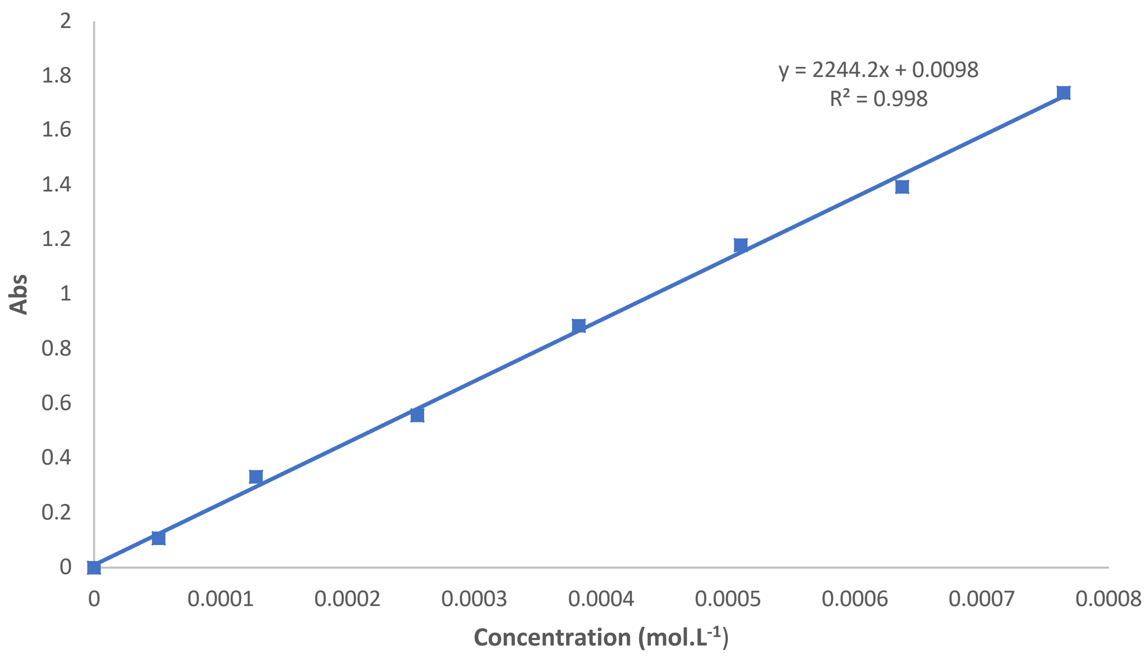

Figure S140. UV-visible spectroscopy calibration curve for the reporter group *N*,2-dimethyl-5-nitroaniline.
in MeCN.

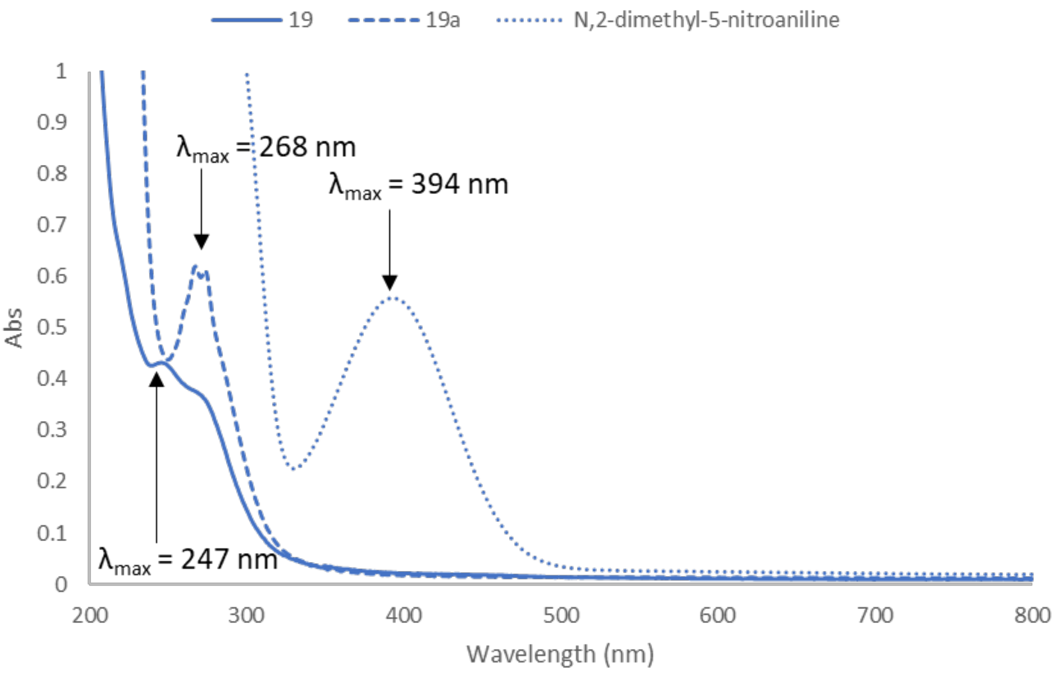

Figure S141. UV-visible spectra of **19**, **19a** and the reporter group *N*,2-dimethyl-5-nitroaniline individually in MeCN at C = $2.5.10^{-3}$ mol.L^{-1}

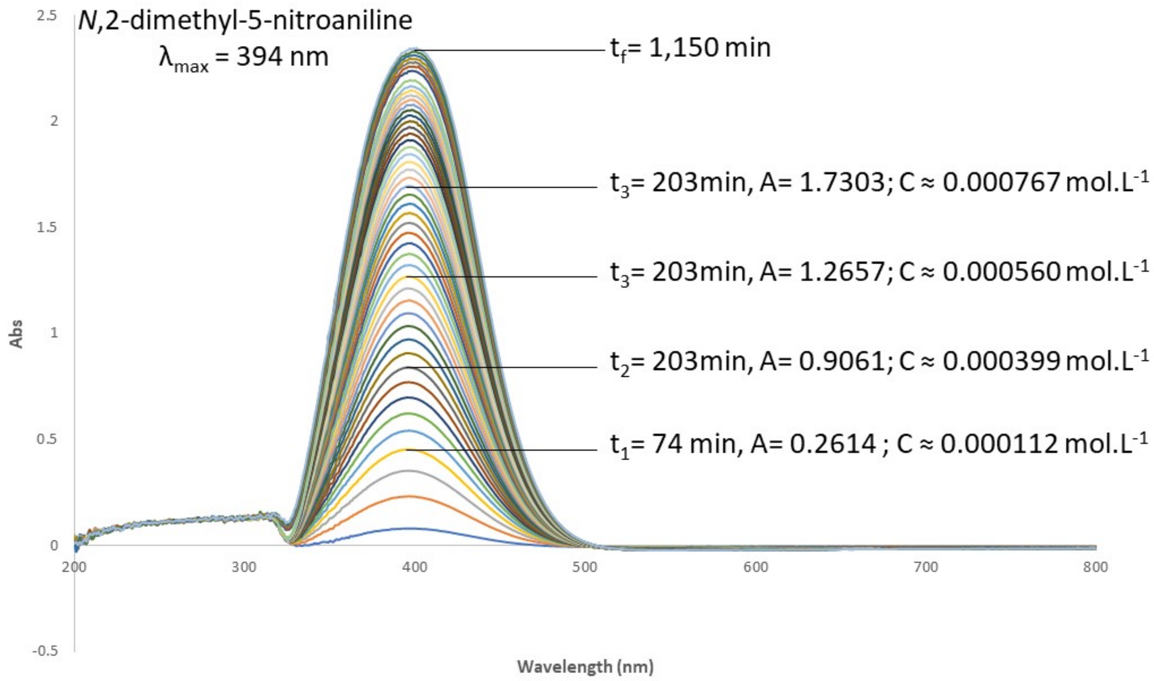

Figure S142. UV-visible spectra displaying the release of *N*,2-dimethyl-5-nitroaniline from alkylated **19** in MeCN ([alkylated 3]$_0$ = 2.5.10^{-3} mol.L^{-1}) upon the addition of 2 equivalent of DIPEA.

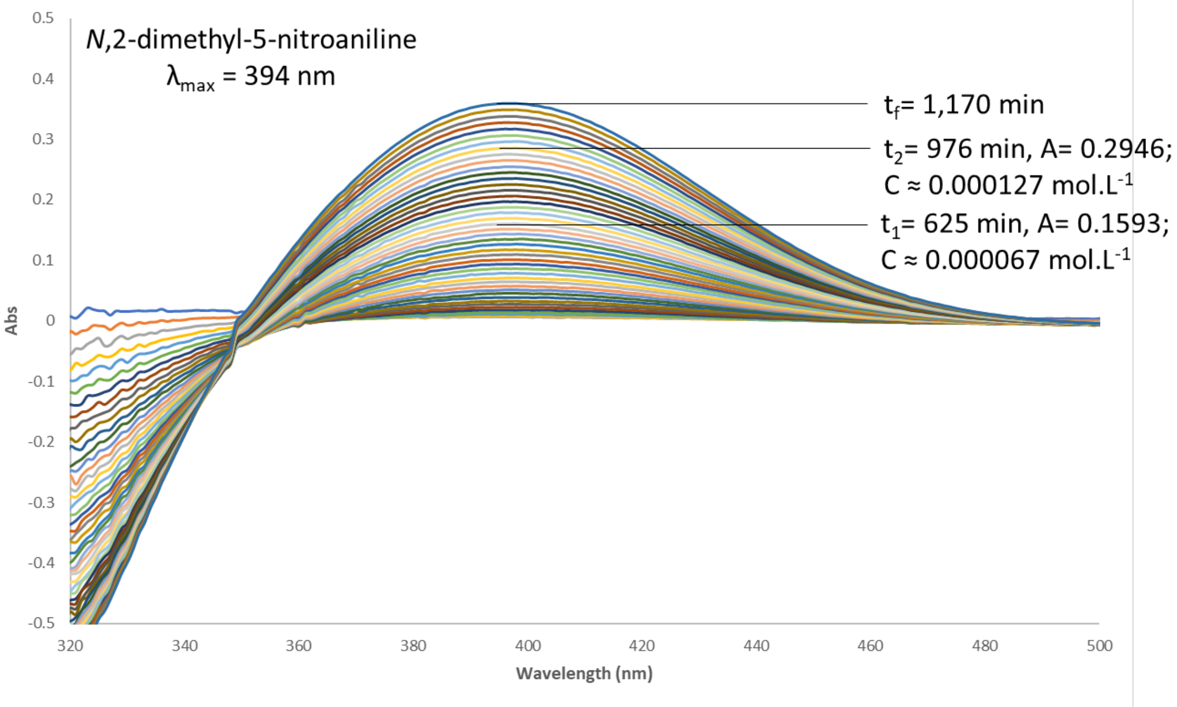

Figure S143. UV-visible spectra displaying the release of *N*,2-dimethyl-5-nitroaniline following the addition of 1.85 equivalents of BnBr to a solution of **19** and 2 equivalents of DIPEA in MeCN ([3]$_0$ = 2.5.10^{-3} mol.L^{-1})

Reference

[1] Shanan-Atidi, H.; Bar-Eli, K. H. A convenient method for obtaining free energies of activation by the coalescence temperature of an unequal doublet. *J. Phys. Chem.* **1970**, 74, 961-963.
